# The burden and trends of late-onset multiple sclerosis, Parkinson’s disease, Alzheimer’s disease and other dementias among adults aged 55 and older, spanning from 1990 to 2021, with projections through 2050

**DOI:** 10.1007/s10072-026-08986-6

**Published:** 2026-04-18

**Authors:** Xiaojun Liu, Xiujuan Mi, Xinyuan Yu, Ning Deng, Yuanhong Lei, Jianzhong Shu, Jun Tang

**Affiliations:** 1 Department of Brain Disease, Chongqing Hospital of Traditional Chinese Medicine, Chongqing, 400021 China; 2 Department of Rheumatology and Immunology, The First Affiliated Hospital (Southwest Hospital) of Army Medical University, Chongqing, 400038 China

**Keywords:** Late-onset multiple sclerosis, Parkinson’s disease, Alzheimer’s disease and other dementias, Global burden of disease, Socio-demographic index

## Abstract

**Objectives:**

This study investigates the global burden of common central nervous system (CNS) disorders—including late-onset multiple sclerosis (LOMS), Parkinson’s disease (PD), Alzheimer’s disease and other dementias (ADOD)—among adults aged 55 years and older, covering the period from 1990 to 2021 with projections extending through 2050.

**Methods:**

Data from the Global Burden of Disease (GBD) Study 2021, spanning the years 1990 to 2021, were analyzed to evaluate the number of incident and prevalent cases, deaths, disability-adjusted life years (DALYs), years lived with disability (YLDs), years of life lost (YLLs), and their corresponding age-standardized rates. These metrics were stratified by socio-demographic index (SDI) regions, sex, and age groups. Future trends were forecasted using the Bayesian age–period–cohort (BAPC) model up to the year 2036 and the Autoregressive Integrated Moving Average (ARIMA) model up to 2050.

**Results:**

In 2021, global cases reached 0.76 million for LOMS, 10.76 million for PD, and 20.84 million for ADOD. ADOD exhibited the highest burden across all metrics. Females bore greater LOMS and ADOD burden; males bore higher PD burden. High SDI regions had highest LOMS burden; high-middle SDI regions led in PD and ADOD burden. Population growth was the primary burden driver. China faced the highest PD and ADOD burden due to rapid aging.

**Conclusion:**

Urgent health policies must target elderly populations with sex-specific approaches—focusing on women for LOMS/ADOD and men for PD—alongside strategies for high-risk demographic groups.

**Supplementary Information:**

The online version contains supplementary material available at 10.1007/s10072-026-08986-6.

## Contributions to the literature

1. This paper provides a comparative analysis on the burden of disease of LOMS, PD, ADOD in populations of older adults. By bringing these clinically discrete neurological disorders into a common analytic model, it has defined the different epidemiologic patterns of their occurrence, such as age of onset peak and sex distributions, and have also defined common demographic factors in increasing burden.
2. This study measures the effects of population growth, aging and epidemiological change on disease burden trend by the use of decomposition analysis and complementary modeling. The projections on using both BAPC and ARIMA models show that the age-standardized rates might stabilize or even decrease, but absolute cases will still increase significantly up to 2050, especially ADOD based on the strong demographic momentum.
3. These results show policy-relevant differences in the disease burden based on geographic areas, level of the socio-demographic index, and gender. Among the findings are high PD mortality in low-SDI areas and concentration of the load of LOMS in the high-income nations as well as disproportionately greater absolute burden in China owing to the scale of its demographics. These lessons can be used to inform additional equity-based intervention in health policies.

## Introduction

Parkinson’s disease (PD) and Alzheimer’s disease (AD) are the two most common neurological disorders worldwide. The global population with PD exceeds 6 million, and its prevalence has increased 2.5-fold compared to the previous generation, positioning PD among the leading causes of neurological dysfunction [1, 2]. Alzheimer’s disease is the primary cause of dementia and is becoming one of the most costly, fatal, and socially burdensome diseases of this century. Recent estimates suggest that the prevalence of dementia in Europe is expected to double by 2050 [3].

Multiple sclerosis (MS) is an immune-mediated inflammatory demyelinating disease of the central nervous system (CNS), with age of onset being a key factor influencing its pathological processes and progression. The disease typically manifests between the ages of 20 and 40, which is defined as normal-onset multiple sclerosis (NOMS). We now know that MS starting after age 50–55, referred to as late-onset MS (LOMS), comprises up to 12% of MS cases [4]. Compared to NOMS, LOMS often exhibits more neurodegenerative characteristics and shows significant clinical differences: about 32% of LOMS patients had a progressive disease course [5], experience poorer recovery after relapses, attain higher levels of disability, and respond relatively less effectively to disease-modifying therapies (DMT) [6, 7]. Currently, research data on LOMS remain limited [8]. In-depth epidemiological studies on LOMS could help more accurately assess its growing disease burden.

The global population is ageing significantly and it is altering the epidemiological profile to be oriented more towards the prevalence of chronic noncommunicable diseases. Simultaneously, the consequences of the COVID-19 pandemic that remain unpredictable over time have also made meeting the growing need to resolve the neurological disorder issue (e.g., MS, PD, and AD) more urgent [9–11]. Still, there is a general lack of available data concerning current spatial distributions and time tendencies of the burden of these issues among older adults. This knowledge gap is essential because it has direct implications on formulating policies on the development of sound policies in terms of public health.

In response, the proposed study intends to perform an initial evaluation of the latest burden patterns of the most relevant subgroups in terms of age, sex, geographical location, and Socio-demographic Index (SDI) level. One of the aims of this analytical framework is to simulate the estimated burden of these diseases to the year 2050 majorly on the dynamic of these diseases in relation to age. To accomplish this, the study will be able to obtain a rather comprehensive analysis of international epidemiological trends among adults aged in 55 and above years with illogical persistence of LOMS, PD, and ADOD.

## Methods

### Data sources

Data were drawn from the GBD 2021 dataset in accordance with the standard data collection protocol of the GBD study. This dataset offers comprehensive information from 1990 to 2021 across what are reported as 204 countries and territories, covering metrics like incident cases, prevalent cases, deaths, and DALYs for conditions including LOMS, PD, ADOD, along with their corresponding age–standardized rates (ASRs) stratified by sex, age, and region and territories [12].

### Disease burden metrics

The GBD 2021 framework was utilized to systematically quantify the global burden of LOMS, PD, ADOD. This entailed integrating multiple epidemiological metrics and uncertainty estimates (95% uncertainty interval [UI]), derived from the 2.5th and 97.5th percentiles of 1000 posterior distribution draws. Temporal trends in age–standardized rates (ASRs) for these neurological disorders were assessed at global, regional, and national levels. This method identified significant trend change points, partitioned overall trends into subsegments, and evaluated epidemiological trends for each subsegment by calculating the annual percentage change (APC) and estimated average APC (EAPC). For age–stratified analyses, individuals were characterized as standard 5–year intervals: 55–59, 60–64, 65–69, 70–74, 75–79, 80–84, 85–89, 90–94, and ≥ 95 years. Further stratification by female and male genders was conducted within these age groups.

### Socio–demographic index

Moreover, the study utilized the SDI, a composite indicator that assesses the sociodemographic status of countries or regions by relying on average income, educational attainment, and fertility rates. SDI values range from 0 to 1, with higher scores reflecting more advanced sociodemographic development. In this analysis, SDI was employed to characterize disparities in neurodegenerative disease burden across socioeconomic contexts, providing a framework for cross–national health inequality analysis. The SDI was classified into five groups: High SDI, High–middle SDI, Middle SDI, Low–middle SDI, and Low SDI.

### Decomposition analysis

To analyze the key factors of neurodegenerative disease burden changes from 1990 to 2021, we conducted a decomposition analysis quantifying three population–level factors: population growth, aging demographics, and epidemiologic shifts (age–standardized disease rates). This approach isolates each factor’s contribution while holding others constant, enabling precise attribution of changes in burden across global, regional, and national scales. Specifically for LOMS, PD, ADOD, the analysis differentiated between demographic influences (population size/age structure) versus true changes in disease risk patterns.

### Projection methodology

To forecast future disease burden trends, we employed complementary BAPC and ARIMA models to project future disease burden trends. The BAPC model was applied for short-term projections (2022–2036), while ARIMA was used for extended forecasting to 2050.

### BAPC model

The BAPC model is particularly well-suited for handling intricate, high-dimensional, and sparse datasets, which are commonly found in extensive epidemiological research such as the GBD study 2021 [13]. It extends the generalized linear model within a Bayesian framework to dynamically model smooth, continuous age, period, and cohort effects using a second-order random walk, improving posterior estimation accuracy [14]. The BAPC model is combined with the Integrated Nested Laplace Approximation (INLA) to approximate marginal posteriors, addressing computational issues like poor mixing and slow convergence common in MCMC-based Bayesian methods [15].

## ARIMA model

To evaluate the stability of the BAPC forecast results, a sensitivity analysis was conducted by applying an ARIMA model [16]. The ARIMA model is widely utilized in econometric analysis for examining both stationary and nonstationary time series, as well as assessing the effects of policies and interventions on particular outcomes across temporal intervals [17]. Within the ARIMA (p, d, q) framework, “p” indicates the number of autoregressive components, “d” refers to the degree of differencing applied, and “q” represents the number of moving average terms. The computational procedures align with established methodologies documented in prior research [18].

### Statistical analysis

All statistical analyses were performed in R Studio (version 4.4.2). The statistical significance level for all analyses was set at *p* < 0.05. To calculate the EAPC, a linear regression analysis was applied to the natural logarithm of annual rates. A sequential numerical label was assigned to each year, starting with t = 0 for 1990 (set as the baseline year), followed by t = 1, 2, …, 31 for subsequent years. The regression equation used in this process is expressed as Ln(Rt) = α + βt + ε. Here, Rt stands for the rate observed in year t, α represents the intercept of the regression line, β denotes the regression coefficient, and ε is the error term accounting for unexplained variation. The EAPC value was then computed using the formula: EAPC = (e^β^−1)×100%. Additionally, the 95% confidence intervals for the EAPC were determined based on the standard error of the regression coefficient β.

## Result

### Global burden and trends (1990–2021)

In 2021, LOMS, PD, and ADOD remained major global health concerns among adults aged 55 years and older. The estimated global number of new cases in 2021 was 5,035.5 for LOMS, 1,184,055.3 for PD, and 9,405,626.3 for ADOD (Tables S1, S2 and S3). Among these, ADOD had the highest age-standardized incidence rate (ASIR), at 679.44 per 100,000 population (95% uncertainty interval [UI]: 466.33–922.85). The ASIR of PD showed an upward trend, increasing from 60.57 in 1990 to 82.17 in 2021, whereas the ASIR of LOMS declined slightly from 0.39 to 0.34 over the same period (Tables S1, S2 and S3).

In 2021, the global number of prevalent cases was 761,107.6 for LOMS, 10,757,500.8 for PD, and 20,836,524.4 for ADOD, with corresponding age-standardized prevalence rates (ASPR) of 51.24, 751.69, and 3,975.78 per 100,000 population, respectively (Tables S1, S2 and S3). The three disorders exhibited distinct age-specific peaks in incidence and prevalence: LOMS peaked in the 55–59 age group, PD in the 70–74 age group, and ADOD in the 80–84 age group (Figs. S1, S2 and S3, Tables 1, 2 and 3).

**Table 1:** Global burden of late-onset multiple sclerosis by age group in 2021 and estimated annual percentage change‌ (EAPC) from 1990 to 2021

Age group	Incidence			Prevalence			Deaths			DALYs		
	No.(95% UI)	Age–specific rate per 100 000 (95% UI)	EAPC (95% CI)	No.(95% UI)	Age–specific rate per 100 000 (95% UI)	EAPC (95% CI)	No.(95% UI)	Age–specific rate per 100 000 (95% UI)	EAPC (95% CI)	No.(95% UI)	Age–specific rate per 100 000 (95% UI)	EAPC (95% CI)
55–59 years	1670.53 (1073.90 to 2380.87)	0.42 (0.27 to 0.69)	–0.63 (–0.70 to − 0.56)	201253.03 (180204.93 to 222997)	50.86 (45.54 to 56.35)	0.33 (0.22 to 0.44)	1923.04 (1838.02 to 2010.42)	0.49 (0.46 to 0.51)	–0.36 (–0.62 to − 0.1)	115366.14 (100855.57 to 131543.24)	29.15 (25.49 to 33.24)	–0.08 (–0.28 to 0.12)
60–64 years	926.37 (588.47 to 1271.01)	0.29 (0.18 to 0.49)	–0.43 (–0.46 to − 0.40)	172994.29 (155457.48 to 192456.37)	54.05 (48.57 to 60.13)	0.36 (0.26 to 0.47)	2169.41 (2064.61 to 2257.67)	0.68 (0.65 to 0.71)	0.2 (0.04 to 0.36)	105913.98 (93098.61 to 119417.93)	33.09 (29.09 to 37.31)	0.26 (0.13 to 0.4)
65–69 years	844.06 (610.30 to 1050.67)	0.31 (0.22 to 0.38)	–0.32 (–0.36 to − 0.29)	137408.76 (123191.58 to 152732.53)	49.81 (44.66 to 55.37)	0.18 (0.05 to 0.31)	2192.33 (2052.72 to 2300.7)	0.79 (0.74 to 0.83)	0.3 (0.14 to 0.47)	87139.63 (76961.67 to 97531.93)	31.59 (27.90 to 35.36)	0.25 (0.1 to 0.4)
70–74 years	650.42 (441.23 to 843.04)	0.32 (0.21 to 0.41)	–0.27 (–0.32 to − 0.22)	104844.92 (93613.72 to 116602.35)	50.94 (45.48 to 56.65)	0.1 (0.02 to 0.17)	2000.74 (1838.82 to 2112.32)	0.97 (0.89 to 1.03)	0.49 (0.31 to 0.66)	65299.91 (57900.58 to 73820.24)	31.72 (28.13 to 35.86)	0.32 (0.19 to 0.45)
75–79 years	431.59 (326.00 to 528.81)	0.33 (0.25 to 0.40)	–0.21 (–0.26 to − 0.17)	65011.27 (57593.14 to 73196.21)	49.29 (43.67 to 55.5)	–0.09 (–0.15 to − 0.02)	1425.12 (1280.81 to 1527.56)	1.08 (0.97 to 1.16)	0.43 (0.28 to 0.59)	38135.27 (33363.6 to 43346.1)	28.92 (25.30 to 32.87)	0.2 (0.09 to 0.31)
80–84 years	285.27 (227.39 to 341.68)	0.33 (0.26 to 0.39)	–0.18 (–0.21 to − 0.15)	43393.79 (38196.07 to 49446.14)	49.55 (43.61 to 56.46)	–0.17 (–0.29 to − 0.06)	978.27 (806.16 to 1068.06)	1.12 (0.92 to 1.22)	0.46 (0.34 to 0.58)	22328.93 (18976.48 to 25688.73)	25.49 (21.67 to 29.33)	0.15 (0.05 to 0.25)
85–89 years	151.06 (120.82 to 180.95)	0.33 (0.26 to 0.40)	–0.15 (–0.2 to − 0.11)	23346.66 (20330.97 to 26877.51)	51.06 (44.47 to 58.78)	–0.08 (–0.23 to 0.07)	552.42 (444.03 to 612.36)	1.21 (0.97 to 1.34)	0.48 (0.36 to 0.6)	10788.12 (9084.29 to 12564.54)	23.60 (19.87 to 27.48)	0.18 (0.07 to 0.29)
90–94 years	58.42 (43.66 to 71.40)	0.33 (0.24 to 0.40)	–0.15 (–0.18 to − 0.11)	9796.55 (8449.71 to 11384.13)	54.76 (47.23 to 63.64)	–0.09 (–0.23 to 0.05)	229.28 (176.49 to 257.35)	1.28 (0.99 to 1.44)	0.67 (0.55 to 0.78)	4154.11 (3424.32 to 4921)	23.22 (19.14 to 27.51)	0.25 (0.14 to 0.36)
95 + years	17.85 (11.89 to 23.26)	0.33 (0.22 to 0.43)	–0.10 (–0.14 to − 0.07)	3058.31 (2614.76 to 3589.24)	56.11 (47.97 to 65.85)	–0.37 (–0.53 to − 0.2)	44.86 (32.51 to 51.6)	0.82 (0.6 to 0.95)	1.09 (0.95 to 1.23)	1028.98 (808.58 to 1254.66)	18.88 (14.84 to 23.02)	0.1 (–0.02 to 0.21)

**Table 2:** Global burden of Parkinson’s disease by age group in 2021 and estimated annual percentage change‌ (EAPC) from 1990 to 2021

Age group	Incidence			Prevalence			Deaths			DALYs		
	No.(95% UI)	Age–specific rate per 100 000 (95% UI)	EAPC (95% CI)	No.(95% UI)	Age–specific rate per 100 000 (95% UI)	EAPC (95% CI)	No.(95% UI)	Age–specific rate per 100 000 (95% UI)	EAPC (95% CI)	No.(95% UI)	Age–specific rate per 100 000 (95% UI)	EAPC (95% CI)
55–59 years	92049.06 (56448.67 to 140184.65)	23.26 (14.26 to 35.42)	1.7 (1.62 to 1.78)	836714.14 (651211.93 to 1066128.56)	211.44 (164.56 to 269.41)	1.83 (1.68 to 1.98)	5091.85 (4607.01 to 5637.4)	1.29 (1.16 to 1.42)	–0.60 (–0.65 to –0.54)	296688.41 (246734.29 to 350658.26)	74.97 (62.35 to 88.61)	0.23 (0.17 to 0.28)
60–64 years	128003.49 (98271.6 to 163068.03)	40 (30.71 to 50.95)	1.31 (1.24 to 1.38)	1060264.09 (825630.52 to 1366791.11)	331.28 (257.97 to 427.06)	1.77 (1.67 to 1.88)	9602.65 (8808.47 to 10522.83)	3 (2.75 to 3.29)	–0.44 (–0.48 to − 0.4)	433994.99 (374423.59 to 501238.46)	135.6 (116.99 to 156.61)	0.22 (0.18 to 0.27)
65–69 years	196500.84 (134364.69 to 263738.78)	71.24 (48.71 to 95.61)	1.05 (0.97 to 1.12)	1676422.85 (1383237.23 to 2060676.76)	607.75 (501.46 to 747.05)	1.6 (1.57 to 1.63)	22522.85 (20687.81 to 24582.41)	8.17 (7.5 to 8.91)	–0.30 (–0.39 to − 0.22)	790574.05 (696626.3 to 884188.07)	286.6 (252.55 to 320.54)	0.17 (0.12 to 0.23)
70–74 years	236234.42 (187603.1 to 284527.56)	114.77 (91.14 to 138.23)	0.94 (0.9 to 0.97)	1999449.48 (1630854.56 to 2417047.66)	971.36 (792.3 to 1174.24)	1.42 (1.39 to 1.44)	49702.12 (45334.22 to 54313.99)	24.15 (22.02 to 26.39)	–0.12 (–0.20 to − 0.04)	1277331.81 (1148465.22 to 1410501.77)	620.55 (557.94 to 685.24)	0.16 (0.1 to 0.22)
75–79 years	228210.33 (167075.32 to 287353.02)	173.04 (126.68 to 217.88)	0.93 (0.91 to 0.95)	1950769.68 (1630158.86 to 2305021.04)	1479.15 (1236.05 to 1747.76)	1.38 (1.35 to 1.41)	78255.75 (71646.77 to 85528.66)	59.34 (54.33 to 64.85)	0.05 (–0.01 to 0.1)	1519123.65 (1395182.62 to 1653083.59)	1151.86 (1057.88 to 1253.43)	0.23 (0.19 to 0.28)
80–84 years	174717.1 (137677.26 to 210363.25)	199.49 (157.2 to 240.19)	0.93 (0.91 to 0.96)	1732586.51 (1427648.92 to 2076015.54)	1978.22 (1630.05 to 2370.34)	1.42 (1.38 to 1.47)	98150.87 (86539.09 to 106194.57)	112.07 (98.81 to 121.25)	0.26 (0.2 to 0.32)	1457938.97 (1298400.51 to 1582686.17)	1664.64 (1482.48 to 1807.07)	0.41 (0.36 to 0.46)
85–89 years	87633.7 (62338.77 to 119403.33)	191.67 (136.34 to 261.15)	0.92 (0.88 to 0.96)	1017289.14 (848111.74 to 1200132.14)	2224.95 (1854.94 to 2624.86)	1.56 (1.53 to 1.59)	76985.97 (65651.14 to 84397.48)	168.38 (143.59 to 184.59)	0.47 (0.38 to 0.56)	895592.58 (775077.45 to 989372.42)	1958.79 (1695.2 to 2163.9)	0.59 (0.52 to 0.66)
90–94 years	31458.42 (21188.4 to 43423.17)	175.85 (118.44 to 242.73)	0.75 (0.67 to 0.82)	380063.43 (311897.5 to 467026.34)	2124.52 (1743.48 to 2610.64)	1.66 (1.63 to 1.69)	33840.65 (27616.75 to 37549.06)	189.17 (154.38 to 209.9)	0.56 (0.50 to 0.62)	340376.29 (284523.07 to 375598.04)	1902.67 (1590.46 to 2099.56)	0.69 (0.63 to 0.74)
95 + years	9247.91 (4736.88 to 16113.1)	169.68 (86.91 to 295.64)	0.55 (0.48 to 0.63)	103941.42 (80213.92 to 134143.92)	1907.07 (1471.73 to 2461.22)	1.54 (1.48 to 1.6)	8793.26 (6561.15 to 10018.17)	161.34 (120.38 to 183.81)	0.54 (0.46 to 0.61)	83992.95 (66432.36 to 95372.85)	1541.07 (1218.87 to 1749.86)	0.63 (0.55 to 0.71)

**Table 3:** Global burden of AD and other dementias by age group in 2021 and estimated annual percentage change‌ (EAPC) from 1990 to 2021

Age group	Incidence			Prevalence			Deaths			DALYs		
	No.(95% UI)	Age–specific rate per 100 000 (95% UI)	EAPC (95% CI)	No.(95% UI)	Age–specific rate per 100 000 (95% UI)	EAPC (95% CI)	No.(95% UI)	Age–specific rate per 100 000 (95% UI)	EAPC (95% CI)	No.(95% UI)	Age–specific rate per 100 000 (95% UI)	EAPC (95% CI)
55–59 years	397940.56 (263070.16 to 565028.94)	100.56 (66.48 to 142.78)	0.2 (0.16 to 0.24)	2345032.54 (1828789.78 to 2960594.02)	592.59 (462.13 to 748.14)	0.08 (0.06 to 0.09)	20752.52 (3809.07 to 65580.07)	5.24 (0.96 to 16.57)	0.07 (0.05 to 0.09)	1138561.94 (515355.64 to 2753866.6)	287.71 (130.23 to 695.9)	0.08 (0.06 to 0.1)
60–64 years	552536.18 (368111.17 to 761429.64)	172.64 (115.02 to 237.91)	0.22 (0.19 to 0.26)	3461247.53 (2683722.22 to 4380526.69)	1081.48 (838.54 to 1368.71)	0.15 (0.13 to 0.17)	43680.24 (8875.95 to 136093.58)	13.65 (2.77 to 42.52)	0.09 (0.07 to 0.1)	1928367.38 (851488.95 to 4570023.09)	602.52 (266.05 to 1427.92)	0.11 (0.1 to 0.13)
65–69 years	894212.34 (596765.71 to 1278304.32)	324.18 (216.34 to 463.42)	0.14 (0.1 to 0.17)	5383792.63 (4185913.08 to 6722380.19)	1951.77 (1517.5 to 2437.04)	0.14 (0.11 to 0.17)	84975.81 (19430.93 to 252737.56)	30.81 (7.04 to 91.62)	0.06 (0.04 to 0.08)	3127092.07 (1409726.53 to 7085605.77)	1133.65 (511.06 to 2568.72)	0.09 (0.07 to 0.11)
70–74 years	1327895.66 (917821.66 to 1824757.72)	645.11 (445.89 to 886.5)	0.08 (0.04 to 0.11)	7364245.23 (5701395.92 to 9397560.88)	3577.67 (2769.83 to 4565.49)	0.08 (0.05 to 0.11)	132160.71 (33237.8 to 374403.84)	64.21 (16.15 to 181.89)	0.02 (–0.01 to 0.04)	4126748.89 (2067522.79 to 8978837.19)	2004.84 (1004.44 to 4362.06)	0.03 (0 to 0.06)
75–79 years	1745039.42 (1180496.18 to 2360958.83)	1323.16 (895.1 to 1790.17)	0 (–0.04 to 0.03)	9325099.49 (7423397.81 to 11476764.31)	7070.66 (5628.72 to 8702.14)	0.04 (0.01 to 0.07)	192822.12 (46221.72 to 557928.05)	146.21 (35.05 to 423.04)	–0.04 (–0.06 to–0.02)	4865372.52 (2392862.16 to 10651564.79)	3689.12 (1814.36 to 8076.44)	–0.02 (–0.05 to 0)
80–84 years	2007933.16 (1382098.85 to 2638986.3)	2292.6 (1578.04 to 3013.12)	–0.09 (–0.13 to − 0.06)	11387765.6 (9080701.96 to 14175059.43)	13002.25 (10368.1 to 16184.71)	–0.01 (–0.04 to 0.02)	399209.63 (102318.57 to 1058968.73)	455.81 (116.82 to 1209.1)	–0.04 (–0.06 to–0.02)	7290270.13 (3360051.75 to 15997697.88)	8323.84 (3836.42 to 18265.75)	–0.05 (–0.07 to–0.02)
85–89 years	1465774.5 (1034355.14 to 1947882.08)	3205.86 (2262.28 to 4260.29)	–0.14 (–0.16 to–0.11)	9159172.08 (7253558.28 to 11346546.18)	20032.40 (15864.55 to 24816.5)	–0.06 (–0.09 to − 0.03)	476952.12 (119693.95 to 1251451.26)	1043.16 (261.79 to 2737.1)	–0.03 (–0.05 to–0.01)	6681788.77 (3013871.49 to 14385687.22)	14614.01 (6591.76 to 31463.53)	–0.06 (–0.07 to–0.04)
90–94 years	740951.67 (531376.73 to 1018131.28)	4141.85 (2970.35 to 5691.26)	–0.13 (–0.14 to–0.12)	4724243.74 (3711195.04 to 5911668.66)	26408.1 (20745.25 to 33045.7)	–0.11 (–0.13 to − 0.09)	380306.78 (96763.86 to 941673.1)	2125.88 (540.9 to 5263.87)	–0.01 (–0.03 to 0.01)	4343993.29 (1829089.57 to 9371596.64)	24282.53 (10224.45 to 52386.39)	–0.04 (–0.05 to–0.03)
95 + years	273342.78 (175149.12 to 392539.75)	5015.18 (3213.56 to 7202.16)	–0.14 (–0.15 to–0.14)	1754687.21 (1360064.53 to 2222942.38)	32194.27 (24953.9 to 40785.62)	–0.18 (–0.2 to − 0.16)	212863.34 (53805.31 to 531059.83)	3905.53 (987.2 to 9743.67)	–0.04 (–0.07 to 0)	2123304.29 (834243.32 to 4683582.64)	38957.51 (15306.35 to 85932.43)	–0.11 (–0.14 to–0.07)

Notable discrepancies between the age peaks of ASIR and ASPR were observed for LOMS and PD. The ASIR of LOMS peaked in the 55–59 age group, while its ASPR peaked in the 60–64 age group. For PD, the ASIR peaked in the 80–84 age group, whereas the ASPR peaked in the 85–89 age group (Figs. S1, S2, Tables 1 and 2). For ADOD, both ASIR and ASPR increased progressively with age, reaching their highest levels in the 95 + age group (Fig. S3, Table 3).

ADOD accounted for the largest DALY burden, with a total of 35,625,499.3 DALYs and an age-standardized DALY rate (ASDALYR) of 2,621.1 per 100,000 population (95% UI: 1,192.8–5,766.2) (Table S3). The ASDALYR for ADOD rose sharply with age, from 287.71 per 100,000 in the 55–59 age group to 38,957.51 per 100,000 in the 95 + age group in 2021 (Table 3). Additionally, the global number of deaths from ADOD nearly tripled from 0.66 million in 1990 to 1.94 million in 2021, with mortality rates increasing substantially over this 32-year period (Table S3).

### Sex differences in disease burden

In 2021, there existed a stable pattern of sex-disparity for all the three illnesses. Women had a greater burden of LOMS and ADOD on all measures but men had a greater burden of PD. In the case of LOMS, incidence, prevalence, deaths, and DALYs female-to-male ratios were 1.03, 2.18, 1.78 and 1.86, respectively (Fig. S10, Table S1). In the case of ADOD, the ratios were 1.73, 1.77, 2.12 and 1.92 (Fig. S11, Table S3). PD, on the contrary, had ratios lower than 1 on all four indicators (0.76, 0.84, 0.77 and 0.76, respectively, Fig. S12, Table S2). Back examination of the data in 1990–2021 showed that there were sex-specific trends: LOMS decreased both in males and females, ASIR in both sexes, ASDR and ASDALYR in women, and did not change in men (Fig. S10, Table S1). For PD, males experienced larger increases in all key age-standardized rates than females (Fig. S11, Table S2). For ADOD, sex disparities in ASIR, ASPR, ASDR, and ASDALYR remained substantial and were either slightly widened or stable over time (Fig. S12; Table S3).

### SDI‑stratified burden patterns

In 2021, the burden of the three neurological disorders varied markedly by SDI. High SDI regions carried the highest age-standardized rates for LOMS across all key metrics: incidence (0.37 per 100,000; 95% UI: 0.24–0.52), prevalence (135.88 per 100,000; 95% UI: 123.55–149.44), mortality (2.41 per 100,000; 95% UI: 2.20–2.57), and DALY rates (89.56 per 100,000; 95% UI: 79.55–99.66) (Fig. S4, Table S4). For PD, high-middle SDI regions exhibited the highest age-standardized incidence (95.44 per 100,000; 95% UI: 66.64–127.52), prevalence (940.38 per 100,000; 95% UI: 752.75–1,164.19), and DALY rates (529.89 per 100,000; 95% UI: 462.21–598.89), while low SDI regions recorded the highest age-standardized death rate (ARDR, 28.75 per 100,000; 95% UI: 22.87–34.81) (Fig. S5, Table S4). ADOD showed the most elevated age-standardized rates across incidence (751.70 per 100,000; 95% UI: 513.68–1,025.46), prevalence (4,393.63 per 100,000; 95% UI: 3,439–5,514.2), mortality (155.71 per 100,000; 95% UI: 38.15–420.95), and DALYs (2,800.58 per 100,000; 95% UI: 1,286.23–6,161.8) in high-middle SDI regions (Fig. S6, Table S4).

### Regional burden distribution

Regionally, the burden was geographically concentrated. For LOMS, Central Asia had the highest ASIR(0.71 per 100,000; 95% UI: 0.53–0.9), Western Europe the highest prevalence (180.06 per 100,000; 95% UI: 159.01–202.91), and high-income North America the highest mortality (3.57 per 100,000; 95% UI: 3.23–3.84) and DALY rates (128.75 per 100,000; 95% UI: 113.54–142.8) (Fig. S7, Table S1). In PD, East Asia recorded the highest age-standardized incidence (122.35 per 100,000; 95% UI: 78.63–173.08), prevalence (1,320.76 per 100,000; 95% UI: 1,026.74–1,682.72), and DALY rates (600.80 per 100,000; 95% UI: 498.93–709.99), whereas high-income North America showed the highest mortality rate (33.50 per 100,000; 95% UI: 28.77–35.91) (Fig. S8, Table S2). For ADOD, East Asia led in age-standardized incidence (850.81 per 100,000; 95% UI: 584.72–1,162.65) and prevalence (5,106.19 per 100,000; 95% UI: 3,979.84–6,412.27), while Central Sub-Saharan Africa bore the highest mortality (205.65 per 100,000; 95% UI: 48.15–566.13) and DALY rates (3,443.11 per 100,000; 95% UI: 1,423.61–8,141.58) (Fig. S9, Table S3).

### National-level burden and temporal trends

**Fig. 1:**
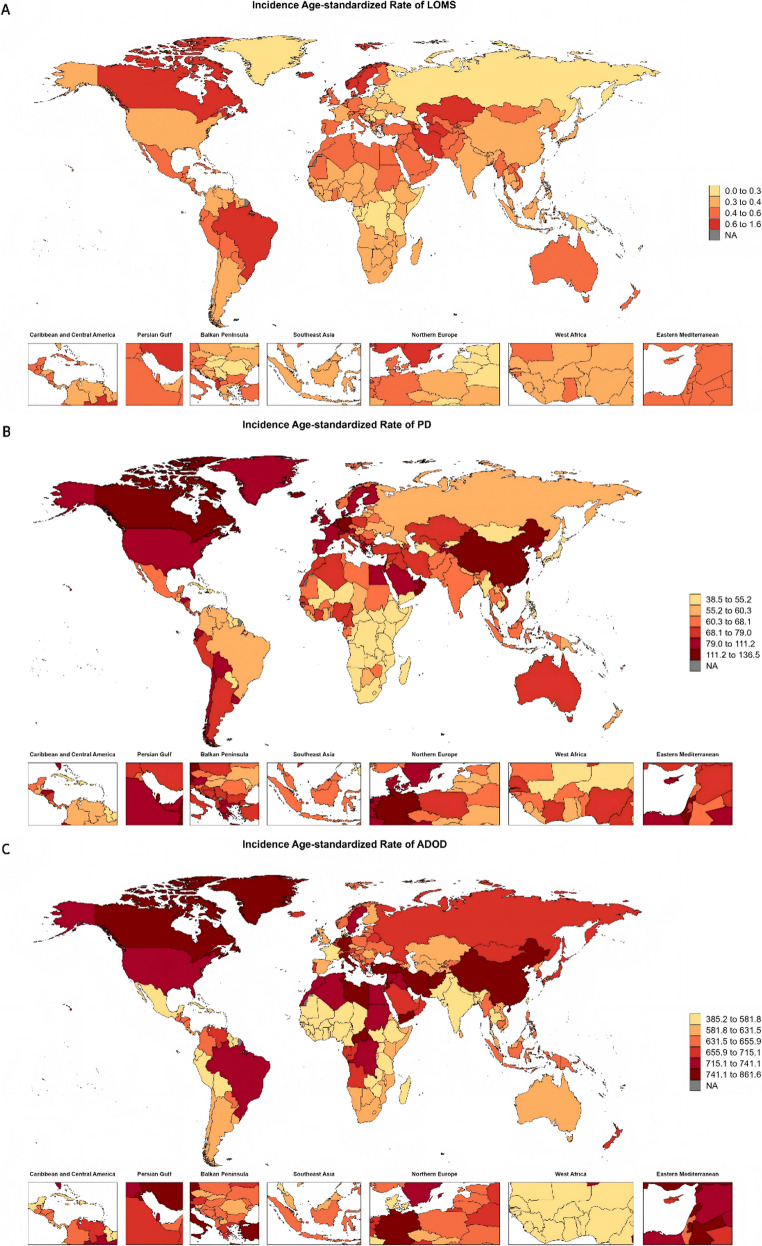
Global maps of age-standardized incidence rate. (**A**), Attributable to late-onset multiple sclerosis (LOMS); (**B**), Attributable to Parkinson’s disease (PD); (**C**), Attributable to Alzheimer’s disease and other dementias (ADOD)

At the national level, Kazakhstan reported the highest ASIR of LOMS (1.5 per 100,000; 95% UI: 1.18–1.86), nearly four times the global average, while Sweden had the highest prevalence (358.91 per 100,000; 95% UI: 313.08–406.64), and the United Kingdom the highest mortality (5.39 per 100,000; 95% UI: 4.99–5.72) and DALY rates (186.63 per 100,000; 95% UI: 166.80–206.24) (Fig. 1, Table S7). For PD, Qatar led in incidence (136.39 per 100,000; 95% UI: 102.07–178.11), China in prevalence (1,332.36 per 100,000; 95% UI: 1,028.75–1,706.51), and Honduras in both mortality (56.47 per 100,000; 95% UI: 43.24–70.10) and DALY rates (894.72 per 100,000; 95% UI: 703.82–1,093.48) (Fig. 1, Table S8). Regarding ADOD, China exhibited the highest incidence (861.46 per 100,000; 95% UI: 591.65–1,178.24), Gabon the highest mortality (208.91 per 100,000; 95% UI: 48.87–565.48), and the Democratic Republic of the Congo the highest DALY rate (3,494.96 per 100,000; 95% UI: 1,435.84–8,199.47) (Fig. 1, Table S9). Temporal analyses (1990–2021) revealed that mainland China experienced the steepest rise in ASIR for ADOD, with an EAPC of 0.42 (95% CI: 0.34–0.50), followed by Taiwan (Province of China) (EAPC 0.39; 95% CI: 0.29–0.48). Similarly, Taiwan (Province of China) recorded the most rapid increase in ASPR (EAPC 0.47; 95% CI: 0.35–0.58), slightly ahead of mainland China (EAPC 0.45; 95% CI: 0.36–0.54).

### Decomposition analysis of incidence and DALY changes

Population growth was identified as the predominant driver of increases in both incident cases and DALYs for all three conditions between 1990 and 2021 (Tables S10, S11). Its impact was most pronounced for incident LOMS cases in the high-middle SDI quintile (contributing to a 170.61% increase) and for incident PD and ADOD cases in the low SDI quintile (contributing 81.24% and 96.00% increases, respectively). Similarly, for DALY changes, population growth contributed most substantially to LOMS burden in the high-middle SDI quintile (144.55% increase) and to PD burden in the low SDI quintile (88.74% increase).

### Projected burden to 2036 and 2050

Using complementary modeling approaches (BAPC for 2022–2036 and ARIMA for 2022–2050), we projected diverging trends in age-standardized rates but consistent increases in absolute burden across all three conditions.

For LOMS, BAPC projections (2022–2036) demonstrated consistent global declines in age-standardized rates: ASIR decreased by 2.2% in females and 7.1% in males; ASPR by 18.9% and 15.8%; ASDR by 33.2% and 27.5%; and total DALYs by 31.1% and 30.3%, respectively (Table S12). Conversely, ARIMA projections (2022–2050) indicated increasing absolute numbers of incident cases, prevalent cases, deaths, and DALYs in both sexes. Age-standardized metrics through 2050 showed ASIR declines of 6.25% (females) and 11.4% (males) and ASPR declines of 3.83% and 5.43%, whereas ASDR diverged substantially—decreasing 41.3% in females but increasing 3.23% in males—with ASDALYR decreasing 35.2% in females yet increasing 3.44% in males (Fig. S13; Tables S13–S14).

For PD, BAPC projections (2022–2036) revealed marked sex disparities. Among females, ASIR increased 21.5% and ASPR 22.4%, whereas ASDR decreased 13.2% and ASDALYR 4.12%. Among males, ASIR rose 23.6% and ASPR 22.3%, while ASDR declined 10.2% and ASDALYR 1.75% (Table S12). ARIMA projections (2022–2050) showed rising absolute counts across all metrics for both sexes. Long-term age-standardized trends diverged by sex: females exhibited increases in ASIR (18.5%), ASPR (30.9%), ASDR (1.57%), and ASDALYR (4.4%), whereas males showed increases in ASIR (21.2%) and ASPR (37.8%) but decreases in ASDR (14.5%) and ASDALYR (3.2%) (Fig. S14; Tables S13–S14).

For ADOD, BAPC projections (2022–2036) indicated universal increases in age-standardized metrics: ASIR rose 18.4% in females and 17.9% in males; ASPR 17.5% and 16.9%; ASDR 6.7% and 6.2%; and ASDALYR 10.7% and 10.5% (Table S12). ARIMA projections (2022–2050) demonstrated substantial increases in absolute numbers for both sexes, with females bearing a disproportionately larger burden of numerical growth. Notably, age-standardized metrics through 2050 showed divergent patterns: ASIR declined 3.2% in females and 2.7% in males; ASPR decreased 3.2% in females but increased 10.5% in males; ASDR declined marginally in females (0.05%) but rose 7.1% in males; and ASDALYR decreased 1.1% in females while increasing 10.6% in males (Fig. S15; Tables S13–S14).

## Discussion

Alzheimer’s disease and other dementias, Parkinson’s disease, and multiple sclerosis have become increasingly significant challenges to public health on a global scale [19–21]. The study offers a systematic evaluation of the burden that can be linked to these three conditions in the population of 55 years and above across the globe in the period between 1990 and 2021. There was an increase in the DALYs of ADOD by 169 per cent, PD by 165 per cent and LOMS by 117 per cent. The decomposition analysis established that the primary source of this growth was the demographic elements. Conversely, the age-adjusted DALY rates varied on a smaller scale: ADOD (0.33 PD, 0.16 LOMS) EAPCs were negative, which means that the disease risk at the population level has not increased in general and drastically. This interpretation is supported by the future projection models further. In the case of LOMS, incidence, prevalence, and DALY rates will decrease during the years 2022–2036, which is probably due to the progress in the diagnosis area and the broader use of disease-modifying therapies. Between 1990 and 2021, the absolute burden of neurological disorders in the global population aged 55 years and older increased dramatically, largely attributable to population growth and aging [22–24]. The ADOD burden among them is expected to grow to a more significant extent, which is one of the challenges that will present challenges to global public health systems in the future. It is important to note that our projections do not incorporate the potential effects of future disease-modifying therapies, such as monoclonal antibodies for AD, which may further prolong survival. Should such therapies prove to be effective and become widely available in the coming years, they could lead to higher-than-estimated prevalence rates than those projected in our current models. This represents an important consideration for healthcare planning and underscores the need for continued surveillance and model updating as new therapeutic evidence emerges.

Geographic disparities: The Northern European countries (e.g., Sweden and Norway) have the highest age-standardized prevalence rates (ASPRs), which correlates with the reported data of the highest prevalence in Europe (142.81 per 100, 000 population) and underscores increased susceptibility to multiple sclerosis (MS) in populations of Northern European countries [25]. At the global level, China ranks high across multiple absolute burden metrics for PD and ADOD, including incidence, prevalence, deaths, and DALYs. This is primarily attributable to its large and rapidly aging population [26, 27]. The demographic shift in China and the consequential strain on its health sector represents the necessary poster child on the demand on the health sector of other countries experiencing the same population aging patterns as well as the urgency of the need to address the issue of neurological disorders given population aging.

Inequalities in SDI: LOMS and ADOD are most age-standardized in high-SDI areas around the world. The given phenomenon could not be merely the coincidence and it is closely connected with the prolonged life expectancy, well-developed diagnostic systems, and increased exposure to the modifiable risk factors (unhealthy diet, physical inactivity, and obesity) within these populations [28]. Higher case identification stems from the accuracy of diagnostic capacity, whereas the increased life expectancy increases the disease stage, which together increases the age-standardized burden [29]. Conversely, low-SDI regions exhibit a much higher death rate resulting of PD in relation to other regions when it comes to age standardized death rate. Such a pointer is a strong indication that these settings have serious shortcomings when it comes to the long-term diagnosis, standardized treatment and overall comprehensive management of PD [30]. Delays in detection of patients probably cause them to miss the best intervention windows, which eventually translates to accelerated disease progressions and increased deaths. It should be mentioned that the reported rate of incidence and prevalence is relatively lower in low-SDI regions, in contrast to actual lower population risk, which is probable to result from under-ascertainment (related to delayed healthcare access, lack of quality service provision, and the quality of medical infrastructure) rather than a genuine lessening of the population risk [31].

The three neurological disorders have age specific age-at-onset distributions whereby LOMS is most frequently 55–64 years, PD falls in the 70–84 years category and ADOD occupies those that are aged 80 and above. Such obvious age stratification causes a sharp need to match the patterns of global health resource allocation and service delivery with the age composition of a particular disease. The stratification of policies and interventions should follow the situation to prevent the mismanagement of resources such as the increase of screening at the age of 55–64 when the incidence of LOMS is the highest and the provision of adequate long-term care services to the elderly populations that are the most affected by PD and ADOD. At the same time, the three disorders have a significant sex variation: even the incidence of LOMS and ADOD in females is greater, and PD is more male. Even though PD occurs more frequently in males, females are misdiagnosed or diagnosed later and variability in symptom expression and response to treatment makes it difficult to manage the disease [32]. These differences are based on a combination both of biological and socioenvironmental factors. Biologically, hormonal differences, genetic predisposition, and pregnancy could predispose the two sexes to the risk of diseases [33–35]. On the socioenvironmental level, women tend to live longer than men, that is, with increasing age in the population, the proportion of women as the elderly increases. Moreover, women tend to earn less and be less educated and take up primary informal care systems. The related elevated caregiving cost might further augment psychological risk variables (e.g. sleep disorders and depression) in females; which might increase the risk and severity of ADOD [36]. These results suggest that prevention and management of neurological disorders cannot take a homogenous approach but should be proactive in incorporating a sex-specific approach, and one needs to implement interventions aimed at shaping the risk profile of the different sexes.

About the future policy and research, the health system planning should focus on the response to the escalating prevalence of the neurological disorders as the population is aging. These involve enhancing specialty capacity of neurology and advancement of systems of tiered diagnosis and treatment and long-term care. The implementation needs to be stratified and precision: high-SDI areas need to emphasize the processes of maximizing diagnosis/treatment and risk factor mitigation; middle- and low-SDI should emphasize the processes of maximizing basic diagnosis and treatment services and increasing the level of disease surveillance; low-SDI areas need some international backing in order to achieve better diagnostic and medicinal access. At the same time, age and sex disparities also need to be targeted, i.e., early identification should be encouraged among older individuals aged 55–64 who are the targeted group of LOMS, comorbidity management among females with PD, and enhanced protection among older women as well as atypical symptom screen among males in the context of ADOD prevention and control. The biological and social determinants of such disparities in burden should be further investigated in future research. Moreover, to present a more distinctive evidence base on policymaking, the long-term efficacy and expense-effectiveness of interventions, e.g., disease-altering treatment, need to be considered in the environment of the real world.

## Limitations

Several important limitations should be considered when interpreting the findings of this study. First, the estimates rely on modeled data from the GBD study, which, like all large-scale epidemiological models, faces particular constraints in regions with less developed health systems. In such settings, limitations in disease reporting, diagnostic access, and registry completeness can affect the accuracy of burden estimates and influence cross-regional comparisons. Second, because the GBD provides population-level rather than individual-level data, this analysis could not adjust for person-specific factors such as comorbidities, education, or detailed socioeconomic circumstances. This may obscure variations in risk and outcomes within broader populations. Third, the clinical differences between dementia subtypes create diagnostic challenges. These are the difficulties inherent in the data underlying and can lead to uncertainty in the burden estimates of the subtype. Fourth, the study has chosen a subgroup of adults (55 years and above) with the purpose of analyzing the disease burden in late adulthood, but it inevitably misses other types of these diseases at an earlier age, including multiple sclerosis. Consequently, the results fail to represent the entire clinical spectrum of the diseases. Lastly, the forecasts forwarded presuppose that the current trends will persist and lacks outlook attitudes to the possible future developments of the disease-modifying specifications that could adjust the long-term path and load of the examined diseases. Combining these points, the set of future research should be empowered in several ways to better its impact in the burden field. Finer data collection, combination of each risk factor individually and continuous optimization of diagnostic standards would enhance usefulness of such studies in informing responsive and specific health policies.

## Conclusion

The increasing burden of LOMS, PD, and AD/other dementia among older adults is a direct result of change in global demographic and population growth is the most important factor that increases the burden resulting through decomposition analysis. This epidemiological fact requires the change between the reactive to proactive health system approaches. These results position the need to use differentiated strategies to control the varying epidemiological patterns of each disease in response to the regional and demographic differences. The subsequent studies are to be dedicated to the elimination of the regional differences of load on one or another level of SDI, the decomposition of the factors of gender inequality, the creation of specific interventions related to the age category of ≥ 55 years. Finally, the efforts to reduce the consequences of these neurological cases will be achieved through a concerted effort through the clinical care, the policy of the public health, and through the research innovation, with special focus on the introduction of equitable access to prevention, diagnosis, and treatment among all populations.

## Supplementary Information

Below is the link to the electronic supplementary material.

Supplementary File 1 (DOCX 3.09 MB)

## Data Availability

All data generated or analyzed during the course of this study came from the GBD database.
