# Supplementary material for: The burden and trends of late-onset multiple sclerosis, Parkinson’s disease, Alzheimer’s disease and other dementias among adults aged 55 and older, spanning from 1990 to 2021, with projections through 2050
Source: Neurol Sci. 2026 Apr 18;47(5):439. doi: 10.1007/s10072-026-08986-6 (PMC13091877; doi:10.1007/s10072-026-08986-6)
Supplement: Supplementary file 1 — (DOCX 3.09 MB) [file 10072_2026_8986_MOESM1_ESM.docx]

**Supplemental material**

**The burden and trends of late-onset multiple sclerosis, Parkinson’s Disease, Alzheimer’s disease and other dementias among adults aged 55 and older, spanning from 1990 to 2021, with projections through 2050**

Xiao-jun Liu ^1^, Xiu-juan Mi^1^, Xin-yuan Yu^1^, Ning Deng^2^, Yuan-hong Lei^1^, Jian-zhong Shu^1^, Jun Tang^1^*

^1^Department of Brain Disease, Chongqing Hospital of Traditional Chinese Medicine, Chongqing 400021, China.

^2^Department of Rheumatology and Immunology, The First Affiliated Hospital (Southwest Hospital) of Army Medical University, Chongqing 400038, China.

* Correspondence: Jun Tang, tj123zyy@163.com

**Items**

**Table S1** Global burden of late-onset multiple sclerosis among adults aged≥55 years from 1990 to 2021, categorized by sex, GDB regions

**Table S2** Global burden of Parkinson’s Disease among adults aged≥55 years from 1990 to 2021, categorized by sex, GDB regions

**Table S3** Global burden of Alzheimer’s and other dementias among adults aged≥55 years from 1990 to 2021, categorized by sex, GDB regions

**Table S4** Global burden of late-onset multiple sclerosis, Parkinson’s disease, Alzheimer’s disease and other dementias among adults aged≥55 years from 1990 to 2021, categorized by SDI quintile

**Table S5** YLDs for Global Burden of LOMS, PD, AD and Other Dementias among Adults Aged ≥55 Years, 1990 to 2021

**Table S6** YLLs for Global Burden of LOMS, PD, AD and Other Dementias among Adults Aged ≥55 Years, 1990 to 2021

**Table S7** Global burden of late-onset multiple sclerosis among adults aged≥55 years from 1990 to 2021, categorized by countries

**Table S8** Global burden of Parkinson’s disease among adults aged≥55 years from 1990 to 2021, categorized by countries

**Table S9** Global burden of Alzheimer’s disease and other dementias among adults aged≥55 years from 1990 to 2021, categorized by countries

**Table S10** Decomposition of change in incidence globally and by SDI quintile, 1990 to 2021

**Table S11** Decomposition of change in DALYs globally and by SDI quintile, 1990 to 2021

**Table S12** Predicted age-standardized rates of LOMS, PD, AD and other dementias spanning 2022-2036, based on the Bayesian Age-Period-Cohort Model

**Table S13**. Predicted number of LOMS, PD, AD and other dementias spanning 2022-2050, based on the ARIMA Model

**Table S14**. Predicted age-standardized rates of LOMS, PD, AD and other dementias spanning 2022-2050, based on the ARIMA Model

**Fig. S1** Global Burden of incidence, prevalence, death, DALYs, YLDs, YLLs and their age-standardized rates for late-onset multiple sclerosis by Age Group in 2021

**Fig. S2** Global Burden of incidence, prevalence, death, DALYs, YLDs, YLLs and their age-standardized rates for Parkinson’s disease by Age Group in 2021

**Fig. S3** Global Burden of incidence, prevalence, death, DALYs, YLDs, YLLs and their age-standardized rates for Alzheimer’s disease and other dementias by Age Group in 2021

**Fig. S4** Global burden incidence, prevalence, death, DALYs, YLDs, YLLs and their age-standardized rates for late-onset multiple sclerosis among adults aged≥55 years in 2021, categorized by SDI

**Fig. S5** Global burden incidence, prevalence, death, DALYs, YLDs, YLLs and their age-standardized rates for Parkinson’s Disease among adults aged≥55 years in 2021, categorized by SDI

**Fig. S6** Global burden incidence, prevalence, death, DALYs, YLDs, YLLs and their age-standardized rates for Alzheimer’s disease and other dementias among adults aged≥55 years in 2021, categorized by SDI

**Fig. S7** Global burden incidence, prevalence, death, DALYs, YLDs, YLLs and their age-standardized rates for late-onset multiple sclerosis among adults aged≥55 years in 2021, categorized by GBD region

**Fig. S8** Global burden incidence, prevalence, death, DALYs, YLDs, YLLs and their age-standardized rates for Parkinson’s disease among adults aged≥55 years in 2021, categorized by GBD region

**Fig. S9** Global burden incidence, prevalence, death, DALYs, YLDs, YLLs and their age-standardized rates for Alzheimer’s disease and other dementias among adults aged≥55 years in 2021, categorized by GBD region

**Fig. S10** Global burden incidence, prevalence, death, DALYs, YLDs, YLLs and their age-standardized rates for late-onset multiple sclerosis among adults aged≥55 years in 2021, categorized by sex

**Fig. S11** Global burden incidence, prevalence, death, DALYs, YLDs, YLLs and their age-standardized rates for Parkinson’s disease among adults aged≥55 years in 2021, categorized by sex

**Fig. S12** Global burden incidence, prevalence, death, DALYs, YLDs, YLLs and their age-standardized rates for Alzheimer’s disease and other dementias among adults aged≥55 years in 2021, categorized by sex

**Fig. S13** Projected global burden of incidence, prevalence, death, DALYs, YLDs, YLLs cases and their age-standardized rates for late-onset multiple sclerosis among adults aged≥55 years by 2050, categorized by sex

**Fig. S14** Projected global burden of incidence, prevalence, death, DALYs, YLDs, YLLs cases and their age-standardized rates for Parkinson’s disease among adults aged≥55 years by 2050, categorized by sex

**Fig. S15** Projected global burden of incidence, prevalence, death, DALYs, YLDs, YLLs cases and their age-standardized rates for Alzheimer’s disease and other dementias among adults aged≥55 years by 2050, categorized by sex

**Table S1 Global burden of late-onset multiple sclerosis among adults aged≥55 years from 1990 to 2021, categorized by sex, GDB regions**

| **Characteristics** | **Incidence** | | | | | **Prevalence** | | | | | **Deaths** | | | | | **DALYs** | | | | |
| --- | --- | --- | --- | --- | --- | --- | --- | --- | --- | --- | --- | --- | --- | --- | --- | --- | --- | --- | --- | --- |
|  | **No.(95% UI) in 1990** | **ASR per 100000 (95% UI) in 1990** | **No.(95% UI) in 2021** | **ASR per 100000 (95% UI) in 2021** | **EAPC**  **(95% CI)** | **No.(95% UI) in 1990** | **ASR per 100000 (95% UI) in 1990** | **No.(95% UI) in 2021** | **ASR per 100000 (95% UI) in 2021** | **EAPC**  **(95% CI)** | **No.(95% UI) in 1990** | **ASR per 100000 (95% UI) in 1990** | **No.(95% UI) in 2021** | **ASR per 100000 (95% UI) in 2021** | **EAPC**  **(95% CI)** | **No.(95% UI) in 1990** | **ASR per 100000 (95% UI) in 1990** | **No.(95% UI) in 2021** | **ASR per 100000 (95% UI) in 2021** | **EAPC**  **(95% CI)** |
| **Global** | 2625.33 (2049.16 -3179.98) | 0.39 (0.27- 0.51) | 5035.5 (3914.89- 6067.94) | 0.34 (0.23- 0.45) | -0.41 (-0.45--0.37) | 343744.65 (298666 - 393824.9) | 51.52 (44.58 - 59.22) | 761107.58 (681925.57 - 849272.05) | 51.24 (45.73 - 57.21) | 0.18 (0.12 - 0.25) | 5054.36 (4824.16 - 5263.27) | 0.77 (0.72 - 0.81) | 11515.48 (10573.82 - 12081.66) | 0.78 (0.71 - 0.83) | 0.24 (0.16 - 0.32) | 207481 (181796.92 - 237118.03) | 30.54 (26.69 - 35) | 450155.06 (395614.81 - 508077.3) | 30.19 (26.44 - 34.23) | 0.16 (0.08 - 0.24) |
| **Sex** |  |  |  |  |  |  |  |  |  |  |  |  |  |  |  |  |  |  |  |  |
| Female | 1377.32 (1062.34 -1673.48) | 0.38 (0.26- 0.51) | 2554.17 (1963.81- 3093.85) | 0.32 (0.22- 0.44) | -0.5 (-0.55--0.44) | 238709.47 (207005.02 - 272487.66) | 66.49 (57.63 - 76.2) | 521961.87 (467538.51 - 581291.12) | 66.4 (59.45 - 73.85) | 0.21 (0.14 - 0.28) | 3120.47 (2964.85 - 3265.33) | 0.87 (0.81 - 0.92) | 7366.1 (6618.08 - 7854.75) | 0.93 (0.83 - 1.01) | 0.41 (0.31 - 0.51) | 132637.67 (114813.59 - 153319.7) | 36.63 (31.75 - 42.34) | 292954.81 (256501.82 - 329227.17) | 37.27 (32.36 - 42.23) | 0.27 (0.17 - 0.36) |
| Male | 1248.01 (980.63- 1509.57) | 0.4 (0.28- 0.51) | 2481.33 (1957.54- 2998.71) | 0.35 (0.25- 0.46) | -0.33 (-0.36--0.3) | 105035.18 (90332.99 - 121386.08) | 33.26 (28.43 - 38.42) | 239145.7 (212941.15 - 268243) | 34 (30.07 - 38.26) | 0.25 (0.19 - 0.31) | 1933.89 (1840.18 - 2010.86) | 0.64 (0.6 - 0.68) | 4149.37 (3892.34 - 4419.69) | 0.61 (0.56 - 0.66) | -0.01  (-0.06- 0.05) | 74843.33 (66545.06 - 84239.4) | 23.38 (20.8 - 26.53) | 157200.25 (139333.6 - 179788.13) | 22.26 (19.59 - 25.47) | 0.02 (-0.04 - 0.08) |
| **GBD Region** |  |  |  |  |  |  |  |  |  |  |  |  |  |  |  |  |  |  |  |  |
| Central Asia | 70.79 (60.92- 81.65) | 0.82 (0.63 - 1.01) | 119.87 (100.05 - 140.9) | 0.71 (0.53 - 0.9) | -0.57 (-0.73 - -0.4) | 7649.01 (6619.16 - 8625.3) | 94.34 (81.65 - 106.91) | 13949.03 (12342.9 - 15718.64) | 94.53 (83.61 - 106.96) | 0.1 (0.02 - 0.18) | 65.62 (56.63 - 75.61) | 0.98 (0.82 - 1.15) | 70.58 (53.44 - 85) | 0.68 (0.51 - 0.84) | -0.15 (-0.85 - 0.55) | 3000.27 (2403.28 - 3609.69) | 38.56 (30.22 - 47.95) | 4658.54 (3662.81 - 5785.8) | 33.52 (25.28 - 42.85) | -0.1 (-0.27 - 0.08) |
| Central Europe | 139.67 (112.16- 171.72) | 0.52 (0.37 - 0.69) | 101.5 (74.38 - 131.92) | 0.3 (0.19 - 0.44) | -1.93 (-2 - -1.85) | 18141.52 (16139.21 - 20341.37) | 66.84 (58.8 - 75.46) | 24669.44 (22514.17 - 27123.45) | 69.23 (62.86 - 76.15) | 0.1 (0.03 - 0.16) | 648.39 (611.72 - 691.96) | 2.42 (2.23 - 2.64) | 717.17 (650.17 - 783.59) | 1.94 (1.73 - 2.17) | -0.82 (-0.98 - -0.67) | 21379.24 (19810.51 - 23095.98) | 78.22 (71.48 - 85.85) | 23540.43 (21387.82 - 25896.68) | 66.35 (59.25 - 73.82) | -0.58 (-0.68 - -0.48) |
| Eastern Europe | 175.4 (136.11- 222.08) | 0.36 (0.24 - 0.5) | 65.59 (48.37 - 88.34) | 0.11 (0.07 - 0.17) | -3.69 (-3.8 - -3.59) | 19684.24 (16489.72 - 23431.84) | 39.48 (32.75 - 47.06) | 26519.56 (24126.15 - 29046.37) | 42.79 (38.71 - 47.09) | 0.26 (0.25 - 0.28) | 465.66 (444.11 - 488.19) | 0.98 (0.92 - 1.05) | 480.39 (432.03 - 524.09) | 0.78 (0.69 - 0.86) | -1.75 (-2.12 - -1.37) | 16755.19 (14986.43 - 18676.49) | 33.86 (30.12 - 38.08) | 19182.88 (16889.38 - 21428.57) | 31.28 (27.33 - 35.27) | -0.9 (-1.13 - -0.67) |
| Australasia | 16.11 (11.63- 19.72) | 0.42 (0.26 - 0.57) | 34.41 (25.01 - 43.5) | 0.4 (0.25 - 0.56) | -0.17 (-0.2 - -0.14) | 3160.63 (2774.22 - 3588.92) | 80.74 (70.46 - 91.65) | 10767.68 (9326.78 - 12330.81) | 123.92 (107.49 - 142.04) | 1.47 (1.12 - 1.82) | 68.04 (60.4 - 76.74) | 1.69 (1.45 - 1.98) | 163.44 (138.63 - 188.01) | 1.8 (1.47 - 2.14) | 0.1 (-0.05 - 0.26) | 2379.97 (2071.74 - 2724.03) | 60.5 (50.26 - 72.11) | 6247.49 (5269.67 - 7278.38) | 72.11 (57.65 - 88.16) | 0.52 (0.33 - 0.72) |
| High-income Asia Pacific | 98.51 (68.61- 128.59) | 0.28 (0.16 - 0.41) | 189.15 (136.37 - 242.64) | 0.27 (0.16 - 0.4) | -0.05 (-0.07 - -0.03) | 6311.24 (5145.5 - 7637.72) | 17.94 (14.44 - 21.85) | 14037.06 (11485.15 - 16894.3) | 20.11 (16.34 - 24.23) | 0.36 (0.3 - 0.42) | 39.45 (37 - 41.62) | 0.11 (0.1 - 0.12) | 78.1 (68.6 - 84.94) | 0.1 (0.09 - 0.12) | -0.19 (-0.4 - 0.02) | 2723.8 (2158.62 - 3472.48) | 7.72 (6.07 - 9.86) | 5535.55 (4267.19 - 7181.76) | 8.02 (6.2 - 10.45) | 0.15 (0.07 - 0.24) |
| High-income North America | 280.75 (222.88- 349.44) | 0.51 (0.35 - 0.69) | 379.28 (287.85 - 487.61) | 0.35 (0.23 - 0.5) | -1.19 (-1.55 - -0.82) | 110984.43 (96218.99 - 126729.04) | 193.73 (168.02 - 221.1) | 219409.37 (203405.89 - 236341.74) | 197.31 (182.88 - 212.54) | 0.43 (0.31 - 0.55) | 1132.73 (1070.14 - 1183.78) | 1.95 (1.83 - 2.06) | 4114.89 (3788.7 - 4379.05) | 3.57 (3.23 - 3.84) | 1.93 (1.56 - 2.3) | 53933.5 (46067.99 - 62503.81) | 95.47 (81.27 - 111.22) | 144175.25 (128355.98 - 158740.92) | 128.75 (113.54 - 142.8) | 1.13 (0.9 - 1.36) |
| Southern Latin America | 24.66 (17.64- 31.61) | 0.31 (0.18 - 0.46) | 39.87 (28.12 - 51.93) | 0.27 (0.16 - 0.41) | -0.37 (-0.41 - -0.33) | 3521.09 (2968.45 - 4110.07) | 44.04 (36.97 - 51.8) | 6546.53 (5554 - 7688.11) | 44.81 (37.86 - 52.74) | 0.02 (-0.01 - 0.05) | 70 (65.68 - 75.28) | 0.88 (0.77 - 1.01) | 82.58 (73.36 - 91.52) | 0.56 (0.47 - 0.65) | -1.57 (-1.86 - -1.28) | 2625.88 (2300.96 - 2980.67) | 32.76 (27.62 - 38.83) | 3642.18 (3027.97 - 4320.98) | 25 (20.1 - 30.95) | -0.96 (-1.15 - -0.77) |
| Western Europe | 461.15 (363.89- 572.58) | 0.49 (0.33 - 0.66) | 693.7 (543.36 - 870.55) | 0.49 (0.32 - 0.67) | 0 (-0.04 - 0.03) | 123642.01 (107442.27 - 140429.24) | 128.89 (112.17 - 146.83) | 262161.93 (230631.8 - 295472.73) | 180.06 (159.01 - 202.91) | 1.13 (1.05 - 1.22) | 2277.57 (2173.41 - 2363.85) | 2.33 (2.17 - 2.47) | 4461.68 (4028.2 - 4767.51) | 2.96 (2.66 - 3.21) | 1.26 (1.11 - 1.41) | 84245.56 (75218.34 - 94620.39) | 88.49 (78.64 - 100.06) | 163160.44 (143684.85 - 184511.61) | 114.19 (99.91 - 129.59) | 1.16 (1.07 - 1.25) |
| Andean Latin America | 12.46 (9.69- 15.03) | 0.37 (0.26 - 0.47) | 40.13 (32.87 - 47.4) | 0.41 (0.3 - 0.5) | 0.31 (0.26 - 0.35) | 469.97 (375.08 - 565.63) | 13.86 (11.01 - 16.83) | 2161.46 (1750.68 - 2582.53) | 21.76 (17.59 - 26.07) | 1.6 (1.51 - 1.7) | 6.9 (5.18 - 8.7) | 0.21 (0.15 - 0.3) | 40.21 (30.1 - 53.68) | 0.41 (0.27 - 0.63) | 2.99 (2.55 - 3.43) | 295.68 (238.69 - 371.86) | 8.72 (6.67 - 11.41) | 1516.88 (1222.75 - 1900.84) | 15.26 (11.31 - 20.52) | 2.31 (2.05 - 2.57) |
| Caribbean | 15.47 (11.49- 18.81) | 0.36 (0.23 - 0.47) | 31.76 (23.49 - 39.09) | 0.34 (0.21 - 0.47) | -0.16 (-0.17 - -0.15) | 775.5 (621.43 - 939.1) | 17.89 (14.28 - 21.76) | 2199.11 (1821.86 - 2598.21) | 23.69 (19.41 - 28.22) | 0.92 (0.85 - 1) | 21.52 (19.59 - 23.64) | 0.5 (0.43 - 0.58) | 64.23 (55.38 - 72.79) | 0.69 (0.57 - 0.83) | 1.34 (1.19 - 1.5) | 758.8 (677.76 - 867.97) | 17.47 (15.2 - 20.57) | 2227.71 (1939.31 - 2585.56) | 23.94 (20.01 - 28.53) | 1.21 (1.1 - 1.32) |
| Central Latin America | 47.51 (36.01- 58.62) | 0.35 (0.23 - 0.46) | 155.59 (120.38 - 187.22) | 0.36 (0.25 - 0.47) | 0.15 (0.11 - 0.18) | 1840.6 (1466.27 - 2257.08) | 13.25 (10.5 - 16.3) | 9193.61 (7608.42 - 10906.51) | 21.27 (17.43 - 25.33) | 1.61 (1.51 - 1.71) | 41.56 (39.84 - 43.19) | 0.31 (0.29 - 0.34) | 284.33 (252.97 - 320.56) | 0.67 (0.59 - 0.76) | 2.44 (1.99 - 2.9) | 1525.63 (1355.63 - 1759.43) | 11.01 (9.68 - 12.69) | 9455.06 (8356.12 - 10717.36) | 21.82 (19.03 - 24.93) | 2.2 (1.84 - 2.56) |
| Tropical Latin America | 84.01 (70.36- 99.63) | 0.54 (0.4 - 0.66) | 247.58 (205 - 296.62) | 0.55 (0.4 - 0.68) | 0.05 (0.03 - 0.08) | 6895.59 (5674.99 - 8114.77) | 44.64 (36.73 - 52.75) | 26026.95 (21796.25 - 30473.49) | 58.54 (48.9 - 68.7) | 1.03 (0.9 - 1.15) | 57.22 (53.38 - 60.52) | 0.43 (0.39 - 0.47) | 219.8 (195.86 - 237.25) | 0.51 (0.44 - 0.56) | -0.2 (-0.81 - 0.42) | 3040.33 (2474.43 - 3749.28) | 20.13 (16.28 - 24.77) | 11375.76 (9297.22 - 13745.92) | 25.6 (20.76 - 31.12) | 0.52 (0.24 - 0.8) |
| North Africa and Middle East | 119.65 (89.14- 148.44) | 0.41 (0.27 - 0.54) | 344.59 (261.92 - 430.11) | 0.44 (0.29 - 0.57) | 0.23 (0.21 - 0.24) | 20658.47 (18006.89 - 23517.03) | 72.09 (62.72 - 82.27) | 76088.06 (66503.96 - 85805.64) | 99.29 (86.93 - 112.3) | 1.26 (1.2 - 1.33) | 83.93 (40.25 - 125.89) | 0.32 (0.14 - 0.52) | 423.49 (339.78 - 504.5) | 0.6 (0.44 - 0.78) | 2.28 (2.11 - 2.44) | 7269.49 (5444.77 - 9449.39) | 25.11 (18.34 - 33.27) | 28781.89 (23595.57 - 35413.33) | 37.28 (29.73 - 46.54) | 1.49 (1.42 - 1.56) |
| South Asia | 311.83 (223.71- 394.06) | 0.33 (0.2 - 0.45) | 799.94 (581.63 - 1005.17) | 0.32 (0.2 - 0.45) | -0.01 (-0.04 - 0.01) | 12203.01 (9723.77 - 14936.19) | 12.09 (9.45 - 14.99) | 38703.52 (31059.72 - 46708.51) | 15.14 (12.06 - 18.35) | 0.8 (0.76 - 0.84) | 29.94 (10.21 - 49.54) | 0.03 (0.01 - 0.06) | 147.7 (97.44 - 194.96) | 0.06 (0.04 - 0.08) | 2 (1.84 - 2.15) | 4209.25 (2933.43 - 5913.76) | 4.18 (2.87 - 5.9) | 14552.79 (10777.37 - 19331.84) | 5.7 (4.19 - 7.64) | 1.04 (0.98 - 1.1) |
| East Asia | 528.93 (408.58- 643.68) | 0.36 (0.26 - 0.45) | 1191.83 (926.04 - 1475.98) | 0.31 (0.22 - 0.41) | -0.58 (-0.61 - -0.55) | 3036.65 (2285.37 - 4010.71) | 1.96 (1.35 - 2.71) | 14477.4 (11418.13 - 17812.64) | 3.64 (2.69 - 4.74) | 1.8 (1.65 - 1.95) | 20.18 (11.69 - 30.42) | 0.02 (0.01 - 0.03) | 67.44 (53.81 - 83.56) | 0.02 (0.01 - 0.02) | -0.15 (-0.72 - 0.43) | 1351.16 (969.88 - 1865.28) | 0.89 (0.61 - 1.26) | 5622.23 (4167.4 - 7585.81) | 1.42 (1.03 - 1.91) | 1.13 (0.96 - 1.29) |
| Oceania | 1.29 (0.9 - 1.7) | 0.28 (0.17 - 0.4) | 3.11 (2.17 - 4.23) | 0.26 (0.17 - 0.38) | -0.21 (-0.23 - -0.19) | 9.35 (6.74 - 12.49) | 1.81 (1.2 - 2.57) | 26.01 (18.88 - 34.38) | 1.96 (1.33 - 2.74) | 0.18 (0.14 - 0.22) | 0 (0 - 0) | 0 (0 - 0) | 0 (0 - 0) | 0 (0 - 0) | 0.41 (0.3 - 0.52) | 2.64 (1.63 - 3.99) | 0.51 (0.3 - 0.8) | 7.35 (4.6 - 10.87) | 0.55 (0.33 - 0.85) | 0.18 (0.14 - 0.22) |
| Southeast Asia | 134.09 (99.34- 170.47) | 0.33 (0.22 - 0.43) | 363.9 (281.23 - 454.78) | 0.33 (0.23 - 0.43) | 0.08 (0.06 - 0.1) | 1265.02 (942.01 - 1640.27) | 2.83 (1.99 - 3.85) | 4319.72 (3313.03 - 5445.15) | 3.64 (2.67 - 4.78) | 0.82 (0.78 - 0.86) | 7.6 (4.31 - 10.25) | 0.02 (0.01 - 0.03) | 37.18 (31.41 - 43.39) | 0.03 (0.03 - 0.04) | 1.75 (1.51 - 1.99) | 544.3 (384.95 - 753.53) | 1.23 (0.84 - 1.72) | 2137.34 (1689.98 - 2731.21) | 1.81 (1.4 - 2.35) | 1.2 (1.13 - 1.28) |
| Central Sub-Saharan Africa | 8.81 (5.85- 12.17) | 0.23 (0.13 - 0.35) | 20.66 (13.57 - 29.04) | 0.23 (0.13 - 0.34) | -0.07 (-0.08 - -0.06) | 267.24 (204.39 - 338.62) | 6.42 (4.8 - 8.26) | 730.87 (565.86 - 913.62) | 7.42 (5.65 - 9.44) | 0.42 (0.36 - 0.48) | 1.64 (0.64 - 2.76) | 0.05 (0.01 - 0.1) | 6.58 (3.02 - 10.7) | 0.08 (0.03 - 0.16) | 1.55 (1.41 - 1.68) | 118.73 (78.07 - 167.55) | 2.9 (1.83 - 4.39) | 379.42 (256.13 - 511.97) | 3.91 (2.5 - 5.92) | 0.9 (0.81 - 0.99) |
| Eastern Sub-Saharan Africa | 31.86 (21.71- 42.32) | 0.26 (0.15 - 0.39) | 68.65 (47.91 - 91.26) | 0.25 (0.15 - 0.37) | -0.07 (-0.09 - -0.05) | 960.08 (739.99 - 1202.4) | 7.37 (5.57 - 9.39) | 2403.53 (1877.17 - 2963.77) | 8.41 (6.48 - 10.52) | 0.4 (0.34 - 0.46) | 4.74 (1.23 - 7.86) | 0.04 (0.01 - 0.07) | 17.91 (6.84 - 26.93) | 0.07 (0.03 - 0.11) | 1.62 (1.56 - 1.67) | 390.53 (242.43 - 559.57) | 3.03 (1.82 - 4.41) | 1124.75 (713.02 - 1524.81) | 3.98 (2.49 - 5.55) | 0.82 (0.78 - 0.87) |
| Southern Sub-Saharan Africa | 13.95 (10.15- 17.56) | 0.32 (0.19 - 0.44) | 31.87 (23.24 - 39.69) | 0.33 (0.2 - 0.45) | 0.19 (0.15 - 0.23) | 572.63 (452.94 - 695.49) | 12.6 (9.86 - 15.52) | 1457.34 (1168.5 - 1743.14) | 14.57 (11.64 - 17.63) | 0.49 (0.41 - 0.56) | 11.6 (6.59 - 15.65) | 0.26 (0.14 - 0.39) | 37.57 (29.33 - 46.08) | 0.39 (0.27 - 0.53) | 1.06 (0.79 - 1.33) | 455.86 (314.38 - 586.82) | 9.98 (6.61 - 13.46) | 1377.82 (1133.95 - 1646.26) | 13.58 (10.54 - 17.3) | 0.86 (0.69 - 1.03) |
| Western Sub-Saharan Africa | 48.43 (35.24- 60.2) | 0.33 (0.21 - 0.45) | 112.54 (82.18 - 140.38) | 0.34 (0.22 - 0.47) | 0.17 (0.13 - 0.2) | 1696.37 (1356.54 - 2075.92) | 11.16 (8.72 - 13.84) | 5259.39 (4382.31 - 6206.09) | 15.37 (12.61 - 18.35) | 1.12 (1.08 - 1.17) | 0.06 (0.02 - 0.09) | 0 (0 - 0) | 0.2 (0.11 - 0.28) | 0 (0 - 0) | 1.57 (1.43 - 1.71) | 475.19 (321.21 - 674.16) | 3.13 (2.08 - 4.54) | 1453.33 (998.74 - 2032.46) | 4.25 (2.85 - 5.97) | 1.07 (1.03 - 1.12) |

**Abbreviations**: UI, uncertainty interval; ASR, age-standerised rate per 100,000; CI, confidence interval; DALYs, disability-adjusted life-year; EAPC, estimated annual percentage change.

**Table S2 Global burden of Parkinson’s Disease among adults aged≥55 years from 1990 to 2021, categorized by sex, GDB regions**

| **Characteristics** | **Incidence** | | | | | **Prevalence** | | | | | **Deaths** | | | | | **DALYs** | | | | |
| --- | --- | --- | --- | --- | --- | --- | --- | --- | --- | --- | --- | --- | --- | --- | --- | --- | --- | --- | --- | --- |
|  | **No.(95% UI) in 1990** | **ASR per 100000 (95% UI) in 1990** | **No.(95% UI) in 2021** | **ASR per 100000 (95% UI) in 2021** | **EAPC**  **(95% CI)** | **No.(95% UI) in 1990** | **ASR per 100000 (95% UI) in 1990** | **No.(95% UI) in 2021** | **ASR per 100000 (95% UI) in 2021** | **EAPC**  **(95% CI)** | **No.(95% UI) in 1990** | **ASR per 100000 (95% UI) in 1990** | **No.(95% UI) in 2021** | **ASR per 100000 (95% UI) in 2021** | **EAPC**  **(95% CI)** | **No.(95% UI) in 1990** | **ASR per 100000 (95% UI) in 1990** | **No.(95% UI) in 2021** | **ASR per 100000 (95% UI) in 2021** | **EAPC**  **(95% CI)** |
| **Global** | 371666.07 (324814.23- 419673.09) | 60.57 (45.09- 77.62) | 1184055.27 (1039201.25- 1333546.01) | 82.17 (60.36- 106.06) | 1.03 (0.99- 1.06) | 2825059.24 (2441505.24- 3270986.85) | 467.82 (377.06- 574.41) | 10757500.76 (9480906.72- 12341387.84) | 751.69 (614.51- 914.06) | 1.53 (1.5- 1.55) | 145055.51 (133004.91 -155103.22) | 26.92 (24.12- 29.23) | 382945.97 (342283.22 - 411719.04) | 28.08 (24.67- 30.71) | 0.19 (0.14- 0.25) | 2676445.49 (2481700.75- 2877899.87) | 458.3 (415.68- 501.64) | 7095613.7 (6403407.26- 7732755.15) | 504.5 (446.61- 557.27) | 0.33 (0.29- 0.37) |
| **Sex** |  |  |  |  |  |  |  |  |  |  |  |  |  |  |  |  |  |  |  |  |
| Female | 174852.78 (153168.7- 197176.74) | 49.79 (36.82- 64.04) | 511862.03 (450415.56- 578183.51) | 64.75 (47.13- 83.95) | 0.88 (0.85- 0.91) | 1455324.28 (1262208.71 - 1678019.54) | 420.04 (340.68 - 514.14) | 4923028.94 (4348060.91 - 5613698.36) | 622.11 (509.84 - 752.72) | 1.25 (1.23 - 1.27) | 68590.7 (61622.19 - 74571.09) | 21.08 (18.12- 23.9) | 166299.15 (141395.27- 182546.63) | 20.96 (17.28 - 24.03) | 0.01 (-0.03 - 0.04) | 1254275.36 (1137425.56 - 1362780.51) | 367.29 (321.36 - 412.81) | 3056259.48 (2710949.41 - 3383192.91) | 386.16 (329.5 - 440.7) | 0.15 (0.12 - 0.18) |
| Male | 196813.29 (171834.12- 222983.45) | 76.97 (57.71- 98.4) | 672193.24 (589403.23- 759780.42) | 104.42 (77.03- 134.52) | 1.03 (1- 1.07) | 1369734.97 (1177469.22 - 1598045.12) | 538.04 (429.86 - 666.43) | 5834471.82 (5110779.02 - 6720292.14) | 913.55 (745.29 - 1115.96) | 1.71 (1.69 - 1.74) | 76464.8 (70720.67 - 83152.59) | 36.69 (33.38 - 39.98) | 216646.83 (195981.35 - 235604.69) | 38.41 (34.09 - 41.92) | 0.22 (0.14 - 0.29) | 1422170.13 (1314308.92 - 1550716.06) | 599.1 (546.16 - 653.05) | 4039354.22 (3648811.8 - 4447316.93) | 663.44 (590.52 - 731.81) | 0.37 (0.31 - 0.43) |
| **GBD Region** |  |  |  |  |  |  |  |  |  |  |  |  |  |  |  |  |  |  |  |  |
| Central Asia | 4005.41 (3542.59- 4442.6) | 55.79 (41.36- 71.83) | 7938.56 (7228.43- 8673.26) | 67.32 (54.49- 81.61) | 0.66 (0.62- 0.7) | 30217.25 (25216.69 - 35546.48) | 424.88 (328.23 - 538.4) | 54993.88 (46910.75 - 63568.72) | 477.95 (385.56 - 574.99) | 0.4 (0.31 - 0.5) | 1458.4 (1273.85 - 1715.11) | 22 (19.04 - 26.15) | 2362.97 (2145.99 - 2577.22) | 23.62 (21.08 - 25.87) | 0.27 (0.16 - 0.38) | 26536.11 (23572.27 - 30283.69) | 384.94 (337.53 - 447.9) | 44442.24 (40536.9 - 48637.06) | 413.11 (371.5 - 455.08) | 0.22 (0.13 - 0.31) |
| Central Europe | 16126.03 (14699.17- 17575.01) | 63.75 (51.2- 77.43) | 27344.18 (25201.75- 29400.82) | 67.2 (54.42- 80.44) | 0.15 (0.13- 0.18) | 120145.94 (105513.89 - 135313.93) | 482.58 (402.78 - 572.48) | 220473.49 (196082.99 - 241586.41) | 536.81 (458.74 - 621.03) | 0.3 (0.26 - 0.34) | 6511.66 (6206.35 - 6714.76) | 28.59 (26.91 - 29.8) | 12050.85 (10992.7 - 12808.83) | 28.74 (26 - 30.76) | 0 (-0.05 - 0.05) | 116024.08 (109723.96 - 121800.5) | 481.29 (449.81 - 511.2) | 202024.05 (186746.52 - 216859.44) | 486.16 (442.64 - 526.24) | 0 (-0.03 - 0.03) |
| Eastern Europe | 26812.02 (22606.62- 31351.33) | 56.96 (39.42- 76.77) | 36748.42 (31216.26- 41835.23) | 58.5 (41.26- 77.45) | -0.01 (-0.09- 0.08) | 231706.62 (192566.24 - 278518.83) | 497.97 (385.25 - 636.43) | 315130.95 (262129.14 - 374421.65) | 504.8 (392.12 - 636.22) | -0.05 (-0.17 - 0.07) | 9508.73 (8941.09 - 9911.42) | 22.58 (20.97 - 23.78) | 15134.3 (13761.05 - 16313.86) | 24.52 (22.11 - 26.51) | 0.03 (-0.09 - 0.14) | 180862.44 (167985.69 - 195500.91) | 402.53 (368.45 - 437.86) | 265454.36 (242760.44 - 289149.4) | 428.66 (386.56 - 469.95) | 0.02 (-0.07 - 0.11) |
| Australasia | 2216.5 (1959.62- 2410.88) | 53.79 (41.88- 66.35) | 6490.48 (5920.34- 7281.2) | 66.34 (52.5- 81.09) | 0.73 (0.67- 0.79) | 15244.57 (12699.8 - 17591.71) | 368.93 (288.32 - 449.73) | 48586.13 (41445.15 - 58372.66) | 488.79 (391.2 - 608.01) | 0.94 (0.87 - 1.01) | 1040.1 (956.65 - 1086.08) | 26.35 (23.89 - 28.01) | 2890.06 (2490.84 - 3111.09) | 27.39 (23.5 - 29.77) | 0.25 (0.11 - 0.38) | 17610.31 (16441.98 - 18672) | 431.32 (393.74 - 465.39) | 45629.75 (40431.33 - 49156.12) | 447.04 (391.97 - 490.98) | 0.2 (0.08 - 0.32) |
| High-income Asia Pacific | 14087.97 (11812.84- 16377.68) | 42.32 (28.96- 57.34) | 38924.82 (34297.58- 43783) | 43.84 (32.15- 56.45) | 0.48 (0.38- 0.58) | 114657.2 (95822.1 - 136563.41) | 344.49 (264.74 - 441.44) | 280756.62 (241278.45 - 326217.74) | 323.79 (256.29 - 401.67) | 0.39 (0.2 - 0.59) | 5845.17 (5304.74 - 6208.65) | 19.28 (17.18 - 20.74) | 22381.69 (18235.27 - 24723.45) | 20.87 (17.27 - 23.08) | 0.54 (0.44 - 0.64) | 104754.55 (96297.7 - 112560.33) | 325.9 (293.72 - 355.53) | 329730.2 (281791.45 - 362046.8) | 340.93 (291.39 - 378.02) | 0.47 (0.37 - 0.58) |
| High-income North America | 39625.23 (33277.79- 45759.06) | 62.24 (43.66- 83.44) | 98598.65 (91048.94- 106720.9) | 82.79 (67.84- 98.98) | 0.85 (0.78- 0.92) | 313767.48 (262232.59 - 371299.51) | 492.72 (381.71 - 623.71) | 818275.8 (760056.17 - 881403.05) | 683.37 (605.99 - 766.75) | 0.97 (0.89 - 1.05) | 14612.33 (12919.63 - 15391.77) | 22.72 (20.01 - 24.03) | 41246.71 (35401.11 - 44177.8) | 33.5 (28.77 - 35.91) | 1.37 (1.25 - 1.5) | 251637.92 (229644.04 - 270573.23) | 391.84 (350.37 - 427.22) | 673888.35 (599071.4 - 724237.04) | 556.46 (492.99 - 602.5) | 1.19 (1.09 - 1.29) |
| Southern Latin America | 5610.71 (5133.11- 6033.87) | 72.88 (59.12- 85.73) | 12228.93 (11106.27- 13759.32) | 79.47 (63.51- 97.14) | 0.4 (0.35- 0.44) | 40026.75 (35108.96 - 44061.55) | 530.88 (438.31 - 622.97) | 94363.03 (81016.2 - 111264.13) | 610.97 (489.51 - 752.23) | 0.6 (0.5 - 0.69) | 2146.72 (2024.1 - 2221.6) | 30.34 (27.98 - 32.06) | 4431.76 (4001.2 - 4687.47) | 28.15 (25.17 - 30.09) | -0.01 (-0.11 - 0.09) | 37920.91 (35722.67 - 40147.74) | 510.02 (469.64 - 547.93) | 75140.65 (69062.68 - 80657.52) | 481.99 (434.03 - 526.36) | 0.01 (-0.06 - 0.08) |
| Western Europe | 80981.26 (74599.65- 87369.18) | 76.69 (61.78- 91.74) | 168560.41 (155557.41- 181845.4) | 97.19 (78.8- 116.36) | 0.67 (0.58- 0.77) | 691320.86 (628284.09 - 762151.12) | 651.77 (559.52 - 751.83) | 1545869.73 (1390770.48 - 1703667.27) | 843.86 (723.27 - 972.9) | 0.69 (0.56 - 0.82) | 29041.99 (26514.87 - 30183.66) | 26.89 (24.45 - 28.11) | 56812.29 (48423.86 - 61018.85) | 27.74 (23.79 - 29.78) | 0.39 (0.3 - 0.48) | 507842.22 (470582.35 - 541426.12) | 470.18 (428.01 - 506.7) | 951627.89 (847563.3 - 1033667.07) | 500.9 (439.89 - 552.24) | 0.39 (0.31 - 0.47) |
| Andean Latin America | 1636.72 (1443.83- 1840.07) | 54.51 (41.89- 69.31) | 7668.93 (6750.75- 8696.86) | 80.36 (59.08- 105.8) | 1.27 (1.25- 1.29) | 13628.52 (11494.53 - 16201.06) | 437.2 (340.09 - 549.35) | 77014.55 (64382.58 - 91960.47) | 802.12 (635.24 - 1011.73) | 1.94 (1.9 - 1.98) | 843.58 (743.25 - 940.72) | 29.72 (25.35 - 34.14) | 2816.36 (2366.52 - 3354.01) | 30.43 (24.84 - 36.77) | 0.18 (0.07 - 0.28) | 14579.89 (13002.28 - 16007.09) | 490.46 (422.87 - 559.66) | 51171.94 (44001.66 - 59259.33) | 542.57 (451.92 - 641.86) | 0.36 (0.28 - 0.45) |
| Caribbean | 1742.65 (1581.84- 1887.89) | 42.57 (34.46- 51.6) | 4654.54 (4310.81- 5044.73) | 50.61 (40.71- 61.99) | 0.52 (0.48- 0.56) | 11735.36 (10057.71 - 13569.09) | 279.59 (220.38 - 342.18) | 34631.74 (30347.76 - 39699.29) | 377.47 (302.12 - 457.09) | 0.9 (0.82 - 0.97) | 1017.24 (948.81 - 1069.68) | 26 (23.81 - 27.86) | 2479.08 (2194.82 - 2741.31) | 27.05 (23.54 - 30.37) | 0.2 (0.14 - 0.26) | 17070.21 (15998.53 - 18072.76) | 414.44 (379.77 - 446.87) | 40793.36 (36704.94 - 45131.55) | 446.76 (393.99 - 499.58) | 0.3 (0.25 - 0.34) |
| Central Latin America | 5664.19 (5025.74- 6316.31) | 47.82 (35.94- 60.92) | 24746.53 (22184- 27438.07) | 61.49 (46.37- 78.22) | 0.62 (0.53- 0.71) | 42119.01 (36925.89 - 48669.14) | 342.12 (271.95 - 423.82) | 207482.68 (182511.15 - 238546.13) | 513.48 (415.16 - 622.39) | 1 (0.87 - 1.13) | 2850.84 (2687.7 - 2931.63) | 26.45 (24.67 - 27.51) | 9518.38 (8415.79 - 10406.73) | 24.77 (21.8 - 27.24) | -0.22 (-0.28 - -0.16) | 48400.19 (45964.6 - 50616.89) | 418.13 (389.88 - 442.71) | 168701.47 (151930.39 - 186290.19) | 427.79 (381.46 - 476.05) | 0 (-0.06 - 0.05) |
| Tropical Latin America | 6264.21 (5119.39- 7404.36) | 48.15 (32.21- 66.01) | 23277.66 (19535.94- 26571.38) | 55.13 (37- 76.01) | 0.37 (0.32- 0.42) | 46719.72 (38583.62 - 56293.57) | 348.61 (259.42 - 455.96) | 196993.52 (164497.57 - 233840.46) | 464.85 (353.11 - 595.98) | 0.81 (0.71 - 0.9) | 2781.08 (2536.97 - 2900.06) | 24.81 (22.23 - 26.18) | 9985.08 (8732.92 - 10692.47) | 24.86 (21.47 - 26.73) | 0.26 (0.18 - 0.33) | 49733.71 (46370.68 - 52657.06) | 401.73 (364.48 - 431.49) | 172116.92 (155372.58 - 185279.91) | 417.84 (368.96 - 456.77) | 0.28 (0.23 - 0.34) |
| North Africa and Middle East | 12610.18 (11099.5- 14087.45) | 56.01 (42.68- 70.44) | 46322.14 (40574.2- 51992.55) | 72.75 (54.87- 92.42) | 0.89 (0.85- 0.93) | 82570.49 (68885.61 - 96875.35) | 364.16 (278.75 - 459.07) | 349610.57 (297657.34 - 405500.16) | 557.24 (436.44 - 687.85) | 1.43 (1.37 - 1.48) | 6214.93 (5516.41 - 6988.92) | 33.52 (28.47 - 39.58) | 16064.45 (14150.61 - 17787.21) | 29.93 (25.45 - 33.81) | -0.26 (-0.37 - -0.15) | 111866.67 (99456.36 - 124073.2) | 532.66 (457.67 - 616.84) | 294527.45 (261660.61 - 323590.26) | 497.83 (431.54 - 560.83) | -0.15 (-0.24 - -0.07) |
| South Asia | 40929.95 (33812.84- 48038.39) | 54.3 (37.86- 72.51) | 139453.72 (116766.16- 161540.22) | 62.26 (43.12- 83.21) | 0.54 (0.47- 0.6) | 270686.18 (222022.43 - 325000.07) | 345.24 (258.08 - 454.17) | 1088418.21 (899524.43 - 1310620.36) | 489.54 (373.3 - 626.37) | 1.3 (1.21 - 1.38) | 15117.9 (11868.84 - 19610.64) | 24.27 (18.45 - 31.67) | 52438.22 (43687.01 - 61703.86) | 27.49 (22.17 - 32.87) | 0.46 (0.3 - 0.62) | 293996.02 (240637.68 - 369734.21) | 412.11 (325.52 - 524.11) | 984858.04 (835256.97 - 1131174.3) | 469.37 (388.92 - 552.59) | 0.44 (0.33 - 0.55) |
| East Asia | 82100.07 (67246.55- 97651.19) | 68.6 (46.67- 94.3) | 447297.71 (365852.93- 536161.38) | 122.35 (78.63- 173.08) | 2.08 (1.99- 2.17) | 593270.99 (486628.51 - 717620.44) | 494.63 (369.75 - 650.33) | 4756224.11 (4003119.8- 5649763.94) | 1320.76 (1026.74- 1682.72) | 3.24 (3.12 - 3.36) | 32588.41 (28755.73 - 36152.99) | 35.22 (29.55 - 40.13) | 94010.72 (78054.95 - 109862.52) | 29.3 (23.98 - 35.05) | -0.72 (-0.88 - -0.56) | 647852.93 (570847.12 - 722770.8) | 584.55 (498.35 - 665.93) | 2098213.73 (1776506.31 - 2431482.43) | 600.8 (498.93 - 709.99) | 0 (-0.11 - 0.11) |
| Oceania | 210.54 (179.73- 245.41) | 57.61 (43.14- 75.56) | 567.99 (484.1-  644.76) | 59.71 (44.44- 77.01) | 0.22 (0.17- 0.26) | 1458.59 (1176.39 - 1754.28) | 412.17 (308.62 - 533.04) | 4159.96 (3403.73 - 4930.14) | 449.83 (337.41 - 570.84) | 0.38 (0.31 - 0.46) | 86.8 (69.77 - 107.7) | 30.95 (24.46 - 39.27) | 209.31 (167.87 - 268.48) | 27.44 (21.53 - 35.9) | -0.42 (-0.48 - -0.37) | 1836.21 (1496.79 - 2241.28) | 542.79 (435.57 - 673.99) | 4337.18 (3511.7 - 5500.04) | 491.14 (393.62 - 629.63) | -0.34 (-0.39 - -0.29) |
| Southeast Asia | 17579.12 (15537.01- 19865.8) | 50.8 (37.96- 64.76) | 59508.16 (53280.32- 66392.85) | 61.79 (47.42- 77.76) | 0.68 (0.65- 0.7) | 122370.68 (104314.25 - 142788.26) | 349.15 (272.69 - 440) | 444958.07 (386908.96 - 515741.45) | 465.04 (375.05 - 570.34) | 0.97 (0.93 - 1.02) | 7521.47 (6567.03 - 8687.98) | 25.85 (21.97 - 31.62) | 24098.49 (21223.27 - 28036.21) | 29.79 (25.35 - 36.52) | 0.41 (0.32 - 0.51) | 139704.3 (124089.81 - 158000.91) | 429.18 (372.1 - 511.2) | 440924.93 (392014.24 - 506124.36) | 495.38 (430.32 - 591.45) | 0.42 (0.34 - 0.5) |
| Central Sub-Saharan Africa | 1166.56 (981.75- 1390.91) | 42.56 (31.23- 55.39) | 3053.02 (2586.06- 3602.95) | 45.21 (33.1- 59.97) | 0.24 (0.15- 0.33) | 7724.82 (6151.9 - 9643.31) | 273.84 (199.07 - 367.15) | 21875.94 (17531.73 - 27229.44) | 322.88 (237.54 - 428.23) | 0.54 (0.41 - 0.67) | 522.8 (435.25 - 623.04) | 26.06 (20.37 - 33.4) | 1304.41 (970.31 - 1649.82) | 25.73 (18.15 - 34.5) | -0.02 (-0.15 - 0.12) | 10809.67 (9113.89 - 12787.28) | 433.69 (346.4 - 541.78) | 25864.76 (19835.24 - 31794.06) | 429.99 (319.41 - 555.97) | 0 (-0.13 - 0.13) |
| Eastern Sub-Saharan Africa | 4118.13 (3574.89- 4690.91) | 42.52 (31.86- 54.66) | 10208.94 (8916.35- 11549.27) | 46.87 (35.4- 59.62) | 0.32 (0.29- 0.34) | 25730.34 (21407.48 - 30670.41) | 261.13 (197.85 - 334.75) | 68427.54 (57903.3 - 79193.22) | 313.31 (243.5 - 392.08) | 0.55 (0.51 - 0.6) | 1866.95 (1514.14 - 2189.93) | 23.62 (18.05 - 29.8) | 4268.86 (3204.81 - 5684.41) | 23.77 (17.29 - 32.48) | -0.06 (-0.09 - -0.02) | 36498.89 (30497.13 - 42107.51) | 396.1 (315.69 - 483.78) | 79807.71 (62454.82 - 101467.05) | 392.88 (299.97 - 513.93) | -0.12 (-0.15 - -0.08) |
| Southern Sub-Saharan Africa | 1683.15 (1442.23- 1933.49) | 44.43 (31.48- 58.99) | 4254.11 (3620.24- 4873.63) | 53.43 (38.36- 70.49) | 0.58 (0.54- 0.62) | 11330.58 (9500.31 - 13501.8) | 295.84 (223.4 - 383.55) | 29120.59 (24522.37 - 34339.61) | 366.65 (280.32 - 470.49) | 0.63 (0.56 - 0.7) | 650.04 (547.9 - 771.99) | 19.46 (16.04 - 24.1) | 1751.06 (1588.46 - 1871.33) | 25.78 (22.01 - 28.7) | 1.02 (0.71 - 1.33) | 11298.44 (9731.62 - 13112.2) | 311.34 (262.03 - 375.84) | 31016.75 (28262.16 - 33487.64) | 411.47 (358.09 - 458.5) | 0.97 (0.69 - 1.25) |
| Western Sub-Saharan Africa | 6495.48 (5707.23- 7357.89) | 54.95 (42.16- 69.17) | 16207.37 (14385.57- 18071.92) | 65.57 (51.77- 81.04) | 0.6 (0.56- 0.65) | 38627.31 (32362.53 - 45089.47) | 326.83 (252 - 412.32) | 100133.64 (85803.01 - 116581.39) | 405.67 (321.78 - 504.23) | 0.73 (0.65 - 0.8) | 2828.35 (2474.18 - 3189.66) | 28.98 (24.49 - 34.11) | 6690.92 (5703.63 - 7554.81) | 33.01 (26.92 - 37.56) | 0.61 (0.53 - 0.69) | 49609.82 (43925.95 - 55763.31) | 451.16 (386.71 - 523.62) | 115341.96 (97521.49 - 130535.35) | 508.18 (419.67 - 576.36) | 0.54 (0.47 - 0.61) |

**Abbreviations**: UI, uncertainty interval; ASR, age-standerised rate per 100,000; CI, confidence interval; DALYs, disability-adjusted life-year; EAPC, estimated annual percentage change.

**Table S3 Global burden of Alzheimer’s and other dementias among adults aged≥55 years from 1990 to 2021, categorized by sex, GDB regions**

| **Characteristics** | **Incidence** | | | | | **Prevalence** | | | | | **Deaths** | | | | | **DALYs** | | | | |
| --- | --- | --- | --- | --- | --- | --- | --- | --- | --- | --- | --- | --- | --- | --- | --- | --- | --- | --- | --- | --- |
|  | **No.(95% UI) in 1990** | **ASR per 100000 (95% UI) in 1990** | **No.(95% UI) in 2021** | **ASR per 100000 (95% UI) in 2021** | **EAPC**  **(95% CI)** | **No.(95% UI) in 1990** | **ASR per 100000 (95% UI) in 1990** | **No.(95% UI) in 2021** | **ASR per 100000 (95% UI) in 2021** | **EAPC**  **(95% CI)** | **No.(95% UI) in 1990** | **ASR per 100000 (95% UI) in 1990** | **No.(95% UI) in 2021** | **ASR per 100000 (95% UI) in 2021** | **EAPC**  **(95% CI)** | **No.(95% UI) in 1990** | **ASR per 100000 (95% UI) in 1990** | **No.(95% UI) in 2021** | **ASR per 100000 (95% UI) in 2021** | **EAPC**  **(95% CI)** |
| **Global** | 3621428.16 (3171580.6-4129746.33) | 662.94 (462- 893.46) | 9405626.27 (8205422.32- 10737459.29) | 679.44 (466.33- 922.85) | -0.02 (-0.04 - 0) | 20836524.35 (18188030.68- 23910226.9) | 3844.72 (3056.1- 4775.79) | 54905286.05 (47641202.11- 62959589.68) | 3975.78 (3131.76- 4965.09) | 0.01(-0.02- 0.03) | 659064.12 (162705.94- 1758754.97) | 147.57 (35.52- 400.47) | 1943723.27 (510007.64- 4971572.18) | 148.24 (37- 392.52) | -0.02(-0.03-  -0.01) | 13230588.43 (6226659.81- 29014297.04) | 2590.19 (1168.15- 5788.85) | 35625499.28 (16835214.41- 75695645.76) | 2621.1 (1192.8- 5766.16) | -0.02 (-0.03 - 0) |
| **Sex** |  |  |  |  |  |  |  |  |  |  |  |  |  |  |  |  |  |  |  |  |
| Female | 2367855.58 (2072171.23- 2694312.07) | 724.46 (507.03 - 973.94) | 5958085.27 (5207752.43- 6770078.26) | 751.18 (517.67- 1019.3) | 0 (-0.02 - 0.03) | 13628802.38 (11891048.71 - 15594159.87) | 4211.65 (3356.96 - 5220.2) | 35049359.2 (30535628.87 - 40070485) | 4414.52 (3492.78 - 5504.77) | 13628802.38 (11891048.71 - 15594159.87) | 460676.49 (114752.34 - 1212217.09) | 162.66 (39.6 - 435.46) | 1321047.42 (355375.39 - 3307426.95) | 164.29 (41.79 - 425.37) | -0.01 (-0.02 - 0.01) | 8921824.53 (4224019.3- 19297105.01) | 2877.25 (1301.69 - 6340.99) | 23423137.1 (11164770.64 - 48982189.27) | 2935.7 (1349.85 - 6306.48) | 0 (-0.01 - 0.02) |
| Male | 1253572.58 (1086171.69- 1444890.63) | 569.18 (392.28 - 773.48) | 3447541 (2962858.27 - 3973380.2) | 585.06 (398.91 - 798.96) | 0.02 (0 - 0.04) | 7207721.96 (6203504.33 - 8272714.84) | 3259.58 (2566.96 - 4074.95) | 19855926.85 (16953842.77 - 22912154.16) | 3367.78 (2626.54 - 4227.63) | 7207721.96 (6203504.33 - 8272714.84) | 198387.62 (47343.45 - 547554.38) | 118.84 (27.7 - 334.55) | 622675.85 (152981.67 - 1671763.27) | 121.94 (28.99 - 340.71) | 0.07 (0.05 - 0.09) | 4308763.9 (1998774.16 - 9717902.71) | 2105.1 (934.38 - 4894.86) | 12202362.18 (5664469.25 - 26635904.06) | 2161.17 (967.82 - 4977.54) | 0.05 (0.03 - 0.07) |
| **GBD Region** |  |  |  |  |  |  |  |  |  |  |  |  |  |  |  |  |  |  |  |  |
| Central Asia | 42361.9 (36518.6 - 48760.99) | 633.15 (432.35 - 866.57) | 65169.69 (56700.8 - 74326.91) | 621.99 (422.1 - 850.3) | -0.06 (-0.08 - -0.04) | 244722.09 (210769.41 - 278684.72) | 3651.05 (2860.43 - 4567.94) | 378314.23 (326872.98 - 433401.81) | 3584.41 (2804.31 - 4491.82) | -0.06 (-0.08 - -0.04) | 7208.77 (1778.88 - 19826) | 121.74 (28.72 - 337.24) | 10932.25 (2717.51 - 30307.33) | 118.21 (27.93 - 329.22) | -0.12 (-0.13 - -0.11) | 145286.79 (69360.95 - 312842.15) | 2256.32 (1045.45 - 5019.71) | 225177.7 (108446.32 - 487226.42) | 2200.77 (1019.22 - 4908.53) | -0.1 (-0.11 - -0.09) |
| Central Europe | 146071.74 (125799.01 - 170125.35) | 651.63 (443.82 - 893.96) | 262075.5 (225496.21 - 302204.34) | 636.4 (433.25 - 868.77) | -0.08 (-0.09 - -0.07) | 839210.63 (716848.95 - 967283.6) | 3759.93 (2933.85 - 4722.31) | 1506794.56 (1296532.9 - 1732087.5) | 3665.92 (2868.06 - 4603.31) | -0.09 (-0.1 - -0.08) | 22361.36 (5434.21 - 63186.41) | 122.24 (28.56 - 342.51) | 49733.55 (12482.31 - 132990.87) | 120.52 (28.72 - 332.65) | -0.04 (-0.05 - -0.03) | 478363.89 (230349.56 - 1060562.88) | 2273.97 (1052.77 - 5105.48) | 921527.72 (439799.91 - 1951607.58) | 2240.33 (1038.36 - 4910.24) | -0.05 (-0.06 - -0.04) |
| Eastern Europe | 277788.83 (239515.87 - 321512.27) | 664.39 (452.18 - 911.44) | 403010.11 (347586.69 - 464794.74) | 654.79 (446 - 897.08) | -0.08 (-0.12 - -0.04) | 1589811.13 (1364654.06 - 1841314.76) | 3824.33 (2976.17 - 4820.15) | 2320690.61 (1997386.32 - 2674227.08) | 3762.55 (2932.28 - 4741.67) | -0.08 (-0.13 - -0.04) | 42921.21 (10481.64 - 121799.94) | 126.66 (29.41 - 356.68) | 75073.02 (18208.54 - 208573.61) | 123.61 (29.25 - 341.96) | -0.09 (-0.1 - -0.08) | 912685.48 (440822.74 - 2004717.48) | 2344.12 (1086.82 - 5265.24) | 1418607.18 (661848.14 - 3073951.99) | 2301.92 (1068.24 - 5097.54) | -0.07 (-0.09 - -0.05) |
| Australasia | 27381.43 (23861.67 - 31215.41) | 703 (492.6 - 946.56) | 61628.7 (53776.41 - 69305.28) | 597.53 (436.4 - 783.57) | -0.57 (-0.61- -0.53) | 155471.9 (134439.25 - 178218.8) | 4041.92 (3194.53 - 4992.67) | 355281.12 (309624.79 - 400422.41) | 3448.66 (2780.7 - 4210.53) | -0.55 (-0.59 - -0.51) | 4986.2 (1226.29 - 13352.32) | 144.56 (34.95 - 388.02) | 15218.19 (4002.03 - 38516.69) | 136.61 (33.97 - 355.86) | -0.17 (-0.19 - -0.15) | 95571.44 (46088.81 - 201126.24) | 2577.32 (1198.03 - 5636.12) | 249388.84 (115882.43 - 518693.28) | 2353.7 (1069.28 - 5054.96) | -0.31 (-0.32 - -0.29) |
| High-income Asia Pacific | 201889.18 (174582.86 - 232594) | 662.25 (458.04 - 897.41) | 688720.45 (601316.75 - 787514.85) | 673.48 (462.48 - 914.06) | 0.2 (0.15 - 0.26) | 1133356.11 (979282.99 - 1298367.97) | 3757.04 (2957 - 4708.21) | 4047652.71 (3489081.33 - 4631816.46) | 3920.66 (3086.65 - 4900.63) | 0.3 (0.24 - 0.35) | 41951.88 (10669.41 - 109053.15) | 163.83 (40.92 - 431.56) | 199893.95 (57878.48 - 462582.02) | 156.77 (42.37 - 381.48) | -0.09 (-0.13 - -0.06) | 778451.33 (362758.55 - 1697729.3) | 2733.76 (1215.06 - 6032.63) | 3006938.83 (1426227.56 - 5979422.57) | 2683.52 (1257.15 - 5511.44) | 0.02 (-0.01 - 0.04) |
| High-income North America | 501390.2 (436461.82 - 573469.61) | 785.64 (545.99 - 1065.19) | 899694.11 (784209.84 - 1024307.98) | 738.59 (511.29 - 998.6) | -0.22 (-0.24 - -0.21) | 2934166.58 (2531740.36 - 3355653.11) | 4635.12 (3660.53 - 5795.45) | 5367579.1 (4664315.83 - 6135264.14) | 4400 (3468.05 - 5477.08) | -0.21 (-0.23 - -0.19) | 106349.74 (27577.04 - 275501.12) | 170.94 (42.5 - 450.97) | 216216.87 (58145.43 - 540353.61) | 166.49 (42.15 - 428.85) | -0.11 (-0.13 - -0.1) | 1903957.26 (898867.71 - 4066123.18) | 3028.22 (1399.21 - 6600.59) | 3619805.69 (1698082.41 - 7576901.2) | 2894.84 (1328 - 6249.9) | -0.18 (-0.2 - -0.16) |
| Southern Latin America | 44831.96 (38497.56 - 51392.84) | 633.32 (433.51 - 865.32) | 94964.71 (81552.67 - 109119.35) | 607.88 (412.29 - 830.71) | -0.13 (-0.15 - -0.12) | 249819.97 (214543.39 - 285657.8) | 3551.9 (2784.05 - 4439.53) | 530317.74 (454663.29 - 608306.96) | 3400.01 (2658.3 - 4272.46) | -0.14 (-0.16 - -0.13) | 7365.59 (1827.58 - 20238.12) | 121.42 (28.92 - 336.19) | 18726.98 (4794.01 - 48786.06) | 118.19 (28.88 - 315.54) | -0.06 (-0.07 - -0.04) | 147395.49 (71710.88 - 314983.56) | 2201.29 (1024.17 - 4890.09) | 334775.65 (159557.64 - 699628.17) | 2136.86 (990.99 - 4640.56) | -0.08 (-0.1 - -0.07) |
| Western Europe | 739967.19 (652785.44 - 829885.23) | 699.94 (511.11 - 914.85) | 1330679.19 (1161872.98 - 1519230.72) | 678.98 (478.98 - 908.4) | -0.14 (-0.18 - -0.1) | 4143154.17 (3612709.87 - 4698139.73) | 3969.33 (3228.53 - 4800.99) | 7635096.82 (6601066.89 - 8696375.07) | 3856.27 (3056.38 - 4766.26) | -0.11 (-0.14 - -0.08) | 152666.08 (38373.85 - 403242.59) | 157.91 (39.02 - 421.56) | 339096.67 (90827 - 835379.9) | 152.09 (39.08 - 387.48) | -0.1 (-0.12 - -0.09) | 2714994.31 (1273302.05 - 5788498.55) | 2681.52 (1226.78 - 5883.12) | 5346876.17 (2517379.44 - 10947048.18) | 2584.51 (1194.92 - 5482.16) | -0.12 (-0.13 - -0.1) |
| Andean Latin America | 13114.77 (11284.92 - 15054.95) | 453.64 (306.98 - 623.92) | 41957.45 (36111.96 - 48225.25) | 447.84 (305.92 - 617.37) | -0.06 (-0.07 - -0.05) | 74451.56 (64315.31 - 84902.85) | 2560.7 (2003.76 - 3209.69) | 236659.56 (204011.63 - 271461.07) | 2522.78 (1972.38 - 3163.43) | -0.06 (-0.08 - -0.05) | 2279.12 (549.61 - 6141.85) | 85.58 (19.63 - 239.48) | 7625.07 (1888.3 - 20524.82) | 82.83 (19.07 - 224.15) | -0.13 (-0.15 - -0.11) | 45758.61 (21384.15 - 99203.38) | 1609.9 (730.59 - 3634.55) | 146877.87 (70517.53 - 311915.34) | 1571.64 (727.62 - 3422.91) | -0.11 (-0.13 - -0.09) |
| Caribbean | 21765.42 (18780.86 - 25038.66) | 552.95 (382.07 - 754.47) | 50266.39 (43976.77 - 57271.51) | 541.92 (373.39 - 737.53) | -0.16 (-0.19 - -0.13) | 125435.99 (108614.41 - 143189.47) | 3187.18 (2514.13 - 3970.53) | 291362.83 (251524.65 - 331648.64) | 3149.17 (2478.55 - 3934.3) | -0.16 (-0.19 - -0.12) | 3215.28 (776.22 - 9076.42) | 96.27 (22.46 - 271.26) | 9023.06 (2240.39 - 24523.49) | 93.1 (21.67 - 258.51) | -0.1 (-0.11 - -0.09) | 69796.2 (34478.17 - 150770.07) | 1863.84 (882.15 - 4127.43) | 170681.63 (83380.68 - 361987.58) | 1817.14 (865.92 - 3940.4) | -0.13 (-0.14 - -0.11) |
| Central Latin America | 70120.58 (60399 - 80539.41) | 630.58 (429.29 - 862.28) | 236108.11 (205250.53 - 269435.08) | 600.91 (408.96 - 827.96) | -0.11 (-0.12 - -0.09) | 398945.84 (345542.36 - 457732.97) | 3579.76 (2811.36 - 4487.01) | 1340610.89 (1166461.57 - 1527487.43) | 3407.96 (2674.45 - 4278.76) | -0.11 (-0.13 - -0.1) | 9625.54 (2360.53 - 26660.19) | 101.02 (23.74 - 282.24) | 38507.05 (9525.13 - 101962.04) | 99.15 (23.75 - 269.89) | -0.07 (-0.08 - -0.06) | 213170.84 (105426.68 - 459477.48) | 1987.73 (953.53 - 4345.9) | 764313.2 (373148.2 - 1608560.19) | 1943.63 (932.8 - 4169.05) | -0.07 (-0.07 - -0.06) |
| Tropical Latin America | 86083.5 (74732.1 - 98163.36) | 727.85 (501.85 - 988.2) | 295720.3 (259119.19 - 337023.58) | 715.41 (490.48 - 981.26) | -0.11 (-0.15 - -0.08) | 511103.65 (441111.02 - 585831.32) | 4329.64 (3406.91 - 5411.97) | 1792058.58 (1552955.8 - 2048693.7) | 4340.51 (3412.19 - 5440.52) | -0.05 (-0.1 - 0) | 15793.2 (3918.54 - 41549.31) | 165.15 (40.67 - 443.82) | 64913.18 (17046.38 - 163820.56) | 160.55 (40.87 - 417.23) | -0.06 (-0.08 - -0.05) | 331377.95 (154169.09 - 737842.03) | 2964.78 (1319.5 - 6677.21) | 1201314.69 (560654.23 - 2570250.92) | 2921.78 (1325.83 - 6398.39) | -0.06 (-0.07 - -0.04) |
| North Africa and Middle East | 158873.61 (138439.43 - 181050.2) | 785.4 (541.68 - 1064.83) | 436122.95 (380249.54 - 499278.44) | 752.12 (515.99 - 1023.42) | -0.14 (-0.15 - -0.13) | 927764.97 (801635.91 - 1056678.39) | 4668.65 (3683.44 - 5833.9) | 2545939.75 (2209443.53 - 2908614.27) | 4438.63 (3481.46 - 5540.43) | -0.17 (-0.17 - -0.16) | 26167.96 (6360.74 - 70206.45) | 165.17 (39.12 - 455.14) | 73205.52 (18011.23 - 189660.51) | 150.94 (36.15 - 409.58) | -0.3 (-0.35 - -0.26) | 561493.74 (270506.84 - 1235565.69) | 3002.28 (1364.75 - 6730.03) | 1517932.38 (728346.07 - 3237716.13) | 2771.37 (1273.93 - 6113.84) | -0.28 (-0.3 - -0.25) |
| South Asia | 287739.57 (247779.19 - 331641.92) | 455.73 (309.41 - 627.27) | 861226.59 (738766.19 - 993966) | 446.19 (301.29 - 614.91) | -0.18 (-0.22 - -0.14) | 1635799.3 (1403680.8 - 1874959.25) | 2545.66 (1989.56 - 3215.8) | 4859475.8 (4158987.48 - 5594588.23) | 2488.75 (1937.51 - 3158.96) | -0.19 (-0.23 - -0.15) | 41229.34 (9499.94 - 117981.69) | 83.03 (18.29 - 244.08) | 163874.29 (39064.44 - 449441.13) | 101.23 (23.44 - 283.78) | 0.7 (0.66 - 0.75) | 965121.21 (442533.51 - 2258073.8) | 1574.69 (707.58 - 3701.21) | 3339100.26 (1467340.45 - 7512873.03) | 1786.26 (763.16 - 4204.34) | 0.41 (0.38 - 0.44) |
| East Asia | 670325.7 (572678.64 - 778925.19) | 679.26 (460.99 - 930.26) | 2856983.31 (2449578.63 - 3287415.85) | 850.81 (584.72 - 1162.65) | 0.41 (0.34 - 0.49) | 3904744.13 (3339564.69 - 4505157.71) | 3980.22 (3097.07 - 5020.04) | 16827718.17 (14239684.59 - 19546628.15) | 5106.19 (3979.84 - 6412.27) | 0.44 (0.36 - 0.53) | 122178.77 (28932.4 - 327890.96) | 181.88 (42.64 - 495.16) | 504860.84 (128475 - 1358064.22) | 179.2 (43.6 - 486.32) | -0.19 (-0.23 - -0.14) | 2687711.87 (1219661.94 - 6063055.6) | 3064.86 (1312.58 - 7001.98) | 10141787.57 (4956646.14 - 22420570.31) | 3230.29 (1485.14 - 7096.23) | -0.02 (-0.06 - 0.02) |
| Oceania | 1845.24 (1559.5 - 2155.42) | 663.29 (450.9 - 914.17) | 4861.77 (4152.75 - 5620.05) | 634.09 (429.77 - 870.93) | -0.19 (-0.21 - -0.16) | 10767.92 (9113.06 - 12500.76) | 3875.25 (3016.92 - 4871.25) | 28331.35 (24150.56 - 32668.6) | 3691.19 (2882.65 - 4643.46) | -0.2 (-0.22 - -0.18) | 251.25 (58.45 - 716.29) | 134.97 (30.6 - 384.59) | 721.34 (170.54 - 2012.46) | 124.02 (28.98 - 350.33) | -0.32 (-0.35 - -0.3) | 6333.86 (3022.25 - 13590.9) | 2479.13 (1115.18 - 5641.64) | 16702.73 (7908.06 - 36749.66) | 2304.85 (1046.27 - 5177.78) | -0.29 (-0.31 - -0.27) |
| Southeast Asia | 197650.85 (170723.73 - 226348.49) | 645.34 (439.95 - 884.1) | 533192.88 (464521.95 - 612672.12) | 618.4 (420.76 - 848.55) | -0.14 (-0.15 - -0.12) | 1188442.16 (1025389.39 - 1361491.52) | 3846.07 (3013.86 - 4816.99) | 3192320.86 (2749717.88 - 3657587.15) | 3670.31 (2863.82 - 4616.83) | -0.14 (-0.16 - -0.12) | 29722.24 (7061.39 - 79631.54) | 121.63 (27.69 - 341.27) | 97451.54 (24019.71 - 257958.84) | 133.29 (31.71 - 363.03) | 0.24 (0.2 - 0.29) | 674993.63 (327797.01 - 1439202.91) | 2315.73 (1065.39 - 5152.2) | 2014034.41 (932959.42 - 4242729.04) | 2424.37 (1089.03 - 5465.59) | 0.12 (0.08 - 0.15) |
| Central Sub-Saharan Africa | 15831.56 (13543.21 - 18313.11) | 718.91 (493.8 - 983.88) | 39495.29 (34420.62 - 45033.9) | 715.38 (492.85 - 969.67) | -0.01 (-0.04 - 0.01) | 92748.63 (79080.16 - 106445.57) | 4310.06 (3381.68 - 5394.79) | 233338.6 (200688.31 - 265311.9) | 4299.64 (3403.52 - 5344.19) | 0 (-0.02 - 0.03) | 2474.78 (588.48 - 6778.4) | 179.11 (41.42 - 496.1) | 7996.98 (1907.97 - 21984.18) | 205.65 (48.15 - 566.13) | 0.51 (0.45 - 0.57) | 60033.91 (26727.12 - 136229.39) | 3115.65 (1327.98 - 7242.49) | 170426.14 (73915.83 - 394170.77) | 3443.11 (1423.61 - 8141.58) | 0.37 (0.33 - 0.4) |
| Eastern Sub-Saharan Africa | 48349.46 (41348.79 - 55599.47) | 604.14 (414.59 - 825.43) | 108838.3 (94225.93 - 124514.91) | 577.2 (397.33 - 784.2) | -0.13 (-0.14 - -0.12) | 284515.33 (243550.87 - 325650.93) | 3526.59 (2765.03 - 4407.89) | 638473.77 (551176.9 - 727884.76) | 3353.51 (2648.68 - 4184.51) | -0.15 (-0.15 - -0.14) | 8036.57 (1954.56 - 22312.4) | 142.29 (32.82 - 398.69) | 23081.83 (5540.45 - 63917.39) | 160.14 (37.97 - 434.33) | 0.41 (0.4 - 0.42) | 183737.89 (84312.9 - 415387.44) | 2500.05 (1086.59 - 5798.58) | 467896.07 (207317.43 - 1048782.64) | 2676.26 (1115.92 - 6231.84) | 0.24 (0.23 - 0.25) |
| Southern Sub-Saharan Africa | 22559.34 (19473.27 - 25699.21) | 637.17 (434.43 - 873.26) | 43872.64 (37819.27 - 50297.64) | 604.48 (411.58 - 830.75) | -0.14 (-0.15 - -0.13) | 129695.28 (111267.05 - 148833.71) | 3647.65 (2849.43 - 4606.31) | 252537.7 (216067.65 - 290021.23) | 3452.75 (2692.7 - 4359.42) | -0.15 (-0.16 - -0.14) | 3852.16 (946.57 - 10508.38) | 127.29 (29.49 - 352.22) | 7787.48 (1878.25 - 21719.25) | 133.71 (31.29 - 374.66) | 0.14 (0.05 - 0.22) | 79420.71 (37460 - 173121.3) | 2331.03 (1060.47 - 5203.23) | 162746.56 (74337.9 - 367007.98) | 2371.49 (1044.46 - 5479.85) | 0.05 (-0.01 - 0.1) |
| Western Sub-Saharan Africa | 45486.13 (39063.02 - 52173.87) | 441.47 (298.43 - 608.17) | 91037.81 (78587.97 - 104226.61) | 411.17 (277.89 - 565.78) | -0.23 (-0.25 - -0.21) | 262397.02 (225740.13 - 299953.14) | 2481.28 (1934.18 - 3126.96) | 524731.29 (451192.5 - 599249.66) | 2301.74 (1791.73 - 2897.72) | -0.25 (-0.26 - -0.23) | 8427.07 (2050.46 - 23324.93) | 106.1 (24.3 - 301.31) | 19783.61 (4662.19 - 55603.72) | 113.36 (25.56 - 323.86) | 0.29 (0.24 - 0.33) | 174932.01 (78556.04 - 389973.6) | 1805.7 (766.51 - 4260.3) | 388587.98 (171269.85 - 910184.33) | 1858.43 (762.61 - 4479.99) | 0.14 (0.1 - 0.18) |

**Abbreviations**: UI, uncertainty interval; ASR, age-standerised rate per 100,000; CI, confidence interval; DALYs, disability-adjusted life-year; EAPC, estimated annual percentage change.

**Table S4 Global burden of late-onset multiple sclerosis, Parkinson’s disease, Alzheimer’s disease and other dementias among adults aged≥55 years from 1990 to 2021, categorized by SDI quintile**

| **Cause** | **Location** | **Incidence** | | | | | **Prevalence** | | | | | **Deaths** | | | | | **DALYs** | | | | |
| --- | --- | --- | --- | --- | --- | --- | --- | --- | --- | --- | --- | --- | --- | --- | --- | --- | --- | --- | --- | --- | --- |
|  |  | **No.(95% UI) in 1990** | **ASR per 100000 (95% UI) in 1990** | **No.(95% UI) in 2021** | **ASR per 100000 (95% UI) in 2021** | **EAPC**  **(95% CI)** | **No.(95% UI) in 1990** | **ASR per 100000 (95% UI) in 1990** | **No.(95% UI) in 2021** | **ASR per 100000 (95% UI) in 2021** | **EAPC**  **(95% CI)** | **No.(95% UI) in 1990** | **ASR per 100000 (95% UI) in 1990** | **No.(95% UI) in 2021** | **ASR per 100000 (95% UI) in 2021** | **EAPC**  **(95% CI)** | **No.(95% UI) in 1990** | **ASR per 100000 (95% UI) in 1990** | **No.(95% UI) in 2021** | **ASR per 100000 (95% UI) in 2021** | **EAPC**  **(95% CI)** |
| **LOMS** | Global | 2625.33 (2049.16 - 3179.98) | 0.39 (0.27 - 0.51) | 5035.5 (3914.89 - 6067.94) | 0.34 (0.23 - 0.45) | -0.41 (-0.45 - -0.37) | 343744.65 (298666 - 393824.9) | 51.52 (44.58 - 59.22) | 761107.58 (681925.57 - 849272.05) | 51.24 (45.73 - 57.21) | 0.18 (0.12 - 0.25) | 5054.36 (4824.16 - 5263.27) | 0.77 (0.72 - 0.81) | 11515.48 (10573.82 - 12081.66) | 0.78 (0.71 - 0.83) | 0.24 (0.16 - 0.32) | 207481 (181796.92 - 237118.03) | 30.54 (26.69 - 35) | 450155.06 (395614.81 - 508077.3) | 30.19 (26.44 - 34.23) | 0.16 (0.08 - 0.24) |
|  | High SDI | 816.38 (636.56 - 1006.07) | 0.45 (0.3 - 0.6) | 1228.46 (949.18 - 1536.34) | 0.37 (0.24 - 0.52) | -0.61 (-0.72 - -0.5) | 222394.95 (192933.72 - 252897.81) | 119.74 (103.97 - 136.78) | 457806.04 (416613.71 - 502735.05) | 135.88 (123.55 - 149.44) | 0.63 (0.56 - 0.7) | 3565.94 (3418.81 - 3671.94) | 1.9 (1.79 - 1.98) | 8492.63 (7708.2 - 8952.78) | 2.41 (2.2 - 2.57) | 1.03 (0.93 - 1.13) | 139982.23 (124143.8 - 158311.16) | 75.71 (66.98 - 85.82) | 301027.03 (267991.84 - 333457.28) | 89.56 (79.55 - 99.66) | 0.8 (0.71 - 0.89) |
|  | High-middle SDI | 697.39 (558.85 - 842.14) | 0.4 (0.29 - 0.52) | 1059.15 (836.48 - 1271.21) | 0.31 (0.22 - 0.4) | -0.86 (-0.91 - -0.81) | 75381.6 (65775.58 - 85408.57) | 43.42 (37.73 - 49.47) | 146397.32 (131583.95 - 162435.45) | 42.36 (37.94 - 47.13) | -0.08 (-0.16 - 0) | 1197.83 (1136.72 - 1266.37) | 0.72 (0.67 - 0.77) | 1795.29 (1659.67 - 1906.1) | 0.52 (0.47 - 0.56) | -1.22 (-1.41 - -1.03) | 48399.86 (42644.55 - 54975.38) | 27.62 (24.23 - 31.56) | 79007.75 (68753.19 - 90185.36) | 22.78 (19.61 - 26.18) | -0.74 (-0.84 - -0.64) |
|  | Middle SDI | 646.24 (498.48 - 788.33) | 0.37 (0.26 - 0.47) | 1661.51 (1316.75 - 2006.47) | 0.35 (0.25 - 0.45) | -0.14 (-0.15 - -0.12) | 25569.88 (21497.7 - 30006.14) | 14.4 (12.02 - 16.89) | 92572.93 (78074.58 - 107458.53) | 19.48 (16.42 - 22.68) | 1.18 (1.12 - 1.24) | 205.79 (164.31 - 238.43) | 0.14 (0.11 - 0.17) | 891.11 (816.56 - 972.17) | 0.2 (0.18 - 0.22) | 1.18 (0.88 - 1.47) | 11482.98 (9249.39 - 14412.4) | 6.6 (5.3 - 8.2) | 44781.49 (37681.09 - 53774.38) | 9.45 (7.87 - 11.34) | 1.24 (1.11 - 1.37) |
|  | Low-middle SDI | 345.71 (253.23 - 428.67) | 0.34 (0.22 - 0.46) | 834.23 (624.58 - 1031.91) | 0.35 (0.23 - 0.46) | 0.07 (0.05 - 0.09) | 15228.17 (12232.93 - 18406.59) | 14.58 (11.65 - 17.76) | 51566.39 (42573.56 - 60870.43) | 20.88 (17.25 - 24.84) | 1.24 (1.18 - 1.31) | 57.15 (34.64 - 78.42) | 0.07 (0.04 - 0.09) | 264.2 (201.44 - 322.96) | 0.12 (0.09 - 0.15) | 1.91 (1.77 - 2.06) | 5490.11 (4088.27 - 7413.92) | 5.33 (3.9 - 7.23) | 20053.56 (15642.43 - 25709.5) | 8.15 (6.3 - 10.54) | 1.46 (1.44 - 1.49) |
|  | Low SDI | 116.1 (82.67 - 147.77) | 0.31 (0.19 - 0.43) | 247.96 (177.52 - 313.43) | 0.3 (0.18 - 0.42) | -0.07 (-0.08 - -0.06) | 4734.88 (3771.55 - 5785.97) | 11.98 (9.41 - 14.81) | 12016.47 (9699.42 - 14474.45) | 14.05 (11.28 - 17.05) | 0.52 (0.49 - 0.55) | 14.58 (4.09 - 25.12) | 0.04 (0.01 - 0.08) | 55.18 (24.2 - 81.66) | 0.07 (0.03 - 0.11) | 1.77 (1.71 - 1.82) | 1682.4 (1136.08 - 2347.65) | 4.26 (2.83 - 6.08) | 4689.92 (3295.64 - 6436.69) | 5.49 (3.82 - 7.56) | 0.81 (0.8 - 0.83) |
| **PD** | Global | 371666.07 (324814.23 - 419673.09) | 60.57 (45.09 - 77.62) | 1184055.27 (1039201.25 - 1333546.01) | 82.17 (60.36 - 106.06) | 1.03 (0.99 - 1.06) | 2825059.24 (2441505.24 - 3270986.85) | 467.82 (377.06 - 574.41) | 10757500.76 (9480906.72 - 12341387.84) | 751.69 (614.51 - 914.06) | 1.53 (1.5 - 1.55) | 145055.51 (133004.91 - 155103.22) | 26.92 (24.12 - 29.23) | 382945.97 (342283.22 - 411719.04) | 28.08 (24.67 - 30.71) | 0.19 (0.14 - 0.25) | 2676445.49 (2481700.75 - 2877899.87) | 458.3 (415.68 - 501.64) | 7095613.7 (6403407.26 - 7732755.15) | 504.5 (446.61 - 557.27) | 0.33 (0.29 - 0.37) |
|  | High SDI | 120787.47 (107549.12 - 134769.48) | 61.7 (47.23 - 77.46) | 311258.41 (286523.53 - 334601.83) | 81.41 (66.28 - 97.76) | 0.93 (0.89 - 0.96) | 976347.18 (860902.09 - 1106363.35) | 499.89 (413.59 - 599.88) | 2712170.37 (2497171.99 - 2949657.12) | 692.82 (606.13 - 790.21) | 1.08 (1.02 - 1.15) | 47654.66 (43364.41 - 49658.8) | 24.57 (22.18 - 25.75) | 118614.7 (101595.26 - 127385.96) | 27.8 (23.87 - 29.84) | 0.59 (0.49 - 0.68) | 826555.62 (767112.02 - 876165.56) | 420.76 (383.09 - 452.62) | 1965815.05 (1758078.64 - 2119892.86) | 485.61 (427.91 - 530.03) | 0.61 (0.52 - 0.69) |
|  | High-middle SDI | 109925.11 (96068.06 - 124305.43) | 68.68 (51.53 - 87.68) | 325650.85 (279143.42 - 375531.23) | 95.44 (66.64 - 127.52) | 1.07 (1.02 - 1.12) | 877324.26 (761077.46 - 1016765.01) | 565.7 (459.88 - 692.19) | 3192074.46 (2759502.71 - 3715960.97) | 940.38 (752.75 - 1164.19) | 1.57 (1.53 - 1.6) | 39978.96 (36916.52 - 42138.15) | 29.08 (26.26 - 31.16) | 92026.71 (81051.83 - 100851.94) | 28.12 (24.49 - 31.37) | -0.16 (-0.23 - -0.09) | 751755.16 (694359.48 - 803918.91) | 501.78 (455.3 - 544.36) | 1775989.34 (1583420.99 - 1972589.81) | 529.89 (462.21 - 598.89) | 0.11 (0.05 - 0.17) |
|  | Middle SDI | 82404.07 (69731.91 - 95880.95) | 58.18 (41.25 - 77.05) | 373080.55 (316091.31 - 434092.59) | 87.5 (59.64 - 118.53) | 1.43 (1.37 - 1.49) | 585467.46 (492036.59 - 700970.01) | 406.01 (311.71 - 521.86) | 3537894.69 (3024134.78 - 4176155.9) | 834.91 (656.33 - 1046.21) | 2.36 (2.31 - 2.41) | 33345.04 (30271.46 - 36476.49) | 28.79 (25 - 32.59) | 103238.69 (91879.98 - 114137.02) | 27.49 (23.88 - 31.29) | -0.19 (-0.25 - -0.13) | 639761.21 (580270.2 - 699824.44) | 479.98 (422.55 - 538.86) | 2072167.83 (1839983.35 - 2303966.16) | 510.23 (444.25 - 580.47) | 0.15 (0.1 - 0.19) |
|  | Low-middle SDI | 43779.38 (37786.35 - 50360.61) | 53.16 (38.9 - 68.92) | 135703.7 (117978.32 - 153663.69) | 63.29 (46.19 - 81.68) | 0.64 (0.59 - 0.68) | 292038.45 (244988.86 - 348107.49) | 345.35 (265.29 - 441.85) | 1043196.42 (887198.67 - 1232300.03) | 488.5 (384.29 - 609.34) | 1.22 (1.16 - 1.28) | 17551.79 (14841.86 - 20888.33) | 25.44 (20.79 - 30.97) | 52766.62 (46081.43 - 59335.44) | 28.58 (24.17 - 32.96) | 0.45 (0.37 - 0.53) | 332812.91 (287238.06 - 391241.79) | 426.44 (357.57 - 508.82) | 979812.97 (871489.25 - 1091333.96) | 485.23 (419.71 - 552.76) | 0.45 (0.4 - 0.5) |
|  | Low SDI | 14336.5 (12479.01 - 16381.65) | 48.9 (36.5 - 62.15) | 37494.83 (32711.08 - 42370.81) | 54.91 (41.74 - 69.41) | 0.42 (0.37 - 0.47) | 90650.98 (75519.52 - 108208.57) | 301.19 (229.82 - 383.66) | 265210.44 (225378.39 - 310580.65) | 388.24 (303.58 - 484.1) | 0.88 (0.8 - 0.96) | 6340.93 (5299.93 - 7689.61) | 26.89 (21.46 - 33.3) | 15927.26 (13356.34 - 18973.66) | 28.75 (22.87 - 34.81) | 0.41 (0.25 - 0.58) | 122294.02 (104497.36 - 146990.73) | 444.04 (365.2 - 538.82) | 295499.64 (249910.7 - 345390.53) | 470.11 (383.8 - 561.31) | 0.32 (0.2 - 0.45) |
| **AD and other dementias** | Global | 3621428.16 (3171580.62 - 4129746.33) | 662.94 (462 - 893.46) | 9405626.27 (8205422.32 - 10737459.29) | 679.44 (466.33 - 922.85) | -0.02 (-0.04 - 0) | 20836524.35 (18188030.68 - 23910226.9) | 3844.72 (3056.12 - 4775.79) | 54905286.05 (47641202.11 - 62959589.68) | 3975.78 (3131.76 - 4965.09) | 0.01 (-0.02 - 0.03) | 659064.12 (162705.94 - 1758754.97) | 147.57 (35.52 - 400.47) | 1943723.27 (510007.64 - 4971572.18) | 148.24 (37 - 392.52) | -0.02 (-0.03 - -0.01) | 13230588.43 (6226659.81 - 29014297.04) | 2590.19 (1168.15 - 5788.85) | 35625499.28 (16835214.41 - 75695645.76) | 2621.1 (1192.88 - 5766.16) | -0.02 (-0.03 - 0) |
|  | High SDI | 1386205.19 (1213984.95 - 1574103.81) | 721.69 (511.97 - 959.81) | 2880559.56 (2519674.64 - 3268741.94) | 695.38 (485.15 - 935.17) | -0.11 (-0.12 - -0.1) | 7863656.26 (6863089.94 - 8937981.33) | 4139.76 (3326.95 - 5094.28) | 16871817.32 (14716139.83 - 19164689.3) | 4056.36 (3222.64 - 5026.88) | -0.05 (-0.05 - -0.04) | 281002.28 (71100.62 - 737445.99) | 159.53 (39.5 - 424.89) | 717978.89 (196368.58 - 1759786.95) | 154.51 (39.91 - 393.7) | -0.12 (-0.13 - -0.11) | 5100630.94 (2400878.88 - 10944651.01) | 2762.09 (1267.37 - 6045.77) | 11616224.56 (5490618.1 - 23840861.74) | 2678.83 (1243.05 - 5685.58) | -0.11 (-0.12 - -0.1) |
|  | High-middle SDI | 938967.85 (812373.95 - 1081434.16) | 670.02 (463.03 - 909.15) | 2483945.08 (2156089.56 - 2845539.33) | 751.7 (513.68 - 1025.46) | 0.22 (0.18 - 0.26) | 5442963.81 (4711008.99 - 6277294.16) | 3916.54 (3094.09 - 4881.07) | 14480533.5 (12432351.33 - 16706108.21) | 4393.63 (3439 - 5514.2) | 0.22 (0.18 - 0.26) | 169644.42 (41496.89 - 460883.08) | 152.69 (36.48 - 417.52) | 488133.01 (127915.48 - 1260697.84) | 155.71 (38.15 - 420.95) | 0.02 (-0.01 - 0.04) | 3465298.91 (1616747.75 - 7644672.44) | 2677.19 (1196.56 - 6034.92) | 9085513.25 (4311721.49 - 19373328.71) | 2800.58 (1286.23 - 6161.8) | 0.07 (0.04 - 0.09) |
|  | Middle SDI | 778373.97 (671805.42 - 895335.94) | 639.61 (435.62 - 874.14) | 2744827.26 (2391406.45 - 3148636.02) | 701.84 (479.57 - 961.29) | 0.1 (0.05 - 0.15) | 4540460.79 (3913128 - 5213170.44) | 3718.35 (2911.76 - 4670.53) | 16101011.37 (13837502.64 - 18558743.35) | 4145.89 (3245.57 - 5208.98) | 0.13 (0.08 - 0.18) | 129511.34 (31052.13 - 345919.99) | 139.51 (32.45 - 386.13) | 490156.43 (122072.42 - 1273913.77) | 144.69 (35.22 - 390.96) | 0.03 (0 - 0.05) | 2869857.1 (1338016.51 - 6411888.52) | 2520.65 (1120.42 - 5733.41) | 9884977.88 (4753496.2 - 21387320.83) | 2645.21 (1207.99 - 5862.83) | 0.03 (0 - 0.06) |
|  | Low-middle SDI | 382322.87 (331107.61 - 437862.3) | 540.61 (368.88 - 738.17) | 989968.19 (855709.41 - 1133618.3) | 523.25 (355.73 - 715.32) | -0.17 (-0.19 - -0.15) | 2203973.44 (1907011.75 - 2521361.6) | 3098.58 (2430.88 - 3887.76) | 5683826.73 (4921745.12 - 6511802.62) | 2992.19 (2345.49 - 3763.73) | -0.18 (-0.2 - -0.16) | 57764.19 (13730.66 - 158597.6) | 104.33 (24 - 297.42) | 187922.62 (45555.75 - 507759.03) | 117.77 (27.85 - 327.01) | 0.43 (0.41 - 0.45) | 1305436.88 (608668.49 - 2923199.56) | 1941.94 (878.74 - 4467.94) | 3800505.27 (1696468.39 - 8381682.22) | 2089.61 (912.95 - 4816.92) | 0.23 (0.22 - 0.24) |
|  | Low SDI | 131336.92 (113075.33 - 151560.1) | 537 (365.73 - 734.84) | 298154.99 (257532.71 - 342738.26) | 512.69 (349 - 698.09) | -0.19 (-0.2 - -0.18) | 761192.11 (651897.06 - 870992.1) | 3078.36 (2405.74 - 3859.73) | 1720808.15 (1483920.47 - 1962027.6) | 2930.09 (2300.71 - 3672.62) | -0.2 (-0.21 - -0.19) | 20467.78 (4914.78 - 56809.83) | 115.2 (26.16 - 330.1) | 57943.17 (13797.52 - 161568.87) | 129.99 (30.16 - 364.6) | 0.45 (0.36 - 0.53) | 475244.46 (217153.52 - 1067876.87) | 2078.4 (905.74 - 4867.73) | 1208919.44 (533169.66 - 2770654.84) | 2222.14 (936.36 - 5274.99) | 0.24 (0.18 - 0.29) |

**Abbreviations**: UI, uncertainty interval; ASR, age-standerised rate per 100,000; CI, confidence interval; DALYs, disability-adjusted life-year; EAPC, estimated annual percentage change.

**Table S5** **YLDs for Global Burden of LOMS, PD, AD and Other Dementias among Adults Aged ≥55 Years, 1990 to 2021**

| **Characteristics** | **LOMS** | | | | | **PD** | | | | | **AD and Other Dementias** | | | | |
| --- | --- | --- | --- | --- | --- | --- | --- | --- | --- | --- | --- | --- | --- | --- | --- |
|  | **No.(95% UI) in 1990** | **ASR per 100000 (95% UI) in 1990** | **No.(95% UI) in 2021** | **ASR per 100000 (95% UI) in 2021** | **EAPC**  **(95% CI)** | **No.(95% UI) in 1990** | **ASR per 100000 (95% UI) in 1990** | **No.(95% UI) in 2021** | **ASR per 100000 (95% UI) in 2021** | **EAPC**  **(95% CI)** | **No.(95% UI) in 1990** | **ASR per 100000 (95% UI) in 1990** | **No.(95% UI) in 2021** | **ASR per 100000 (95% UI) in 2021** | **EAPC**  **(95% CI)** |
| **Global** | 85106.21 (60636.94 - 113282.76) | 12.7 (8.99 - 17) | 187477.31 (132782.58 - 245110.33) | 12.6 (8.95 - 16.56) | 0.17 (0.11 - 0.23) | 397779.79 (279021.45 - 530330.44) | 65.39 (44.79 - 89.95) | 1510619.81 (1070042.04 - 2001830.98) | 105.24 (72.34 - 143.48) | 1.53 (1.51 - 1.56) | 4234184.25 (2910696.3 - 5630065.04) | 794.16 (528.34 - 1106.01) | 11226142.53 (7759387.21 - 14881037.73) | 816.08 (540.4 - 1141.89) | -0.01 (-0.03 - 0.01) |
| **Sex** |  |  |  |  |  |  |  |  |  |  |  |  |  |  |  |
| Female | 58451.59 (41515.51 - 77864.2) | 16.24 (11.45 - 21.68) | 127216.85 (91377.9 - 166385.54) | 16.19 (11.52 - 21.24) | 0.2 (0.13 - 0.27) | 203465.52 (142521.9 - 271123.43) | 58.49 (40.05 - 80.02) | 683050.29 (486794.62 - 905015.6) | 86.34 (59.51 - 117.98) | 1.25 (1.22 - 1.27) | 2862036.76 (1960950.24 - 3827050.76) | 893.45 (588.54 - 1257.33) | 7403661.99 (5079029.57 - 9843701.97) | 931.42 (610.87 - 1313.61) | 0.03 (0 - 0.05) |
| Male | 26654.61 (19255.95 - 35733.65) | 8.41  (5.92 - 11.36) | 60260.46 (41907.48 - 79719) | 8.55 (5.98 - 11.35) | 0.22 (0.16 - 0.27) | 194314.27 (136663.73 - 259774.85) | 75.41 (51.53 - 104.39) | 827569.52 (581071.01 - 1094495.8) | 128.72 (88.23 - 175.33) | 1.73 (1.7 - 1.76) | 1372147.5 (947200.49 - 1804353.13) | 635.31 (416.75 - 900.53) | 3822480.54 (2642931.37 - 5076895.27) | 655.79 (429.85 - 932.63) | 0.04 (0.02 - 0.06) |
| **Age** |  |  |  |  |  |  |  |  |  |  |  |  |  |  |  |
| 55–59 years | 23193.93 (16393.37 - 30790.15) | 12.52 (8.85 - 16.63) | 51023.02 (36082.6 - 66913.77) | 12.89 (9.12 - 16.91) | 0.32 (0.2 - 0.43) | 30320.22 (19262.87 - 43558.54) | 16.37 (10.4 - 23.52) | 126009.07 (83731.26 - 180690.17) | 31.84 (21.16 - 45.66) | 1.84 (1.69 - 1.99) | 199944.31 (124464.96 - 282708.24) | 107.96 (67.21 - 152.65) | 443030.33 (273166.13 - 631248.07) | 111.95 (69.03 - 159.52) | 0.09 (0.07 - 0.1) |
| 60–64 years | 20527.39 (14598.02 - 27548.75) | 12.78 (9.09 - 17.15) | 43316.91 (30666.89 - 56618.49) | 13.53 (9.58 - 17.69) | 0.35 (0.24 - 0.45) | 45398.91 (29654.16 - 64964.81) | 28.27 (18.46 - 40.45) | 156855.7 (102825.66 - 220141.09) | 49.01 (32.13 - 68.78) | 1.79 (1.68 - 1.91) | 319468.81 (205248.21 - 460272.94) | 198.91 (127.79 - 286.58) | 667958.72 (426335.49 - 958650.8) | 208.71 (133.21 - 299.53) | 0.15 (0.13 - 0.18) |
| 65–69 years | 16020.03 (11518.02 - 21395.43) | 12.96 (9.32 - 17.31) | 33870.64 (24133.99 - 44325.33) | 12.28 (8.75 - 16.07) | 0.16 (0.03 - 0.28) | 65248.35 (44319.08 - 88716.84) | 52.79 (35.85 - 71.77) | 243266.58 (164953.7 - 328109.87) | 88.19 (59.8 - 118.95) | 1.62 (1.58 - 1.65) | 442413.22 (291273.02 - 643410.45) | 357.91 (235.64 - 520.52) | 1063102.44 (694820.82 - 1549608.5) | 385.4 (251.89 - 561.77) | 0.14 (0.11 - 0.16) |
| 70–74 years | 10369.5 (7334.5 - 13843.2) | 12.25 (8.66 - 16.35) | 25447.12 (18092.91 - 33672.82) | 12.36 (8.79 - 16.36) | 0.08 (0 - 0.15) | 74981.63 (50666.62 - 105459.56) | 88.57 (59.85 - 124.57) | 283703.62 (192699.61 - 392271.33) | 137.83 (93.62 - 190.57) | 1.43 (1.4 - 1.45) | 573841.98 (376814.6 - 821626.62) | 677.81 (445.09 - 970.49) | 1486345.44 (976579.01 - 2130737.73) | 722.09 (474.44 - 1035.15) | 0.07 (0.04 - 0.1) |
| 75–79 years | 7777.1 (5405.24 - 10544.63) | 12.63 (8.78 - 17.13) | 15462.24 (11033.02 - 20597.16) | 11.72 (8.37 - 15.62) | -0.11 (-0.17 - -0.04) | 84133.98 (59853.15 - 112259.86) | 136.68 (97.23 - 182.37) | 270476.7 (190563.39 - 363069.44) | 205.09 (144.49 - 275.29) | 1.38 (1.35 - 1.41) | 798146.91 (543382.65 - 1110986.39) | 1296.63 (882.75 - 1804.85) | 1792578.21 (1222617.17 - 2498002.31) | 1359.2 (927.04 - 1894.08) | 0.02 (0 - 0.05) |
| 80–84 years | 4482.95 (3173.69 - 6004.06) | 12.67 (8.97 - 16.97) | 10172.5 (7304.05 - 13283.07) | 11.61 (8.34 - 15.17) | -0.18 (-0.29 - -0.07) | 61873.7 (42851.43 - 84367.78) | 174.9 (121.13 - 238.49) | 234538.94 (162459.13 - 314569.39) | 267.79 (185.49 - 359.17) | 1.42 (1.37 - 1.46) | 910100.14 (592631.62 - 1243943.9) | 2572.65 (1675.24 - 3516.36) | 2321558.41 (1499740.79 - 3210564.94) | 2650.69 (1712.36 - 3665.74) | -0.03 (-0.06 - 0) |
| 85–89 years | 1970.1 (1348.62 - 2678.97) | 13.04 (8.92 - 17.73) | 5338.93 (3759.3 - 7091.8) | 11.68 (8.22 - 15.51) | -0.08 (-0.23 - 0.06) | 27184.45 (18949.33 - 36829.44) | 179.9 (125.4 - 243.72) | 134021.04 (95707.03 - 178324.31) | 293.12 (209.32 - 390.02) | 1.56 (1.53 - 1.58) | 646955.24 (435749.38 - 893339.44) | 4281.33 (2883.64 - 5911.82) | 1969185.34 (1323169.82 - 2726479.07) | 4306.89 (2893.96 - 5963.19) | -0.08 (-0.11 - -0.05) |
| 90–94 years | 608.69 (424.35 - 840.64) | 14.2 (9.9 - 19.62) | 2180.11 (1562.81 - 2902.37) | 12.19 (8.74 - 16.22) | -0.08 (-0.22 - 0.05) | 7080.83 (5043.75 - 9572.67) | 165.24 (117.7 - 223.39) | 48793.12 (34805.64 - 66057.66) | 272.75 (194.56 - 369.26) | 1.65 (1.62 - 1.69) | 261756.96 (179075.06 - 355048.77) | 6108.41 (4178.93 - 8285.49) | 1068544.36 (722726.71 - 1459102.29) | 5973.07 (4039.98 - 8156.25) | -0.13 (-0.15 - -0.11) |
| 95+ years | 156.52 (107.57 - 212.61) | 15.37 (10.57 - 20.88) | 665.84 (477.05 - 889.9) | 12.22 (8.75 - 16.33) | -0.34 (-0.5 - -0.18) | 1557.73 (1058.29 - 2247.72) | 153.01 (103.95 - 220.78) | 12955.04 (9075.65 - 18025.49) | 237.69 (166.52 - 330.72) | 1.53 (1.47 - 1.59) | 81556.69 (56257.57 - 112130.02) | 8010.78 (5525.81 - 11013.8) | 413839.28 (285509.37 - 568057.55) | 7592.95 (5238.41 - 10422.48) | -0.2 (-0.22 - -0.18) |
| **SDI region** |  |  |  |  |  |  |  |  |  |  |  |  |  |  |  |
| High SDI | 54008.9 (38427.94 - 71499.85) | 29.12 (20.65 - 38.68) | 110131.82 (78451.68 - 142453.3) | 32.83 (23.26 - 42.65) | 0.61 (0.54 - 0.68) | 136943.3 (98509 - 180376.3) | 70.1 (48.48 - 95.72) | 375967.93 (275694.06 - 491540.47) | 96.61 (69.11 - 128.45) | 1.07 (1 - 1.13) | 1624509.47 (1117179.29 - 2159340.05) | 860.91 (576.03 - 1196.69) | 3497555.41 (2406923.52 - 4653659.57) | 833.09 (552.94 - 1160.25) | -0.09 (-0.1 - -0.08) |
| High-middle SDI | 18889.16 (13539.31 - 25085.56) | 10.85 (7.6 - 14.59) | 36358.15 (26555.37 - 47780.75) | 10.51 (7.49 - 13.86) | -0.1 (-0.18 - -0.03) | 123716.51 (86277.78 - 166008.88) | 79.2 (54.22 - 108.52) | 450899.39 (315007.06 - 598637.79) | 132.54 (89.37 - 182.28) | 1.59 (1.56 - 1.63) | 1108881.97 (759691.3 - 1480907.54) | 813.08 (536.28 - 1136.97) | 2983992.67 (2058877.67 - 3970146.82) | 908.35 (598.79 - 1276.87) | 0.21 (0.17 - 0.25) |
| Middle SDI | 6641.41 (4684.17 - 9143.91) | 3.73 (2.61 - 5.15) | 23811.92 (16832.62 - 32505.6) | 5 (3.5 - 6.83) | 1.13 (1.08 - 1.19) | 83201.16 (57626.27 - 112182.47) | 56.96 (38.1 - 80.45) | 500393.59 (349593.88 - 669291.01) | 117.18 (78.65 - 162.98) | 2.36 (2.3 - 2.41) | 912298.18 (622179.32 - 1216366.37) | 762.93 (501.91 - 1073) | 3264496.36 (2259464.76 - 4347191.76) | 849.33 (560.44 - 1193.46) | 0.13 (0.08 - 0.18) |
| Low-middle SDI | 4154.02 (2856.14 - 5811.17) | 3.97 (2.66 - 5.63) | 13696.64 (9455.56 - 19042.09) | 5.54 (3.74 - 7.71) | 1.16 (1.11 - 1.21) | 40792.42 (28263.4 - 55075.2) | 47.67 (31.79 - 67.96) | 145296.22 (102755.96 - 194891.24) | 67.46 (46.15 - 93.69) | 1.22 (1.17 - 1.28) | 434474.59 (301501.34 - 574465.97) | 622.71 (413.08 - 868.72) | 1129784.95 (781642.25 - 1488840.35) | 603.21 (399.89 - 844.72) | -0.16 (-0.17 - -0.14) |
| Low SDI | 1303.94 (884.93 - 1816.14) | 3.3 (2.18 - 4.68) | 3292.6 (2248.06 - 4609.13) | 3.84 (2.59 - 5.48) | 0.51 (0.48 - 0.53) | 12673.16 (8746.82 - 17099.06) | 41.44 (27.59 - 58.62) | 37091.31 (26066.47 - 49999.27) | 53.63 (36.51 - 74.9) | 0.9 (0.82 - 0.98) | 149094.3 (103175.75 - 197031.95) | 616.88 (408.47 - 860.3) | 340575.53 (236024.31 - 454115.5) | 591.62 (392.46 - 826.31) | -0.16 (-0.17 - -0.15) |

**Abbreviations**: LOMS, late-onset multiple sclerosis; PD, Parkinson’s disease; AD and other dementias, Alzheimer’s disease and other dementias; UI, uncertainty interval; ASR, age-standerised rate per 100,000; CI, confidence interval; YLDs: years lived with disability; EAPC, estimated annual percentage

**Table S6** **YLLs for Global Burden of LOMS, PD, AD and Other Dementias among Adults Aged ≥55 Years, 1990 to 2021**

| **Characteristics** | **LOMS** | | | | | **PD** | | | | | **AD and Other Dementias** | | | | |
| --- | --- | --- | --- | --- | --- | --- | --- | --- | --- | --- | --- | --- | --- | --- | --- |
|  | **No.(95% UI) in 1990** | **ASR per 100000 (95% UI) in 1990** | **No.(95% UI) in 2021** | **ASR per 100000 (95% UI) in 2021** | **EAPC**  **(95% CI)** | **No.(95% UI) in 1990** | **ASR per 100000 (95% UI) in 1990** | **No.(95% UI) in 2021** | **ASR per 100000 (95% UI) in 2021** | **EAPC**  **(95% CI)** | **No.(95% UI) in 1990** | **ASR per 100000 (95% UI) in 1990** | **No.(95% UI) in 2021** | **ASR per 100000 (95% UI) in 2021** | **EAPC**  **(95% CI)** |
| **Global** | 122374.79 (117213.77 - 126995.66) | 17.85 (16.88 - 18.7) | 262677.75 (246024.48 - 274068.92) | 17.59 (16.32 - 18.54) | 0.15 (0.06 - 0.25) | 2278665.7 (2103459.33 - 2430695.14) | 392.92 (353.91 - 426.39) | 5584993.89 (5040000.01 - 6006955.55) | 399.26 (355.17 - 436.41) | 0.08 (0.02 - 0.13) | 8996404.18 (2154830.65 - 23922954.42) | 1796.03 (426.79 - 4958.66) | 24399356.75 (6145754.73 - 62634135.27) | 1805.01 (443.55 - 4897.63) | -0.02 (-0.04 - -0.01) |
| **Sex** |  |  |  |  |  |  |  |  |  |  |  |  |  |  |  |
| Female | 74186.08 (71009.24 - 77374.51) | 20.39 (19.17 - 21.54) | 165737.96 (152215.62 - 175561.75) | 21.08 (19.2 - 22.59) | 0.32 (0.19 - 0.44) | 1050809.84 (944615.95 - 1147959.03) | 308.8 (265.46 - 351.06) | 2373209.19 (2044025.09 - 2622702.81) | 299.82 (250.16 - 344.66) | -0.1 (-0.14 - -0.07) | 6059787.77 (1477913.46 - 15772067.57) | 1983.8 (475.97 - 5404.34) | 16019475.11 (4149334.93 - 40491141.25) | 2004.28 (501.51 - 5319.96) | -0.01 (-0.02 - 0) |
| Male | 48188.72 (45939.44 - 50106.36) | 14.97 (14.07 - 15.77) | 96939.79 (91599.16 - 103070.89) | 13.71 (12.76 - 14.73) | -0.09 (-0.16 - -0.03) | 1227855.86 (1142931.58 - 1342204.99) | 523.69 (478.54 - 573.18) | 3211784.7 (2908978.24 - 3494718.14) | 534.72 (476.74 - 584.89) | 0.11 (0.04 - 0.19) | 2936616.4 (682246.57 - 8152384.86) | 1469.79 (339.86 - 4190.74) | 8379881.64 (2009074.17 - 22631643.44) | 1505.38 (354.02 - 4282.85) | 0.05 (0.03 - 0.07) |
| **Age** |  |  |  |  |  |  |  |  |  |  |  |  |  |  |  |
| 55–59 years | 36018.78 (34325.58 - 37554.63) | 19.45 (18.53 - 20.28) | 64343.11 (61498.58 - 67270.14) | 16.26 (15.54 - 17) | -0.36 (-0.62 - -0.09) | 92955.91 (82458.98 - 102000.41) | 50.19 (44.52 - 55.08) | 170679.35 (154425.07 - 188980.45) | 43.13 (39.02 - 47.76) | -0.59 (-0.65 - -0.53) | 317201.18 (58768.48 - 991661.59) | 171.27 (31.73 - 535.45) | 695531.61 (127661.28 - 2197930.42) | 175.76 (32.26 - 555.41) | 0.07 (0.05 - 0.09) |
| 60–64 years | 32117.51 (30744.98 - 33344.9) | 20 (19.14 - 20.76) | 62597.07 (59573.11 - 65144.35) | 19.56 (18.61 - 20.35) | 0.21 (0.05 - 0.37) | 156832.43 (139286.61 - 171779.99) | 97.65 (86.72 - 106.96) | 277139.29 (254221.18 - 303671.77) | 86.59 (79.43 - 94.88) | -0.44 (-0.48 - -0.4) | 617962.92 (124655.83 - 1915428.79) | 384.76 (77.61 - 1192.6) | 1260408.66 (256125.34 - 3927375.21) | 393.82 (80.03 - 1227.12) | 0.09 (0.07 - 0.1) |
| 65–69 years | 24812.42 (23536.42 - 26008.27) | 20.07 (19.04 - 21.04) | 53268.99 (49876.69 - 55901.82) | 19.31 (18.08 - 20.27) | 0.31 (0.14 - 0.48) | 264330.42 (237791.09 - 287487.27) | 213.84 (192.37 - 232.58) | 547307.47 (502735.63 - 597322.33) | 198.41 (182.25 - 216.54) | -0.3 (-0.39 - -0.22) | 901436.5 (202698.08 - 2598125.4) | 729.26 (163.98 - 2101.88) | 2063989.63 (471952.71 - 6138913.59) | 748.25 (171.1 - 2225.52) | 0.07 (0.04 - 0.09) |
| 70–74 years | 13637.53 (12976.04 - 14326.8) | 16.11 (15.33 - 16.92) | 39852.79 (36630.27 - 42074.57) | 19.36 (17.8 - 20.44) | 0.48 (0.3 - 0.66) | 418842.3 (381749.78 - 455006.26) | 494.73 (450.91 - 537.44) | 993628.19 (906375.83 - 1085914.97) | 482.72 (440.33 - 527.55) | -0.13 (-0.2 - -0.05) | 1066250.46 (258212.52 - 3000796.32) | 1259.43 (305 - 3544.48) | 2640403.45 (663983.86 - 7480552.07) | 1282.75 (322.57 - 3634.17) | 0.01 (-0.02 - 0.04) |
| 75–79 years | 9330.88 (8699.45 - 9878.03) | 15.16 (14.13 - 16.05) | 22673.03 (20378.47 - 24301.34) | 17.19 (15.45 - 18.43) | 0.43 (0.27 - 0.58) | 576970.59 (534608.4 - 622522.51) | 937.32 (868.5 - 1011.32) | 1248646.94 (1143299.29 - 1364997.46) | 946.77 (866.89 - 1035) | 0.03 (-0.02 - 0.09) | 1418082.09 (330345.46 - 4106284.12) | 2303.75 (536.66 - 6670.86) | 3072794.31 (736502.68 - 8892298.87) | 2329.91 (558.45 - 6742.49) | -0.05 (-0.07 - -0.03) |
| 80–84 years | 4321.62 (3892.08 - 4608.83) | 12.22 (11 - 13.03) | 12156.43 (10018.76 - 13270.06) | 13.88 (11.44 - 15.15) | 0.44 (0.32 - 0.56) | 467617.78 (423609.05 - 502311.57) | 1321.85 (1197.45 - 1419.92) | 1223400.03 (1078848.78 - 1323726.78) | 1396.85 (1231.8 - 1511.4) | 0.25 (0.19 - 0.31) | 2007260.21 (493487.01 - 5566356.06) | 5674.09 (1394.98 - 15734.87) | 4968711.73 (1273167.74 - 13183151.72) | 5673.14 (1453.67 - 15052.17) | -0.05 (-0.07 - -0.03) |
| 85–89 years | 1665.41 (1456.5 - 1807.41) | 11.02 (9.64 - 11.96) | 5449.19 (4381.23 - 6039.92) | 11.92 (9.58 - 13.21) | 0.46 (0.34 - 0.57) | 227487.49 (199469.93 - 247452.88) | 1505.44 (1320.02 - 1637.56) | 761571.54 (649477.18 - 834827.66) | 1665.66 (1420.5 - 1825.89) | 0.45 (0.37 - 0.54) | 1556398.01 (373942.11 - 4166085.85) | 10299.72 (2474.62 - 27569.76) | 4712603.43 (1182257.8 - 12368541.51) | 10307.13 (2585.76 - 27051.74) | -0.04 (-0.06 - -0.03) |
| 90–94 years | 417.19 (342.97 - 463.13) | 9.74 (8 - 10.81) | 1974 (1519.51 - 2215.57) | 11.03 (8.49 - 12.38) | 0.66 (0.55 - 0.78) | 61882.76 (52624.97 - 68680.97) | 1444.11 (1228.07 - 1602.75) | 291583.18 (237991.71 - 323526.44) | 1629.92 (1330.35 - 1808.48) | 0.55 (0.49 - 0.61) | 787199.76 (193644.67 - 2029290.73) | 18370.25 (4518.93 - 47355.94) | 3275448.93 (833349.13 - 8110851.92) | 18309.47 (4658.35 - 45338.94) | -0.01 (-0.03 - 0) |
| 95+ years | 53.45 (40.73 - 61.25) | 5.25 (4 - 6.02) | 363.14 (263.32 - 417.45) | 6.66 (4.83 - 7.66) | 1.04 (0.91 - 1.18) | 11746.03 (9248.8 - 13411.4) | 1153.74 (908.45 - 1317.31) | 71037.9 (53077.86 - 80903.42) | 1303.37 (973.85 - 1484.38) | 0.49 (0.41 - 0.57) | 324613.05 (79263.7 - 846012.44) | 31884.62 (7785.56 - 83098.29) | 1709465.01 (431470.88 - 4270668.18) | 31364.55 (7916.45 - 78356.45) | -0.08 (-0.13 - -0.04) |
| **SDI region** |  |  |  |  |  |  |  |  |  |  |  |  |  |  |  |
| High SDI | 85973.33 (83060.79 - 88175.72) | 46.59 (44.4 - 48.56) | 190895.21 (177253.79 - 200012.32) | 56.73 (52.43 - 60.13) | 0.91 (0.8 - 1.02) | 689612.32 (638493.52 - 713500.83) | 350.66 (322.4 - 365.09) | 1589847.13 (1398560.64 - 1691453.41) | 389 (342.08 - 413.79) | 0.5 (0.41 - 0.6) | 3476121.47 (864607.5 - 9067616.04) | 1901.17 (464.01 - 5166.14) | 8118669.15 (2154659.13 - 19991573.36) | 1845.74 (468.2 - 4829.32) | -0.12 (-0.13 - -0.11) |
| High-middle SDI | 29510.71 (28001.85 - 31212.06) | 16.77 (15.69 - 18) | 42649.6 (39805.28 - 45259.86) | 12.27 (11.21 - 13.24) | -1.21 (-1.38 - -1.05) | 628038.65 (582837.53 - 661674.69) | 422.58 (383.86 - 452.59) | 1325089.96 (1180271.23 - 1458515.38) | 397.35 (348.49 - 443.9) | -0.26 (-0.34 - -0.18) | 2356416.94 (558937.17 - 6335631.78) | 1864.11 (440.75 - 5161.07) | 6101520.58 (1545479.36 - 15974748.11) | 1892.23 (458.35 - 5223.04) | 0 (-0.02 - 0.02) |
| Middle SDI | 4841.57 (3834.93 - 5628.88) | 2.87 (2.24 - 3.4) | 20969.58 (19358.6 - 22841.68) | 4.45 (3.99 - 4.94) | 1.37 (1.07 - 1.67) | 556560.05 (504652.49 - 606898.87) | 423.02 (367.97 - 478.34) | 1571774.24 (1409600.49 - 1744909.05) | 393.06 (344.47 - 447.47) | -0.3 (-0.36 - -0.24) | 1957558.92 (447664 - 5348695.6) | 1757.71 (405.09 - 4923.92) | 6620481.51 (1620406.66 - 17765581.84) | 1795.88 (432.43 - 4953.76) | -0.02 (-0.04 - 0.01) |
| Low-middle SDI | 1336.09 (796.48 - 1849.52) | 1.36 (0.8 - 1.94) | 6356.92 (4844.33 - 7794.75) | 2.61 (1.92 - 3.35) | 2.19 (2.04 - 2.34) | 292020.49 (247587.37 - 348623.66) | 378.78 (311.56 - 460.08) | 834516.74 (728294.69 - 937682.11) | 417.76 (354.71 - 480.31) | 0.34 (0.28 - 0.4) | 870962.3 (201583.27 - 2456572.46) | 1319.23 (301.24 - 3817.76) | 2670720.32 (641265.12 - 7311396.78) | 1486.4 (347.45 - 4196.46) | 0.4 (0.38 - 0.42) |
| Low SDI | 378.46 (110.02 - 650.01) | 0.96 (0.25 - 1.78) | 1397.33 (626.21 - 2070.18) | 1.64 (0.67 - 2.64) | 1.66 (1.6 - 1.73) | 109620.86 (92860.19 - 133209.42) | 402.6 (325.11 - 496.79) | 258408.33 (215534.65 - 306543.93) | 416.48 (332.59 - 500.77) | 0.26 (0.13 - 0.39) | 326150.17 (76409.24 - 909260.12) | 1461.52 (330.39 - 4239.57) | 868343.91 (204177.23 - 2399861.78) | 1630.52 (374.35 - 4638.55) | 0.39 (0.32 - 0.46) |

**Abbreviations**: LOMS, late-onset multiple sclerosis; PD, Parkinson’s disease; AD and other dementias, Alzheimer’s disease and other dementias; UI, uncertainty interval; ASR, age-standerised rate per 100,000; CI, confidence interval; YLLs: years of life lost; EAPC, estimated annual percentage change.

**Table S7 Global burden of late-onset multiple sclerosis among adults aged≥55 years from 1990 to 2021, categorized by countries**

| **Countries** | **Incidence** | | | | | **Prevalence** | | | | | **Deaths** | | | | | **DALYs** | | | | |
| --- | --- | --- | --- | --- | --- | --- | --- | --- | --- | --- | --- | --- | --- | --- | --- | --- | --- | --- | --- | --- |
|  | **No.(95% UI) in 1990** | **ASR per 100000 (95% UI) in 1990** | **No.(95% UI) in 2021** | **ASR per 100000 (95% UI) in 2021** | **EAPC**  **(95% CI)** | **No.(95% UI) in 1990** | **ASR per 100000 (95% UI) in 1990** | **No.(95% UI) in 2021** | **ASR per 100000 (95% UI) in 2021** | **EAPC**  **(95% CI)** | **No.(95% UI) in 1990** | **ASR per 100000 (95% UI) in 1990** | **No.(95% UI) in 2021** | **ASR per 100000 (95% UI) in 2021** | **EAPC**  **(95% CI)** | **No.(95% UI) in 1990** | **ASR per 100000 (95% UI) in 1990** | **No.(95% UI) in 2021** | **ASR per 100000 (95% UI) in 2021** | **EAPC**  **(95% CI)** |
| Afghanistan | 5.25 (3.89 - 6.57) | 0.42 (0.28 - 0.56) | 5.56 (4.08 - 7.09) | 0.43 (0.28 - 0.57) | 0.06 (0.05 - 0.07) | 730.09 (608.17 - 868.76) | 58.16 (47.72 - 69.83) | 1171.91 (989.16 - 1373.63) | 94.03 (79.16 - 110.59) | 1.62 (1.53 - 1.7) | 2.88 (0.63 - 6.16) | 0.25 (0.04 - 0.68) | 7.46 (2.78 - 13.83) | 0.62 (0.16 - 1.49) | 2.92 (2.73 - 3.12) | 257.97 (166.72 - 391.94) | 20.5 (11.62 - 33.21) | 463.79 (306.77 - 648.99) | 37.01 (21.12 - 60.58) | 1.96 (1.93 - 1.99) |
| Albania | 8.47 (7.26 - 9.71) | 2.13 (1.76 - 2.5) | 6.29 (4.61 - 8.17) | 0.85 (0.52 - 1.16) | -3.15 (-3.3 - -2.99) | 1040.87 (949.58 - 1148.98) | 290.46 (264.03 - 321.07) | 1722.45 (1587.23 - 1857.05) | 217.37 (200.23 - 235.37) | -0.94 (-1.07 - -0.82) | 17.24 (11.34 - 24.27) | 5.42 (3.03 - 9) | 28.02 (14.64 - 48.7) | 3.63 (1.47 - 7.44) | -1.52 (-1.85 - -1.18) | 653.85 (500.07 - 844.78) | 186.73 (128.98 - 269.15) | 1034.86 (727.58 - 1510.48) | 131.47 (79.9 - 220.28) | -1.17 (-1.32 - -1.02) |
| Algeria | 8.7 (6.25 - 10.81) | 0.41 (0.26 - 0.55) | 26.25 (19.2 - 33.59) | 0.42 (0.27 - 0.57) | 0.12 (0.11 - 0.13) | 1460.58 (1226.51 - 1734.03) | 68.02 (57 - 80.91) | 6598.68 (5630.76 - 7720.82) | 107.29 (91.15 - 125.51) | 1.48 (1.46 - 1.51) | 4.36 (1.67 - 7.4) | 0.25 (0.08 - 0.57) | 33.74 (19.4 - 51.86) | 0.61 (0.24 - 1.26) | 2.89 (2.51 - 3.27) | 466.4 (330.16 - 648.38) | 22.07 (13.3 - 33.82) | 2426.43 (1843.42 - 3258.24) | 39.43 (25.39 - 58.35) | 1.9 (1.77 - 2.03) |
| American Samoa | 0.01 (0.01 - 0.01) | 0.28 (0.17 - 0.41) | 0.02 (0.02 - 0.03) | 0.27 (0.17 - 0.39) | -0.18 (-0.19 - -0.16) | 0.1 (0.07 - 0.13) | 2.5 (1.72 - 3.44) | 0.25 (0.19 - 0.32) | 2.76 (1.95 - 3.71) | 0.27 (0.22 - 0.31) | 0 (0 - 0) | 0 (0 - 0) | 0 (0 - 0) | 0 (0 - 0) | 1.53 (1.38 - 1.68) | 0.03 (0.02 - 0.04) | 0.71 (0.43 - 1.08) | 0.07 (0.05 - 0.1) | 0.78 (0.48 - 1.17) | 0.27 (0.23 - 0.31) |
| Andorra | 0.05 (0.03 - 0.06) | 0.47 (0.3 - 0.64) | 0.13 (0.09 - 0.17) | 0.48 (0.3 - 0.67) | 0.11 (0.09 - 0.12) | 11.44 (9.83 - 13.15) | 115.03 (98.66 - 132.29) | 42.15 (36.47 - 49.02) | 159.5 (138.05 - 185.69) | 1.11 (1.02 - 1.2) | 0.23 (0.13 - 0.39) | 2.25 (0.93 - 4.94) | 0.6 (0.37 - 0.9) | 2.29 (0.97 - 4.63) | 0.55 (0.32 - 0.77) | 8.55 (5.95 - 13.04) | 85.11 (49.95 - 154.15) | 24.67 (17.85 - 33.72) | 93.74 (57.82 - 151.72) | 0.64 (0.48 - 0.79) |
| Angola | 1.65 (1.12 - 2.22) | 0.26 (0.15 - 0.39) | 4.96 (3.36 - 6.87) | 0.25 (0.15 - 0.38) | -0.05 (-0.07 - -0.02) | 50.86 (39.39 - 64.18) | 7.34 (5.5 - 9.47) | 187.53 (146.78 - 231.29) | 8.87 (6.84 - 11.14) | 0.61 (0.54 - 0.68) | 0.28 (0.07 - 0.53) | 0.05 (0.01 - 0.12) | 1.74 (0.62 - 3.24) | 0.1 (0.03 - 0.23) | 2.13 (2.07 - 2.19) | 21.86 (13.44 - 32.08) | 3.2 (1.89 - 5.14) | 98.76 (61.2 - 142.01) | 4.74 (2.76 - 7.81) | 1.24 (1.18 - 1.29) |
| Antigua and Barbuda | 0.03 (0.02 - 0.04) | 0.37 (0.24 - 0.49) | 0.07 (0.05 - 0.09) | 0.37 (0.23 - 0.5) | -0.01 (-0.03 - 0.02) | 2.08 (1.73 - 2.45) | 24.81 (20.25 - 29.43) | 7.99 (7.02 - 9.01) | 41.41 (36.17 - 47.03) | 1.85 (1.62 - 2.08) | 0.05 (0.05 - 0.06) | 0.61 (0.46 - 0.79) | 0.18 (0.15 - 0.2) | 0.94 (0.71 - 1.22) | 1.55 (1.24 - 1.87) | 1.79 (1.55 - 2.11) | 21.93 (17.22 - 27.88) | 6.7 (5.73 - 7.75) | 34.2 (26.56 - 43.13) | 1.6 (1.38 - 1.83) |
| Argentina | 17.89 (12.89 - 23.06) | 0.32 (0.19 - 0.47) | 25.97 (18.48 - 33.78) | 0.28 (0.16 - 0.43) | -0.37 (-0.4 - -0.34) | 2599.42 (2208.04 - 3031.62) | 46.26 (38.86 - 54.31) | 4292.69 (3664.68 - 5018.89) | 46.48 (39.43 - 54.43) | -0.04 (-0.08 - 0) | 51.3 (47.64 - 55.36) | 0.92 (0.78 - 1.09) | 60.17 (53.51 - 66.52) | 0.64 (0.52 - 0.77) | -1.26 (-1.57 - -0.96) | 1926.36 (1664.19 - 2205.77) | 34.21 (27.75 - 41.9) | 2531.29 (2105.85 - 2996.89) | 27.52 (21.44 - 35.05) | -0.8 (-1.01 - -0.59) |
| Armenia | 3.34 (2.69 - 4.01) | 0.62 (0.43 - 0.81) | 5.25 (4.28 - 6.31) | 0.71 (0.5 - 0.92) | 0.51 (0.36 - 0.66) | 333 (277.39 - 393.53) | 67.33 (55.85 - 79.63) | 796.46 (693.07 - 911.55) | 100.85 (87.59 - 115.11) | 1.5 (1.36 - 1.65) | 3.15 (2.56 - 3.76) | 0.85 (0.68 - 1.03) | 6.9 (5.77 - 8.21) | 0.93 (0.73 - 1.16) | 2.63 (1.35 - 3.94) | 136.77 (104.92 - 176.23) | 29.73 (21.5 - 40.43) | 289.36 (226.99 - 368.67) | 37.29 (25.42 - 51.72) | 1.73 (1.26 - 2.2) |
| Australia | 13.29 (9.48 - 16.35) | 0.42 (0.26 - 0.57) | 28.65 (20.67 - 36.31) | 0.4 (0.25 - 0.56) | -0.15 (-0.18 - -0.12) | 2666.46 (2339.63 - 3017.15) | 81.72 (71.6 - 92.65) | 9829.61 (8479.32 - 11261.9) | 134.93 (116.54 - 155.08) | 1.71 (1.31 - 2.11) | 53.62 (46.46 - 61.91) | 1.6 (1.33 - 1.92) | 133.54 (111.82 - 155.72) | 1.75 (1.4 - 2.12) | 0.28 (0.16 - 0.4) | 1921.34 (1651.37 - 2239.33) | 58.59 (47.35 - 71.77) | 5347.15 (4459.38 - 6278.11) | 73.69 (57.86 - 92.28) | 0.76 (0.57 - 0.95) |
| Austria | 9.1 (6.93 - 11.24) | 0.49 (0.32 - 0.65) | 14.84 (11.51 - 18.79) | 0.52 (0.34 - 0.7) | 0.2 (0.18 - 0.22) | 2141.23 (1814.07 - 2534.01) | 111.73 (94.71 - 132.2) | 4766.94 (4128.19 - 5509.29) | 164.85 (143.02 - 189.94) | 1.34 (1.23 - 1.45) | 44.15 (40.11 - 48.99) | 2.24 (1.86 - 2.67) | 91 (80.74 - 102.79) | 3.06 (2.48 - 3.69) | 1.8 (1.52 - 2.08) | 1550.58 (1365.42 - 1758.39) | 81.9 (67.56 - 99.47) | 3203.43 (2771.76 - 3646.69) | 112.37 (91.32 - 135.39) | 1.58 (1.41 - 1.75) |
| Azerbaijan | 4.93 (3.83 - 6.17) | 0.5 (0.33 - 0.67) | 10.23 (7.88 - 12.79) | 0.46 (0.3 - 0.62) | -0.27 (-0.31 - -0.24) | 417.71 (331.34 - 513.13) | 47 (37.31 - 58.2) | 1046.36 (857.13 - 1239.47) | 53.2 (43.34 - 63.53) | 0.49 (0.42 - 0.56) | 3 (1.84 - 4.61) | 0.45 (0.25 - 0.73) | 4.57 (2.33 - 8.2) | 0.36 (0.15 - 0.7) | 0.45 (-0.14 - 1.04) | 158.34 (113.87 - 216.62) | 18.8 (11.94 - 27.73) | 352.88 (250.09 - 487.04) | 19.33 (11.88 - 29.43) | 0.47 (0.28 - 0.65) |
| Bahamas | 0.09 (0.07 - 0.11) | 0.38 (0.25 - 0.49) | 0.26 (0.19 - 0.33) | 0.36 (0.23 - 0.48) | -0.17 (-0.18 - -0.15) | 4.69 (3.76 - 5.76) | 18.85 (14.92 - 23.14) | 17.88 (14.59 - 21.38) | 24.11 (19.54 - 29.07) | 0.84 (0.77 - 0.9) | 0.19 (0.17 - 0.21) | 0.74 (0.61 - 0.9) | 0.75 (0.58 - 0.94) | 1.02 (0.75 - 1.34) | 1.54 (1.35 - 1.73) | 6.32 (5.57 - 7.15) | 24.99 (20.85 - 29.81) | 25.12 (20.02 - 30.66) | 32.79 (25.26 - 41.36) | 1.3 (1.15 - 1.44) |
| Bahrain | 0.11 (0.08 - 0.14) | 0.37 (0.23 - 0.52) | 0.68 (0.49 - 0.89) | 0.39 (0.25 - 0.54) | 0.16 (0.15 - 0.18) | 16.02 (13.17 - 19.17) | 56.99 (46.58 - 68.31) | 138.82 (114.76 - 165.4) | 86.93 (71.55 - 103.54) | 1.39 (1.36 - 1.42) | 0.01 (0.01 - 0.01) | 0.04 (0.02 - 0.08) | 0.51 (0.35 - 0.71) | 0.41 (0.22 - 0.72) | 6.02 (4.38 - 7.69) | 4.29 (2.75 - 6.02) | 15.3 (9.05 - 23.13) | 47.24 (35.79 - 61.96) | 29.3 (19.36 - 41.84) | 2.23 (1.91 - 2.56) |
| Bangladesh | 22.98 (16.22 - 29.64) | 0.3 (0.18 - 0.44) | 64.83 (45.38 - 84.07) | 0.28 (0.16 - 0.41) | -0.31 (-0.32 - -0.29) | 905.16 (716.84 - 1105.94) | 11.48 (8.94 - 14.3) | 3343.27 (2682.67 - 4020) | 13.98 (11.08 - 17.07) | 0.66 (0.61 - 0.71) | 2.14 (0.37 - 4.24) | 0.03 (0 - 0.08) | 11.83 (3.39 - 21.18) | 0.05 (0.01 - 0.12) | 1.85 (1.77 - 1.93) | 308.34 (207.38 - 439.87) | 3.92 (2.55 - 5.75) | 1226.49 (840.74 - 1706.09) | 5.14 (3.44 - 7.54) | 0.88 (0.83 - 0.94) |
| Barbados | 0.18 (0.14 - 0.22) | 0.39 (0.26 - 0.5) | 0.35 (0.27 - 0.44) | 0.39 (0.25 - 0.52) | 0 (-0.03 - 0.03) | 11.32 (9.24 - 13.36) | 25.19 (20.53 - 30) | 36.25 (31.25 - 41.2) | 39.86 (34.22 - 45.4) | 1.59 (1.4 - 1.78) | 0.42 (0.38 - 0.46) | 0.91 (0.75 - 1.1) | 1.27 (0.96 - 1.6) | 1.39 (1 - 1.87) | 2.05 (1.8 - 2.29) | 12.88 (11.44 - 14.62) | 29.6 (24.58 - 35.68) | 40.73 (32.59 - 50.63) | 44.83 (33.87 - 58.66) | 1.89 (1.68 - 2.09) |
| Belarus | 8.01 (6.43 - 10.09) | 0.34 (0.25 - 0.46) | 4.85 (3.27 - 6.59) | 0.17 (0.1 - 0.27) | -2.23 (-2.57 - -1.88) | 567.35 (463.4 - 696.57) | 23.94 (19.31 - 29.39) | 854.93 (738.37 - 982.7) | 29.66 (25.35 - 34.28) | 0.68 (0.5 - 0.86) | 19.94 (17.94 - 22.02) | 0.86 (0.72 - 1.02) | 20.34 (15.84 - 24.82) | 0.7 (0.52 - 0.91) | -1.12 (-1.46 - -0.78) | 663.7 (585.4 - 742.1) | 27.91 (23.7 - 32.8) | 733.1 (584.89 - 888.48) | 25.47 (19.51 - 32.13) | -0.7 (-0.96 - -0.43) |
| Belgium | 12.91 (10.02 - 16.34) | 0.51 (0.33 - 0.68) | 19.15 (14.95 - 24.46) | 0.53 (0.34 - 0.72) | 0.19 (0.16 - 0.21) | 3257.61 (2862.25 - 3718.81) | 125.87 (110.54 - 143.85) | 6099.12 (5117.13 - 7166.5) | 165.17 (139.36 - 193.2) | 0.98 (0.91 - 1.04) | 67.75 (61.97 - 73.9) | 2.56 (2.15 - 3.03) | 113.92 (99.63 - 127.53) | 2.98 (2.43 - 3.63) | 0.66 (0.54 - 0.79) | 2407.34 (2132.51 - 2715.72) | 93.16 (76.7 - 112.99) | 4015.98 (3467.57 - 4628.85) | 110.21 (89.04 - 133.96) | 0.71 (0.6 - 0.81) |
| Belize | 0.05 (0.04 - 0.06) | 0.33 (0.2 - 0.46) | 0.15 (0.11 - 0.2) | 0.31 (0.18 - 0.44) | -0.23 (-0.25 - -0.2) | 2.1 (1.69 - 2.56) | 13.62 (10.74 - 16.76) | 8.4 (6.8 - 10.09) | 16.35 (13.08 - 19.92) | 0.62 (0.57 - 0.66) | 0.02 (0.02 - 0.03) | 0.15 (0.12 - 0.18) | 0.15 (0.13 - 0.17) | 0.29 (0.23 - 0.36) | 2.22 (1.85 - 2.59) | 1.18 (0.98 - 1.44) | 7.66 (6.19 - 9.41) | 6.4 (5.42 - 7.58) | 12.1 (9.89 - 14.73) | 1.57 (1.38 - 1.75) |
| Benin | 1.08 (0.78 - 1.36) | 0.34 (0.21 - 0.46) | 2.66 (1.95 - 3.43) | 0.32 (0.19 - 0.45) | -0.14 (-0.16 - -0.12) | 35.18 (27.76 - 43.56) | 10.72 (8.25 - 13.5) | 104.68 (82.43 - 129.65) | 12 (9.29 - 15.03) | 0.35 (0.34 - 0.37) | 0 (0 - 0) | 0 (0 - 0) | 0 (0 - 0.01) | 0 (0 - 0) | 0.79 (0.7 - 0.87) | 9.93 (6.61 - 14.68) | 3.03 (1.99 - 4.46) | 29.56 (19.96 - 42.59) | 3.39 (2.25 - 4.91) | 0.36 (0.34 - 0.37) |
| Bermuda | 0.04 (0.03 - 0.05) | 0.4 (0.27 - 0.51) | 0.09 (0.07 - 0.11) | 0.38 (0.24 - 0.5) | -0.16 (-0.17 - -0.15) | 2.96 (2.41 - 3.51) | 27.83 (22.56 - 33.26) | 8.31 (7.01 - 9.9) | 35.9 (30.05 - 42.73) | 0.85 (0.77 - 0.93) | 0.09 (0.08 - 0.1) | 0.86 (0.71 - 1.04) | 0.16 (0.13 - 0.2) | 0.68 (0.52 - 0.91) | -0.7 (-0.95 - -0.46) | 3.06 (2.71 - 3.48) | 28.53 (23.22 - 34.82) | 5.91 (4.91 - 7.24) | 25.87 (19.94 - 33.31) | -0.29 (-0.45 - -0.13) |
| Bhutan | 0.13 (0.09 - 0.16) | 0.31 (0.19 - 0.45) | 0.33 (0.24 - 0.41) | 0.33 (0.2 - 0.46) | 0.21 (0.17 - 0.25) | 5.42 (4.22 - 6.68) | 12.68 (9.77 - 15.74) | 16.79 (13.44 - 20.03) | 16.6 (13.2 - 20.05) | 0.97 (0.92 - 1.02) | 0.01 (0 - 0.02) | 0.03 (0 - 0.07) | 0.05 (0.02 - 0.1) | 0.05 (0.01 - 0.13) | 2.51 (2.45 - 2.56) | 1.8 (1.22 - 2.6) | 4.21 (2.77 - 6.14) | 5.97 (4.15 - 8.43) | 5.92 (3.96 - 8.49) | 1.21 (1.16 - 1.26) |
| Bolivia (Plurinational State of) | 2.21 (1.77 - 2.62) | 0.42 (0.32 - 0.51) | 7.06 (5.76 - 8.27) | 0.46 (0.34 - 0.55) | 0.31 (0.25 - 0.36) | 86.02 (68.29 - 106.09) | 15.98 (12.57 - 19.76) | 416.62 (343.83 - 498.06) | 26.44 (21.73 - 31.43) | 1.87 (1.73 - 2.01) | 1.49 (0.69 - 2.6) | 0.3 (0.1 - 0.69) | 7.8 (4.29 - 13.85) | 0.53 (0.2 - 1.2) | 2.09 (1.97 - 2.21) | 60.83 (40.38 - 90.89) | 11.35 (6.55 - 20.28) | 298.4 (199.69 - 441.59) | 19.07 (10.92 - 33.72) | 1.88 (1.75 - 2.01) |
| Bosnia and Herzegovina | 3.42 (2.65 - 4.34) | 0.44 (0.29 - 0.59) | 3.23 (2.36 - 4.22) | 0.31 (0.18 - 0.46) | -1.32 (-1.38 - -1.27) | 365.55 (313.85 - 418.49) | 46.72 (39.8 - 54.06) | 564.68 (507.47 - 623.57) | 51.81 (46.35 - 57.39) | 0.4 (0.35 - 0.44) | 12.08 (8.24 - 16.27) | 1.63 (1.01 - 2.44) | 12.27 (7.72 - 18.44) | 1.11 (0.64 - 1.8) | -1.45 (-1.68 - -1.21) | 418.49 (312.07 - 543.17) | 52.9 (36.79 - 73.32) | 439.8 (321.39 - 582.61) | 40.44 (27.25 - 57.84) | -1 (-1.15 - -0.85) |
| Botswana | 0.29 (0.2 - 0.38) | 0.31 (0.19 - 0.45) | 0.74 (0.52 - 0.97) | 0.3 (0.18 - 0.44) | -0.05 (-0.1 - -0.01) | 10.06 (7.82 - 12.51) | 10.21 (7.83 - 12.86) | 30.07 (23.59 - 36.5) | 11.77 (9.16 - 14.55) | 0.47 (0.41 - 0.53) | 0.04 (0.01 - 0.06) | 0.04 (0.01 - 0.09) | 0.12 (0.08 - 0.18) | 0.05 (0.02 - 0.11) | 1.09 (0.81 - 1.36) | 3.77 (2.62 - 5.36) | 3.82 (2.54 - 5.57) | 11.65 (8.37 - 15.77) | 4.56 (3.12 - 6.4) | 0.64 (0.56 - 0.71) |
| Brazil | 82.33 (68.94 - 97.69) | 0.54 (0.4 - 0.66) | 243 (201.15 - 291.24) | 0.55 (0.4 - 0.69) | 0.06 (0.03 - 0.08) | 6776.81 (5576.51 - 7970.15) | 44.93 (36.96 - 53.08) | 25654.77 (21492.22 - 30032.2) | 59.02 (49.33 - 69.25) | 1.03 (0.91 - 1.16) | 56.53 (52.79 - 59.78) | 0.44 (0.39 - 0.48) | 215.3 (191.74 - 232.21) | 0.51 (0.44 - 0.56) | -0.25 (-0.86 - 0.37) | 2994.09 (2438.71 - 3687.95) | 20.32 (16.44 - 25) | 11182.36 (9149.7 - 13532.46) | 25.74 (20.82 - 31.31) | 0.5 (0.22 - 0.78) |
| Brunei Darussalam | 0.03 (0.02 - 0.05) | 0.2 (0.11 - 0.33) | 0.14 (0.09 - 0.19) | 0.23 (0.13 - 0.36) | 0.52 (0.44 - 0.6) | 1.33 (1.05 - 1.66) | 8.24 (6.36 - 10.37) | 5.93 (4.68 - 7.18) | 9.47 (7.4 - 11.58) | 0.47 (0.42 - 0.52) | 0.01 (0 - 0.03) | 0.07 (0.02 - 0.19) | 0.05 (0.02 - 0.13) | 0.08 (0.02 - 0.24) | 0.62 (0.48 - 0.75) | 0.66 (0.42 - 1.13) | 4.09 (2.44 - 7.23) | 2.86 (1.75 - 5.32) | 4.53 (2.63 - 8.61) | 0.49 (0.44 - 0.55) |
| Bulgaria | 20.28 (16.74 - 24.96) | 0.9 (0.67 - 1.16) | 11.06 (8.71 - 13.76) | 0.55 (0.37 - 0.76) | -1.64 (-1.69 - -1.58) | 2805.2 (2521.9 - 3102.67) | 120.15 (107.5 - 132.86) | 2674.12 (2427.12 - 2944.05) | 114.9 (103.78 - 126.49) | -0.16 (-0.29 - -0.03) | 46.29 (40.89 - 51.49) | 2.17 (1.8 - 2.61) | 43.04 (35.05 - 52.17) | 1.87 (1.48 - 2.32) | -1.18 (-1.62 - -0.74) | 1823.97 (1590.74 - 2085.77) | 79.29 (64.46 - 97.08) | 1690.31 (1424.51 - 1982.23) | 75.76 (60.3 - 94.53) | -0.5 (-0.73 - -0.28) |
| Burkina Faso | 2.52 (1.84 - 3.1) | 0.35 (0.22 - 0.47) | 5.06 (3.7 - 6.34) | 0.33 (0.21 - 0.46) | -0.12 (-0.13 - -0.11) | 84.6 (66.87 - 104.75) | 11.01 (8.5 - 13.75) | 188.96 (150.65 - 231.8) | 11.92 (9.33 - 14.72) | 0.27 (0.26 - 0.29) | 0 (0 - 0) | 0 (0 - 0) | 0.01 (0 - 0.01) | 0 (0 - 0) | 0.86 (0.74 - 0.98) | 23.87 (16.01 - 34.23) | 3.11 (2.04 - 4.51) | 53.35 (36.21 - 74.88) | 3.37 (2.24 - 4.81) | 0.28 (0.26 - 0.29) |
| Burundi | 0.92 (0.62 - 1.24) | 0.24 (0.14 - 0.37) | 1.84 (1.24 - 2.58) | 0.23 (0.13 - 0.35) | -0.24 (-0.26 - -0.22) | 24.8 (18.74 - 31.2) | 6.28 (4.66 - 8.18) | 56.22 (42.6 - 70.94) | 6.47 (4.87 - 8.32) | 0.04 (-0.01 - 0.09) | 0.14 (0.04 - 0.27) | 0.04 (0.01 - 0.1) | 0.36 (0.07 - 0.73) | 0.05 (0.01 - 0.13) | 0.41 (0.27 - 0.56) | 10.46 (6.4 - 15.47) | 2.67 (1.59 - 4.38) | 25.04 (14.49 - 36.73) | 2.94 (1.64 - 4.85) | 0.15 (0.08 - 0.22) |
| Cabo Verde | 0.12 (0.09 - 0.15) | 0.31 (0.19 - 0.44) | 0.25 (0.18 - 0.32) | 0.32 (0.19 - 0.45) | 0.13 (0.08 - 0.18) | 5.39 (4.35 - 6.48) | 13.83 (10.96 - 16.94) | 13.33 (10.77 - 15.98) | 16.95 (13.63 - 20.43) | 0.77 (0.72 - 0.82) | 0 (0 - 0) | 0 (0 - 0) | 0 (0 - 0) | 0 (0 - 0) | 2.31 (2.2 - 2.42) | 1.52 (1.03 - 2.14) | 3.9 (2.6 - 5.57) | 3.76 (2.62 - 5.31) | 4.79 (3.26 - 6.75) | 0.77 (0.72 - 0.82) |
| Cambodia | 2.51 (1.83 - 3.14) | 0.35 (0.23 - 0.45) | 7.23 (5.51 - 8.99) | 0.34 (0.24 - 0.45) | 0.02 (0 - 0.04) | 17.72 (12.86 - 23.26) | 2.24 (1.51 - 3.13) | 68.64 (51.58 - 89.45) | 2.99 (2.12 - 4.09) | 0.99 (0.94 - 1.04) | 0.09 (0.03 - 0.18) | 0.01 (0 - 0.04) | 0.55 (0.23 - 0.95) | 0.03 (0.01 - 0.07) | 2.45 (2.27 - 2.63) | 7.18 (4.64 - 10.8) | 0.93 (0.55 - 1.51) | 32.83 (21.92 - 46.87) | 1.46 (0.89 - 2.42) | 1.54 (1.48 - 1.59) |
| Cameroon | 2.51 (1.82 - 3.2) | 0.34 (0.21 - 0.46) | 6.88 (4.99 - 8.71) | 0.33 (0.21 - 0.46) | 0.01 (-0.05 - 0.06) | 80.06 (61.99 - 98.51) | 10.04 (7.66 - 12.69) | 256.14 (202.29 - 310.82) | 11.73 (9.09 - 14.54) | 0.5 (0.46 - 0.55) | 0 (0 - 0.01) | 0 (0 - 0) | 0.01 (0.01 - 0.02) | 0 (0 - 0) | 0.69 (0.59 - 0.79) | 22.62 (14.94 - 33.5) | 2.84 (1.83 - 4.17) | 72.38 (48.72 - 103.22) | 3.32 (2.21 - 4.78) | 0.5 (0.46 - 0.55) |
| Canada | 32.95 (29.62 - 36.32) | 0.63 (0.53 - 0.71) | 71.99 (62.58 - 81.89) | 0.61 (0.49 - 0.73) | -0.25 (-0.55 - 0.05) | 12134.07 (11788.59 - 12482.28) | 223.48 (217.21 - 230.04) | 33938.75 (32990.47 - 34962.03) | 279.04 (271.14 - 287.36) | 0.66 (0.59 - 0.73) | 153.42 (138.92 - 168.14) | 2.77 (2.33 - 3.28) | 444.64 (392.69 - 499.12) | 3.55 (2.9 - 4.28) | 0.71 (0.43 - 0.99) | 6614.6 (5686.76 - 7539.83) | 121.74 (99.57 - 146.77) | 18197.29 (15624.06 - 20680.12) | 150.03 (122.18 - 179.49) | 0.61 (0.47 - 0.75) |
| Central African Republic | 0.5 (0.33 - 0.69) | 0.26 (0.15 - 0.4) | 0.93 (0.62 - 1.29) | 0.25 (0.15 - 0.38) | -0.09 (-0.1 - -0.08) | 12.99 (9.68 - 16.71) | 6.12 (4.5 - 8.04) | 26.25 (20.14 - 33.04) | 6.48 (4.83 - 8.41) | 0.13 (0.09 - 0.16) | 0.08 (0.02 - 0.15) | 0.05 (0.01 - 0.11) | 0.2 (0.07 - 0.4) | 0.06 (0.01 - 0.15) | 0.8 (0.68 - 0.91) | 5.84 (3.63 - 8.65) | 2.8 (1.64 - 4.59) | 12.92 (8.1 - 19.36) | 3.26 (1.92 - 5.47) | 0.4 (0.36 - 0.44) |
| Chad | 1.65 (1.23 - 2.03) | 0.36 (0.22 - 0.48) | 3.23 (2.37 - 4.05) | 0.34 (0.21 - 0.47) | -0.11 (-0.12 - -0.1) | 53.5 (41.97 - 66.71) | 11.22 (8.59 - 14.13) | 116.58 (93.25 - 141.21) | 11.69 (9.18 - 14.4) | 0.1 (0.04 - 0.16) | 0 (0 - 0) | 0 (0 - 0) | 0 (0 - 0.01) | 0 (0 - 0) | 1.25 (1.18 - 1.32) | 15.09 (10.28 - 21.48) | 3.16 (2.08 - 4.6) | 32.91 (22.1 - 47.09) | 3.3 (2.19 - 4.74) | 0.1 (0.05 - 0.16) |
| Chile | 4.74 (3.21 - 6.21) | 0.28 (0.16 - 0.42) | 11.45 (7.79 - 15.12) | 0.26 (0.14 - 0.39) | -0.24 (-0.31 - -0.18) | 617.61 (507.65 - 731.96) | 36.32 (29.6 - 43.45) | 1814.96 (1493.93 - 2147.61) | 40.53 (33.31 - 48.2) | 0.37 (0.32 - 0.41) | 8.91 (8.05 - 9.84) | 0.53 (0.44 - 0.63) | 12.39 (10.77 - 14.02) | 0.28 (0.22 - 0.34) | -2.07 (-2.3 - -1.84) | 383.97 (325.24 - 456.71) | 22.47 (17.91 - 28.4) | 767.24 (603.12 - 959.41) | 17.13 (12.52 - 23.48) | -0.87 (-0.97 - -0.77) |
| China | 508.61 (392.12 - 619.52) | 0.36 (0.26 - 0.45) | 1151.42 (894.81 - 1427.5) | 0.31 (0.22 - 0.41) | -0.58 (-0.6 - -0.55) | 2834.41 (2123.35 - 3768.69) | 1.9 (1.3 - 2.64) | 13513.3 (10638.61 - 16740.54) | 3.51 (2.58 - 4.61) | 1.76 (1.59 - 1.92) | 19.12 (10.89 - 29) | 0.02 (0.01 - 0.03) | 63.2 (50.09 - 79.33) | 0.02 (0.01 - 0.02) | 0.05 (-0.53 - 0.62) | 1270.65 (907.34 - 1760.98) | 0.87 (0.59 - 1.24) | 5250.6 (3863.53 - 7091.77) | 1.37 (0.98 - 1.85) | 1.17 (1.02 - 1.33) |
| Colombia | 8.18 (5.83 - 10.65) | 0.29 (0.17 - 0.42) | 26.99 (19.26 - 34.8) | 0.28 (0.16 - 0.42) | -0.06 (-0.09 - -0.04) | 281.1 (223.83 - 345.63) | 9.53 (7.45 - 11.9) | 1374.82 (1113.44 - 1642.16) | 14.35 (11.52 - 17.26) | 1.36 (1.3 - 1.42) | 8.56 (7.8 - 9.43) | 0.3 (0.25 - 0.36) | 43.86 (35.41 - 53.36) | 0.46 (0.35 - 0.59) | 1.29 (0.85 - 1.73) | 291.17 (258.51 - 329.94) | 9.85 (8.29 - 11.65) | 1426.23 (1186.86 - 1712.29) | 14.9 (11.85 - 18.49) | 1.27 (0.92 - 1.61) |
| Comoros | 0.09 (0.06 - 0.12) | 0.26 (0.15 - 0.39) | 0.21 (0.14 - 0.28) | 0.25 (0.15 - 0.38) | -0.1 (-0.12 - -0.08) | 2.92 (2.23 - 3.66) | 8.4 (6.34 - 10.73) | 7.99 (6.3 - 9.8) | 9.43 (7.34 - 11.78) | 0.42 (0.38 - 0.45) | 0.02 (0 - 0.03) | 0.05 (0.01 - 0.13) | 0.07 (0.03 - 0.13) | 0.09 (0.02 - 0.23) | 1.86 (1.71 - 2.02) | 1.21 (0.74 - 1.78) | 3.53 (2.1 - 5.6) | 4.03 (2.53 - 5.71) | 4.81 (2.86 - 7.92) | 0.97 (0.92 - 1.02) |
| Congo | 0.42 (0.28 - 0.59) | 0.24 (0.13 - 0.36) | 1.01 (0.68 - 1.43) | 0.23 (0.13 - 0.35) | -0.06 (-0.1 - -0.03) | 12.12 (9.28 - 15.35) | 6.28 (4.69 - 8.15) | 34.11 (26.16 - 42.22) | 7.23 (5.42 - 9.12) | 0.49 (0.46 - 0.52) | 0.11 (0.07 - 0.16) | 0.06 (0.03 - 0.13) | 0.4 (0.25 - 0.62) | 0.1 (0.04 - 0.2) | 1.15 (1.07 - 1.24) | 6.2 (4.62 - 8.25) | 3.27 (2.12 - 4.92) | 19.99 (14.64 - 27.13) | 4.3 (2.73 - 6.88) | 0.82 (0.78 - 0.85) |
| Cook Islands | 0.01 (0 - 0.01) | 0.31 (0.2 - 0.43) | 0.01 (0.01 - 0.02) | 0.28 (0.17 - 0.4) | -0.47 (-0.5 - -0.44) | 0.07 (0.06 - 0.09) | 3.28 (2.3 - 4.43) | 0.19 (0.14 - 0.23) | 3.89 (2.86 - 5.08) | 0.47 (0.45 - 0.5) | 0 (0 - 0) | 0 (0 - 0) | 0 (0 - 0) | 0 (0 - 0) | 1.07 (0.96 - 1.17) | 0.02 (0.01 - 0.03) | 0.93 (0.57 - 1.4) | 0.05 (0.03 - 0.08) | 1.1 (0.7 - 1.62) | 0.47 (0.45 - 0.5) |
| Costa Rica | 1.01 (0.79 - 1.22) | 0.36 (0.24 - 0.46) | 3.47 (2.66 - 4.24) | 0.36 (0.24 - 0.48) | 0.12 (0.07 - 0.16) | 46.73 (37.66 - 56.42) | 16.33 (13.11 - 19.86) | 233.44 (196.33 - 273.91) | 24.32 (20.32 - 28.75) | 1.46 (1.31 - 1.6) | 1.03 (0.94 - 1.13) | 0.36 (0.3 - 0.44) | 6.8 (5.66 - 7.74) | 0.71 (0.55 - 0.89) | 2.01 (1.6 - 2.42) | 37.47 (32.31 - 43.6) | 13.08 (10.9 - 15.79) | 225.48 (191.28 - 261.14) | 23.41 (18.47 - 29.03) | 1.77 (1.45 - 2.09) |
| Croatia | 4.61 (3.5 - 5.76) | 0.41 (0.27 - 0.56) | 3.83 (2.63 - 5.21) | 0.27 (0.16 - 0.42) | -1.23 (-1.33 - -1.14) | 403.01 (336.96 - 463.86) | 34.29 (28.58 - 39.89) | 797.35 (711.53 - 892.1) | 55.66 (49.51 - 62.33) | 1.74 (1.59 - 1.9) | 24.74 (22.57 - 27.23) | 2.26 (1.91 - 2.66) | 24.25 (20.54 - 28.07) | 1.61 (1.29 - 1.97) | -0.95 (-1.12 - -0.78) | 731.07 (661.5 - 813.98) | 63.19 (53.78 - 74.27) | 763.79 (644.68 - 877.95) | 53.37 (42.6 - 65.16) | -0.37 (-0.49 - -0.26) |
| Cuba | 6.14 (4.54 - 7.4) | 0.36 (0.23 - 0.48) | 12.26 (8.97 - 15.22) | 0.36 (0.22 - 0.49) | -0.05 (-0.07 - -0.03) | 361.2 (295.31 - 434.39) | 21.38 (17.22 - 25.8) | 1023.04 (853.9 - 1200.86) | 29.99 (24.8 - 35.56) | 1.14 (1.05 - 1.22) | 11.29 (10.29 - 12.32) | 0.67 (0.56 - 0.79) | 36.9 (30.83 - 43.67) | 1.09 (0.85 - 1.36) | 1.91 (1.72 - 2.09) | 382 (339.92 - 432.78) | 22.77 (19.08 - 27.15) | 1206.82 (1010.72 - 1415.95) | 35.58 (28.31 - 44.11) | 1.71 (1.57 - 1.84) |
| Cyprus | 0.59 (0.44 - 0.72) | 0.43 (0.28 - 0.57) | 1.49 (1.09 - 1.85) | 0.44 (0.27 - 0.61) | 0.07 (0.04 - 0.1) | 90.98 (79.67 - 101.88) | 65.01 (56.98 - 73.15) | 348.74 (294.99 - 407.97) | 100.52 (84.74 - 117.77) | 1.74 (1.59 - 1.89) | 2.37 (1.08 - 4.05) | 1.77 (0.59 - 4.07) | 5.84 (3.93 - 8.27) | 1.65 (0.82 - 2.98) | -0.08 (-0.18 - 0.02) | 77.08 (46.58 - 117.6) | 55.57 (27.68 - 106.32) | 219.65 (167.55 - 282.35) | 63.3 (40.04 - 95.9) | 0.62 (0.53 - 0.71) |
| Czechia | 10.78 (8.41 - 13.67) | 0.47 (0.31 - 0.65) | 8.61 (6.02 - 11.64) | 0.27 (0.16 - 0.41) | -1.85 (-1.91 - -1.79) | 1285.45 (1142.74 - 1443.5) | 54.83 (48.21 - 61.88) | 1798.55 (1593.86 - 2034.05) | 54.84 (48.41 - 61.8) | -0.15 (-0.21 - -0.09) | 80.84 (73.19 - 89.78) | 3.39 (2.86 - 4) | 73.36 (62.75 - 85.38) | 2.08 (1.67 - 2.56) | -1.47 (-1.71 - -1.23) | 2337.06 (2106.76 - 2574.5) | 99.17 (84.73 - 115.16) | 2136.79 (1850.09 - 2444.57) | 64.3 (52.71 - 77.78) | -1.3 (-1.49 - -1.11) |
| Côte d'Ivoire | 2.17 (1.56 - 2.76) | 0.33 (0.21 - 0.46) | 5.8 (4.14 - 7.52) | 0.32 (0.19 - 0.45) | -0.15 (-0.18 - -0.12) | 68.22 (53.33 - 84.96) | 9.65 (7.38 - 12.22) | 212.43 (167.98 - 260.81) | 10.98 (8.52 - 13.7) | 0.41 (0.39 - 0.44) | 0 (0 - 0) | 0 (0 - 0) | 0.01 (0 - 0.02) | 0 (0 - 0) | 0.78 (0.73 - 0.83) | 19.26 (12.96 - 27.7) | 2.73 (1.79 - 3.97) | 60 (40.09 - 87.37) | 3.1 (2.07 - 4.5) | 0.42 (0.39 - 0.44) |
| Democratic People’s Republic of Korea | 10.69 (8.47 - 12.69) | 0.41 (0.3 - 0.49) | 20.54 (16.04 - 24.95) | 0.37 (0.26 - 0.46) | -0.33 (-0.34 - -0.31) | 93.77 (69.77 - 122.48) | 3.31 (2.36 - 4.52) | 266.48 (207.18 - 341.98) | 4.63 (3.39 - 6.11) | 1.17 (1.11 - 1.23) | 0.48 (0.16 - 0.97) | 0.02 (0 - 0.05) | 1.34 (0.5 - 2.43) | 0.02 (0.01 - 0.06) | 0.81 (0.71 - 0.9) | 38.56 (24.83 - 57.14) | 1.38 (0.84 - 2.22) | 107.63 (73.88 - 153.48) | 1.87 (1.2 - 2.89) | 1.08 (1.04 - 1.12) |
| Democratic Republic of the Congo | 5.94 (3.94 - 8.38) | 0.22 (0.13 - 0.35) | 13.18 (8.6 - 18.78) | 0.21 (0.12 - 0.33) | -0.12 (-0.13 - -0.12) | 182.39 (139.05 - 231.44) | 6.23 (4.64 - 8.02) | 460.95 (358.89 - 577.88) | 6.99 (5.28 - 8.96) | 0.3 (0.24 - 0.37) | 1.1 (0.35 - 2) | 0.04 (0.01 - 0.11) | 4 (1.49 - 7.2) | 0.07 (0.02 - 0.17) | 1.41 (1.2 - 1.62) | 80.46 (52.26 - 116.05) | 2.8 (1.72 - 4.48) | 235.13 (146.17 - 338.7) | 3.61 (2.13 - 5.97) | 0.76 (0.64 - 0.88) |
| Denmark | 4.77 (3.39 - 6.24) | 0.39 (0.22 - 0.59) | 6.91 (4.88 - 9.52) | 0.38 (0.21 - 0.6) | -0.13 (-0.15 - -0.1) | 2230.7 (1983.98 - 2497.06) | 178.65 (158.7 - 199.12) | 3943.59 (3414.4 - 4640.82) | 212.58 (184.8 - 248.37) | 0.75 (0.68 - 0.82) | 63.94 (55.86 - 72.89) | 4.91 (4.03 - 5.91) | 102.85 (89.55 - 114.8) | 5.19 (4.21 - 6.2) | 0.24 (0.13 - 0.36) | 2068.57 (1834.61 - 2380.09) | 167.36 (139.6 - 199.83) | 3246.06 (2818.34 - 3696.19) | 174.34 (145.19 - 208.11) | 0.25 (0.15 - 0.35) |
| Djibouti | 0.06 (0.04 - 0.08) | 0.26 (0.15 - 0.39) | 0.27 (0.18 - 0.38) | 0.26 (0.15 - 0.39) | 0.03 (0 - 0.05) | 1.96 (1.51 - 2.48) | 8.29 (6.27 - 10.66) | 10.36 (8.12 - 12.59) | 9.2 (7.17 - 11.43) | 0.33 (0.26 - 0.39) | 0.01 (0 - 0.02) | 0.05 (0.01 - 0.14) | 0.09 (0.03 - 0.17) | 0.09 (0.02 - 0.23) | 1.56 (1.42 - 1.69) | 0.82 (0.51 - 1.23) | 3.55 (2.11 - 5.8) | 5.13 (3.12 - 7.62) | 4.66 (2.76 - 7.83) | 0.81 (0.78 - 0.84) |
| Dominica | 0.03 (0.02 - 0.04) | 0.33 (0.21 - 0.46) | 0.05 (0.03 - 0.06) | 0.31 (0.19 - 0.44) | -0.24 (-0.26 - -0.21) | 1.42 (1.15 - 1.73) | 14.63 (11.57 - 18.03) | 2.46 (2 - 2.98) | 16.08 (12.89 - 19.65) | 0.22 (0.17 - 0.26) | 0.02 (0.02 - 0.03) | 0.23 (0.14 - 0.36) | 0.05 (0.03 - 0.08) | 0.35 (0.15 - 0.68) | 1.54 (1.38 - 1.71) | 0.96 (0.78 - 1.19) | 10.03 (7.27 - 13.42) | 2.07 (1.52 - 2.87) | 13.36 (8.14 - 21.95) | 1.06 (0.96 - 1.16) |
| Dominican Republic | 2.08 (1.51 - 2.59) | 0.34 (0.21 - 0.46) | 5.41 (3.91 - 6.84) | 0.32 (0.2 - 0.45) | -0.17 (-0.19 - -0.15) | 86.31 (70.01 - 104.89) | 13.78 (10.93 - 16.93) | 295.14 (238.42 - 353.98) | 17.56 (14.06 - 21.34) | 0.75 (0.71 - 0.78) | 0.76 (0.55 - 1) | 0.12 (0.07 - 0.2) | 3 (1.88 - 4.68) | 0.18 (0.08 - 0.35) | 1.56 (1.28 - 1.84) | 44.41 (34.94 - 57.36) | 7.02 (5.09 - 9.44) | 160.89 (115.96 - 217.91) | 9.52 (6.11 - 14.65) | 1.13 (1 - 1.26) |
| Ecuador | 2.89 (2.17 - 3.58) | 0.34 (0.22 - 0.45) | 10.62 (8.52 - 12.67) | 0.38 (0.28 - 0.48) | 0.44 (0.38 - 0.5) | 97.64 (77.62 - 119.1) | 11.26 (8.85 - 13.91) | 490.64 (394.32 - 594.37) | 17.67 (14.19 - 21.45) | 1.53 (1.45 - 1.61) | 2.35 (2.18 - 2.53) | 0.29 (0.24 - 0.34) | 13.55 (10.49 - 17.36) | 0.51 (0.37 - 0.67) | 3.27 (2.47 - 4.07) | 82.12 (72.21 - 94.81) | 9.51 (7.99 - 11.22) | 445.53 (356.85 - 551.99) | 16.14 (12.57 - 20.35) | 2.61 (2.1 - 3.13) |
| Egypt | 15.92 (11.26 - 19.93) | 0.35 (0.21 - 0.48) | 44.04 (31.48 - 56.16) | 0.38 (0.24 - 0.53) | 0.31 (0.27 - 0.35) | 1553.93 (1265.42 - 1854.85) | 33.74 (27.48 - 40.48) | 8381.21 (6949.4 - 9909.12) | 73.81 (60.89 - 87.56) | 2.63 (2.48 - 2.77) | 1.11 (0.62 - 1.66) | 0.03 (0.01 - 0.05) | 4.86 (3 - 7.22) | 0.05 (0.02 - 0.09) | 2.02 (1.88 - 2.16) | 440.21 (303.25 - 619.03) | 9.52 (5.72 - 14.56) | 2176.96 (1434.71 - 3032.46) | 19.03 (10.5 - 30.33) | 2.33 (2.25 - 2.41) |
| El Salvador | 1.58 (1.14 - 1.98) | 0.32 (0.2 - 0.45) | 3.25 (2.41 - 4.01) | 0.32 (0.19 - 0.44) | -0.03 (-0.05 - -0.01) | 50.35 (39.54 - 61.87) | 10.21 (7.99 - 12.68) | 150.34 (123.93 - 178.95) | 14.85 (11.95 - 17.9) | 1.25 (1.21 - 1.29) | 0.61 (0.43 - 0.8) | 0.12 (0.07 - 0.19) | 2.97 (2.11 - 4.03) | 0.29 (0.16 - 0.49) | 2.94 (2.6 - 3.29) | 29.26 (22.95 - 37.18) | 5.92 (4.37 - 7.92) | 113.99 (88.82 - 145.97) | 11.33 (7.73 - 16.19) | 2.25 (2.04 - 2.45) |
| Equatorial Guinea | 0.07 (0.05 - 0.1) | 0.22 (0.13 - 0.35) | 0.18 (0.12 - 0.25) | 0.22 (0.12 - 0.35) | 0.06 (0.01 - 0.1) | 2.06 (1.56 - 2.62) | 5.89 (4.39 - 7.62) | 6.88 (5.26 - 8.61) | 8.2 (6.22 - 10.45) | 1.22 (1.16 - 1.28) | 0.01 (0.01 - 0.02) | 0.04 (0.01 - 0.11) | 0.08 (0.04 - 0.13) | 0.11 (0.04 - 0.24) | 3.08 (2.88 - 3.29) | 0.93 (0.61 - 1.34) | 2.7 (1.66 - 4.34) | 4 (2.81 - 5.62) | 4.82 (3 - 7.91) | 2.04 (1.93 - 2.15) |
| Eritrea | 0.54 (0.37 - 0.73) | 0.3 (0.18 - 0.43) | 1.35 (0.94 - 1.78) | 0.3 (0.18 - 0.44) | 0.09 (0.06 - 0.11) | 17.07 (13.16 - 21.52) | 8.5 (6.39 - 10.96) | 47.74 (37.14 - 59.13) | 9.94 (7.62 - 12.48) | 0.51 (0.49 - 0.54) | 0.09 (0.02 - 0.18) | 0.05 (0.01 - 0.14) | 0.4 (0.12 - 0.73) | 0.1 (0.02 - 0.25) | 2 (1.74 - 2.25) | 7.18 (4.42 - 10.47) | 3.62 (2.14 - 5.8) | 23.94 (14.81 - 34.77) | 5.09 (2.92 - 8.61) | 1.07 (0.99 - 1.16) |
| Estonia | 1.08 (0.84 - 1.35) | 0.3 (0.2 - 0.42) | 0.57 (0.39 - 0.79) | 0.15 (0.08 - 0.23) | -2.37 (-2.68 - -2.05) | 83.86 (68.7 - 99.45) | 23.14 (18.73 - 27.82) | 105.03 (90.4 - 120.96) | 25.98 (22.13 - 30.19) | 0.49 (0.41 - 0.56) | 7.24 (6.36 - 8.31) | 2.03 (1.64 - 2.45) | 4.49 (3.65 - 5.39) | 0.98 (0.77 - 1.21) | -3.23 (-3.63 - -2.83) | 204.95 (178.36 - 236.37) | 56.69 (46.46 - 68.01) | 124.48 (104.06 - 147.22) | 30.13 (23.99 - 36.59) | -2.82 (-3.15 - -2.48) |
| Eswatini | 0.15 (0.1 - 0.19) | 0.32 (0.19 - 0.46) | 0.3 (0.22 - 0.38) | 0.33 (0.2 - 0.46) | 0.06 (0.03 - 0.09) | 5.21 (4.07 - 6.4) | 10.97 (8.42 - 13.73) | 11.94 (9.46 - 14.64) | 12.51 (9.73 - 15.46) | 0.38 (0.29 - 0.47) | 0.02 (0.01 - 0.03) | 0.04 (0.01 - 0.1) | 0.06 (0.04 - 0.1) | 0.07 (0.03 - 0.15) | 1.74 (1.51 - 1.98) | 1.97 (1.35 - 2.71) | 4.14 (2.75 - 5.99) | 5.05 (3.73 - 6.85) | 5.26 (3.59 - 7.62) | 0.8 (0.77 - 0.83) |
| Ethiopia | 8.95 (6.12 - 11.7) | 0.28 (0.16 - 0.4) | 17.65 (12.26 - 23.27) | 0.26 (0.15 - 0.38) | -0.23 (-0.25 - -0.21) | 254.44 (195.3 - 317.53) | 7.27 (5.48 - 9.27) | 608.9 (479.62 - 749.35) | 8.58 (6.67 - 10.73) | 0.56 (0.46 - 0.65) | 1.28 (0.3 - 2.5) | 0.04 (0.01 - 0.09) | 4.32 (1.19 - 7.32) | 0.07 (0.02 - 0.13) | 1.23 (1.07 - 1.38) | 105.04 (63.89 - 155.26) | 3.04 (1.79 - 4.62) | 276.52 (167.26 - 377.7) | 3.92 (2.33 - 5.66) | 0.75 (0.65 - 0.86) |
| Fiji | 0.17 (0.12 - 0.23) | 0.31 (0.2 - 0.43) | 0.39 (0.28 - 0.51) | 0.3 (0.2 - 0.42) | -0.1 (-0.11 - -0.09) | 1.45 (1.06 - 1.91) | 2.4 (1.62 - 3.36) | 3.93 (2.91 - 5.21) | 2.7 (1.89 - 3.71) | 0.31 (0.26 - 0.37) | 0 (0 - 0) | 0 (0 - 0) | 0 (0 - 0) | 0 (0 - 0) | 0.86 (0.62 - 1.1) | 0.41 (0.26 - 0.62) | 0.68 (0.4 - 1.06) | 1.11 (0.71 - 1.65) | 0.76 (0.47 - 1.16) | 0.31 (0.26 - 0.37) |
| Finland | 5.75 (4.52 - 7.1) | 0.5 (0.33 - 0.66) | 9.83 (7.75 - 12.16) | 0.53 (0.35 - 0.71) | 0.14 (0.06 - 0.22) | 1478.62 (1317.03 - 1639.57) | 125.72 (111.76 - 139.1) | 3288.66 (2979.54 - 3632.81) | 167.49 (152.08 - 184.49) | 0.93 (0.84 - 1.02) | 28.44 (25.44 - 31.44) | 2.37 (1.96 - 2.82) | 59.37 (51.72 - 66.69) | 2.89 (2.34 - 3.49) | 1.04 (0.91 - 1.17) | 1038.3 (914.01 - 1181.76) | 88.78 (72.5 - 107.57) | 2111.55 (1844.66 - 2381.1) | 109.92 (89.69 - 132.13) | 0.96 (0.87 - 1.05) |
| France | 47.57 (33.62 - 62.99) | 0.35 (0.21 - 0.54) | 68.74 (49.5 - 91.64) | 0.33 (0.19 - 0.51) | -0.28 (-0.38 - -0.17) | 15951.41 (13587.29 - 18684.71) | 115.97 (98.5 - 136.13) | 37294.13 (32683 - 41781.79) | 172.17 (151.79 - 192.46) | 1.43 (1.28 - 1.58) | 249.53 (225.74 - 274.91) | 1.81 (1.53 - 2.14) | 493.13 (419.79 - 571.58) | 2.2 (1.76 - 2.69) | 0.95 (0.81 - 1.09) | 9890.72 (8591.08 - 11380.62) | 73.22 (59.63 - 89.82) | 20002.96 (16820.94 - 23687.4) | 94.6 (75.56 - 117.6) | 1.08 (0.95 - 1.21) |
| Gabon | 0.22 (0.15 - 0.3) | 0.23 (0.13 - 0.35) | 0.41 (0.28 - 0.57) | 0.23 (0.13 - 0.35) | 0.11 (0.05 - 0.17) | 6.83 (5.23 - 8.72) | 6.67 (4.97 - 8.59) | 15.16 (11.86 - 18.83) | 7.95 (6.1 - 10.05) | 0.56 (0.53 - 0.58) | 0.06 (0.04 - 0.09) | 0.07 (0.03 - 0.14) | 0.17 (0.1 - 0.27) | 0.1 (0.04 - 0.22) | 1.23 (1.09 - 1.38) | 3.44 (2.52 - 4.53) | 3.4 (2.19 - 5.18) | 8.63 (6.22 - 11.89) | 4.61 (2.92 - 7.23) | 0.87 (0.79 - 0.94) |
| Gambia | 0.19 (0.14 - 0.24) | 0.34 (0.21 - 0.46) | 0.55 (0.41 - 0.68) | 0.35 (0.22 - 0.48) | 0.16 (0.11 - 0.2) | 6.58 (5.17 - 8.09) | 11.26 (8.69 - 14.06) | 22.39 (18.12 - 26.97) | 13.89 (11.04 - 17.04) | 0.76 (0.72 - 0.8) | 0 (0 - 0) | 0 (0 - 0) | 0 (0 - 0) | 0 (0 - 0) | 1.44 (1.33 - 1.56) | 1.86 (1.23 - 2.68) | 3.18 (2.08 - 4.6) | 6.33 (4.34 - 9.02) | 3.93 (2.65 - 5.63) | 0.76 (0.72 - 0.8) |
| Georgia | 5.89 (4.56 - 7.32) | 0.52 (0.35 - 0.69) | 5.47 (4.41 - 6.6) | 0.56 (0.37 - 0.74) | 0.27 (0.15 - 0.4) | 602.12 (479.22 - 732.87) | 53.76 (42.69 - 65.72) | 749.39 (632.87 - 859.18) | 71.81 (60.6 - 82.75) | 1.04 (0.95 - 1.12) | 4.32 (3.52 - 5.27) | 0.47 (0.37 - 0.58) | 6.6 (5.36 - 7.84) | 0.58 (0.45 - 0.73) | 3.47 (1.7 - 5.27) | 221.07 (166.78 - 286.48) | 20.52 (14.09 - 28.72) | 283.72 (219.74 - 357.72) | 26.82 (18.94 - 36.88) | 1.73 (1.32 - 2.14) |
| Germany | 105.34 (82.44 - 133.67) | 0.52 (0.34 - 0.69) | 160.08 (124.6 - 204.64) | 0.53 (0.35 - 0.72) | 0.07 (0.06 - 0.09) | 27545.38 (23434.67 - 32689.98) | 133.36 (113.32 - 157.27) | 54103.56 (46461.16 - 63636.02) | 176.39 (152.34 - 206.33) | 0.91 (0.82 - 0.99) | 561.05 (518.84 - 612.05) | 2.66 (2.2 - 3.16) | 1116.36 (989.35 - 1240.54) | 3.53 (2.85 - 4.27) | 1.36 (1.15 - 1.56) | 20091 (17680.94 - 22893.52) | 98.12 (80.53 - 119.7) | 38090.38 (33324.94 - 44036.56) | 126.64 (104.19 - 154.46) | 1.15 (1.04 - 1.26) |
| Ghana | 4.34 (3.36 - 5.35) | 0.42 (0.28 - 0.54) | 13.01 (9.94 - 17.26) | 0.46 (0.3 - 0.63) | 0.44 (0.37 - 0.51) | 235.13 (198.63 - 277.81) | 21.29 (17.77 - 25.29) | 1233.27 (1105.94 - 1352.9) | 42.44 (37.85 - 47.09) | 2.34 (2.14 - 2.54) | 0 (0 - 0.01) | 0 (0 - 0) | 0.02 (0.01 - 0.03) | 0 (0 - 0) | 1.85 (1.71 - 1.98) | 62.67 (41.18 - 89.38) | 5.7 (3.17 - 9.07) | 316.27 (210.46 - 441.23) | 10.91 (5.96 - 17.35) | 2.22 (2.03 - 2.41) |
| Greece | 8.67 (6.13 - 11.29) | 0.33 (0.19 - 0.48) | 11.79 (8.52 - 15.22) | 0.34 (0.2 - 0.5) | 0.04 (-0.01 - 0.08) | 1154.86 (998.41 - 1337.13) | 43.18 (36.8 - 50.07) | 2310.92 (1951.84 - 2735.99) | 66.21 (56 - 77.89) | 1.45 (1.41 - 1.5) | 33.18 (30.29 - 36.54) | 1.23 (1.03 - 1.46) | 81.13 (71.88 - 89.58) | 2.24 (1.84 - 2.69) | 2.13 (2 - 2.27) | 1085.88 (957.64 - 1221.93) | 40.47 (33.7 - 48.76) | 2428.94 (2152.26 - 2693.89) | 72.2 (59.16 - 86.03) | 2.06 (1.95 - 2.18) |
| Greenland | 0 (0 - 0) | 0.01 (0 - 0.04) | 0 (0 - 0) | 0.01 (0 - 0.04) | -0.03 (-0.1 - 0.04) | 4.1 (3.48 - 4.79) | 71.15 (59.89 - 84.11) | 11.06 (9.39 - 12.9) | 78.22 (66.02 - 91.36) | 0.32 (0.24 - 0.39) | 0.01 (0.01 - 0.02) | 0.22 (0.1 - 0.42) | 0.09 (0.06 - 0.15) | 0.7 (0.29 - 1.42) | 6.69 (5.28 - 8.11) | 1.3 (0.92 - 1.71) | 22.7 (14.33 - 33.09) | 5.25 (3.89 - 7.08) | 36.73 (22.4 - 56.8) | 2.6 (2.14 - 3.06) |
| Grenada | 0.05 (0.04 - 0.06) | 0.39 (0.26 - 0.5) | 0.08 (0.06 - 0.1) | 0.39 (0.26 - 0.51) | -0.01 (-0.04 - 0.02) | 2.11 (1.69 - 2.54) | 18.11 (14.42 - 22.08) | 5.07 (4.23 - 5.98) | 24.64 (20.21 - 29.33) | 0.98 (0.83 - 1.13) | 0.09 (0.08 - 0.1) | 0.74 (0.59 - 0.9) | 0.21 (0.18 - 0.25) | 1.05 (0.82 - 1.3) | 1.69 (1.45 - 1.93) | 2.75 (2.43 - 3.14) | 24.13 (20.18 - 28.8) | 6.94 (5.85 - 8.04) | 32.98 (26.42 - 40.14) | 1.47 (1.26 - 1.67) |
| Guam | 0.03 (0.02 - 0.04) | 0.26 (0.15 - 0.38) | 0.08 (0.06 - 0.11) | 0.22 (0.13 - 0.32) | -0.67 (-0.74 - -0.59) | 0.39 (0.29 - 0.5) | 2.84 (1.99 - 3.86) | 1.11 (0.87 - 1.41) | 2.93 (2.1 - 3.92) | 0.04 (-0.03 - 0.11) | 0 (0 - 0) | 0 (0 - 0) | 0 (0 - 0) | 0 (0 - 0) | 0.43 (-0.44 - 1.31) | 0.11 (0.07 - 0.16) | 0.8 (0.49 - 1.21) | 0.31 (0.21 - 0.45) | 0.83 (0.51 - 1.24) | 0.04 (-0.04 - 0.11) |
| Guatemala | 2.03 (1.52 - 2.54) | 0.36 (0.24 - 0.47) | 7.14 (5.73 - 8.64) | 0.39 (0.28 - 0.49) | 0.36 (0.3 - 0.41) | 58.98 (46.21 - 73.57) | 9.82 (7.58 - 12.43) | 301.79 (241.41 - 363.03) | 16.3 (13.02 - 19.8) | 1.75 (1.66 - 1.84) | 1.11 (1.02 - 1.2) | 0.2 (0.17 - 0.24) | 6.01 (5 - 7.16) | 0.33 (0.26 - 0.42) | 1.72 (1.3 - 2.14) | 44.79 (38.76 - 52.66) | 7.48 (6.25 - 8.93) | 234.81 (197.55 - 278.61) | 12.64 (10.29 - 15.22) | 1.84 (1.54 - 2.14) |
| Guinea | 1.86 (1.35 - 2.31) | 0.34 (0.21 - 0.46) | 3.04 (2.22 - 3.82) | 0.33 (0.2 - 0.46) | -0.07 (-0.09 - -0.06) | 58.89 (45.59 - 72.72) | 10.25 (7.78 - 12.95) | 108.86 (85.21 - 133.01) | 11.33 (8.77 - 14.07) | 0.27 (0.24 - 0.29) | 0 (0 - 0) | 0 (0 - 0) | 0 (0 - 0.01) | 0 (0 - 0) | 1.25 (1.22 - 1.28) | 16.62 (10.96 - 23.39) | 2.89 (1.88 - 4.19) | 30.75 (20.52 - 43.8) | 3.2 (2.1 - 4.65) | 0.27 (0.24 - 0.3) |
| Guinea-Bissau | 0.24 (0.18 - 0.3) | 0.38 (0.25 - 0.5) | 0.43 (0.32 - 0.53) | 0.37 (0.24 - 0.49) | -0.05 (-0.09 - -0.01) | 7.32 (5.68 - 9.2) | 10.61 (8.09 - 13.58) | 15.83 (12.54 - 19.47) | 12.51 (9.83 - 15.53) | 0.51 (0.49 - 0.53) | 0 (0 - 0) | 0 (0 - 0) | 0 (0 - 0) | 0 (0 - 0) | 0.98 (0.94 - 1.02) | 2.07 (1.37 - 3.04) | 3 (1.94 - 4.42) | 4.47 (3.01 - 6.35) | 3.54 (2.37 - 5.1) | 0.51 (0.49 - 0.53) |
| Guyana | 0.19 (0.14 - 0.25) | 0.32 (0.19 - 0.44) | 0.32 (0.23 - 0.42) | 0.29 (0.18 - 0.43) | -0.25 (-0.27 - -0.23) | 5.89 (4.56 - 7.24) | 9.34 (7.1 - 11.83) | 13.81 (10.89 - 16.95) | 11.84 (9.23 - 14.74) | 0.72 (0.68 - 0.75) | 0.12 (0.1 - 0.14) | 0.19 (0.15 - 0.24) | 0.33 (0.23 - 0.45) | 0.28 (0.19 - 0.4) | 1.84 (1.66 - 2.02) | 4.93 (4.18 - 5.81) | 7.66 (6.17 - 9.49) | 13 (9.87 - 16.58) | 10.74 (7.89 - 14.19) | 1.47 (1.35 - 1.58) |
| Haiti | 2.19 (1.69 - 2.64) | 0.41 (0.29 - 0.51) | 4.47 (3.32 - 5.58) | 0.38 (0.25 - 0.49) | -0.25 (-0.26 - -0.24) | 75.06 (57.11 - 93.6) | 13.13 (9.94 - 16.58) | 201.29 (159.82 - 242.39) | 15.91 (12.5 - 19.42) | 0.57 (0.52 - 0.61) | 1.58 (0.55 - 3.12) | 0.28 (0.07 - 0.7) | 5.18 (1.99 - 10.09) | 0.42 (0.11 - 1.08) | 1.43 (1.29 - 1.57) | 64.25 (34.48 - 108.86) | 10.97 (5.29 - 21.78) | 199.17 (109.07 - 348.48) | 15.34 (7.25 - 32.74) | 1.16 (1.06 - 1.26) |
| Honduras | 1.12 (0.81 - 1.43) | 0.34 (0.21 - 0.46) | 3.69 (2.77 - 4.55) | 0.35 (0.23 - 0.47) | 0.15 (0.12 - 0.19) | 33.01 (25.54 - 40.4) | 9.63 (7.38 - 12.03) | 146.52 (116.87 - 178.43) | 13.54 (10.7 - 16.64) | 1.13 (1.11 - 1.15) | 0.21 (0.11 - 0.34) | 0.06 (0.02 - 0.14) | 1.13 (0.56 - 1.93) | 0.11 (0.03 - 0.27) | 1.98 (1.85 - 2.11) | 14.63 (10.62 - 19.8) | 4.26 (2.81 - 6.43) | 69.04 (47.45 - 92.81) | 6.38 (3.94 - 10.52) | 1.43 (1.37 - 1.48) |
| Hungary | 10.98 (8.4 - 13.87) | 0.44 (0.28 - 0.61) | 6.89 (4.74 - 9.12) | 0.24 (0.13 - 0.37) | -1.99 (-2.1 - -1.89) | 2049.44 (1842.3 - 2245.37) | 79.46 (71.41 - 87.46) | 1610.79 (1384.54 - 1848.96) | 53.59 (46.27 - 61.62) | -1.33 (-1.88 - -0.78) | 57.69 (52.01 - 63.44) | 2.24 (1.87 - 2.68) | 53.41 (46.12 - 61.54) | 1.71 (1.37 - 2.13) | -0.84 (-1.07 - -0.61) | 1976.15 (1753.57 - 2213.85) | 76.7 (64.06 - 91.35) | 1705.61 (1470.27 - 1938.01) | 57.64 (47.12 - 69.06) | -0.93 (-1 - -0.86) |
| Iceland | 0.31 (0.25 - 0.38) | 0.68 (0.51 - 0.86) | 0.62 (0.51 - 0.76) | 0.66 (0.49 - 0.85) | -0.06 (-0.08 - -0.04) | 95.29 (88.45 - 102.34) | 201.82 (187.31 - 216.84) | 219.03 (184.43 - 260.66) | 226.78 (191.57 - 269.14) | 0.44 (0.39 - 0.5) | 1.32 (1.19 - 1.47) | 2.76 (2.27 - 3.31) | 3.11 (2.63 - 3.56) | 3.17 (2.54 - 3.87) | 0.71 (0.59 - 0.84) | 54.83 (47.6 - 62.65) | 116.9 (95.79 - 140.3) | 125.44 (107.66 - 146.66) | 131.23 (106.53 - 159.77) | 0.51 (0.43 - 0.59) |
| India | 251.49 (179.43 - 318.25) | 0.33 (0.2 - 0.45) | 648.89 (471.21 - 813.62) | 0.32 (0.2 - 0.45) | 0.01 (-0.02 - 0.04) | 9787.89 (7808.39 - 12004.14) | 11.96 (9.34 - 14.84) | 31187.53 (25022.53 - 37629.34) | 15.09 (12.04 - 18.29) | 0.82 (0.78 - 0.86) | 24.09 (8.77 - 40.43) | 0.03 (0.01 - 0.06) | 121.41 (86.29 - 157.32) | 0.06 (0.04 - 0.08) | 2.09 (1.89 - 2.29) | 3384.96 (2359.42 - 4780.16) | 4.14 (2.86 - 5.83) | 11792.95 (8814.37 - 15662.62) | 5.71 (4.23 - 7.62) | 1.08 (1.01 - 1.16) |
| Indonesia | 46.83 (33.36 - 61.9) | 0.3 (0.19 - 0.42) | 130.61 (98.77 - 167.92) | 0.33 (0.23 - 0.43) | 0.4 (0.36 - 0.45) | 414.79 (304.29 - 545.32) | 2.39 (1.63 - 3.31) | 1362.57 (1028.4 - 1747.7) | 3.08 (2.21 - 4.13) | 0.84 (0.78 - 0.9) | 1.72 (1.19 - 2.33) | 0.01 (0.01 - 0.02) | 9.52 (6.5 - 14.7) | 0.03 (0.02 - 0.04) | 2.23 (1.88 - 2.57) | 159.25 (117.1 - 223.08) | 0.93 (0.64 - 1.33) | 617.28 (445.8 - 824.89) | 1.43 (1.01 - 1.99) | 1.29 (1.19 - 1.38) |
| Iran (Islamic Republic of) | 24.98 (19.61 - 31.63) | 0.51 (0.36 - 0.66) | 79.92 (64.67 - 99.14) | 0.59 (0.43 - 0.75) | 0.44 (0.42 - 0.46) | 5149.35 (4351.77 - 5968.62) | 109.34 (92.25 - 127.41) | 16362.78 (14296.33 - 18533.75) | 124.56 (108.63 - 141.2) | 1.25 (0.98 - 1.51) | 32.57 (15.93 - 47.56) | 0.79 (0.35 - 1.27) | 154.63 (125.82 - 186.56) | 1.25 (0.94 - 1.65) | 1.75 (1.58 - 1.93) | 2105.56 (1520.26 - 2743.18) | 44.25 (31.39 - 58.51) | 7592.91 (6333.5 - 8962.24) | 57.59 (46.52 - 70.42) | 1.42 (1.26 - 1.57) |
| Iraq | 5.2 (3.77 - 6.44) | 0.41 (0.26 - 0.55) | 17.04 (12.54 - 21.88) | 0.43 (0.27 - 0.57) | 0.15 (0.13 - 0.17) | 789.92 (643.27 - 953.07) | 62.4 (50.64 - 75.31) | 3586.8 (2935.46 - 4275.77) | 92.21 (75.6 - 109.72) | 1.35 (1.26 - 1.43) | 1.54 (0.54 - 2.67) | 0.12 (0.03 - 0.28) | 7.72 (4.89 - 11.48) | 0.2 (0.09 - 0.4) | 1.49 (1.42 - 1.55) | 240.48 (173.78 - 332.42) | 18.89 (11.8 - 28.33) | 1063.25 (765.95 - 1404.34) | 26.86 (16.6 - 40.06) | 1.19 (1.1 - 1.27) |
| Ireland | 2.61 (1.81 - 3.48) | 0.4 (0.24 - 0.61) | 5.45 (3.94 - 6.99) | 0.43 (0.26 - 0.62) | 0.33 (0.27 - 0.39) | 1392.29 (1214.36 - 1599.35) | 206.56 (180.33 - 236.87) | 3231.55 (2799.16 - 3873.78) | 248.59 (215.59 - 297.57) | 0.81 (0.61 - 1.02) | 25.52 (23.43 - 27.99) | 3.66 (3.05 - 4.37) | 43.08 (36.14 - 49.34) | 3.24 (2.52 - 4.04) | 0 (-0.16 - 0.15) | 940.64 (820.78 - 1055.49) | 139.67 (115.1 - 166.91) | 1777.19 (1476.33 - 2103.03) | 137.55 (109.8 - 169.18) | 0.29 (0.17 - 0.41) |
| Israel | 2.86 (2.04 - 3.58) | 0.36 (0.22 - 0.51) | 6.84 (4.91 - 8.74) | 0.36 (0.21 - 0.51) | 0.03 (0.01 - 0.05) | 457.17 (377.68 - 539.43) | 56.78 (46.76 - 67.43) | 1427.34 (1193.07 - 1679.64) | 72.21 (60.28 - 84.93) | 0.85 (0.81 - 0.89) | 7.34 (6.73 - 8.04) | 0.89 (0.74 - 1.07) | 20.02 (17.73 - 22.41) | 0.99 (0.8 - 1.19) | 0.48 (0.39 - 0.57) | 292.47 (253.63 - 343.43) | 36.26 (28.6 - 45.17) | 813.1 (684.18 - 950.3) | 41.52 (32.26 - 53.03) | 0.55 (0.49 - 0.61) |
| Italy | 75.68 (60.81 - 92.51) | 0.51 (0.36 - 0.66) | 112.07 (90.03 - 136.84) | 0.52 (0.35 - 0.69) | 0.14 (0.09 - 0.19) | 19293.92 (16177.27 - 22432.44) | 127.3 (107.02 - 148.29) | 37719.43 (31961.79 - 43905.62) | 168.28 (142.83 - 196.03) | 1.14 (1.02 - 1.25) | 178.25 (166.64 - 188.13) | 1.17 (1.07 - 1.25) | 424.56 (373.73 - 461.26) | 1.87 (1.64 - 2.06) | 2.22 (1.92 - 2.52) | 9067 (7639.71 - 10652.67) | 60.25 (50.51 - 71.7) | 18552.84 (15755.74 - 21886.99) | 85.63 (72.31 - 100.28) | 1.58 (1.42 - 1.74) |
| Jamaica | 0.98 (0.71 - 1.21) | 0.33 (0.2 - 0.46) | 1.63 (1.18 - 2.06) | 0.31 (0.18 - 0.44) | -0.22 (-0.25 - -0.18) | 44.78 (36.1 - 54.62) | 15.41 (12.14 - 19) | 94.98 (77.48 - 114.58) | 17.98 (14.5 - 21.91) | 0.52 (0.51 - 0.54) | 0.76 (0.7 - 0.83) | 0.26 (0.22 - 0.31) | 2.47 (1.86 - 3.19) | 0.47 (0.33 - 0.63) | 2.27 (1.97 - 2.56) | 31.4 (26.77 - 37.15) | 10.95 (9.07 - 13.11) | 89.3 (70.85 - 111.36) | 16.88 (13.04 - 21.58) | 1.65 (1.44 - 1.86) |
| Japan | 83.48 (58.44 - 108.37) | 0.28 (0.16 - 0.41) | 142.39 (103.66 - 181.66) | 0.28 (0.16 - 0.41) | 0.01 (-0.01 - 0.04) | 5388.8 (4401.51 - 6523.42) | 18.13 (14.6 - 22.06) | 10571.25 (8686.04 - 12721.39) | 20.56 (16.75 - 24.75) | 0.39 (0.32 - 0.45) | 33.23 (31.23 - 34.62) | 0.11 (0.1 - 0.12) | 65.75 (56.62 - 71.05) | 0.12 (0.1 - 0.13) | 0.25 (0.03 - 0.47) | 2306.59 (1819.51 - 2922.64) | 7.74 (6.09 - 9.88) | 4275.23 (3316.06 - 5490.16) | 8.51 (6.64 - 10.92) | 0.33 (0.24 - 0.41) |
| Jordan | 0.89 (0.64 - 1.16) | 0.4 (0.25 - 0.55) | 5.55 (4.02 - 7.17) | 0.42 (0.26 - 0.57) | 0.17 (0.16 - 0.19) | 216.16 (188.48 - 247.45) | 100.11 (87.2 - 114.69) | 1500 (1246.5 - 1781.71) | 119.01 (98.82 - 141.08) | 0.5 (0.44 - 0.56) | 1.39 (0.84 - 2.1) | 0.66 (0.32 - 1.21) | 7.84 (5.53 - 10.61) | 0.67 (0.36 - 1.13) | -0.08 (-0.46 - 0.31) | 89.77 (68.31 - 116.67) | 40.28 (26.68 - 56.66) | 568.78 (436.53 - 720.78) | 44.2 (29.98 - 61.03) | 0.2 (0.01 - 0.38) |
| Kazakhstan | 32.01 (27.89 - 36.39) | 1.5 (1.21 - 1.8) | 54.03 (45.22 - 62.83) | 1.5 (1.18 - 1.86) | 0.08 (-0.04 - 0.19) | 3966.83 (3493.76 - 4470.87) | 186.8 (164.53 - 210.69) | 6868.72 (6170.17 - 7726.28) | 212.15 (190.1 - 238.88) | 0.54 (0.44 - 0.64) | 37.64 (30.63 - 45.4) | 2.17 (1.72 - 2.69) | 24.58 (15.82 - 32.22) | 1.1 (0.72 - 1.49) | -2.13 (-2.46 - -1.8) | 1610.36 (1291.57 - 1929.27) | 79.45 (59.67 - 101.32) | 2107.8 (1582.04 - 2708.7) | 68.01 (47.22 - 92.24) | -0.4 (-0.48 - -0.31) |
| Kenya | 3.07 (2.08 - 4.2) | 0.23 (0.13 - 0.35) | 9.5 (6.6 - 12.64) | 0.25 (0.15 - 0.38) | 0.45 (0.39 - 0.5) | 98.54 (76.7 - 122.17) | 7.07 (5.39 - 8.97) | 329.61 (258.11 - 405.06) | 8.38 (6.51 - 10.44) | 0.49 (0.43 - 0.54) | 0.49 (0.18 - 0.73) | 0.04 (0.01 - 0.06) | 2.67 (1.67 - 3.8) | 0.08 (0.05 - 0.11) | 2.28 (2.23 - 2.33) | 39.53 (26.09 - 54.11) | 2.86 (1.86 - 3.97) | 159.48 (120.21 - 208.51) | 4.11 (3.02 - 5.4) | 1.15 (1.12 - 1.18) |
| Kiribati | 0.02 (0.02 - 0.03) | 0.37 (0.27 - 0.46) | 0.04 (0.03 - 0.06) | 0.37 (0.27 - 0.46) | -0.01 (-0.08 - 0.06) | 0.11 (0.08 - 0.15) | 1.81 (1.22 - 2.52) | 0.29 (0.21 - 0.38) | 2.13 (1.5 - 2.89) | 0.49 (0.44 - 0.55) | 0 (0 - 0) | 0 (0 - 0) | 0 (0 - 0) | 0 (0 - 0) | 0.72 (0.66 - 0.79) | 0.03 (0.02 - 0.05) | 0.51 (0.3 - 0.8) | 0.08 (0.05 - 0.12) | 0.6 (0.37 - 0.92) | 0.49 (0.44 - 0.55) |
| Kuwait | 0.39 (0.28 - 0.5) | 0.41 (0.26 - 0.55) | 2.17 (1.6 - 2.83) | 0.44 (0.28 - 0.58) | 0.21 (0.19 - 0.23) | 63.01 (51.68 - 75.26) | 69.45 (57.06 - 82.93) | 546.36 (458.18 - 643.34) | 116.78 (98.18 - 137.59) | 1.74 (1.59 - 1.89) | 0 (0 - 0) | 0 (0 - 0) | 0.79 (0.64 - 0.95) | 0.18 (0.13 - 0.23) | 14.95 (10.55 - 19.52) | 15.76 (10.44 - 22.51) | 17.19 (9.56 - 26.92) | 150.91 (106.35 - 201.93) | 31.71 (19.63 - 46.47) | 2.15 (1.84 - 2.45) |
| Kyrgyzstan | 2.75 (2.15 - 3.39) | 0.51 (0.34 - 0.68) | 4.24 (3.21 - 5.32) | 0.44 (0.29 - 0.6) | -0.56 (-0.59 - -0.53) | 250.04 (200.65 - 309.93) | 47.94 (38.35 - 59.51) | 439.36 (353.34 - 536.64) | 50.95 (40.86 - 62.34) | 0.22 (0.13 - 0.32) | 2.31 (1.96 - 2.68) | 0.54 (0.43 - 0.65) | 2.97 (2.34 - 3.82) | 0.51 (0.39 - 0.66) | 1.76 (0.28 - 3.27) | 102.43 (79.53 - 131.83) | 20.49 (14.22 - 28.33) | 160.72 (120.15 - 210.99) | 20.39 (14.04 - 28.6) | 0.7 (0.15 - 1.26) |
| Lao People's Democratic Republic | 1.27 (0.98 - 1.54) | 0.38 (0.27 - 0.47) | 2.68 (2.06 - 3.32) | 0.36 (0.25 - 0.46) | -0.18 (-0.19 - -0.17) | 8.5 (6.24 - 11.42) | 2.33 (1.6 - 3.26) | 24.18 (17.88 - 31.77) | 2.95 (2.08 - 4.05) | 0.75 (0.68 - 0.82) | 0.04 (0.01 - 0.08) | 0.01 (0 - 0.03) | 0.18 (0.07 - 0.32) | 0.03 (0.01 - 0.06) | 2.24 (1.96 - 2.51) | 3.33 (2.11 - 5.06) | 0.93 (0.54 - 1.51) | 11.1 (7.39 - 15.81) | 1.38 (0.82 - 2.28) | 1.26 (1.2 - 1.32) |
| Latvia | 1.93 (1.47 - 2.44) | 0.31 (0.2 - 0.44) | 0.99 (0.68 - 1.34) | 0.17 (0.1 - 0.26) | -2.08 (-2.4 - -1.75) | 143.51 (119.51 - 172.4) | 22.77 (18.66 - 27.63) | 166.98 (143.39 - 192.26) | 27.36 (23.33 - 31.64) | 0.67 (0.57 - 0.78) | 13.63 (12.13 - 15.09) | 2.19 (1.82 - 2.62) | 8.78 (7.3 - 10.26) | 1.34 (1.04 - 1.65) | -2.36 (-2.72 - -2) | 383.07 (345.25 - 421.15) | 60.88 (51.32 - 71.93) | 248.11 (209.79 - 286.4) | 40.63 (32.39 - 49.57) | -2.03 (-2.34 - -1.71) |
| Lebanon | 1.63 (1.21 - 2.06) | 0.42 (0.27 - 0.57) | 4.32 (3.31 - 5.41) | 0.44 (0.28 - 0.61) | 0.18 (0.16 - 0.19) | 301.56 (250.71 - 355.57) | 78.96 (65.5 - 93.57) | 1288.45 (1079.22 - 1537.42) | 132.02 (110.7 - 157.52) | 1.73 (1.7 - 1.77) | 1.06 (0.49 - 1.85) | 0.29 (0.1 - 0.67) | 3.85 (2.54 - 5.44) | 0.38 (0.17 - 0.72) | 1.36 (1.17 - 1.56) | 100.73 (71.51 - 139.56) | 25.92 (15.56 - 40.04) | 387.17 (290.03 - 502.21) | 39.77 (25.63 - 57.24) | 1.55 (1.47 - 1.62) |
| Lesotho | 0.47 (0.33 - 0.59) | 0.33 (0.2 - 0.47) | 0.64 (0.47 - 0.78) | 0.35 (0.22 - 0.48) | 0.29 (0.25 - 0.34) | 16.47 (12.82 - 20.51) | 11.43 (8.74 - 14.52) | 24.12 (19.11 - 29.48) | 12.73 (9.88 - 15.78) | 0.26 (0.2 - 0.33) | 0.05 (0.01 - 0.09) | 0.03 (0.01 - 0.08) | 0.12 (0.07 - 0.2) | 0.07 (0.03 - 0.15) | 2.57 (2.25 - 2.88) | 5.87 (3.94 - 8.47) | 4.07 (2.68 - 6.08) | 9.95 (7.28 - 13.72) | 5.23 (3.57 - 7.62) | 0.88 (0.83 - 0.93) |
| Liberia | 0.6 (0.42 - 0.76) | 0.32 (0.19 - 0.44) | 1 (0.72 - 1.3) | 0.3 (0.18 - 0.43) | -0.23 (-0.24 - -0.22) | 17.94 (14.1 - 22.63) | 9.2 (7.03 - 11.74) | 38.29 (30.15 - 47.09) | 10.81 (8.42 - 13.46) | 0.52 (0.51 - 0.54) | 0 (0 - 0) | 0 (0 - 0) | 0 (0 - 0) | 0 (0 - 0) | 1.07 (0.97 - 1.17) | 5.07 (3.35 - 7.43) | 2.6 (1.69 - 3.82) | 10.82 (7.22 - 15.37) | 3.06 (2.03 - 4.38) | 0.53 (0.51 - 0.54) |
| Libya | 1.25 (0.92 - 1.56) | 0.39 (0.25 - 0.54) | 3.6 (2.6 - 4.58) | 0.41 (0.25 - 0.56) | 0.15 (0.14 - 0.16) | 202.29 (166.96 - 238.68) | 64.56 (53.22 - 76.46) | 872.51 (741.77 - 1029.37) | 103.43 (87.56 - 122.16) | 1.69 (1.59 - 1.79) | 0.9 (0.46 - 1.46) | 0.3 (0.11 - 0.67) | 9.91 (5.88 - 16.03) | 1.23 (0.51 - 2.62) | 4.9 (4.68 - 5.11) | 71.51 (51.9 - 97.57) | 22.73 (13.78 - 34.77) | 463.21 (323.29 - 649.13) | 53.65 (32.56 - 87.85) | 3.05 (2.95 - 3.15) |
| Lithuania | 2.65 (2.03 - 3.31) | 0.33 (0.21 - 0.45) | 1.62 (1.16 - 2.16) | 0.18 (0.11 - 0.27) | -1.93 (-2.22 - -1.63) | 190.29 (159.63 - 223.18) | 23.61 (19.44 - 28.07) | 252.57 (219.73 - 287.86) | 27.82 (23.96 - 31.84) | 0.58 (0.53 - 0.64) | 16.14 (14.07 - 18.31) | 2.02 (1.68 - 2.41) | 13.46 (11.13 - 15.7) | 1.37 (1.1 - 1.68) | -1.78 (-2.1 - -1.46) | 462.81 (405.39 - 525.11) | 57.27 (48.57 - 67.04) | 381.75 (318.92 - 446.26) | 41.72 (33.63 - 50.18) | -1.49 (-1.76 - -1.22) |
| Luxembourg | 0.48 (0.38 - 0.61) | 0.53 (0.35 - 0.7) | 0.92 (0.71 - 1.22) | 0.52 (0.34 - 0.72) | -0.09 (-0.1 - -0.08) | 128.51 (110.98 - 149.65) | 138.58 (119.61 - 160.49) | 306.22 (261.24 - 350.2) | 173.66 (148.34 - 199.29) | 0.71 (0.64 - 0.78) | 2.65 (2.41 - 2.9) | 2.82 (2.32 - 3.41) | 4.7 (4.08 - 5.33) | 2.64 (2.1 - 3.27) | 0.31 (0.12 - 0.5) | 95.04 (84.14 - 107.23) | 102.71 (83.77 - 123.77) | 183.71 (157.67 - 216.08) | 104.93 (83.75 - 129.91) | 0.39 (0.26 - 0.52) |
| Madagascar | 2.44 (1.68 - 3.21) | 0.29 (0.17 - 0.42) | 5.04 (3.39 - 6.77) | 0.27 (0.16 - 0.4) | -0.25 (-0.28 - -0.21) | 84.18 (65.94 - 104.38) | 9.32 (7.06 - 11.82) | 202.14 (159.41 - 247.08) | 9.97 (7.67 - 12.48) | 0.13 (0.08 - 0.19) | 0.42 (0.08 - 0.82) | 0.05 (0.01 - 0.13) | 1.24 (0.29 - 2.51) | 0.07 (0.01 - 0.19) | 1.01 (0.9 - 1.12) | 34.3 (20.83 - 49.62) | 3.83 (2.23 - 6.05) | 89.64 (54.6 - 131.27) | 4.49 (2.6 - 7.4) | 0.43 (0.35 - 0.5) |
| Malawi | 1.75 (1.17 - 2.31) | 0.28 (0.16 - 0.41) | 3.12 (2.16 - 4.1) | 0.27 (0.15 - 0.4) | -0.1 (-0.12 - -0.09) | 53.32 (40.93 - 66.65) | 7.91 (5.95 - 10.17) | 111.11 (87.11 - 136.61) | 9.03 (6.92 - 11.3) | 0.41 (0.35 - 0.47) | 0.25 (0.04 - 0.47) | 0.04 (0 - 0.11) | 0.91 (0.21 - 1.64) | 0.08 (0.01 - 0.22) | 2.1 (1.99 - 2.22) | 21.29 (12.83 - 31.82) | 3.19 (1.87 - 5.03) | 53.91 (30.38 - 78.73) | 4.43 (2.41 - 7.65) | 1.01 (0.96 - 1.07) |
| Malaysia | 4.02 (2.91 - 5.34) | 0.28 (0.17 - 0.4) | 12.66 (9.04 - 16.28) | 0.27 (0.17 - 0.38) | -0.16 (-0.19 - -0.14) | 37.91 (28.52 - 48.53) | 2.49 (1.72 - 3.43) | 156.07 (119.34 - 197.2) | 3.09 (2.22 - 4.14) | 0.71 (0.69 - 0.72) | 0.51 (0.22 - 0.9) | 0.03 (0.01 - 0.09) | 2.66 (1.77 - 3.84) | 0.05 (0.02 - 0.11) | 1.48 (1.1 - 1.86) | 23.68 (15 - 36.2) | 1.55 (0.83 - 2.92) | 112.81 (84.01 - 151.91) | 2.22 (1.35 - 3.81) | 1.18 (0.99 - 1.38) |
| Maldives | 0.04 (0.03 - 0.05) | 0.27 (0.17 - 0.4) | 0.13 (0.09 - 0.17) | 0.24 (0.15 - 0.35) | -0.42 (-0.46 - -0.37) | 0.4 (0.29 - 0.52) | 2.33 (1.59 - 3.24) | 1.76 (1.37 - 2.22) | 3.09 (2.24 - 4.06) | 0.93 (0.91 - 0.96) | 0 (0 - 0) | 0.01 (0 - 0.02) | 0.01 (0 - 0.01) | 0.02 (0.01 - 0.04) | 1.46 (0.98 - 1.95) | 0.14 (0.1 - 0.21) | 0.85 (0.53 - 1.31) | 0.67 (0.47 - 0.91) | 1.2 (0.77 - 1.78) | 1.02 (0.86 - 1.18) |
| Mali | 2.46 (1.81 - 3.05) | 0.36 (0.23 - 0.48) | 5.05 (3.66 - 6.31) | 0.34 (0.21 - 0.47) | -0.17 (-0.19 - -0.16) | 89.63 (70.27 - 109.88) | 12.34 (9.53 - 15.36) | 210.04 (169.98 - 253.32) | 13.52 (10.76 - 16.57) | 0.27 (0.25 - 0.29) | 0 (0 - 0.01) | 0 (0 - 0) | 0.01 (0 - 0.02) | 0 (0 - 0) | 0.8 (0.66 - 0.94) | 25.29 (16.69 - 36.68) | 3.48 (2.27 - 5.07) | 59.3 (40.95 - 84.03) | 3.82 (2.57 - 5.45) | 0.27 (0.25 - 0.29) |
| Malta | 0.3 (0.24 - 0.37) | 0.42 (0.28 - 0.55) | 0.59 (0.45 - 0.73) | 0.39 (0.25 - 0.53) | -0.29 (-0.32 - -0.26) | 25.52 (21.73 - 29.77) | 35.16 (29.69 - 41.03) | 78.93 (66.41 - 93.07) | 51.69 (43.4 - 60.81) | 1.23 (1.1 - 1.37) | 0.79 (0.72 - 0.87) | 1.08 (0.89 - 1.31) | 1.88 (1.61 - 2.17) | 1.17 (0.92 - 1.46) | 0.44 (0.32 - 0.57) | 26.07 (23.2 - 29.65) | 35.64 (29.3 - 43.05) | 63.21 (54.32 - 72.61) | 41.86 (33.38 - 52.18) | 0.69 (0.59 - 0.78) |
| Marshall Islands | 0.01 (0.01 - 0.01) | 0.32 (0.21 - 0.44) | 0.02 (0.01 - 0.02) | 0.31 (0.21 - 0.43) | -0.16 (-0.2 - -0.12) | 0.05 (0.04 - 0.07) | 1.94 (1.28 - 2.77) | 0.14 (0.1 - 0.18) | 2.19 (1.52 - 3.05) | 0.36 (0.31 - 0.41) | 0 (0 - 0) | 0 (0 - 0) | 0 (0 - 0) | 0 (0 - 0) | 0.48 (0.39 - 0.56) | 0.01 (0.01 - 0.02) | 0.55 (0.32 - 0.86) | 0.04 (0.03 - 0.06) | 0.62 (0.37 - 0.96) | 0.36 (0.31 - 0.41) |
| Mauritania | 0.65 (0.49 - 0.78) | 0.39 (0.26 - 0.5) | 1.28 (0.94 - 1.58) | 0.35 (0.22 - 0.48) | -0.28 (-0.29 - -0.27) | 25.83 (20.26 - 31.27) | 14.91 (11.59 - 18.4) | 63.71 (52.39 - 75.59) | 17.02 (13.83 - 20.5) | 0.39 (0.35 - 0.42) | 0 (0 - 0) | 0 (0 - 0) | 0 (0 - 0.01) | 0 (0 - 0) | 0.86 (0.68 - 1.04) | 7.26 (4.74 - 10.31) | 4.2 (2.75 - 6.14) | 17.77 (11.97 - 25) | 4.75 (3.11 - 6.83) | 0.38 (0.35 - 0.41) |
| Mauritius | 0.4 (0.29 - 0.5) | 0.33 (0.21 - 0.45) | 1.05 (0.79 - 1.33) | 0.32 (0.21 - 0.43) | -0.14 (-0.17 - -0.11) | 4.46 (3.31 - 5.71) | 3.53 (2.5 - 4.73) | 14.21 (10.87 - 17.85) | 4.1 (3.01 - 5.39) | 0.39 (0.34 - 0.44) | 0 (0 - 0) | 0 (0 - 0) | 0.43 (0.39 - 0.47) | 0.13 (0.11 - 0.16) | 18.64 (15.26 - 22.12) | 1.27 (0.83 - 1.89) | 1.01 (0.64 - 1.52) | 14.75 (12.79 - 17.01) | 4.28 (3.52 - 5.17) | 5.86 (5.22 - 6.5) |
| Mexico | 26.77 (20.79 - 32.33) | 0.39 (0.26 - 0.49) | 88.15 (70.09 - 105.71) | 0.41 (0.29 - 0.51) | 0.24 (0.2 - 0.27) | 1136.72 (904.78 - 1388.47) | 15.94 (12.62 - 19.47) | 5842.47 (4882.61 - 6891.73) | 26.71 (22.07 - 31.56) | 1.77 (1.64 - 1.9) | 23.1 (22.14 - 24.03) | 0.35 (0.32 - 0.37) | 174.78 (151.05 - 200.76) | 0.82 (0.7 - 0.95) | 2.8 (2.29 - 3.32) | 869.74 (762.02 - 1009.88) | 12.29 (10.75 - 14.31) | 5859.3 (5053.54 - 6767.74) | 26.72 (22.86 - 30.98) | 2.51 (2.1 - 2.92) |
| Micronesia (Federated States of) | 0.02 (0.02 - 0.03) | 0.31 (0.2 - 0.43) | 0.04 (0.03 - 0.05) | 0.3 (0.2 - 0.42) | -0.1 (-0.14 - -0.06) | 0.15 (0.11 - 0.2) | 1.83 (1.2 - 2.61) | 0.31 (0.23 - 0.42) | 2.2 (1.51 - 3.07) | 0.6 (0.58 - 0.61) | 0 (0 - 0) | 0 (0 - 0) | 0 (0 - 0) | 0 (0 - 0) | 1.02 (0.9 - 1.15) | 0.04 (0.03 - 0.07) | 0.52 (0.3 - 0.82) | 0.09 (0.06 - 0.14) | 0.62 (0.37 - 0.97) | 0.6 (0.58 - 0.61) |
| Monaco | 0.05 (0.04 - 0.06) | 0.44 (0.28 - 0.59) | 0.07 (0.05 - 0.08) | 0.45 (0.29 - 0.6) | 0.04 (0.03 - 0.05) | 9.48 (7.89 - 11.19) | 87.48 (72.94 - 103.18) | 17.05 (14.5 - 19.97) | 110.74 (94.18 - 129.19) | 0.82 (0.77 - 0.87) | 0.08 (0.05 - 0.13) | 0.71 (0.25 - 1.62) | 0.18 (0.1 - 0.31) | 1.11 (0.37 - 2.65) | 1.74 (1.36 - 2.11) | 4.12 (3.01 - 5.55) | 38.67 (23.22 - 62.8) | 8.12 (5.8 - 11.26) | 53.75 (31.54 - 90.01) | 1.23 (1.03 - 1.44) |
| Mongolia | 1.01 (0.79 - 1.24) | 0.55 (0.37 - 0.72) | 2.12 (1.59 - 2.65) | 0.45 (0.29 - 0.61) | -0.7 (-0.73 - -0.66) | 81.28 (64.33 - 100.56) | 46 (36.21 - 57.22) | 200.76 (161.78 - 242.69) | 49.52 (39.72 - 60.31) | 0.34 (0.21 - 0.47) | 1.72 (0.58 - 3.57) | 1.36 (0.4 - 3.32) | 3.91 (2.01 - 6.89) | 1.64 (0.74 - 3.23) | 0.54 (0.37 - 0.71) | 49.45 (29.57 - 78.44) | 31.61 (15.61 - 60.23) | 117.94 (78.48 - 172.69) | 35.94 (20.43 - 60.25) | 0.45 (0.32 - 0.58) |
| Montenegro | 0.63 (0.49 - 0.79) | 0.55 (0.38 - 0.73) | 0.65 (0.43 - 0.9) | 0.39 (0.22 - 0.6) | -1.1 (-1.15 - -1.05) | 88.17 (79.67 - 96.33) | 78.75 (70.58 - 86.59) | 169.03 (155.06 - 182.47) | 95.74 (87.77 - 103.8) | 0.68 (0.61 - 0.75) | 2.05 (1.2 - 3.22) | 1.86 (0.91 - 3.42) | 3.68 (2.44 - 5.16) | 2.14 (1.15 - 3.6) | 0.53 (0.4 - 0.66) | 75.38 (52.53 - 107.36) | 66.65 (41.03 - 105.64) | 131.02 (99.17 - 171.89) | 74.74 (49.07 - 110.37) | 0.48 (0.42 - 0.55) |
| Morocco | 9.62 (7.11 - 11.98) | 0.41 (0.26 - 0.55) | 26.29 (19.23 - 33.27) | 0.43 (0.27 - 0.58) | 0.2 (0.18 - 0.21) | 1495.58 (1234.29 - 1784.75) | 63.19 (51.98 - 75.75) | 6274.41 (5256.59 - 7389.48) | 103.58 (86.84 - 122.08) | 1.64 (1.62 - 1.66) | 4.88 (1.4 - 8.79) | 0.22 (0.04 - 0.54) | 36.49 (19.71 - 58.85) | 0.63 (0.24 - 1.36) | 3.5 (3.22 - 3.77) | 485.77 (318.42 - 682.4) | 20.54 (11.62 - 31.74) | 2414.84 (1789.63 - 3243.65) | 39.42 (25.27 - 59.15) | 2.2 (2.12 - 2.28) |
| Mozambique | 2.96 (2.08 - 3.89) | 0.3 (0.18 - 0.44) | 5.55 (3.96 - 7.24) | 0.31 (0.19 - 0.44) | 0.15 (0.12 - 0.18) | 90.61 (69.46 - 114.73) | 8.65 (6.53 - 11.11) | 191.44 (151.07 - 237.94) | 10.02 (7.69 - 12.66) | 0.4 (0.37 - 0.44) | 0.38 (0.04 - 0.78) | 0.04 (0 - 0.11) | 1.36 (0.24 - 2.81) | 0.08 (0.01 - 0.22) | 2.41 (2.33 - 2.49) | 35.13 (20.53 - 52.81) | 3.39 (1.96 - 5.4) | 88.59 (49.15 - 135.43) | 4.7 (2.53 - 8.15) | 1.09 (1.06 - 1.12) |
| Myanmar | 14.61 (11.1 - 17.69) | 0.38 (0.27 - 0.47) | 30.23 (23.73 - 36.97) | 0.37 (0.27 - 0.46) | -0.12 (-0.13 - -0.12) | 108.83 (79.05 - 144.3) | 2.61 (1.8 - 3.6) | 300.81 (226.66 - 385.8) | 3.41 (2.44 - 4.55) | 0.89 (0.81 - 0.97) | 0.51 (0.14 - 1.06) | 0.01 (0 - 0.04) | 2.23 (0.99 - 3.8) | 0.03 (0.01 - 0.07) | 1.98 (1.7 - 2.26) | 43.18 (27.69 - 64.33) | 1.05 (0.63 - 1.71) | 138.18 (92.88 - 203.11) | 1.59 (0.97 - 2.59) | 1.27 (1.2 - 1.34) |
| Namibia | 0.32 (0.23 - 0.43) | 0.3 (0.18 - 0.44) | 0.67 (0.47 - 0.88) | 0.3 (0.17 - 0.43) | 0.04 (0 - 0.07) | 11.65 (9.15 - 14.43) | 10.16 (7.77 - 12.83) | 28.29 (22.79 - 34.06) | 11.99 (9.44 - 14.71) | 0.55 (0.47 - 0.62) | 0.04 (0.02 - 0.07) | 0.04 (0.01 - 0.09) | 0.15 (0.09 - 0.23) | 0.07 (0.03 - 0.13) | 1.5 (1.36 - 1.64) | 4.44 (3.15 - 6.11) | 3.87 (2.59 - 5.57) | 11.81 (8.59 - 15.6) | 4.99 (3.41 - 7.11) | 0.83 (0.79 - 0.86) |
| Nauru | 0 (0 - 0) | 0.3 (0.19 - 0.42) | 0 (0 - 0) | 0.28 (0.18 - 0.39) | -0.23 (-0.3 - -0.16) | 0.01 (0.01 - 0.02) | 1.64 (1.08 - 2.35) | 0.02 (0.01 - 0.03) | 1.93 (1.31 - 2.74) | 0.48 (0.42 - 0.55) | 0 (0 - 0) | 0 (0 - 0) | 0 (0 - 0) | 0 (0 - 0) | 1.3 (1.1 - 1.51) | 0 (0 - 0.01) | 0.46 (0.27 - 0.73) | 0.01 (0 - 0.01) | 0.55 (0.33 - 0.85) | 0.49 (0.42 - 0.56) |
| Nepal | 4.8 (3.39 - 6.21) | 0.31 (0.19 - 0.44) | 12.76 (9.18 - 16.39) | 0.32 (0.2 - 0.46) | 0.22 (0.18 - 0.26) | 199.17 (159.32 - 245.32) | 12.05 (9.41 - 14.98) | 647.62 (516.93 - 785.2) | 15.81 (12.51 - 19.29) | 0.97 (0.93 - 1.01) | 0.38 (0.06 - 0.8) | 0.03 (0 - 0.07) | 1.87 (0.5 - 3.43) | 0.05 (0.01 - 0.12) | 2.34 (2.14 - 2.55) | 65.9 (44.77 - 93.81) | 4 (2.66 - 5.89) | 227.82 (156.24 - 314.15) | 5.58 (3.71 - 8.02) | 1.19 (1.13 - 1.25) |
| Netherlands | 16.94 (13.28 - 21.6) | 0.53 (0.35 - 0.71) | 29.69 (23.52 - 38.35) | 0.53 (0.35 - 0.74) | 0.01 (0 - 0.02) | 4919.31 (4209.51 - 5654.09) | 150.88 (129.49 - 173.67) | 10706.86 (9267.11 - 12543.16) | 186.31 (161.35 - 217.67) | 0.74 (0.66 - 0.81) | 107.85 (100.1 - 117.23) | 3.21 (2.73 - 3.79) | 208.41 (184.1 - 230.59) | 3.48 (2.81 - 4.18) | 0.37 (0.18 - 0.55) | 3754.67 (3337.38 - 4247.93) | 115.27 (95.37 - 138.66) | 7262.48 (6265.85 - 8257.44) | 126.84 (103.48 - 152.05) | 0.45 (0.34 - 0.55) |
| New Zealand | 2.82 (2.09 - 3.49) | 0.44 (0.28 - 0.61) | 5.76 (4.32 - 7.03) | 0.41 (0.26 - 0.56) | -0.25 (-0.28 - -0.22) | 494.17 (416.59 - 576.4) | 75.87 (63.66 - 88.88) | 938.07 (816.78 - 1069.71) | 67.21 (58.04 - 76.74) | -0.43 (-0.47 - -0.38) | 14.42 (12.93 - 16.12) | 2.15 (1.76 - 2.59) | 29.9 (25.18 - 34.9) | 2.05 (1.61 - 2.55) | -0.55 (-0.96 - -0.14) | 458.63 (401.66 - 520.35) | 70.08 (57.62 - 84.87) | 900.34 (770.17 - 1041.09) | 63.98 (51.41 - 78.17) | -0.62 (-0.93 - -0.31) |
| Nicaragua | 0.91 (0.7 - 1.11) | 0.37 (0.25 - 0.47) | 3.38 (2.72 - 4.1) | 0.42 (0.3 - 0.52) | 0.42 (0.35 - 0.5) | 32.21 (25.81 - 39.13) | 12.76 (10.13 - 15.64) | 195.69 (162.45 - 232.34) | 23.81 (19.71 - 28.25) | 2.23 (2.06 - 2.4) | 0.35 (0.23 - 0.48) | 0.14 (0.07 - 0.24) | 2.49 (1.66 - 3.69) | 0.31 (0.14 - 0.59) | 3.01 (2.44 - 3.6) | 18.04 (14 - 23.16) | 7.08 (5.02 - 9.96) | 115.19 (86.55 - 151.05) | 13.9 (8.65 - 21.39) | 2.57 (2.18 - 2.96) |
| Niger | 1.58 (1.17 - 1.97) | 0.36 (0.23 - 0.48) | 4.82 (3.54 - 6.04) | 0.34 (0.21 - 0.47) | -0.17 (-0.18 - -0.15) | 54.64 (42.26 - 67.83) | 11.48 (8.82 - 14.51) | 193.89 (153.55 - 233.09) | 12.83 (10 - 15.8) | 0.4 (0.37 - 0.42) | 0 (0 - 0) | 0 (0 - 0) | 0.01 (0 - 0.01) | 0 (0 - 0) | 0.9 (0.78 - 1.01) | 15.41 (10.02 - 21.91) | 3.24 (2.11 - 4.71) | 54.69 (37.04 - 77.15) | 3.62 (2.42 - 5.21) | 0.4 (0.37 - 0.42) |
| Nigeria | 22.81 (16.33 - 28.66) | 0.31 (0.19 - 0.43) | 50.95 (37.13 - 63.62) | 0.33 (0.21 - 0.45) | 0.32 (0.28 - 0.36) | 751.66 (592.5 - 929.08) | 9.92 (7.66 - 12.4) | 2135.35 (1713.64 - 2589.41) | 13.4 (10.64 - 16.35) | 1.08 (0.99 - 1.16) | 0.03 (0.01 - 0.05) | 0 (0 - 0) | 0.11 (0.06 - 0.15) | 0 (0 - 0) | 2.02 (1.8 - 2.24) | 212.25 (143.01 - 301.99) | 2.8 (1.85 - 4.03) | 603.34 (414.49 - 858.79) | 3.79 (2.56 - 5.4) | 1.08 (1 - 1.17) |
| Niue | 0 (0 - 0) | 0.34 (0.23 - 0.45) | 0 (0 - 0) | 0.32 (0.22 - 0.43) | -0.21 (-0.23 - -0.18) | 0.01 (0.01 - 0.01) | 3.01 (2.09 - 4.12) | 0.01 (0.01 - 0.02) | 3.42 (2.47 - 4.59) | 0.41 (0.4 - 0.42) | 0 (0 - 0) | 0 (0 - 0) | 0 (0 - 0) | 0 (0 - 0) | 0.74 (0.64 - 0.84) | 0 (0 - 0) | 0.85 (0.52 - 1.3) | 0 (0 - 0.01) | 0.97 (0.61 - 1.45) | 0.42 (0.4 - 0.43) |
| North Macedonia | 1.73 (1.36 - 2.18) | 0.51 (0.35 - 0.67) | 2.05 (1.34 - 2.74) | 0.35 (0.2 - 0.54) | -1.16 (-1.22 - -1.09) | 203.22 (176.67 - 231.03) | 59.55 (51.2 - 67.74) | 530.58 (481.25 - 580.13) | 87.25 (78.84 - 95.97) | 1.35 (1.19 - 1.51) | 5.66 (3.74 - 7.88) | 1.73 (0.97 - 2.8) | 9.63 (6.2 - 14.74) | 1.68 (0.9 - 2.85) | -0.25 (-0.39 - -0.11) | 198.51 (143.76 - 254.69) | 57.72 (38.47 - 85.58) | 366.94 (273.8 - 504.73) | 60.9 (39.68 - 90.48) | 0.11 (0.02 - 0.2) |
| Northern Mariana Islands | 0.01 (0 - 0.01) | 0.29 (0.18 - 0.41) | 0.03 (0.02 - 0.04) | 0.29 (0.19 - 0.41) | -0.01 (-0.03 - 0.01) | 0.08 (0.06 - 0.11) | 3.06 (2.15 - 4.15) | 0.37 (0.28 - 0.47) | 3.55 (2.56 - 4.72) | 0.33 (0.28 - 0.38) | 0 (0 - 0) | 0 (0 - 0) | 0 (0 - 0) | 0 (0 - 0) | 0.15 (-0.1 - 0.4) | 0.02 (0.01 - 0.03) | 0.86 (0.53 - 1.3) | 0.1 (0.07 - 0.15) | 1 (0.63 - 1.49) | 0.33 (0.28 - 0.38) |
| Norway | 6.16 (4.89 - 7.72) | 0.65 (0.44 - 0.89) | 10.19 (7.73 - 13.76) | 0.68 (0.44 - 0.99) | 0.3 (0.22 - 0.37) | 1660.37 (1417.78 - 1925.36) | 160.52 (136.88 - 186.1) | 4487.36 (3779.48 - 5224.17) | 281.66 (237.91 - 327.67) | 1.83 (1.77 - 1.89) | 46.46 (43.78 - 49.01) | 4.25 (3.91 - 4.57) | 72.77 (65.47 - 78.3) | 4.34 (3.86 - 4.78) | 0.18 (-0.14 - 0.5) | 1466.67 (1338.52 - 1610.33) | 143.61 (129.49 - 159.16) | 2689.32 (2317.82 - 3067.93) | 169.25 (144.43 - 194.21) | 0.6 (0.36 - 0.83) |
| Oman | 0.4 (0.29 - 0.5) | 0.39 (0.24 - 0.54) | 1.37 (0.97 - 1.88) | 0.41 (0.25 - 0.59) | 0.21 (0.19 - 0.23) | 61.11 (50.97 - 71.93) | 60.44 (50.27 - 71.32) | 330.48 (277.59 - 384.15) | 105.46 (88.65 - 122.97) | 1.85 (1.78 - 1.91) | 0.21 (0.12 - 0.34) | 0.22 (0.09 - 0.48) | 1.63 (0.7 - 3.42) | 0.56 (0.17 - 1.47) | 3.32 (2.73 - 3.9) | 20.62 (14.84 - 27.29) | 20.25 (12.67 - 29.75) | 122.87 (79.63 - 176.87) | 38.27 (22.15 - 61.1) | 2.24 (2 - 2.48) |
| Pakistan | 32.43 (23.82 - 40.13) | 0.35 (0.22 - 0.47) | 73.13 (54.64 - 90.29) | 0.37 (0.24 - 0.48) | 0.12 (0.08 - 0.16) | 1305.36 (1035.85 - 1588.35) | 13.87 (10.85 - 17.1) | 3508.32 (2807.12 - 4247.68) | 16.76 (13.24 - 20.42) | 0.71 (0.66 - 0.76) | 3.32 (0.76 - 5.88) | 0.04 (0.01 - 0.08) | 12.54 (5.71 - 20.19) | 0.07 (0.03 - 0.13) | 1.7 (1.54 - 1.85) | 448.25 (308.87 - 629.46) | 4.77 (3.2 - 6.78) | 1299.55 (935.03 - 1749.75) | 6.25 (4.4 - 8.69) | 0.9 (0.88 - 0.93) |
| Palau | 0 (0 - 0.01) | 0.27 (0.17 - 0.4) | 0.01 (0.01 - 0.01) | 0.27 (0.17 - 0.38) | -0.11 (-0.12 - -0.09) | 0.03 (0.03 - 0.05) | 2.13 (1.44 - 2.99) | 0.11 (0.08 - 0.14) | 2.45 (1.7 - 3.37) | 0.4 (0.39 - 0.42) | 0 (0 - 0) | 0 (0 - 0) | 0 (0 - 0) | 0 (0 - 0) | 0.8 (0.65 - 0.94) | 0.01 (0.01 - 0.02) | 0.6 (0.36 - 0.94) | 0.03 (0.02 - 0.05) | 0.69 (0.42 - 1.06) | 0.4 (0.39 - 0.42) |
| Palestine | 0.6 (0.44 - 0.74) | 0.41 (0.26 - 0.55) | 1.9 (1.36 - 2.46) | 0.42 (0.26 - 0.57) | 0.11 (0.1 - 0.12) | 104.23 (85.76 - 122.52) | 71.31 (58.45 - 84.01) | 452.28 (376.3 - 535.4) | 104.94 (87.28 - 124.51) | 1.29 (1.28 - 1.31) | 0.71 (0.34 - 1.21) | 0.53 (0.17 - 1.22) | 4.2 (3.06 - 5.71) | 1.05 (0.51 - 1.93) | 3.46 (2.93 - 3.99) | 42 (29.05 - 58.86) | 28.8 (17.08 - 46.94) | 217.06 (166.6 - 275.09) | 49.24 (32.17 - 72.13) | 2.4 (2.13 - 2.66) |
| Panama | 0.71 (0.51 - 0.91) | 0.29 (0.17 - 0.42) | 2.1 (1.52 - 2.66) | 0.28 (0.17 - 0.41) | -0.12 (-0.15 - -0.1) | 26.23 (21.32 - 32.15) | 10.74 (8.44 - 13.18) | 108.3 (88.51 - 130.34) | 14.59 (11.74 - 17.77) | 0.89 (0.86 - 0.93) | 0.47 (0.42 - 0.52) | 0.19 (0.16 - 0.24) | 3.15 (2.45 - 3.94) | 0.42 (0.3 - 0.55) | 2.48 (1.96 - 3.01) | 18.65 (15.87 - 22.1) | 7.62 (6.27 - 9.21) | 105.24 (84.19 - 126.37) | 14.18 (11.01 - 17.64) | 1.99 (1.63 - 2.35) |
| Papua New Guinea | 0.75 (0.51 - 1.01) | 0.26 (0.16 - 0.38) | 1.97 (1.35 - 2.76) | 0.25 (0.15 - 0.37) | -0.19 (-0.23 - -0.16) | 4.78 (3.31 - 6.55) | 1.5 (0.95 - 2.18) | 14.54 (10.24 - 19.6) | 1.62 (1.06 - 2.34) | 0.18 (0.12 - 0.25) | 0 (0 - 0) | 0 (0 - 0) | 0 (0 - 0) | 0 (0 - 0) | 0.74 (0.67 - 0.81) | 1.35 (0.81 - 2.09) | 0.42 (0.24 - 0.67) | 4.11 (2.52 - 6.19) | 0.46 (0.26 - 0.72) | 0.18 (0.12 - 0.25) |
| Paraguay | 1.68 (1.34 - 2.01) | 0.46 (0.32 - 0.57) | 4.58 (3.66 - 5.45) | 0.46 (0.32 - 0.57) | 0.01 (-0.02 - 0.03) | 118.79 (95.08 - 143.63) | 32.57 (26.07 - 39.7) | 372.19 (306.96 - 444.8) | 37.46 (30.8 - 44.78) | 0.45 (0.38 - 0.52) | 0.69 (0.52 - 0.87) | 0.2 (0.12 - 0.3) | 4.5 (3.22 - 6.21) | 0.48 (0.26 - 0.81) | 2.35 (1.81 - 2.9) | 46.24 (34.5 - 60.59) | 12.74 (8.53 - 18.38) | 193.39 (151.79 - 246.68) | 19.57 (13.25 - 27.99) | 1.23 (1.02 - 1.44) |
| Peru | 7.36 (5.71 - 8.98) | 0.38 (0.26 - 0.48) | 22.45 (18.11 - 26.94) | 0.4 (0.29 - 0.5) | 0.25 (0.22 - 0.28) | 286.31 (228.64 - 344.45) | 14.42 (11.48 - 17.5) | 1254.2 (1013.91 - 1489.46) | 22.44 (18.07 - 26.9) | 1.57 (1.48 - 1.65) | 3.06 (2.02 - 4.09) | 0.16 (0.08 - 0.26) | 18.86 (11.1 - 29.69) | 0.34 (0.15 - 0.66) | 3.21 (2.81 - 3.6) | 152.74 (114.48 - 196.97) | 7.68 (5.43 - 10.57) | 772.95 (554.78 - 1060.91) | 13.8 (8.85 - 20.98) | 2.29 (2.09 - 2.48) |
| Philippines | 16.01 (12.16 - 20.09) | 0.35 (0.24 - 0.45) | 44.45 (34.03 - 56.12) | 0.33 (0.23 - 0.43) | -0.15 (-0.16 - -0.14) | 157.49 (118.32 - 204.01) | 3.15 (2.24 - 4.24) | 514.51 (391.21 - 654) | 3.55 (2.58 - 4.66) | 0.3 (0.22 - 0.37) | 2.81 (1.63 - 3.64) | 0.06 (0.04 - 0.09) | 11.81 (9.77 - 14.04) | 0.09 (0.07 - 0.11) | 0.95 (0.82 - 1.08) | 115.31 (79.28 - 147.13) | 2.33 (1.57 - 3.04) | 448.16 (370.82 - 534.78) | 3.08 (2.5 - 3.76) | 0.78 (0.68 - 0.88) |
| Poland | 45.59 (35.96 - 57.54) | 0.59 (0.4 - 0.79) | 35.62 (26.09 - 45.69) | 0.32 (0.19 - 0.47) | -2.26 (-2.42 - -2.11) | 6584.47 (5663.29 - 7637.48) | 84.05 (71.12 - 97.96) | 9699.04 (8856.05 - 10604.88) | 82.94 (74.94 - 91.38) | -0.1 (-0.2 - 0) | 243.34 (230.27 - 255.76) | 3.1 (2.9 - 3.3) | 292.03 (260.91 - 322.27) | 2.42 (2.14 - 2.7) | -0.85 (-1.09 - -0.62) | 8204.79 (7572.07 - 8853.01) | 103.83 (95.39 - 113.13) | 9734.6 (8719.72 - 10659.45) | 83.15 (74.1 - 92.16) | -0.73 (-0.93 - -0.53) |
| Portugal | 10.28 (7.7 - 12.45) | 0.43 (0.27 - 0.57) | 16.14 (12.44 - 19.41) | 0.43 (0.28 - 0.57) | -0.08 (-0.12 - -0.04) | 1398.38 (1180.74 - 1632.76) | 57.39 (48.02 - 67.47) | 2472.37 (2228.55 - 2717.44) | 64.91 (58.42 - 71.36) | 0.39 (0.36 - 0.42) | 25.71 (23.65 - 27.95) | 1.04 (0.88 - 1.23) | 44.09 (38.69 - 49.35) | 1.13 (0.92 - 1.36) | 0.48 (0.34 - 0.61) | 975.44 (847.3 - 1142.43) | 39.82 (32.1 - 49.15) | 1620.76 (1396.18 - 1867.7) | 43.99 (35 - 54.65) | 0.47 (0.39 - 0.55) |
| Puerto Rico | 2.15 (1.62 - 2.66) | 0.36 (0.22 - 0.48) | 3.88 (2.88 - 4.79) | 0.33 (0.2 - 0.47) | -0.17 (-0.19 - -0.16) | 119.82 (96.63 - 144) | 19.84 (15.8 - 24.04) | 315.77 (260.06 - 375.6) | 27.95 (23.07 - 33.35) | 1.16 (1.07 - 1.26) | 4.56 (4.19 - 5.01) | 0.76 (0.63 - 0.9) | 8.83 (7.17 - 10.7) | 0.78 (0.59 - 1) | 0.33 (0.08 - 0.58) | 147.35 (132.29 - 165.29) | 24.63 (20.8 - 29.11) | 295.36 (244.01 - 354.46) | 27.32 (21.29 - 34.54) | 0.54 (0.34 - 0.73) |
| Qatar | 0.07 (0.05 - 0.09) | 0.38 (0.23 - 0.53) | 0.69 (0.48 - 0.95) | 0.4 (0.25 - 0.56) | 0.22 (0.18 - 0.27) | 13.62 (11.05 - 16.21) | 86.55 (70.27 - 102.74) | 226.34 (199.58 - 256.96) | 154.82 (136.63 - 175.45) | 1.22 (1 - 1.43) | 0.01 (0 - 0.02) | 0.07 (0.02 - 0.19) | 0.18 (0.11 - 0.28) | 0.16 (0.07 - 0.31) | 3.53 (2.29 - 4.79) | 3.59 (2.44 - 5.2) | 22.38 (13.31 - 34.77) | 59.31 (42.5 - 80.36) | 39.54 (25.67 - 57.17) | 1.24 (1.01 - 1.47) |
| Republic of Korea | 14.41 (9.99 - 19.07) | 0.28 (0.16 - 0.42) | 44.19 (31.07 - 59.16) | 0.26 (0.15 - 0.4) | -0.2 (-0.24 - -0.17) | 888.72 (715.91 - 1083.69) | 17.37 (13.8 - 21.4) | 3311.21 (2691.47 - 3984.16) | 19.77 (16.03 - 23.92) | 0.43 (0.4 - 0.46) | 5.99 (4.56 - 7.57) | 0.12 (0.08 - 0.18) | 11.79 (8.35 - 15.54) | 0.07 (0.04 - 0.11) | -1.76 (-2.01 - -1.51) | 401.9 (319.04 - 519.78) | 7.81 (5.9 - 10.32) | 1203.37 (895.13 - 1613.1) | 7.18 (5.22 - 9.66) | -0.24 (-0.31 - -0.17) |
| Republic of Moldova | 1.89 (1.39 - 2.43) | 0.25 (0.16 - 0.35) | 2.02 (1.55 - 2.59) | 0.19 (0.13 - 0.27) | -0.69 (-0.89 - -0.48) | 56.54 (39.45 - 76.18) | 6.85 (4.73 - 9.33) | 131.15 (100.72 - 165.82) | 12.4 (9.41 - 15.69) | 1.83 (1.58 - 2.08) | 3.19 (2.87 - 3.53) | 0.42 (0.35 - 0.5) | 2.65 (2.17 - 3.14) | 0.25 (0.2 - 0.31) | -2.3 (-2.71 - -1.89) | 101.4 (89.91 - 113.2) | 12.5 (10.55 - 14.73) | 105.67 (88.84 - 126.83) | 10.02 (8.18 - 12.24) | -1.28 (-1.58 - -0.97) |
| Romania | 18.1 (13.63 - 22.48) | 0.36 (0.23 - 0.49) | 11.22 (7.62 - 15.13) | 0.2 (0.11 - 0.31) | -1.95 (-2.02 - -1.88) | 1143.97 (919.99 - 1366.53) | 21.78 (17.33 - 26.4) | 1389.44 (1152.89 - 1646.36) | 24.84 (20.43 - 29.6) | 0.36 (0.32 - 0.4) | 69.33 (62.7 - 76.52) | 1.38 (1.16 - 1.64) | 59.58 (50.78 - 69.82) | 1 (0.8 - 1.24) | -1.45 (-1.62 - -1.28) | 2120.27 (1910.79 - 2338.09) | 40.71 (34.52 - 47.49) | 1789.51 (1513.3 - 2050.56) | 32.01 (26.16 - 38.66) | -1.13 (-1.27 - -0.98) |
| Russian Federation | 102.37 (76.9 - 133.03) | 0.32 (0.21 - 0.47) | 19.95 (11.99 - 30.49) | 0.05 (0.02 - 0.09) | -5.66 (-6.01 - -5.31) | 12551.79 (10483.33 - 14931.41) | 38.91 (32.2 - 46.4) | 19495.66 (17867.18 - 21130.17) | 45.84 (41.9 - 49.97) | 0.55 (0.53 - 0.57) | 287.05 (274.79 - 301.41) | 0.94 (0.88 - 1) | 341.74 (308.1 - 373.8) | 0.81 (0.72 - 0.89) | -1.46 (-1.95 - -0.98) | 10522.07 (9459.87 - 11815.37) | 32.77 (29.32 - 36.96) | 13900.98 (12255.96 - 15440.66) | 32.89 (28.82 - 36.84) | -0.6 (-0.87 - -0.33) |
| Rwanda | 1.13 (0.75 - 1.53) | 0.24 (0.14 - 0.36) | 2.36 (1.55 - 3.33) | 0.22 (0.12 - 0.34) | -0.26 (-0.28 - -0.24) | 31.87 (24.27 - 41.06) | 6.28 (4.69 - 8.19) | 83.28 (63.33 - 103.27) | 7.38 (5.53 - 9.36) | 0.56 (0.52 - 0.6) | 0.21 (0.09 - 0.36) | 0.05 (0.01 - 0.11) | 0.68 (0.24 - 1.3) | 0.07 (0.02 - 0.16) | 0.87 (0.73 - 1) | 14.31 (9.39 - 20.55) | 2.87 (1.75 - 4.6) | 40.65 (25.6 - 58.11) | 3.66 (2.16 - 5.98) | 0.66 (0.6 - 0.71) |
| Saint Kitts and Nevis | 0.03 (0.02 - 0.03) | 0.44 (0.32 - 0.54) | 0.06 (0.05 - 0.08) | 0.46 (0.31 - 0.59) | 0.18 (0.14 - 0.22) | 1.4 (1.14 - 1.7) | 22.83 (18.54 - 27.53) | 4.81 (4.15 - 5.6) | 35.07 (29.79 - 40.89) | 1.52 (1.35 - 1.69) | 0.08 (0.07 - 0.09) | 1.26 (1.04 - 1.5) | 0.19 (0.15 - 0.23) | 1.46 (1.09 - 1.87) | 1.36 (1.03 - 1.69) | 2.33 (2.09 - 2.56) | 38.55 (32.45 - 45.09) | 6.35 (5.1 - 7.51) | 45.7 (35.71 - 56.67) | 1.27 (1 - 1.54) |
| Saint Lucia | 0.05 (0.04 - 0.06) | 0.34 (0.21 - 0.46) | 0.13 (0.1 - 0.17) | 0.32 (0.19 - 0.45) | -0.23 (-0.25 - -0.21) | 2.1 (1.68 - 2.57) | 14.34 (11.29 - 17.63) | 7.51 (6.05 - 9.1) | 17.68 (14.13 - 21.55) | 0.69 (0.65 - 0.74) | 0.07 (0.06 - 0.07) | 0.45 (0.38 - 0.54) | 0.22 (0.18 - 0.27) | 0.53 (0.4 - 0.68) | 0.36 (0.12 - 0.61) | 2.23 (2 - 2.52) | 15.36 (12.96 - 17.98) | 7.83 (6.44 - 9.48) | 18.23 (14.52 - 22.84) | 0.5 (0.33 - 0.67) |
| Saint Vincent and the Grenadines | 0.04 (0.03 - 0.05) | 0.32 (0.2 - 0.44) | 0.07 (0.05 - 0.1) | 0.3 (0.18 - 0.42) | -0.26 (-0.28 - -0.24) | 1.65 (1.33 - 2.03) | 13.81 (10.86 - 17.03) | 4.21 (3.41 - 5.04) | 16.35 (13.13 - 19.84) | 0.57 (0.54 - 0.6) | 0.03 (0.02 - 0.03) | 0.24 (0.18 - 0.3) | 0.07 (0.06 - 0.09) | 0.28 (0.21 - 0.38) | 0.43 (0.13 - 0.72) | 1.17 (0.98 - 1.38) | 9.82 (7.79 - 12.04) | 3.08 (2.55 - 3.73) | 11.84 (9.27 - 14.85) | 0.46 (0.28 - 0.63) |
| Samoa | 0.04 (0.03 - 0.06) | 0.31 (0.2 - 0.42) | 0.07 (0.05 - 0.09) | 0.28 (0.18 - 0.41) | -0.29 (-0.31 - -0.28) | 0.37 (0.27 - 0.49) | 2.45 (1.66 - 3.44) | 0.71 (0.54 - 0.92) | 2.78 (1.95 - 3.79) | 0.33 (0.3 - 0.37) | 0 (0 - 0) | 0 (0 - 0) | 0 (0 - 0) | 0 (0 - 0) | 0.83 (0.65 - 1.01) | 0.1 (0.07 - 0.16) | 0.69 (0.42 - 1.07) | 0.2 (0.13 - 0.3) | 0.79 (0.48 - 1.19) | 0.33 (0.3 - 0.37) |
| San Marino | 0.02 (0.02 - 0.03) | 0.43 (0.27 - 0.58) | 0.05 (0.04 - 0.06) | 0.43 (0.27 - 0.57) | -0.01 (-0.04 - 0.01) | 4.39 (3.6 - 5.23) | 75.24 (61.47 - 89.73) | 9.91 (8.11 - 11.71) | 84.46 (69.2 - 100.63) | 0.35 (0.31 - 0.39) | 0 (0 - 0) | 0 (0 - 0) | 0 (0 - 0) | 0 (0 - 0) | 0.23 (-0.13 - 0.6) | 1.09 (0.73 - 1.56) | 18.72 (9.82 - 29.63) | 2.44 (1.61 - 3.52) | 20.98 (11.43 - 34.26) | 0.36 (0.32 - 0.4) |
| Sao Tome and Principe | 0.03 (0.02 - 0.04) | 0.27 (0.15 - 0.4) | 0.05 (0.04 - 0.07) | 0.27 (0.16 - 0.4) | 0.12 (0.08 - 0.16) | 1.03 (0.81 - 1.28) | 8.92 (6.83 - 11.23) | 2.12 (1.68 - 2.57) | 10.95 (8.58 - 13.48) | 0.72 (0.68 - 0.76) | 0 (0 - 0) | 0 (0 - 0) | 0 (0 - 0) | 0 (0 - 0) | 2.16 (2.07 - 2.25) | 0.29 (0.2 - 0.42) | 2.52 (1.64 - 3.66) | 0.6 (0.41 - 0.85) | 3.08 (2.06 - 4.41) | 0.72 (0.68 - 0.76) |
| Saudi Arabia | 3.21 (2.27 - 4.06) | 0.35 (0.21 - 0.49) | 12.02 (8.46 - 15.92) | 0.37 (0.23 - 0.52) | 0.18 (0.15 - 0.2) | 413.69 (338.47 - 497.14) | 46.22 (37.63 - 55.64) | 2174.73 (1773.03 - 2620.11) | 71.69 (58.52 - 86.22) | 1.47 (1.44 - 1.49) | 0.62 (0.22 - 1.18) | 0.07 (0.02 - 0.17) | 5.63 (3.36 - 8.67) | 0.19 (0.08 - 0.38) | 3.77 (3.28 - 4.27) | 123.56 (84.13 - 171.05) | 13.7 (8.74 - 20.27) | 696.08 (507.29 - 939.02) | 22.06 (13.54 - 32.58) | 1.65 (1.58 - 1.71) |
| Senegal | 1.86 (1.38 - 2.31) | 0.35 (0.22 - 0.48) | 4.53 (3.36 - 5.65) | 0.35 (0.22 - 0.47) | 0.01 (-0.02 - 0.03) | 65.8 (51.85 - 80.82) | 11.86 (9.26 - 14.75) | 191.56 (153.62 - 230.73) | 14.08 (11.09 - 17.19) | 0.55 (0.53 - 0.56) | 0 (0 - 0) | 0 (0 - 0) | 0.01 (0 - 0.02) | 0 (0 - 0) | 1.4 (1.28 - 1.53) | 18.58 (12.53 - 27.01) | 3.35 (2.21 - 4.88) | 54.12 (37.29 - 75.38) | 3.98 (2.7 - 5.63) | 0.55 (0.54 - 0.56) |
| Serbia | 6.83 (4.7 - 9.18) | 0.32 (0.18 - 0.48) | 4.44 (2.91 - 6.52) | 0.17 (0.09 - 0.28) | -2.13 (-2.21 - -2.06) | 1244.44 (1104.47 - 1398.18) | 56.41 (49.49 - 63.57) | 2119.99 (1942.96 - 2323.91) | 77.48 (70.74 - 85.2) | 1.18 (1.08 - 1.28) | 47.25 (29.78 - 69.88) | 2.36 (1.13 - 4.3) | 67.62 (46.27 - 95.89) | 2.42 (1.36 - 3.95) | -0.11 (-0.21 - -0.02) | 1533.92 (1065.97 - 2134.42) | 70.59 (41.21 - 116.24) | 2144.17 (1604.18 - 2803.32) | 78.93 (50.97 - 116.4) | 0.29 (0.21 - 0.37) |
| Seychelles | 0.03 (0.02 - 0.04) | 0.31 (0.2 - 0.43) | 0.06 (0.05 - 0.08) | 0.3 (0.19 - 0.43) | -0.03 (-0.06 - 0.01) | 0.29 (0.22 - 0.37) | 3.03 (2.12 - 4.1) | 0.86 (0.66 - 1.08) | 3.94 (2.88 - 5.15) | 0.82 (0.77 - 0.87) | 0 (0 - 0) | 0.01 (0 - 0.01) | 0 (0 - 0.01) | 0.02 (0.01 - 0.04) | 4.34 (3.14 - 5.56) | 0.09 (0.06 - 0.14) | 0.99 (0.64 - 1.47) | 0.33 (0.24 - 0.45) | 1.52 (0.98 - 2.24) | 1.51 (1.21 - 1.8) |
| Sierra Leone | 1.07 (0.76 - 1.34) | 0.32 (0.19 - 0.45) | 1.88 (1.35 - 2.38) | 0.31 (0.18 - 0.44) | -0.12 (-0.14 - -0.1) | 32.72 (25.69 - 40.04) | 9.63 (7.34 - 12.09) | 68.61 (53.46 - 84.64) | 10.8 (8.35 - 13.38) | 0.35 (0.32 - 0.38) | 0 (0 - 0) | 0 (0 - 0) | 0 (0 - 0.01) | 0 (0 - 0) | 1.18 (1.1 - 1.26) | 9.24 (6.08 - 13.11) | 2.72 (1.77 - 3.92) | 19.37 (13.02 - 27.61) | 3.05 (2.03 - 4.4) | 0.35 (0.32 - 0.38) |
| Singapore | 0.6 (0.38 - 0.88) | 0.16 (0.08 - 0.27) | 2.43 (1.59 - 3.45) | 0.16 (0.08 - 0.27) | 0 (-0.03 - 0.03) | 32.38 (25.73 - 39.7) | 8.74 (6.84 - 10.87) | 148.67 (119.4 - 180.12) | 9.73 (7.67 - 11.94) | 0.37 (0.31 - 0.43) | 0.22 (0.2 - 0.24) | 0.06 (0.05 - 0.07) | 0.52 (0.46 - 0.58) | 0.03 (0.03 - 0.04) | -1.7 (-2.18 - -1.22) | 14.64 (11.51 - 18.64) | 3.93 (3.05 - 5.04) | 54.09 (40.77 - 72.03) | 3.54 (2.61 - 4.77) | -0.29 (-0.47 - -0.12) |
| Slovakia | 3.85 (2.96 - 4.73) | 0.38 (0.25 - 0.51) | 3.75 (2.57 - 5.04) | 0.24 (0.14 - 0.37) | -1.41 (-1.46 - -1.37) | 347.65 (289.97 - 406.2) | 33.26 (27.43 - 39.28) | 668.16 (586.1 - 753.87) | 41.38 (35.82 - 47.19) | 0.74 (0.72 - 0.77) | 16.72 (12.25 - 22.27) | 1.6 (0.95 - 2.52) | 24.72 (17.07 - 36.26) | 1.51 (0.84 - 2.54) | -0.38 (-0.47 - -0.29) | 517.53 (400.27 - 666.87) | 49.5 (32.69 - 72.05) | 781.56 (590.13 - 1059.09) | 48.56 (30.36 - 74.45) | -0.2 (-0.27 - -0.12) |
| Slovenia | 2.17 (1.69 - 2.74) | 0.5 (0.33 - 0.67) | 2.39 (1.71 - 3.18) | 0.35 (0.2 - 0.53) | -1.08 (-1.12 - -1.03) | 289.96 (260.36 - 322.89) | 66.47 (59.31 - 74.3) | 566.19 (516.87 - 622.12) | 81.16 (73.64 - 89.22) | 0.7 (0.65 - 0.74) | 14.78 (13.24 - 16.51) | 3.43 (2.84 - 4.12) | 15.13 (12.46 - 18.33) | 2.02 (1.57 - 2.55) | -1.8 (-2.01 - -1.59) | 446.35 (399.32 - 497.03) | 102.39 (85.46 - 120.44) | 478.83 (396.85 - 568.61) | 67.76 (53.62 - 84.06) | -1.38 (-1.54 - -1.21) |
| Solomon Islands | 0.06 (0.04 - 0.08) | 0.28 (0.17 - 0.41) | 0.14 (0.1 - 0.19) | 0.27 (0.17 - 0.4) | -0.09 (-0.12 - -0.06) | 0.6 (0.44 - 0.79) | 2.38 (1.62 - 3.29) | 1.63 (1.21 - 2.12) | 2.76 (1.94 - 3.74) | 0.46 (0.44 - 0.49) | 0 (0 - 0) | 0 (0 - 0) | 0 (0 - 0) | 0 (0 - 0) | 1.04 (0.88 - 1.21) | 0.17 (0.11 - 0.26) | 0.67 (0.41 - 1.03) | 0.46 (0.3 - 0.68) | 0.78 (0.48 - 1.19) | 0.47 (0.44 - 0.49) |
| Somalia | 0.92 (0.6 - 1.3) | 0.25 (0.14 - 0.38) | 2.3 (1.55 - 3.18) | 0.24 (0.14 - 0.37) | -0.11 (-0.14 - -0.08) | 25.83 (19.25 - 33.24) | 6.37 (4.69 - 8.31) | 69.89 (53.02 - 86.61) | 6.73 (5.05 - 8.58) | 0.12 (0.06 - 0.19) | 0.09 (0.01 - 0.2) | 0.03 (0 - 0.08) | 0.32 (0.03 - 0.69) | 0.04 (0 - 0.1) | 0.76 (0.61 - 0.9) | 9.77 (6.04 - 14.64) | 2.44 (1.44 - 3.95) | 28.3 (16.49 - 41.3) | 2.75 (1.55 - 4.52) | 0.32 (0.28 - 0.36) |
| South Africa | 10.85 (7.94 - 13.62) | 0.32 (0.2 - 0.45) | 26.32 (19.34 - 32.64) | 0.33 (0.21 - 0.45) | 0.17 (0.14 - 0.21) | 464.97 (369.14 - 562.3) | 13.47 (10.57 - 16.51) | 1244.84 (1001.53 - 1483.7) | 15.41 (12.37 - 18.58) | 0.47 (0.39 - 0.55) | 11.45 (6.51 - 15.44) | 0.34 (0.17 - 0.51) | 37.11 (28.9 - 45.64) | 0.47 (0.33 - 0.64) | 0.82 (0.55 - 1.1) | 421.7 (286.16 - 540.72) | 12.14 (7.92 - 16.47) | 1306.09 (1076.21 - 1556.7) | 15.92 (12.3 - 20.4) | 0.71 (0.53 - 0.89) |
| South Sudan | 0.99 (0.68 - 1.33) | 0.24 (0.14 - 0.36) | 1.43 (0.94 - 2.02) | 0.23 (0.13 - 0.35) | -0.2 (-0.23 - -0.17) | 28.48 (21.85 - 35.83) | 6.63 (4.98 - 8.57) | 49.65 (38.37 - 61.31) | 7.32 (5.57 - 9.21) | 0.28 (0.23 - 0.34) | 0.13 (0.02 - 0.25) | 0.03 (0 - 0.08) | 0.32 (0.07 - 0.63) | 0.05 (0.01 - 0.14) | 1.59 (1.53 - 1.65) | 11.12 (6.95 - 15.98) | 2.61 (1.56 - 4.01) | 22.28 (13.04 - 33.29) | 3.33 (1.87 - 5.52) | 0.74 (0.69 - 0.79) |
| Spain | 47.02 (38.44 - 55.6) | 0.5 (0.36 - 0.62) | 75.66 (61.71 - 90.35) | 0.5 (0.35 - 0.63) | -0.03 (-0.06 - 0) | 8803.87 (7677.59 - 9959.37) | 92.33 (80.31 - 104.52) | 22340.06 (20220.73 - 24367.78) | 145.74 (131.63 - 158.85) | 1.14 (0.97 - 1.3) | 88.53 (81.27 - 95.36) | 0.92 (0.77 - 1.09) | 183.52 (160.78 - 206.03) | 1.19 (0.97 - 1.44) | 1.18 (1.06 - 1.3) | 4271.27 (3586.16 - 5136.39) | 44.84 (33.92 - 58.41) | 9623.95 (7927.81 - 11420.9) | 64.64 (49.55 - 83.29) | 1.12 (1.03 - 1.21) |
| Sri Lanka | 5.53 (4.12 - 7.01) | 0.32 (0.21 - 0.43) | 14.96 (11.48 - 18.7) | 0.31 (0.22 - 0.42) | -0.04 (-0.06 - -0.01) | 57.62 (43.61 - 73.96) | 3.1 (2.21 - 4.17) | 199.24 (154.66 - 251.54) | 4.08 (3.01 - 5.31) | 0.92 (0.91 - 0.93) | 0.31 (0.17 - 0.45) | 0.02 (0.01 - 0.04) | 0.95 (0.56 - 1.46) | 0.02 (0.01 - 0.04) | 0.16 (-0.12 - 0.44) | 23.68 (16.87 - 32.34) | 1.29 (0.86 - 1.85) | 78.66 (56.81 - 107.07) | 1.62 (1.11 - 2.24) | 0.7 (0.61 - 0.8) |
| Sudan | 5.11 (3.67 - 6.52) | 0.34 (0.2 - 0.48) | 11.36 (8.11 - 14.7) | 0.35 (0.21 - 0.5) | 0.14 (0.12 - 0.16) | 587.56 (482.59 - 703.35) | 39.07 (31.98 - 46.88) | 1889.73 (1562.81 - 2225.72) | 59.95 (49.82 - 70.78) | 1.4 (1.37 - 1.44) | 2 (0.72 - 3.73) | 0.14 (0.04 - 0.36) | 11.07 (6.61 - 17.72) | 0.37 (0.15 - 0.76) | 2.98 (2.72 - 3.24) | 202.06 (139.36 - 278.02) | 13.46 (8.42 - 20.13) | 759.54 (558.36 - 1008.55) | 23.88 (15.25 - 36.3) | 1.84 (1.77 - 1.9) |
| Suriname | 0.13 (0.09 - 0.17) | 0.3 (0.18 - 0.43) | 0.32 (0.23 - 0.42) | 0.28 (0.17 - 0.41) | -0.16 (-0.18 - -0.14) | 4.58 (3.63 - 5.62) | 10.23 (7.89 - 12.78) | 14.78 (11.88 - 18) | 12.96 (10.27 - 15.9) | 0.83 (0.81 - 0.85) | 0.1 (0.07 - 0.14) | 0.23 (0.13 - 0.39) | 0.37 (0.21 - 0.63) | 0.32 (0.12 - 0.71) | 1.48 (1.27 - 1.7) | 4.07 (3.12 - 5.36) | 8.93 (5.87 - 13.16) | 14.19 (9.18 - 21.98) | 12.16 (6.57 - 22.66) | 1.31 (1.16 - 1.47) |
| Sweden | 15.19 (12.45 - 18.93) | 0.73 (0.53 - 0.98) | 24.94 (19.44 - 32.26) | 0.83 (0.58 - 1.14) | 0.49 (0.45 - 0.54) | 6266.1 (5454.44 - 7143.21) | 267.67 (232.52 - 304.78) | 11923.24 (10393.8 - 13528.91) | 358.91 (313.08 - 406.64) | 0.86 (0.8 - 0.92) | 61.29 (56.13 - 66.87) | 2.53 (2.15 - 2.97) | 121.46 (104.15 - 138.87) | 3.39 (2.73 - 4.14) | 1.42 (1.21 - 1.62) | 2893.03 (2467.77 - 3419.81) | 126.75 (103.32 - 154.65) | 5394.57 (4481.32 - 6417.51) | 163.91 (130.96 - 200.71) | 1.02 (0.9 - 1.13) |
| Switzerland | 8.79 (6.75 - 11.29) | 0.54 (0.34 - 0.76) | 14.96 (11.51 - 19.39) | 0.53 (0.34 - 0.73) | -0.08 (-0.09 - -0.07) | 2760.25 (2425.64 - 3176.33) | 168.19 (148.27 - 192.75) | 5370.74 (4664.88 - 6186.04) | 188.81 (164.72 - 216.56) | 0.36 (0.31 - 0.41) | 71.92 (63.91 - 80.26) | 4.16 (3.36 - 5.09) | 108.02 (94.74 - 123.43) | 3.6 (2.89 - 4.42) | -0.08 (-0.23 - 0.08) | 2296.03 (2034.29 - 2615.96) | 139.73 (115.66 - 167.63) | 3652.5 (3159.03 - 4189.01) | 128.84 (105.31 - 154.63) | 0 (-0.13 - 0.12) |
| Syrian Arab Republic | 3.63 (2.6 - 4.63) | 0.4 (0.25 - 0.54) | 10.28 (7.43 - 13.03) | 0.42 (0.27 - 0.57) | 0.17 (0.16 - 0.19) | 556.7 (455.87 - 670.08) | 62.44 (50.86 - 75.04) | 2253.65 (1863.96 - 2675.67) | 94.39 (77.93 - 111.94) | 1.36 (1.34 - 1.39) | 1.37 (0.44 - 2.53) | 0.16 (0.04 - 0.39) | 5.73 (3.12 - 9.1) | 0.25 (0.1 - 0.53) | 1.38 (1.25 - 1.51) | 172.47 (117.15 - 239.03) | 19.11 (11.1 - 29.87) | 703.12 (523.05 - 941.45) | 28.99 (18.15 - 42.89) | 1.35 (1.32 - 1.38) |
| Taiwan (Province of China) | 9.63 (7.63 - 11.89) | 0.35 (0.25 - 0.45) | 19.87 (14.93 - 25.14) | 0.26 (0.17 - 0.38) | -0.99 (-1.06 - -0.93) | 108.46 (92.31 - 126.38) | 3.84 (3.08 - 4.68) | 697.62 (585.89 - 810.49) | 9.26 (7.63 - 10.97) | 3.25 (2.94 - 3.57) | 0.57 (0.53 - 0.62) | 0.03 (0.02 - 0.03) | 2.89 (2.42 - 3.34) | 0.04 (0.03 - 0.05) | -2.66 (-4.08 - -1.21) | 41.95 (32.1 - 53.34) | 1.57 (1.19 - 2.02) | 264 (201.95 - 340.61) | 3.51 (2.63 - 4.55) | 0.67 (-0.15 - 1.49) |
| Tajikistan | 2.54 (1.98 - 3.11) | 0.5 (0.33 - 0.67) | 5.05 (3.88 - 6.32) | 0.41 (0.27 - 0.56) | -0.68 (-0.72 - -0.64) | 212.29 (168.6 - 255.97) | 44.67 (35.47 - 54.01) | 458.63 (375.51 - 544.93) | 43.05 (34.78 - 51.9) | -0.13 (-0.21 - -0.05) | 1.19 (0.77 - 1.65) | 0.3 (0.17 - 0.47) | 1.67 (0.92 - 2.76) | 0.27 (0.12 - 0.52) | 0.11 (-0.39 - 0.62) | 73.56 (53.8 - 98.94) | 15.99 (9.9 - 23.88) | 148.69 (104.15 - 207.55) | 15.14 (9.22 - 23.5) | -0.06 (-0.23 - 0.11) |
| Thailand | 18.36 (13.27 - 24.04) | 0.32 (0.21 - 0.44) | 58.03 (42.7 - 73.46) | 0.3 (0.2 - 0.42) | -0.17 (-0.18 - -0.16) | 211.45 (158.37 - 272.37) | 3.39 (2.4 - 4.59) | 831.12 (645.27 - 1041.55) | 4.24 (3.12 - 5.52) | 0.71 (0.7 - 0.73) | 0.5 (0.22 - 0.9) | 0.01 (0 - 0.02) | 2.75 (1.6 - 4.18) | 0.01 (0.01 - 0.03) | 1.32 (1.17 - 1.47) | 72.76 (50.59 - 104.79) | 1.16 (0.76 - 1.7) | 302.63 (219.17 - 411.91) | 1.54 (1.06 - 2.15) | 0.84 (0.81 - 0.87) |
| Timor-Leste | 0.13 (0.09 - 0.17) | 0.31 (0.19 - 0.43) | 0.44 (0.33 - 0.55) | 0.31 (0.21 - 0.42) | 0.09 (0.06 - 0.11) | 1.02 (0.73 - 1.35) | 2.13 (1.41 - 3.02) | 3.83 (2.87 - 4.91) | 2.65 (1.85 - 3.63) | 0.81 (0.76 - 0.87) | 0 (0 - 0.01) | 0.01 (0 - 0.02) | 0.03 (0.01 - 0.04) | 0.02 (0.01 - 0.05) | 2.66 (2.42 - 2.91) | 0.37 (0.24 - 0.56) | 0.79 (0.47 - 1.25) | 1.67 (1.12 - 2.33) | 1.17 (0.71 - 1.9) | 1.39 (1.32 - 1.45) |
| Togo | 0.66 (0.48 - 0.84) | 0.34 (0.21 - 0.46) | 2.08 (1.53 - 2.63) | 0.33 (0.2 - 0.46) | -0.04 (-0.08 - 0) | 22.19 (17.31 - 27.54) | 10.63 (8.15 - 13.38) | 83.29 (65.97 - 103.06) | 12.32 (9.64 - 15.31) | 0.5 (0.48 - 0.52) | 0 (0 - 0) | 0 (0 - 0) | 0 (0 - 0.01) | 0 (0 - 0) | 1.05 (1 - 1.1) | 6.27 (4.16 - 9.17) | 3 (1.96 - 4.42) | 23.53 (15.55 - 34.15) | 3.48 (2.28 - 5.04) | 0.5 (0.48 - 0.52) |
| Tokelau | 0 (0 - 0) | 0.28 (0.18 - 0.41) | 0 (0 - 0) | 0.26 (0.16 - 0.38) | -0.3 (-0.32 - -0.29) | 0.01 (0 - 0.01) | 2.15 (1.45 - 3.04) | 0.01 (0 - 0.01) | 2.57 (1.8 - 3.5) | 0.51 (0.48 - 0.54) | 0 (0 - 0) | 0 (0 - 0) | 0 (0 - 0) | 0 (0 - 0) | 0.68 (0.6 - 0.76) | 0 (0 - 0) | 0.61 (0.36 - 0.94) | 0 (0 - 0) | 0.73 (0.45 - 1.11) | 0.51 (0.48 - 0.54) |
| Tonga | 0.03 (0.02 - 0.04) | 0.31 (0.19 - 0.43) | 0.04 (0.03 - 0.05) | 0.29 (0.19 - 0.41) | -0.15 (-0.16 - -0.14) | 0.29 (0.22 - 0.38) | 2.95 (2.06 - 4.03) | 0.46 (0.35 - 0.58) | 3.35 (2.39 - 4.45) | 0.37 (0.33 - 0.41) | 0 (0 - 0) | 0 (0 - 0) | 0 (0 - 0) | 0 (0 - 0) | 1.64 (1.57 - 1.72) | 0.08 (0.05 - 0.12) | 0.83 (0.51 - 1.27) | 0.13 (0.08 - 0.19) | 0.95 (0.59 - 1.42) | 0.37 (0.33 - 0.41) |
| Trinidad and Tobago | 0.43 (0.31 - 0.55) | 0.31 (0.19 - 0.44) | 0.97 (0.68 - 1.27) | 0.28 (0.16 - 0.41) | -0.43 (-0.45 - -0.4) | 17.11 (13.88 - 21.08) | 12.42 (9.78 - 15.4) | 55.5 (44.83 - 66.96) | 15.8 (12.67 - 19.34) | 0.86 (0.84 - 0.89) | 0.5 (0.46 - 0.54) | 0.37 (0.3 - 0.44) | 1.59 (1.18 - 2.06) | 0.45 (0.32 - 0.62) | 0.62 (0.5 - 0.73) | 17.66 (15.81 - 19.78) | 12.86 (10.87 - 15.19) | 57.46 (45.6 - 71.37) | 16.19 (12.33 - 20.95) | 0.68 (0.59 - 0.77) |
| Tunisia | 3.74 (2.73 - 4.71) | 0.42 (0.27 - 0.57) | 10.37 (7.74 - 13.4) | 0.44 (0.28 - 0.6) | 0.14 (0.13 - 0.15) | 667.81 (553.26 - 790.59) | 76.05 (62.96 - 90.3) | 2877.77 (2427.98 - 3383.84) | 122.82 (103.44 - 144.47) | 1.58 (1.56 - 1.61) | 2.16 (0.74 - 3.75) | 0.28 (0.07 - 0.66) | 15.7 (8.82 - 24.85) | 0.7 (0.27 - 1.48) | 2.81 (2.42 - 3.21) | 217.72 (153.72 - 301.99) | 24.79 (14.94 - 37.72) | 1065.09 (789.53 - 1440.34) | 45.22 (28.68 - 67.64) | 1.96 (1.82 - 2.1) |
| Turkey | 25.93 (19 - 32.51) | 0.42 (0.27 - 0.55) | 70.02 (52.87 - 86.11) | 0.42 (0.28 - 0.55) | 0.01 (0 - 0.03) | 5912.36 (5556 - 6302.51) | 98.28 (91.92 - 104.91) | 17367.98 (16285.54 - 18465.74) | 105.01 (98.39 - 111.88) | 0.27 (0.22 - 0.33) | 25.04 (11.3 - 43.77) | 0.43 (0.14 - 0.98) | 102.66 (71.35 - 140.44) | 0.64 (0.34 - 1.09) | 1.37 (1.21 - 1.53) | 2085.86 (1499.62 - 2753.93) | 34.02 (20.85 - 50.5) | 6719.63 (5221.44 - 8475.94) | 40.34 (27.62 - 56.81) | 0.61 (0.52 - 0.69) |
| Turkmenistan | 3.23 (2.72 - 3.75) | 0.89 (0.67 - 1.09) | 7.27 (6.13 - 8.64) | 0.85 (0.65 - 1.07) | -0.09 (-0.17 - -0.01) | 318.66 (283.09 - 355.25) | 95.71 (84.57 - 107.2) | 776.27 (695.34 - 870.42) | 106.86 (95.43 - 119.56) | 0.46 (0.4 - 0.52) | 4.33 (3.65 - 5.1) | 1.83 (1.43 - 2.26) | 6.64 (3.77 - 9.37) | 1.3 (0.72 - 1.93) | -0.97 (-1.35 - -0.58) | 152.7 (123.57 - 183.96) | 51.11 (37.85 - 66.11) | 308.23 (222.28 - 391.7) | 46.55 (30.19 - 64.28) | -0.15 (-0.31 - 0.01) |
| Tuvalu | 0 (0 - 0) | 0.3 (0.2 - 0.42) | 0 (0 - 0.01) | 0.27 (0.17 - 0.39) | -0.37 (-0.39 - -0.34) | 0.02 (0.02 - 0.03) | 1.87 (1.25 - 2.65) | 0.04 (0.03 - 0.05) | 2.19 (1.48 - 3.03) | 0.43 (0.4 - 0.47) | 0 (0 - 0) | 0 (0 - 0) | 0 (0 - 0) | 0 (0 - 0) | 1.03 (0.95 - 1.1) | 0.01 (0 - 0.01) | 0.53 (0.31 - 0.83) | 0.01 (0.01 - 0.02) | 0.62 (0.37 - 0.96) | 0.43 (0.4 - 0.47) |
| Uganda | 2.31 (1.55 - 3.21) | 0.22 (0.12 - 0.34) | 5.18 (3.5 - 7.29) | 0.22 (0.12 - 0.34) | -0.08 (-0.09 - -0.06) | 67.95 (52.1 - 85.87) | 6.17 (4.59 - 7.96) | 176.39 (135.9 - 219.86) | 7.03 (5.32 - 8.94) | 0.45 (0.39 - 0.5) | 0.27 (0.05 - 0.5) | 0.03 (0 - 0.07) | 1.32 (0.45 - 2.34) | 0.06 (0.01 - 0.14) | 2.41 (2.21 - 2.6) | 25.68 (16.08 - 36.7) | 2.35 (1.42 - 3.61) | 82.37 (52.69 - 121.47) | 3.33 (2.02 - 5.33) | 1.11 (1.08 - 1.14) |
| Ukraine | 57.46 (45.61 - 71.18) | 0.48 (0.33 - 0.65) | 35.6 (27.85 - 44.79) | 0.27 (0.18 - 0.38) | -2.03 (-2.28 - -1.78) | 6090.91 (5097.23 - 7244.14) | 48.29 (40.21 - 57.38) | 5513.24 (4770.09 - 6337.22) | 41.03 (35.13 - 47.16) | -0.55 (-0.6 - -0.5) | 118.47 (106.54 - 130.93) | 1 (0.84 - 1.17) | 88.93 (64.76 - 117.76) | 0.67 (0.47 - 0.93) | -2.45 (-2.89 - -2.01) | 4417.19 (3797.73 - 5104.48) | 35.6 (29.05 - 43.49) | 3688.8 (2868.34 - 4565.8) | 28.03 (20.78 - 36.55) | -1.51 (-1.79 - -1.23) |
| United Arab Emirates | 0.16 (0.1 - 0.24) | 0.27 (0.14 - 0.43) | 2.64 (1.64 - 3.82) | 0.31 (0.17 - 0.48) | 0.17 (-0.05 - 0.38) | 26.88 (22.82 - 31.16) | 45.8 (38.59 - 53.47) | 377.9 (333.12 - 429.13) | 47.59 (41.46 - 54.38) | 0.28 (0.17 - 0.39) | 0.1 (0.03 - 0.2) | 0.19 (0.05 - 0.46) | 1.02 (0.57 - 1.62) | 0.23 (0.09 - 0.47) | 1.91 (1.32 - 2.5) | 9.75 (6.51 - 13.72) | 16.43 (9.94 - 25.64) | 125.95 (88.82 - 165.74) | 16.92 (10.57 - 24.92) | 0.52 (0.32 - 0.73) |
| United Kingdom | 79.33 (59.91 - 101.69) | 0.59 (0.38 - 0.83) | 101.93 (74.03 - 133.61) | 0.52 (0.32 - 0.75) | -0.19 (-0.42 - 0.04) | 22463.32 (19717.11 - 25623.57) | 156.28 (136.17 - 179.31) | 49423.2 (43248.94 - 56149.73) | 242.45 (212.18 - 275.94) | 1.5 (1.4 - 1.6) | 607.36 (584.24 - 624.31) | 4.06 (3.88 - 4.21) | 1157.75 (1071.31 - 1219.31) | 5.39 (4.99 - 5.72) | 1.68 (1.44 - 1.91) | 19819.93 (18129.39 - 21642.39) | 138.65 (126.87 - 151.65) | 37903.5 (33972.4 - 41768.81) | 186.63 (166.8 - 206.24) | 1.52 (1.35 - 1.68) |
| United Republic of Tanzania | 4.4 (2.93 - 6.02) | 0.24 (0.14 - 0.37) | 9.77 (6.65 - 13.31) | 0.24 (0.13 - 0.36) | -0.05 (-0.07 - -0.03) | 138.2 (106.47 - 172.94) | 7.22 (5.44 - 9.3) | 348.37 (270.96 - 431.7) | 8.04 (6.13 - 10.12) | 0.28 (0.24 - 0.32) | 0.74 (0.17 - 1.34) | 0.04 (0.01 - 0.11) | 2.83 (0.94 - 4.96) | 0.07 (0.02 - 0.17) | 1.49 (1.42 - 1.55) | 57.44 (35.44 - 84.32) | 3.04 (1.78 - 4.81) | 168.17 (107.25 - 243.36) | 3.92 (2.33 - 6.41) | 0.74 (0.7 - 0.77) |
| United States Virgin Islands | 0.05 (0.04 - 0.06) | 0.35 (0.22 - 0.47) | 0.11 (0.08 - 0.13) | 0.34 (0.21 - 0.47) | -0.08 (-0.09 - -0.07) | 2.65 (2.14 - 3.17) | 18.52 (14.83 - 22.47) | 7.5 (6.22 - 8.83) | 23.59 (19.39 - 28.08) | 0.78 (0.73 - 0.84) | 0.06 (0.04 - 0.1) | 0.44 (0.19 - 0.91) | 0.12 (0.07 - 0.18) | 0.38 (0.16 - 0.78) | -0.37 (-0.57 - -0.17) | 2.37 (1.65 - 3.48) | 16.25 (9.62 - 27.97) | 4.99 (3.64 - 6.79) | 16.08 (9.63 - 26.74) | 0.06 (-0.06 - 0.18) |
| United States of America | 247.79 (191.52 - 314.05) | 0.5 (0.32 - 0.69) | 307.28 (222.41 - 406.38) | 0.32 (0.19 - 0.48) | -1.37 (-1.74 - -0.99) | 98843.71 (84153.05 - 114340.26) | 190.55 (162.33 - 220.56) | 185456.12 (169860.98 - 201494.7) | 187.31 (171.31 - 203.94) | 0.38 (0.23 - 0.52) | 979.27 (922.84 - 1028.89) | 1.87 (1.75 - 1.98) | 3670.09 (3375.07 - 3898.97) | 3.57 (3.23 - 3.84) | 2.08 (1.7 - 2.47) | 47316.36 (40251.02 - 55168.51) | 92.7 (78.64 - 108.85) | 125970.45 (111974.78 - 138562.59) | 126.15 (111.44 - 139.7) | 1.2 (0.95 - 1.44) |
| Uruguay | 2.03 (1.45 - 2.61) | 0.31 (0.18 - 0.45) | 2.44 (1.69 - 3.22) | 0.28 (0.16 - 0.42) | -0.25 (-0.3 - -0.2) | 303.89 (251.85 - 355.86) | 45.23 (37.57 - 53.28) | 438.52 (373.08 - 515.69) | 49.93 (42.39 - 58.65) | 0.32 (0.3 - 0.35) | 9.79 (9.01 - 10.61) | 1.45 (1.21 - 1.72) | 10.02 (8.69 - 11.34) | 1.1 (0.88 - 1.34) | -1.1 (-1.35 - -0.84) | 315.42 (281.99 - 350.56) | 47.06 (39.17 - 56.17) | 343.45 (293.41 - 396.23) | 39.51 (31.65 - 48.56) | -0.7 (-0.89 - -0.51) |
| Uzbekistan | 15.09 (12.29 - 17.91) | 0.71 (0.51 - 0.91) | 26.2 (20.55 - 32.91) | 0.48 (0.32 - 0.66) | -1.44 (-1.55 - -1.33) | 1467.07 (1239.31 - 1718.28) | 74.18 (62.33 - 86.88) | 2613.08 (2190.98 - 3056.71) | 54.64 (45.47 - 64.39) | -1.14 (-1.21 - -1.06) | 7.98 (6.46 - 9.68) | 0.45 (0.35 - 0.56) | 12.75 (9.83 - 15.88) | 0.42 (0.31 - 0.56) | 1.95 (0.88 - 3.03) | 495.59 (373.31 - 638.01) | 25.51 (17.32 - 35.98) | 889.2 (664.76 - 1149.97) | 20.22 (13.87 - 28.14) | -0.28 (-0.58 - 0.02) |
| Vanuatu | 0.03 (0.02 - 0.04) | 0.33 (0.21 - 0.45) | 0.09 (0.06 - 0.11) | 0.32 (0.21 - 0.43) | -0.13 (-0.14 - -0.11) | 0.22 (0.16 - 0.29) | 2.1 (1.39 - 2.97) | 0.72 (0.53 - 0.94) | 2.35 (1.62 - 3.25) | 0.32 (0.29 - 0.36) | 0 (0 - 0) | 0 (0 - 0) | 0 (0 - 0) | 0 (0 - 0) | 1.31 (1.2 - 1.43) | 0.06 (0.04 - 0.1) | 0.59 (0.35 - 0.92) | 0.2 (0.13 - 0.31) | 0.66 (0.4 - 1.03) | 0.33 (0.29 - 0.36) |
| Venezuela (Bolivarian Republic of) | 5.19 (3.83 - 6.53) | 0.33 (0.21 - 0.45) | 17.42 (13.02 - 21.41) | 0.33 (0.21 - 0.46) | 0.03 (-0.02 - 0.08) | 175.28 (138.85 - 216.28) | 10.96 (8.58 - 13.62) | 840.22 (680.33 - 1006.25) | 15.81 (12.66 - 19.03) | 1.25 (1.16 - 1.35) | 6.11 (5.61 - 6.63) | 0.39 (0.32 - 0.47) | 43.12 (33.63 - 55.75) | 0.83 (0.6 - 1.13) | 2.34 (1.99 - 2.69) | 201.88 (180.78 - 225.95) | 12.57 (10.65 - 14.78) | 1305.78 (1039.94 - 1643.44) | 24.48 (18.62 - 31.78) | 2.06 (1.76 - 2.35) |
| Viet Nam | 24.15 (18.4 - 30.25) | 0.35 (0.25 - 0.45) | 60.87 (48.02 - 74.15) | 0.36 (0.27 - 0.45) | 0.13 (0.1 - 0.16) | 242.71 (184.63 - 312.5) | 3.38 (2.42 - 4.56) | 835.88 (650.52 - 1032.08) | 4.63 (3.46 - 5.96) | 1.04 (1.02 - 1.05) | 1.1 (0.32 - 2) | 0.02 (0 - 0.04) | 6.01 (3.2 - 10.22) | 0.04 (0.01 - 0.09) | 2.56 (2.26 - 2.87) | 93.28 (60.57 - 135.64) | 1.31 (0.79 - 2.07) | 375.28 (261.85 - 517.83) | 2.11 (1.32 - 3.33) | 1.56 (1.46 - 1.66) |
| Yemen | 2.81 (1.96 - 3.54) | 0.34 (0.2 - 0.48) | 8.18 (5.84 - 10.44) | 0.35 (0.21 - 0.5) | 0.13 (0.11 - 0.14) | 324.73 (263.34 - 385.85) | 40.09 (32.44 - 47.85) | 1344.29 (1126.31 - 1581.4) | 59 (49.17 - 69.58) | 1.35 (1.31 - 1.4) | 0.98 (0.26 - 2.07) | 0.13 (0.03 - 0.35) | 7.46 (4.1 - 12.82) | 0.35 (0.13 - 0.76) | 3.23 (2.86 - 3.6) | 109.42 (74.29 - 156.97) | 13.39 (7.96 - 20.78) | 530.89 (386.53 - 736.15) | 23.11 (14.63 - 35.25) | 1.87 (1.76 - 1.97) |
| Zambia | 1.34 (0.93 - 1.76) | 0.29 (0.17 - 0.42) | 3.04 (2.11 - 3.98) | 0.28 (0.16 - 0.41) | -0.08 (-0.11 - -0.05) | 39.22 (30.37 - 49.1) | 7.9 (5.95 - 10.11) | 108.36 (83.46 - 133.94) | 9.4 (7.2 - 11.82) | 0.58 (0.51 - 0.65) | 0.23 (0.06 - 0.44) | 0.05 (0.01 - 0.13) | 1 (0.41 - 1.65) | 0.1 (0.03 - 0.23) | 1.86 (1.77 - 1.94) | 16.97 (10.31 - 25.09) | 3.47 (2.02 - 5.6) | 55.7 (36.57 - 76.98) | 4.91 (3.03 - 8) | 1.09 (1.03 - 1.16) |

**Abbreviations**: UI, uncertainty interval; ASR, age-standerised rate per 100,000; CI, confidence interval; DALYs, disability-adjusted life-year; EAPC, estimated annual percentage change.

**Table S8 Global burden of Parkinson’s disease among adults aged≥55 years from 1990 to 2021, categorized by countries**

| **Countries** | **Incidence** | | | | | **Prevalence** | | | | | **Deaths** | | | | | **DALYs** | | | | |
| --- | --- | --- | --- | --- | --- | --- | --- | --- | --- | --- | --- | --- | --- | --- | --- | --- | --- | --- | --- | --- |
|  | **No.(95% UI) in 1990** | **ASR per 100000 (95% UI) in 1990** | **No.(95% UI) in 2021** | **ASR per 100000 (95% UI) in 2021** | **EAPC**  **(95% CI)** | **No.(95% UI) in 1990** | **ASR per 100000 (95% UI) in 1990** | **No.(95% UI) in 2021** | **ASR per 100000 (95% UI) in 2021** | **EAPC**  **(95% CI)** | **No.(95% UI) in 1990** | **ASR per 100000 (95% UI) in 1990** | **No.(95% UI) in 2021** | **ASR per 100000 (95% UI) in 2021** | **EAPC**  **(95% CI)** | **No.(95% UI) in 1990** | **ASR per 100000 (95% UI) in 1990** | **No.(95% UI) in 2021** | **ASR per 100000 (95% UI) in 2021** | **EAPC**  **(95% CI)** |
| Afghanistan | 539.93 (463.92 - 619.7) | 54.9 (41.13 - 70.32) | 688.24 (589.02 - 767.24) | 60.51 (44.06 - 77.73) | 0.35 (0.29 - 0.42) | 3230.1 (2637.8 - 3995.81) | 320.89 (233.06 - 431.71) | 4751.76 (3782.83 - 5659.22) | 419.89 (305.36 - 542.22) | 0.95 (0.8 - 1.09) | 368.18 (254.35 - 520.46) | 43.73 (29.83 - 64.46) | 432.59 (327.21 - 552.01) | 41.55 (29.76 - 57.12) | -0.13 (-0.16 - -0.1) | 7098.57 (4785.96 - 9808.9) | 735.31 (499.82 - 1061.03) | 7846.36 (5889.81 - 9798.41) | 699.14 (504.36 - 936.44) | -0.15 (-0.19 - -0.12) |
| Albania | 210.09 (180.98 - 240.64) | 67.44 (48.97 - 88.4) | 571.94 (494.05 - 641.28) | 72.13 (51.43 - 94.69) | 0.28 (0.25 - 0.31) | 1523.8 (1233.63 - 1836.82) | 503.44 (376.32 - 645.09) | 4355.66 (3456.39 - 5156.72) | 553.79 (403.59 - 705.68) | 0.36 (0.31 - 0.41) | 88.21 (78.2 - 99.5) | 34.28 (28.67 - 40.89) | 239.11 (194.61 - 289.06) | 33.13 (25.89 - 41.04) | 0.08 (-0.07 - 0.23) | 1548.4 (1383.2 - 1726.14) | 556.82 (473.53 - 657.82) | 3944.48 (3309.71 - 4647.36) | 521.74 (420.11 - 633.84) | -0.04 (-0.17 - 0.1) |
| Algeria | 1059.66 (890.51 - 1221.32) | 62.06 (45.96 - 79.47) | 3575.25 (3048.17 - 4161.83) | 69.95 (51.19 - 95.68) | 0.4 (0.35 - 0.46) | 6613.8 (5295.27 - 8077.36) | 389.02 (286.32 - 505.43) | 26536.12 (21425.49 - 32468.35) | 518.31 (383.29 - 688.61) | 0.94 (0.86 - 1.02) | 333.85 (270.46 - 409.78) | 29.76 (21.69 - 39.92) | 1171.69 (946.2 - 1447.65) | 29 (20.91 - 39.23) | 0.32 (0.18 - 0.46) | 6385.04 (5138.19 - 7881.08) | 450.6 (335.07 - 596.42) | 20937.31 (17196.66 - 25979.88) | 450.5 (339.8 - 594.8) | 0.24 (0.15 - 0.33) |
| American Samoa | 1.36 (1.13 - 1.6) | 46.5 (32.41 - 62.62) | 3.34 (2.88 - 3.85) | 47.48 (34.52 - 62.38) | 0.19 (0.13 - 0.25) | 10.63 (8.54 - 12.9) | 379.06 (273.08 - 490.52) | 27.58 (23.32 - 32.82) | 402.08 (306.1 - 509.31) | 0.33 (0.24 - 0.43) | 0.76 (0.65 - 0.86) | 33.03 (26.36 - 39.84) | 1.78 (1.49 - 2.15) | 29.69 (23.14 - 37.36) | -0.23 (-0.3 - -0.15) | 14.85 (12.88 - 16.68) | 555.09 (452.63 - 660.03) | 32.98 (27.92 - 39.61) | 498.86 (399.73 - 616.65) | -0.23 (-0.3 - -0.16) |
| Andorra | 6.86 (5.73 - 8.14) | 71.98 (51.01 - 98.3) | 22.71 (20.09 - 26.19) | 86.35 (62.81 - 116.48) | 0.6 (0.55 - 0.64) | 54.7 (44.02 - 65.58) | 592.65 (441.48 - 778.52) | 208.31 (175.61 - 246.16) | 774.22 (603.53 - 984.56) | 0.89 (0.82 - 0.96) | 2.28 (1.68 - 3.03) | 27.1 (19.36 - 36.86) | 6.28 (4.45 - 8.12) | 22.19 (15.24 - 29.83) | -0.3 (-0.53 - -0.07) | 42.55 (33.06 - 54.82) | 468.5 (350.74 - 617.16) | 112.52 (86.12 - 142.49) | 414.75 (308.21 - 532.67) | -0.12 (-0.32 - 0.07) |
| Angola | 193.7 (164.92 - 229.77) | 42.88 (31.36 - 56.24) | 681.88 (574.89 - 812.74) | 48.23 (34.55 - 64.33) | 0.45 (0.4 - 0.5) | 1260.25 (1009.14 - 1568.33) | 270.57 (198.06 - 361.18) | 4801.82 (3801.4 - 6046.32) | 336.95 (241.81 - 453.03) | 0.75 (0.67 - 0.83) | 82.68 (67.93 - 101.42) | 24.21 (18.27 - 32.02) | 285.24 (220.28 - 365.86) | 27.14 (20.17 - 36.75) | 0.3 (0.24 - 0.36) | 1712.16 (1430.37 - 2089.27) | 411.03 (319.82 - 527.3) | 5684.29 (4551.02 - 7036.86) | 450.96 (347.84 - 587.44) | 0.23 (0.17 - 0.29) |
| Antigua and Barbuda | 4.88 (4.38 - 5.38) | 48.95 (37.82 - 61.63) | 10.89 (9.72 - 12.5) | 66.13 (51.51 - 83.63) | 0.86 (0.8 - 0.93) | 31.02 (25.83 - 36.89) | 323.05 (244.06 - 415.86) | 77.58 (64.43 - 97.85) | 458.53 (346.38 - 603) | 0.9 (0.79 - 1.01) | 3.02 (2.79 - 3.19) | 28.92 (26.17 - 31.34) | 5.36 (4.9 - 5.72) | 36.04 (31.96 - 39.49) | 0.91 (0.69 - 1.12) | 47.59 (44.44 - 50.78) | 468.77 (426.29 - 512.8) | 92.33 (83.81 - 98.99) | 577.68 (511.18 - 640.07) | 0.73 (0.55 - 0.9) |
| Argentina | 4027.81 (3637.32 - 4348.63) | 74.3 (59.45 - 88.15) | 7508.37 (6801.24 - 8686.4) | 75.81 (59.69 - 95.92) | 0.16 (0.1 - 0.22) | 29164.91 (25366.79 - 32239.58) | 551.06 (446.14 - 649.04) | 57393.85 (48172.83 - 69372.25) | 578.28 (449.64 - 735.19) | 0.26 (0.12 - 0.39) | 1504.69 (1410.53 - 1563.85) | 30.44 (27.91 - 32.5) | 2794.32 (2529.3 - 2964.49) | 27.67 (24.69 - 29.89) | -0.06 (-0.17 - 0.05) | 26770.07 (25027.2 - 28446.42) | 514.02 (469.48 - 557.38) | 47503.93 (43611.01 - 51189.33) | 473.67 (423.86 - 521.48) | -0.06 (-0.14 - 0.02) |
| Armenia | 201.33 (182.67 - 223.51) | 49.15 (37.23 - 61.74) | 410.41 (363.23 - 467.05) | 54.1 (41.57 - 68.79) | 0.33 (0.32 - 0.35) | 1604.04 (1388.99 - 1880.28) | 401.39 (318.31 - 494.33) | 3240.78 (2671.26 - 3903.28) | 431.52 (332.19 - 541.33) | 0.27 (0.22 - 0.31) | 78.72 (71.71 - 87.13) | 21.73 (19.47 - 24.26) | 163.03 (143.86 - 181.13) | 22.37 (19.45 - 25.02) | -0.16 (-0.36 - 0.04) | 1430.24 (1300.96 - 1580.8) | 376.1 (335.9 - 421.1) | 2777.45 (2446.69 - 3079.2) | 380.02 (328.85 - 428.85) | -0.19 (-0.36 - -0.01) |
| Australia | 1866.31 (1615.88 - 2034.92) | 54.48 (41.47 - 67.79) | 5613.23 (5091.83 - 6384.59) | 68.11 (53.03 - 84.83) | 0.77 (0.71 - 0.84) | 12936.92 (10331.33 - 15180.11) | 376.33 (284.35 - 468.17) | 42627.27 (35814.59 - 52238.82) | 507.92 (397.62 - 646.54) | 1 (0.91 - 1.08) | 877.88 (805.38 - 919.64) | 26.81 (24.22 - 28.69) | 2437.57 (2096.52 - 2626.57) | 27.23 (23.29 - 29.69) | 0.16 (0.02 - 0.31) | 14864.08 (13855.79 - 15814.85) | 438.42 (398.35 - 476.54) | 38657.72 (34196.65 - 41790.1) | 447.59 (390.96 - 494.39) | 0.15 (0.02 - 0.28) |
| Austria | 1599.11 (1409.63 - 1814.25) | 72.8 (55.72 - 93.31) | 3141.93 (2547.89 - 3770.73) | 94.19 (64.87 - 123.78) | 0.91 (0.87 - 0.94) | 12952.92 (11030.98 - 15350.56) | 587.82 (471.55 - 731.71) | 28509.73 (21383.82 - 35099.84) | 813.52 (579.48 - 1041.29) | 1.11 (1.07 - 1.14) | 614.94 (566.94 - 644.57) | 26.95 (24.39 - 28.72) | 1069.15 (907.91 - 1151.95) | 27.69 (23.61 - 30.13) | 0.46 (0.24 - 0.68) | 10460.78 (9738.17 - 11206.1) | 461.51 (417.11 - 505.69) | 17817.41 (15973.32 - 19543.45) | 491.98 (426.84 - 554.48) | 0.52 (0.36 - 0.67) |
| Azerbaijan | 402.18 (348.58 - 460.61) | 55.01 (39.01 - 73.9) | 1008.76 (880.88 - 1144.29) | 73.38 (56.2 - 92.97) | 1.01 (0.96 - 1.05) | 3023.32 (2465.16 - 3711.88) | 414.93 (305.58 - 549.54) | 6719 (5415.25 - 8008.14) | 498.56 (369.94 - 639.85) | 0.63 (0.58 - 0.68) | 164.84 (129.28 - 214.13) | 25.52 (18.78 - 35.57) | 259.42 (212.87 - 310.4) | 22.69 (17.8 - 28.43) | 0.2 (-0.06 - 0.46) | 2876.25 (2363.43 - 3510.36) | 418.46 (325.92 - 548.37) | 4950.04 (4197.79 - 5761.41) | 397.07 (324.51 - 486.65) | 0.22 (0.04 - 0.4) |
| Bahamas | 10.92 (9.65 - 12.16) | 48.21 (37.78 - 60.88) | 33.98 (30.73 - 37.5) | 56.67 (44.39 - 69.81) | 0.43 (0.36 - 0.51) | 74.83 (62.62 - 90.44) | 320.65 (240.68 - 410.78) | 253.61 (214.21 - 295.89) | 403.81 (313.94 - 501.27) | 0.53 (0.36 - 0.69) | 5.32 (4.93 - 5.67) | 24.29 (21.97 - 26.38) | 17.54 (14.69 - 20.76) | 32.42 (26.86 - 38.28) | 0.99 (0.78 - 1.2) | 97.28 (90.45 - 104.88) | 422.68 (382.01 - 463.82) | 306.6 (260.89 - 362.02) | 527.82 (441.49 - 621.34) | 0.79 (0.63 - 0.94) |
| Bahrain | 12.65 (10.59 - 14.62) | 67.23 (51.08 - 84.96) | 85.62 (71.48 - 105.37) | 89.87 (67.3 - 120.15) | 0.88 (0.84 - 0.92) | 80.17 (65.46 - 97.78) | 417.33 (307.38 - 542.52) | 593.88 (489.79 - 725.74) | 642.27 (484.56 - 843.81) | 1.38 (1.34 - 1.43) | 6.13 (5.49 - 6.83) | 48.29 (40.28 - 57.42) | 21.39 (17.94 - 25.12) | 39.98 (32.3 - 48.95) | -0.59 (-1.02 - -0.16) | 118.09 (105.54 - 130.43) | 744.17 (625.38 - 873.87) | 434.45 (370.92 - 508.86) | 618.77 (505.98 - 746.26) | -0.66 (-1.01 - -0.31) |
| Bangladesh | 3503.43 (3022.16 - 4033.69) | 52.1 (38.25 - 67.89) | 12158.69 (10362.36 - 14210.14) | 55.89 (37.75 - 78.37) | 0.25 (0.17 - 0.33) | 23670.73 (18830.46 - 29000.93) | 345.98 (252.89 - 460.07) | 98478.92 (80297.29 - 121872.46) | 455.68 (338.57 - 619.9) | 0.95 (0.85 - 1.05) | 1649.88 (1319.74 - 2113.71) | 26.86 (20.27 - 35.82) | 4944.75 (3555.15 - 6923.36) | 26.55 (18.86 - 38.78) | -0.32 (-0.68 - 0.04) | 29522.54 (24208.12 - 37454.92) | 448.87 (349.37 - 585.16) | 87965.89 (65921.45 - 121489.98) | 433.46 (317.97 - 614.03) | -0.3 (-0.56 - -0.04) |
| Barbados | 25.6 (22.87 - 28.35) | 45.9 (35.77 - 58.56) | 52.61 (46.8 - 59.38) | 57.92 (44.97 - 71.75) | 0.66 (0.6 - 0.71) | 166.69 (138.73 - 201.1) | 309.8 (233.62 - 399.33) | 377.12 (319.46 - 455.6) | 413.44 (313.24 - 524.24) | 0.71 (0.6 - 0.82) | 14.51 (13.57 - 15.22) | 25.22 (23.17 - 26.95) | 27.76 (22.89 - 32.92) | 31.18 (25.22 - 37.25) | 0.79 (0.48 - 1.1) | 230.41 (215.9 - 244.86) | 402.08 (366.01 - 437.32) | 442.11 (367.75 - 514.66) | 488.81 (400.59 - 579.66) | 0.7 (0.45 - 0.96) |
| Belarus | 1381.22 (1232.82 - 1535.63) | 62.67 (44.24 - 83.65) | 1924 (1733.66 - 2128.82) | 67.46 (51.81 - 84.83) | 0.09 (0 - 0.18) | 11827.99 (9729.23 - 14259.95) | 542.79 (419.53 - 700.16) | 16404.03 (13830.19 - 19194.03) | 581 (462.24 - 719.66) | 0.07 (-0.06 - 0.21) | 550.91 (481.08 - 622.69) | 26.6 (22.98 - 30.51) | 787.95 (674.6 - 910.95) | 28.14 (23.67 - 32.69) | -0.1 (-0.28 - 0.07) | 9759.96 (8579.17 - 11052.45) | 458.49 (394.48 - 530.36) | 13588.79 (11746.09 - 15520.41) | 485.92 (411.76 - 566.37) | -0.11 (-0.29 - 0.07) |
| Belgium | 1935.8 (1742.39 - 2212.72) | 68.87 (53.91 - 85.23) | 3751.63 (3326.42 - 4226.53) | 88.61 (68.24 - 112.3) | 0.87 (0.84 - 0.91) | 15731.15 (13771.23 - 18447.16) | 559.58 (455.29 - 688.35) | 35099.21 (29877.61 - 40376.98) | 784.38 (634.69 - 959.46) | 1.15 (1.11 - 1.19) | 759.31 (687.93 - 800.77) | 26.55 (23.69 - 28.53) | 1301.79 (1095.06 - 1423.99) | 26.16 (22.13 - 28.74) | 0.31 (0.22 - 0.39) | 12939.04 (11949.12 - 13819.6) | 452.8 (405.04 - 495.19) | 22057.68 (19271.68 - 24358.04) | 478.01 (414.16 - 537.24) | 0.45 (0.38 - 0.52) |
| Belize | 6.74 (6.06 - 7.52) | 44.22 (32.82 - 57.07) | 22.85 (20.21 - 25.42) | 53.42 (43.06 - 65.04) | 0.6 (0.54 - 0.66) | 45.14 (37.79 - 54) | 295.61 (219.01 - 384.41) | 170.15 (141.91 - 203.43) | 381.41 (292.84 - 481.69) | 0.74 (0.63 - 0.85) | 3.13 (2.88 - 3.31) | 20.76 (18.8 - 22.42) | 11.7 (10.31 - 12.98) | 29.47 (25.43 - 33.31) | 1.09 (0.5 - 1.69) | 53.26 (49.65 - 57.07) | 351.22 (316.19 - 386.21) | 201.64 (178.91 - 224.19) | 482.91 (421.1 - 543.84) | 0.96 (0.44 - 1.49) |
| Benin | 167.81 (148.1 - 188.1) | 57.25 (44.31 - 71.94) | 430.39 (374.33 - 486.51) | 63.75 (49.4 - 79.27) | 0.35 (0.3 - 0.4) | 970.68 (788.82 - 1163.48) | 331.28 (247.03 - 431.74) | 2637.4 (2104.64 - 3236.97) | 391.36 (290.16 - 499.97) | 0.53 (0.45 - 0.61) | 75.47 (62.69 - 90.02) | 28.58 (22.61 - 36.12) | 170.77 (139.34 - 207.21) | 29.62 (23 - 37.11) | 0.22 (0.16 - 0.29) | 1267.76 (1068.12 - 1484.96) | 447.8 (361.7 - 552.96) | 2928.04 (2406.05 - 3524.91) | 462.94 (364.81 - 571.81) | 0.2 (0.14 - 0.26) |
| Bermuda | 6.36 (5.58 - 7.14) | 64.75 (49.57 - 82.72) | 17.29 (15.66 - 19.34) | 67.41 (52.43 - 83.79) | -0.1 (-0.18 - -0.02) | 41.33 (34.25 - 50.68) | 411.14 (307.42 - 535.94) | 125.86 (106.11 - 144.06) | 497.43 (385.46 - 615.58) | 0.3 (0.17 - 0.43) | 3.41 (2.69 - 3.91) | 36.4 (28.26 - 42.25) | 6.2 (5.01 - 7.7) | 22.93 (17.99 - 28.88) | -1.46 (-1.63 - -1.29) | 56.55 (44.77 - 64.24) | 575.02 (452 - 664.52) | 99.34 (82.85 - 120.22) | 380.5 (304.28 - 467.83) | -1.34 (-1.49 - -1.18) |
| Bhutan | 14.52 (12.06 - 17.17) | 47.19 (33.92 - 62.33) | 62.66 (53.32 - 73.38) | 65.95 (47.58 - 87.91) | 1.28 (1.23 - 1.33) | 102.41 (79.56 - 129.04) | 329.98 (236.33 - 440.77) | 477.11 (371.87 - 588.35) | 503.61 (361.42 - 661.59) | 1.6 (1.54 - 1.67) | 6.23 (4.46 - 8.04) | 26.29 (18.22 - 35.86) | 29.31 (24.41 - 34.52) | 33.13 (26.12 - 41.03) | 0.83 (0.8 - 0.86) | 122.57 (91.49 - 154.06) | 442.61 (319.61 - 586.27) | 496.51 (416.48 - 574.61) | 539.64 (433.61 - 660.89) | 0.72 (0.69 - 0.75) |
| Bolivia (Plurinational State of) | 275.57 (247.46 - 302.75) | 62.99 (50.92 - 76.48) | 1295.55 (1091.89 - 1495.52) | 93.84 (68.17 - 128.71) | 1.43 (1.38 - 1.48) | 2074.11 (1832.33 - 2333.82) | 438.79 (356.51 - 529.35) | 11426.73 (9278.31 - 14342.06) | 809.1 (610.79 - 1073.08) | 2.11 (2.02 - 2.2) | 155.97 (115.64 - 190.49) | 39.84 (28.91 - 50.86) | 465.66 (353.88 - 611.93) | 39.61 (28.94 - 52.93) | 0.11 (0.08 - 0.15) | 2815.77 (2141.76 - 3415.64) | 648.43 (476.6 - 816.01) | 8824.61 (6903.69 - 11289.42) | 678.09 (507.44 - 884.7) | 0.24 (0.21 - 0.27) |
| Bosnia and Herzegovina | 451.42 (392.12 - 507.69) | 72.91 (55.09 - 92.86) | 802.06 (699.11 - 901.54) | 71.37 (50.27 - 95.63) | -0.01 (-0.03 - 0.01) | 3302.21 (2648.84 - 3944.58) | 555.06 (421.36 - 701.33) | 6124.48 (4934.74 - 7541.14) | 544.04 (398.72 - 723.02) | 0.02 (-0.03 - 0.07) | 160.83 (142.22 - 177.5) | 30.7 (25.43 - 36.57) | 334.72 (273.61 - 405.59) | 30.06 (23.65 - 37.35) | -0.23 (-0.47 - 0) | 3113.04 (2761.83 - 3444.16) | 553.65 (464.01 - 647.85) | 5753.79 (4721.21 - 6837.73) | 514.13 (411.97 - 630.04) | -0.37 (-0.54 - -0.2) |
| Botswana | 31.98 (26.75 - 38.57) | 45.58 (33.24 - 61.33) | 105.04 (90.21 - 122.5) | 56.35 (42.38 - 74.8) | 0.73 (0.66 - 0.8) | 206.47 (159.41 - 266.72) | 290.89 (209.08 - 400.17) | 702.75 (568.06 - 878.03) | 377.86 (281.25 - 505.73) | 0.85 (0.72 - 0.97) | 13.4 (10.64 - 16.61) | 25.9 (19.56 - 34.1) | 35.16 (29.44 - 43.32) | 23.5 (18.31 - 30.53) | -0.15 (-0.36 - 0.06) | 257.77 (207.03 - 314.15) | 409.89 (316.09 - 527.65) | 651.39 (552.92 - 788.09) | 383.58 (306.82 - 486.25) | -0.11 (-0.3 - 0.07) |
| Brazil | 6113.95 (4976.37 - 7258.62) | 48.25 (32.05 - 66.45) | 22797.93 (19084.47 - 26081.32) | 55.19 (36.87 - 76.36) | 0.37 (0.31 - 0.42) | 45538.71 (37533.92 - 55018.92) | 348.69 (258.37 - 457.93) | 192802.6 (160870.58 - 229370.76) | 465.1 (351.99 - 597.75) | 0.81 (0.71 - 0.9) | 2721.61 (2477.98 - 2838.66) | 25.01 (22.4 - 26.41) | 9756.7 (8541.53 - 10440.41) | 24.82 (21.43 - 26.69) | 0.22 (0.15 - 0.3) | 48691.24 (45360.7 - 51516.3) | 404.35 (366.69 - 434.29) | 168175.47 (151781.59 - 181099.45) | 417.2 (368.3 - 456.48) | 0.26 (0.2 - 0.31) |
| Brunei Darussalam | 11.96 (10.85 - 13.17) | 87.45 (71.03 - 106.89) | 41.81 (37.14 - 47.87) | 91.31 (72.48 - 114.77) | 0.01 (-0.15 - 0.18) | 57.52 (46.84 - 69.54) | 409.03 (304.61 - 540.22) | 252.43 (212.54 - 308.45) | 533.33 (408.12 - 699.75) | 0.67 (0.48 - 0.86) | 3.65 (1.78 - 5.14) | 28.67 (13.87 - 41.69) | 9.37 (5.2 - 12.99) | 26.34 (14.08 - 37.33) | 0.46 (0.19 - 0.73) | 67.62 (39.55 - 91.32) | 499.03 (281.03 - 701.12) | 195.11 (124.86 - 256.9) | 462.03 (283.75 - 625.28) | 0.23 (0.06 - 0.41) |
| Bulgaria | 2052.26 (1861.4 - 2249.93) | 94.92 (76.02 - 114.43) | 2035.29 (1848.43 - 2263.24) | 74.99 (59.1 - 93.33) | -0.89 (-1.14 - -0.64) | 14487.65 (13117.06 - 16310.79) | 719.3 (601.66 - 843.64) | 14743.91 (12254.91 - 17282.61) | 534.13 (420.45 - 668.28) | -1.19 (-1.57 - -0.81) | 592.94 (558.98 - 628.01) | 39.43 (36.87 - 41.95) | 990.4 (885.37 - 1089.03) | 36.63 (32.35 - 40.59) | -0.18 (-0.31 - -0.05) | 11365.14 (10542.94 - 12215.21) | 632.29 (581.44 - 685.07) | 16290.92 (14609.64 - 17952.37) | 591.16 (523.84 - 656.64) | -0.25 (-0.37 - -0.14) |
| Burkina Faso | 331.48 (281.78 - 377.52) | 57.2 (43.71 - 73.64) | 768.32 (683.72 - 860.89) | 61.83 (49.32 - 79.02) | 0.23 (0.21 - 0.26) | 1885.65 (1483.38 - 2322.21) | 325.83 (234.1 - 433.47) | 4612.66 (3851.14 - 5523.61) | 374.03 (286.32 - 484.56) | 0.41 (0.35 - 0.47) | 136.23 (105.05 - 176.59) | 29.75 (22.3 - 40.01) | 309.09 (233.72 - 401.33) | 30.09 (22.04 - 40.33) | 0.15 (0.1 - 0.21) | 2472.59 (1949.84 - 3173.24) | 464.68 (356.66 - 611.85) | 5393.66 (4260.4 - 6902.2) | 469.27 (354.27 - 615.84) | 0.15 (0.1 - 0.2) |
| Burundi | 142.63 (123.57 - 165.35) | 42.92 (31.95 - 56.26) | 275.39 (233.99 - 320.64) | 45.53 (33.64 - 59.83) | 0.18 (0.17 - 0.2) | 863.81 (681.46 - 1103.91) | 257.69 (185.52 - 353.58) | 1817.09 (1473.77 - 2221.67) | 295.31 (213.9 - 393.77) | 0.43 (0.4 - 0.45) | 77.49 (57.03 - 99.57) | 26.87 (18.45 - 36.06) | 125.43 (76.21 - 170.8) | 26.19 (15.22 - 37.67) | -0.36 (-0.47 - -0.25) | 1450.97 (1089.08 - 1834.76) | 448.89 (320.2 - 590.6) | 2381.61 (1534.98 - 3100.94) | 429.96 (268.4 - 594.97) | -0.41 (-0.51 - -0.31) |
| Cabo Verde | 19.67 (16.97 - 22.59) | 46.83 (34.39 - 61.33) | 43.25 (38.11 - 48.95) | 66.03 (48.92 - 85.68) | 1.35 (1.23 - 1.46) | 135.08 (107.31 - 165.97) | 321.56 (235.2 - 422.36) | 305.41 (248.17 - 363.85) | 457.44 (339.66 - 587.59) | 1.33 (1.21 - 1.44) | 10.09 (8.17 - 12.42) | 23.55 (18.21 - 30.27) | 21.38 (16.32 - 25.46) | 32.38 (23.66 - 40.38) | 0.76 (0.49 - 1.04) | 158.03 (131.17 - 189.01) | 367.98 (293.69 - 458.4) | 327.94 (256.65 - 384.76) | 503.17 (381.41 - 617.86) | 0.76 (0.52 - 1.01) |
| Cambodia | 262.65 (227.81 - 301.55) | 43.47 (32.06 - 56.35) | 946.15 (813.89 - 1078.27) | 53.12 (39.26 - 68.13) | 0.71 (0.67 - 0.75) | 1817.94 (1478.19 - 2208.25) | 295.5 (222.07 - 387.55) | 7027.93 (5798 - 8386.71) | 397.32 (303.33 - 507.06) | 1.06 (0.99 - 1.12) | 137.01 (113.55 - 167.56) | 28.19 (21.85 - 36.67) | 425.03 (338.01 - 521.49) | 30.96 (23.63 - 39.95) | 0.39 (0.26 - 0.52) | 2648.35 (2247.51 - 3130.44) | 471.19 (373.84 - 595.23) | 8011.63 (6338.82 - 9724.15) | 505.35 (391.57 - 643.89) | 0.3 (0.18 - 0.41) |
| Cameroon | 354.12 (311.33 - 395.97) | 63.46 (49.88 - 78.11) | 1076.29 (924.43 - 1244.1) | 69.56 (53.24 - 89.34) | 0.28 (0.22 - 0.34) | 2036.03 (1663.25 - 2405.95) | 359.58 (265.98 - 460.3) | 6544.34 (5284.66 - 7897.68) | 424.08 (311.27 - 562.51) | 0.52 (0.43 - 0.62) | 176.36 (147.55 - 209.24) | 38.24 (30.44 - 47.4) | 463.25 (359.42 - 594.61) | 37 (27.78 - 48.41) | -0.06 (-0.1 - -0.02) | 3057.08 (2586.63 - 3590.34) | 588.67 (476.72 - 723.22) | 8196.43 (6422.29 - 10414.18) | 577.68 (438.12 - 746.69) | -0.02 (-0.07 - 0.02) |
| Canada | 4598.87 (4327.99 - 4920.82) | 80.5 (68.17 - 93.74) | 14750.02 (13940.33 - 15463.39) | 113.38 (96.58 - 131.28) | 1.14 (1.05 - 1.23) | 38931.11 (36447 - 41841.54) | 688.52 (607.93 - 773.58) | 146059.9 (136816.55 - 155136.01) | 1098.8 (981 - 1209.78) | 1.51 (1.37 - 1.64) | 1530.73 (1407.95 - 1597.63) | 27.88 (25.02 - 29.78) | 4002.1 (3499.34 - 4288.67) | 28.36 (24.54 - 30.87) | 0.28 (0.1 - 0.47) | 27507.33 (25463.13 - 29521.92) | 489.29 (438.05 - 536.39) | 73835.48 (65780.97 - 80687.7) | 543.5 (472.66 - 609.22) | 0.51 (0.34 - 0.68) |
| Central African Republic | 60.27 (51.23 - 71.06) | 43.86 (32.35 - 56.52) | 112.56 (94.89 - 131.86) | 44.88 (33.58 - 58.45) | 0.09 (0.06 - 0.13) | 380.53 (303.54 - 469.33) | 267.48 (191.52 - 358.49) | 753.78 (597.44 - 938.31) | 293.31 (213.14 - 394.45) | 0.27 (0.2 - 0.34) | 27.29 (21.42 - 32.3) | 27.81 (20.4 - 35.73) | 45.69 (34.84 - 56.65) | 26.39 (18.38 - 35.06) | -0.11 (-0.15 - -0.08) | 584.24 (453.38 - 698.16) | 472.27 (354.2 - 594.44) | 978.3 (760.99 - 1207) | 446.95 (325.06 - 578.66) | -0.15 (-0.18 - -0.12) |
| Chad | 214.25 (186 - 244.28) | 50.59 (38.5 - 65.58) | 416.63 (361.94 - 483.58) | 56.78 (42.92 - 75.59) | 0.43 (0.38 - 0.48) | 1257.25 (997.63 - 1503.22) | 297.84 (217.31 - 394.79) | 2551.58 (2035.76 - 3140.29) | 345.47 (255.26 - 469.25) | 0.58 (0.47 - 0.69) | 85.78 (66.09 - 105.49) | 23.52 (17.56 - 30.89) | 181.9 (143.07 - 230.2) | 30.22 (22.99 - 39.26) | 0.93 (0.84 - 1.03) | 1510.83 (1225.59 - 1815.2) | 374.98 (290.32 - 479.91) | 3250.48 (2594.37 - 4014.53) | 477.6 (367.91 - 609.61) | 0.9 (0.82 - 0.98) |
| Chile | 1115.58 (1020 - 1214.31) | 70.82 (56.79 - 86.29) | 3903.56 (3460.09 - 4312.54) | 87.55 (67.6 - 106.64) | 0.9 (0.82 - 0.97) | 7536.43 (6547.18 - 8628.18) | 486.42 (391.18 - 592.26) | 30212.66 (25280.82 - 35404.05) | 676.16 (527.77 - 818.48) | 1.38 (1.27 - 1.5) | 448.29 (419.87 - 466.02) | 31.06 (28.4 - 33.03) | 1258.08 (1118.86 - 1333.13) | 28.01 (24.58 - 30.21) | 0.03 (-0.12 - 0.17) | 7749.76 (7259.16 - 8171.2) | 508.09 (464.68 - 548.67) | 21562.22 (19585.63 - 23261.63) | 482.28 (426.1 - 532.23) | 0.14 (0.03 - 0.26) |
| China | 79629.26 (64945.26 - 94984.56) | 69.13 (46.56 - 95.63) | 433906.19 (352906.91 - 522735.97) | 123.08 (78.02 - 175.56) | 2.07 (1.98 - 2.16) | 575715.59 (470402.58 - 700145.41) | 498.92 (370.28 - 658.98) | 4628799.09 (3873746.83 - 5514727.36) | 1332.36 (1028.75 - 1706.51) | 3.23 (3.11 - 3.35) | 31457.09 (27704.59 - 34984.43) | 35.48 (29.59 - 40.51) | 90516.31 (74675.5 - 106280.31) | 29.35 (23.9 - 35.3) | -0.75 (-0.91 - -0.58) | 625936.26 (549752.88 - 698048.36) | 587.82 (499.68 - 671.37) | 2027919.24 (1706010.45 - 2357579.74) | 602.49 (498.5 - 714.8) | -0.02 (-0.13 - 0.1) |
| Colombia | 1128.36 (984.44 - 1262.35) | 44.93 (34.2 - 56.97) | 5248.36 (4416.94 - 5987.31) | 55.67 (41.38 - 72.42) | 0.5 (0.45 - 0.55) | 8880.53 (7266.09 - 10562.3) | 344.45 (260.84 - 432.23) | 47504.45 (39264.62 - 56354.12) | 505.16 (389.14 - 635.94) | 1 (0.92 - 1.07) | 513.55 (477.59 - 534.42) | 22.53 (20.51 - 24.06) | 2081.11 (1743.92 - 2422.41) | 22.44 (18.63 - 26.17) | -0.34 (-0.45 - -0.23) | 9147.67 (8593.53 - 9645.47) | 375.71 (341.33 - 407.82) | 36546.34 (31690.08 - 41791.23) | 396.37 (335.21 - 458.35) | -0.14 (-0.23 - -0.05) |
| Comoros | 11.5 (9.94 - 13.26) | 45.2 (34.34 - 58.75) | 33.37 (28.07 - 38.52) | 48.85 (36.33 - 64.67) | 0.29 (0.26 - 0.31) | 71.62 (58.13 - 87.54) | 274.64 (201.86 - 365.37) | 221.13 (170.11 - 278.97) | 321.7 (229.53 - 436.19) | 0.53 (0.49 - 0.56) | 5.23 (4.14 - 6.5) | 25.39 (18.81 - 33.49) | 15.72 (11.06 - 21.55) | 26.56 (18 - 38.13) | 0.06 (0 - 0.12) | 101.98 (81.78 - 126.19) | 421.88 (320.02 - 543.96) | 281.31 (208.76 - 371.57) | 431.91 (306.45 - 594.59) | -0.01 (-0.07 - 0.05) |
| Congo | 68.49 (57.6 - 79.41) | 49.09 (37.72 - 62.3) | 176.56 (148.88 - 205.87) | 53.95 (39.94 - 70.18) | 0.34 (0.29 - 0.39) | 442.45 (350.6 - 542.12) | 308.64 (225.26 - 399.53) | 1239.81 (993.11 - 1564.52) | 374.76 (275.46 - 498.98) | 0.59 (0.48 - 0.7) | 32.05 (26.43 - 39.43) | 30.6 (23.91 - 39.27) | 69.78 (57.34 - 85.53) | 28.18 (21.72 - 37.1) | -0.37 (-0.45 - -0.3) | 652.28 (531.98 - 788.64) | 508.37 (403.64 - 638.89) | 1377.2 (1138.99 - 1651.74) | 467.36 (369.46 - 592.56) | -0.39 (-0.46 - -0.31) |
| Cook Islands | 1.24 (1.05 - 1.43) | 67.38 (49.36 - 87.71) | 3.08 (2.63 - 3.75) | 68.13 (48.5 - 92.74) | 0.01 (-0.04 - 0.07) | 9.12 (7.48 - 11.27) | 510.52 (380.26 - 668.97) | 25.69 (20.83 - 32.03) | 574.59 (433.2 - 764.63) | 0.39 (0.31 - 0.46) | 0.54 (0.46 - 0.63) | 34.26 (27.7 - 41.59) | 1.03 (0.8 - 1.35) | 23.66 (17.79 - 31.78) | -1.22 (-1.29 - -1.15) | 10.32 (8.92 - 11.75) | 591.63 (487.36 - 707.27) | 19.12 (15.54 - 24.44) | 427.94 (333.67 - 558.23) | -1.07 (-1.13 - -1) |
| Costa Rica | 141.39 (124.01 - 158.19) | 51.74 (37.89 - 66.39) | 580.05 (498.61 - 664.57) | 62.42 (45.04 - 83.12) | 0.53 (0.45 - 0.62) | 1136.8 (927.1 - 1333.78) | 412.8 (307.94 - 519.15) | 5215.42 (4398.5 - 6514.95) | 560.79 (424.16 - 725.6) | 0.86 (0.75 - 0.98) | 57.91 (52.96 - 61.16) | 21.78 (19.56 - 23.46) | 204.56 (176.27 - 227.61) | 22.34 (19.04 - 24.97) | 0.09 (-0.12 - 0.31) | 1008.56 (928.67 - 1085.79) | 371.08 (331.77 - 409.16) | 3617.79 (3231.34 - 4013.59) | 396.14 (343.05 - 449.18) | 0.17 (0.01 - 0.33) |
| Côte d'Ivoire | 636.76 (556.18 - 720.23) | 64.52 (49.59 - 81.91) | 1148.27 (1003.44 - 1283.64) | 69.55 (51.94 - 87.3) | 0.23 (0.21 - 0.25) | 4873.72 (4029.95 - 5840.85) | 508 (395.56 - 645.92) | 9512.65 (7593.65 - 11363.5) | 559.7 (422.17 - 702.17) | 0.24 (0.2 - 0.29) | 294.36 (277.34 - 310.8) | 34.21 (31.86 - 36.35) | 519.38 (458.54 - 562.22) | 28.91 (25.29 - 31.68) | -0.49 (-0.63 - -0.35) | 4989.81 (4658.5 - 5316.93) | 555.18 (512.22 - 599.67) | 8444.61 (7622.48 - 9161.71) | 482.86 (427.67 - 534.68) | -0.35 (-0.46 - -0.25) |
| Croatia | 674.37 (605.68 - 742.02) | 39.58 (31.19 - 49.33) | 1698.72 (1554.42 - 1844.24) | 48.37 (37.88 - 61.14) | 0.67 (0.64 - 0.7) | 4509.19 (3787.1 - 5254.77) | 262.58 (198.68 - 329.78) | 13062.94 (11333.98 - 15082.28) | 376.55 (295.99 - 465.65) | 1.17 (1.11 - 1.23) | 378.01 (356.61 - 391.1) | 22.54 (20.86 - 23.93) | 949.19 (832.89 - 1060.95) | 26.35 (22.85 - 29.8) | 0.47 (0.4 - 0.54) | 6177.52 (5824.64 - 6498.28) | 356.84 (328.61 - 387.34) | 15203.61 (13509.32 - 16931.53) | 432.1 (376.48 - 488.81) | 0.61 (0.54 - 0.69) |
| Cuba | 136.57 (112.45 - 165.16) | 96.5 (68.07 - 135.82) | 377.06 (314.88 - 458.77) | 99.43 (70.1 - 137.1) | 0 (-0.03 - 0.03) | 937.65 (738.51 - 1161.97) | 702.91 (511.18 - 958.95) | 3169.42 (2493.81 - 3931.25) | 849.87 (631.63 - 1143.08) | 0.54 (0.48 - 0.6) | 66.34 (55.58 - 76.19) | 75.4 (58.5 - 96.35) | 94.53 (81.11 - 108.11) | 27.64 (21.99 - 33.53) | -3.33 (-3.63 - -3.02) | 1095.73 (938.47 - 1254.19) | 1044.87 (826.83 - 1303.62) | 1749.68 (1520.09 - 1991.29) | 481.75 (394.69 - 576.14) | -2.59 (-2.82 - -2.36) |
| Cyprus | 1368.08 (1229.12 - 1504.48) | 55.81 (42.82 - 69.11) | 2547.84 (2225.89 - 2830.83) | 63.7 (47.75 - 81.29) | 0.39 (0.37 - 0.42) | 10952.02 (8999.47 - 12803.46) | 448.74 (352.39 - 547.24) | 21632.41 (18530.21 - 25325.67) | 531.49 (422.79 - 662.18) | 0.48 (0.43 - 0.53) | 608.78 (578.58 - 630.41) | 25.39 (23.71 - 26.74) | 1129.66 (985.57 - 1246.07) | 27.16 (23.54 - 30.15) | 0.45 (0.33 - 0.58) | 10786.15 (10172.86 - 11408.57) | 440.8 (405.91 - 477.33) | 19096.39 (16985.43 - 21029.64) | 459.27 (400.88 - 514.91) | 0.29 (0.2 - 0.38) |
| Czechia | 268.14 (232.78 - 304.6) | 62.73 (48.95 - 78.59) | 902.3 (796.51 - 1015.01) | 69.09 (54.24 - 85.97) | 0.32 (0.26 - 0.38) | 1557.65 (1264.44 - 1894.97) | 356.39 (265.1 - 460.88) | 5493.8 (4449.44 - 6517.97) | 421.84 (315.74 - 539.32) | 0.56 (0.46 - 0.66) | 112.56 (94.38 - 131.32) | 34.97 (28.09 - 42.68) | 363.13 (302.31 - 449.01) | 35.05 (26.89 - 44.52) | 0.09 (0.01 - 0.17) | 2080.73 (1763.39 - 2416.52) | 542.4 (437.92 - 653.59) | 6480.84 (5295.95 - 8003.68) | 547.33 (426.4 - 686.05) | 0.09 (0.02 - 0.16) |
| Democratic People's Republic of Korea | 1275.15 (1088.72 - 1505.59) | 58.62 (41.64 - 79.87) | 4637.56 (3904.83 - 5510.91) | 87.55 (58.3 - 121.52) | 1.38 (1.29 - 1.46) | 9689.92 (7909.46 - 12044.39) | 441.44 (316.59 - 590.29) | 45711.17 (36856.87 - 54146.45) | 877.7 (651.29 - 1137.8) | 2.39 (2.31 - 2.48) | 558 (437.35 - 682.07) | 31.01 (22.97 - 40.57) | 1387.93 (1011.51 - 1720.89) | 28.15 (19.57 - 38.15) | -0.12 (-0.23 - -0.02) | 11051.32 (8900.79 - 13388.77) | 538.68 (410.76 - 686.68) | 29365.06 (23073.55 - 35189.49) | 565.58 (423.95 - 726.45) | 0.35 (0.26 - 0.44) |
| Democratic Republic of the Congo | 786.42 (648.63 - 955.68) | 41.25 (29.33 - 54.71) | 1955.98 (1645.84 - 2337.08) | 42.84 (30.52 - 58.24) | 0.16 (0.05 - 0.27) | 5273.72 (4054.29 - 6817.19) | 268.84 (191.17 - 370.28) | 14217.41 (11232.94 - 17729.86) | 311.1 (224.71 - 419.92) | 0.48 (0.33 - 0.63) | 348.73 (276.59 - 431.49) | 25.41 (18.74 - 34.11) | 850.99 (578.34 - 1125.37) | 24.75 (15.83 - 34.74) | -0.01 (-0.19 - 0.16) | 7268.93 (5887.82 - 8864.94) | 421.72 (321.15 - 547.84) | 16833.29 (12032.05 - 21292.44) | 414.59 (282.55 - 562.3) | 0.01 (-0.16 - 0.18) |
| Denmark | 811.85 (702.98 - 905.61) | 53.98 (40.28 - 68.16) | 1892.38 (1589.78 - 2240.04) | 84.47 (61.29 - 111.5) | 1.58 (1.51 - 1.65) | 6573.03 (5403.58 - 7547.42) | 437.19 (333.62 - 537.38) | 16983.43 (13136.35 - 20891.17) | 746.93 (555.62 - 966.57) | 1.9 (1.81 - 1.98) | 314.6 (288.96 - 331.18) | 19.87 (17.81 - 21.59) | 653.5 (574.09 - 700.77) | 27.25 (23.66 - 29.6) | 1.2 (1.05 - 1.35) | 5410.89 (5025.99 - 5809.84) | 348 (311.44 - 385.49) | 11247.23 (10126.31 - 12320.8) | 480.48 (421.35 - 539.64) | 1.21 (1.09 - 1.33) |
| Djibouti | 6.04 (4.95 - 7.2) | 40.83 (29.62 - 54.37) | 36.09 (30.59 - 41.81) | 49.37 (36.58 - 64.54) | 0.67 (0.64 - 0.7) | 39.39 (30.33 - 49.56) | 258.27 (184.01 - 348.06) | 239.41 (196.11 - 290.78) | 322.63 (238.41 - 424.78) | 0.74 (0.68 - 0.79) | 2.2 (1.52 - 2.99) | 20.25 (13.6 - 29.64) | 13.1 (9.14 - 19.71) | 24.52 (16.47 - 38.37) | 0.57 (0.49 - 0.65) | 45.18 (33.35 - 59.47) | 337.99 (238.65 - 473.34) | 263.28 (193.29 - 376.53) | 404.25 (285.28 - 602.02) | 0.52 (0.45 - 0.59) |
| Dominica | 5.03 (4.41 - 5.61) | 49.29 (38.38 - 63.06) | 7.8 (6.82 - 8.63) | 58.1 (44.53 - 73.22) | 0.5 (0.46 - 0.54) | 31.17 (25.14 - 37.33) | 304.29 (222.56 - 394.96) | 53.14 (44.42 - 62.19) | 383.39 (286.37 - 489.86) | 0.6 (0.49 - 0.7) | 3.76 (3.12 - 4.65) | 37.51 (29.36 - 47.6) | 4.85 (3.96 - 5.86) | 38.55 (29.43 - 48.43) | -0.06 (-0.14 - 0.02) | 60.79 (51.09 - 73.74) | 584.5 (465.5 - 736.83) | 80.76 (67.03 - 96.23) | 611.41 (479.15 - 755.84) | -0.01 (-0.07 - 0.05) |
| Dominican Republic | 210.98 (185.33 - 239.94) | 40.65 (30.12 - 53.16) | 853.38 (769.6 - 937.72) | 52.96 (41.04 - 67.62) | 0.76 (0.71 - 0.81) | 1455.75 (1203.87 - 1782.12) | 260.96 (194.35 - 346.88) | 6050.23 (5078.33 - 7058.29) | 372.6 (285.37 - 477.18) | 0.99 (0.87 - 1.11) | 123.8 (105.96 - 148.97) | 27.2 (21.9 - 34.39) | 418.9 (331.53 - 529.29) | 26.38 (19.78 - 34.98) | 0.36 (0.09 - 0.64) | 2041.17 (1754.3 - 2414.13) | 404.99 (331.18 - 504.35) | 6900.24 (5620.17 - 8511.7) | 430.33 (332.82 - 558.03) | 0.54 (0.34 - 0.74) |
| Ecuador | 377.84 (329.47 - 445.91) | 48.26 (34.24 - 65.95) | 2153.67 (1813.46 - 2467.03) | 80.36 (57.36 - 108.11) | 1.62 (1.59 - 1.65) | 3239.57 (2651.87 - 4029.53) | 400.82 (299.95 - 541.41) | 20990.82 (17012.17 - 24978.56) | 778.79 (594.59 - 988.53) | 2.06 (1.99 - 2.14) | 170.77 (160.58 - 177.38) | 23.37 (21.59 - 24.79) | 706.96 (584.51 - 853.58) | 28.04 (22.95 - 33.81) | 1.09 (0.67 - 1.52) | 2975.18 (2776.2 - 3177.82) | 385.07 (350.11 - 422.55) | 12855.73 (10873.64 - 15305.32) | 489.19 (404.79 - 588.27) | 1.03 (0.67 - 1.4) |
| Egypt | 2262.72 (1922.62 - 2594.66) | 67.84 (50.89 - 87.07) | 7592.59 (6486.11 - 8571.45) | 92.83 (69.1 - 119.27) | 1.04 (1.01 - 1.07) | 15203.7 (12411.67 - 18396.56) | 464.8 (347.99 - 597.81) | 57064.67 (47878.3 - 67934.37) | 743.87 (577.32 - 932.54) | 1.5 (1.46 - 1.54) | 1066.72 (945.17 - 1203.64) | 44.13 (35.46 - 54.54) | 1909.48 (1620.74 - 2263.27) | 35.8 (28.96 - 44) | -0.38 (-0.5 - -0.26) | 20225.38 (17770.04 - 22150.04) | 692.44 (561.05 - 830.49) | 39920.31 (34488.05 - 46779.33) | 605.57 (498 - 728.08) | -0.32 (-0.41 - -0.23) |
| El Salvador | 214.34 (190.79 - 237.73) | 45.96 (34.46 - 60.26) | 667.24 (586.25 - 753.17) | 61.29 (45 - 79.66) | 0.77 (0.66 - 0.87) | 1592.56 (1334.51 - 1893.31) | 338.75 (256.97 - 437.98) | 5604.33 (4635.86 - 6490.42) | 518.95 (394.63 - 656.11) | 1.08 (0.9 - 1.25) | 111.64 (96.96 - 130.82) | 24.67 (20.4 - 30.39) | 294.18 (238.94 - 351.4) | 25.69 (19.94 - 31.89) | 0.08 (-0.04 - 0.2) | 1874.62 (1687.64 - 2139.97) | 408.6 (346.1 - 490.81) | 4842.13 (4101.78 - 5631.47) | 440.97 (354.18 - 535.78) | 0.14 (0.02 - 0.25) |
| Equatorial Guinea | 10.61 (8.96 - 12.21) | 42.95 (31.45 - 56.42) | 37.74 (31.91 - 43.48) | 60.26 (43.12 - 79.43) | 1.31 (1.25 - 1.38) | 68.77 (55.21 - 83.1) | 270.13 (196.6 - 359.34) | 262.74 (207.29 - 321) | 420.44 (301.36 - 561.22) | 1.63 (1.58 - 1.68) | 5.17 (4.13 - 6.28) | 26.31 (19.39 - 34.54) | 14.99 (11.2 - 19.75) | 29.59 (21.27 - 39.91) | 0.48 (0.43 - 0.53) | 104.23 (83.39 - 125.95) | 446.28 (337.89 - 569.53) | 282.5 (215.6 - 369.21) | 489.15 (363.24 - 648.97) | 0.37 (0.31 - 0.43) |
| Eritrea | 46.43 (38.87 - 55.34) | 41.34 (30.69 - 53.91) | 151.07 (129 - 177) | 47.64 (35.65 - 61.56) | 0.51 (0.48 - 0.53) | 298.44 (238.71 - 366.82) | 251.42 (180.89 - 337.45) | 989.55 (793.38 - 1193.98) | 303.85 (222.65 - 395.8) | 0.59 (0.56 - 0.62) | 18.93 (15.53 - 23.2) | 24.25 (17.55 - 32.48) | 71.25 (54.42 - 89.36) | 30.07 (21.39 - 40.63) | 0.67 (0.58 - 0.77) | 419.35 (348.89 - 513.13) | 412.2 (310.65 - 534.71) | 1386.18 (1089.9 - 1706.35) | 486.05 (358.55 - 638.03) | 0.51 (0.43 - 0.59) |
| Estonia | 219.99 (192.33 - 255.9) | 61.75 (43.79 - 80.85) | 298.77 (263.41 - 339.62) | 61.51 (46.42 - 77.64) | -0.05 (-0.19 - 0.08) | 2143.61 (1798 - 2615.27) | 603.23 (472.83 - 768.94) | 2875.86 (2425.13 - 3365.06) | 565.9 (447.04 - 695.5) | -0.2 (-0.41 - 0.01) | 85.51 (79.25 - 92.61) | 25.08 (22.74 - 27.42) | 149.18 (127.95 - 166.09) | 25.43 (21.64 - 28.64) | -0.15 (-0.27 - -0.02) | 1570.75 (1428.78 - 1727.82) | 447.61 (398.9 - 500) | 2380.77 (2103.41 - 2644.47) | 434.29 (373.9 - 493.95) | -0.25 (-0.34 - -0.17) |
| Eswatini | 16.86 (14.47 - 19.89) | 47.01 (34.54 - 63.38) | 37.97 (32.45 - 45.05) | 55.24 (41.3 - 72.44) | 0.53 (0.48 - 0.58) | 109.97 (88.36 - 136.67) | 302.46 (221.52 - 410.02) | 249.13 (198.51 - 305.92) | 363.13 (267.3 - 480.52) | 0.54 (0.42 - 0.66) | 8.44 (6.31 - 10.97) | 28.75 (20.93 - 38.72) | 17.63 (12.15 - 24.12) | 31.82 (21.53 - 44.75) | 0.84 (0.53 - 1.16) | 156.54 (119.17 - 197.78) | 463.37 (344.82 - 607.5) | 345 (246.43 - 467.39) | 529.2 (369.4 - 731.25) | 0.82 (0.53 - 1.12) |
| Ethiopia | 1098.87 (887.91 - 1329.53) | 44.27 (30.44 - 59.93) | 2821.44 (2314.28 - 3307.19) | 47.23 (32.6 - 63.68) | 0.18 (0.15 - 0.2) | 6777.51 (5422.49 - 8388.39) | 265.03 (192.43 - 355.21) | 19221.93 (15565.97 - 23142.99) | 322.21 (237.18 - 420.18) | 0.59 (0.54 - 0.63) | 506.23 (415.45 - 670.56) | 26.59 (19.88 - 37.09) | 1115.13 (797.75 - 1707.4) | 21.36 (14.62 - 33.39) | -0.95 (-1.06 - -0.83) | 10576.26 (8877.08 - 13878.14) | 453.78 (353.88 - 613.45) | 20300.92 (15030.45 - 29577.2) | 355.94 (255.21 - 532.48) | -1.04 (-1.16 - -0.92) |
| Fiji | 29.2 (24.7 - 33.62) | 65.84 (48.55 - 87.54) | 71.31 (58.5 - 84.84) | 65.54 (47.35 - 88.37) | 0.1 (0.06 - 0.14) | 202.11 (161.92 - 242.8) | 461.81 (337.66 - 608.67) | 523.32 (405.18 - 643.39) | 487.7 (351.73 - 653.14) | 0.27 (0.21 - 0.34) | 11.61 (10.03 - 13.5) | 32.59 (26.67 - 40.03) | 26.04 (20.77 - 31.35) | 31.46 (24.73 - 39.26) | -0.46 (-0.66 - -0.27) | 234.18 (205.02 - 269.32) | 565.59 (469.88 - 682.56) | 526.89 (431.49 - 630.73) | 537.74 (430.51 - 660.71) | -0.43 (-0.59 - -0.28) |
| Finland | 851.9 (760.36 - 973.46) | 66.4 (51.93 - 82.58) | 2241.08 (1903.93 - 2729.66) | 94.2 (67.55 - 123.71) | 1.27 (1.19 - 1.35) | 7034.71 (6103.73 - 8226.98) | 551.12 (448.3 - 681.31) | 20979.62 (17226.87 - 26478.16) | 843.43 (635.83 - 1096.82) | 1.55 (1.45 - 1.65) | 318.68 (289.32 - 334.57) | 24.78 (22.12 - 26.63) | 799.07 (672.26 - 868.88) | 29.35 (24.74 - 32.37) | 0.8 (0.71 - 0.89) | 5658.3 (5262.2 - 6032.47) | 436.43 (391.71 - 479.6) | 13662.43 (12065.83 - 15001.82) | 529.82 (455.92 - 597.01) | 0.84 (0.76 - 0.93) |
| France | 11499.21 (10481.57 - 12619.71) | 74.85 (58.38 - 93.71) | 24691.51 (20650.52 - 27711.92) | 96.26 (65.75 - 128.04) | 0.86 (0.81 - 0.92) | 92425.08 (80761.97 - 103280.17) | 595.7 (488.41 - 713.96) | 231777.71 (176623.3 - 270621.72) | 845.84 (605.1 - 1072.02) | 1.2 (1.12 - 1.28) | 4398.44 (4034.69 - 4608.96) | 26.85 (24.29 - 28.56) | 8217.87 (6983.6 - 8942.89) | 25.99 (22 - 28.6) | 0.09 (-0.01 - 0.18) | 72169.21 (66765.98 - 77140.03) | 452.13 (406.01 - 495.95) | 136288.89 (120418.7 - 150921.93) | 474.91 (407.88 - 540.7) | 0.32 (0.24 - 0.4) |
| Gabon | 47.08 (41.14 - 54.46) | 54.95 (41.29 - 70.79) | 88.3 (77.74 - 102.04) | 63.04 (46.9 - 82.81) | 0.44 (0.38 - 0.49) | 299.09 (247.15 - 369.37) | 344.92 (253.03 - 453.79) | 600.38 (500.21 - 728.77) | 428.59 (321.56 - 570.89) | 0.64 (0.53 - 0.75) | 26.88 (20.2 - 34.7) | 36.15 (25.82 - 48.08) | 37.73 (30.15 - 46.97) | 33.97 (26.19 - 43.81) | -0.27 (-0.34 - -0.21) | 487.82 (373.06 - 617.44) | 593.92 (434.47 - 777.86) | 709.18 (580.66 - 870.55) | 558.12 (440.78 - 709.53) | -0.26 (-0.31 - -0.21) |
| Gambia | 24.94 (21.62 - 28.29) | 55.53 (42.32 - 70.68) | 90.64 (77.96 - 103.47) | 68.52 (52.23 - 86.2) | 0.74 (0.69 - 0.78) | 146.33 (116.2 - 173.89) | 326.82 (238.18 - 423.74) | 548.1 (440.5 - 674.29) | 415.76 (302.92 - 548.38) | 0.82 (0.75 - 0.9) | 9.45 (6.95 - 12.05) | 26.27 (18.98 - 35.23) | 37.64 (27.76 - 46.98) | 32.46 (23.37 - 41.89) | 0.76 (0.71 - 0.82) | 172.26 (132.21 - 216.96) | 415.34 (308.94 - 542.88) | 643.22 (484.73 - 785.59) | 510.41 (377.75 - 646.7) | 0.73 (0.66 - 0.79) |
| Georgia | 571.82 (507.27 - 644.22) | 55.07 (40.6 - 73.55) | 569.57 (521.38 - 622.49) | 53.42 (42.33 - 66.34) | -0.14 (-0.2 - -0.09) | 4598.25 (3823.86 - 5538.55) | 450.9 (345.67 - 584.87) | 4349.9 (3766.17 - 5079.48) | 405.33 (328.16 - 503.34) | -0.45 (-0.53 - -0.36) | 226.92 (198.39 - 264.06) | 23.83 (20.32 - 27.86) | 270.29 (238.45 - 300.05) | 24.41 (21.44 - 27.23) | 0.47 (0.24 - 0.69) | 4162.74 (3679.01 - 4735.17) | 420.17 (362.51 - 488.11) | 4538.45 (4074.14 - 5000.67) | 420.75 (371.85 - 469.88) | 0.23 (0.08 - 0.37) |
| Germany | 14310.66 (13494.12 - 14975.82) | 62.73 (52.1 - 73.34) | 43831.68 (41985.9 - 45651.2) | 118.79 (105.04 - 132.84) | 1.97 (1.82 - 2.11) | 115747.2 (107848.7 - 122629.9) | 505.5 (447.2 - 564.83) | 410909.71 (395295.58 - 428567.73) | 1041.18 (963.56 - 1122.08) | 2.22 (2.03 - 2.41) | 5774.64 (5256.58 - 6079.19) | 24.09 (21.51 - 25.89) | 12396.69 (10471.63 - 13393.61) | 28.7 (24.3 - 31.41) | 0.8 (0.57 - 1.03) | 98135.53 (91090.17 - 104429.59) | 414.22 (371.42 - 453.94) | 218597.99 (192440.24 - 238952.9) | 543.76 (473.8 - 608.6) | 1.01 (0.81 - 1.21) |
| Ghana | 352.22 (305.67 - 404.99) | 45.73 (34.6 - 59.32) | 1201.05 (1034.17 - 1381.69) | 56.7 (43.46 - 72.02) | 0.68 (0.63 - 0.73) | 2246.04 (1841.18 - 2680.9) | 291.89 (215.85 - 385.26) | 7783.26 (6349.41 - 9463.87) | 374.09 (280.54 - 485.46) | 0.71 (0.61 - 0.81) | 138.28 (113.91 - 164.24) | 22.62 (17.66 - 28.12) | 469.78 (379.2 - 564.93) | 27.71 (21.12 - 35.24) | 0.77 (0.7 - 0.84) | 2553.85 (2129.87 - 3012) | 360.76 (289.4 - 441.33) | 8486.58 (6900.15 - 10108.84) | 439.81 (339.51 - 546.78) | 0.74 (0.68 - 0.81) |
| Greece | 1992.94 (1722.24 - 2267.33) | 73.82 (54.2 - 97.89) | 3901.83 (3451.89 - 4491.89) | 87.24 (64.1 - 116.17) | 0.49 (0.42 - 0.55) | 16231.61 (13292.55 - 19612.75) | 605.64 (463.99 - 781.79) | 36784.5 (30625.22 - 44774.54) | 757.89 (591.61 - 962.37) | 0.65 (0.57 - 0.74) | 707.65 (653.25 - 742.71) | 27.2 (24.66 - 29.11) | 1807.29 (1563.92 - 1950.83) | 31.69 (27.5 - 34.43) | 0.45 (0.33 - 0.57) | 12278.5 (11338.46 - 13185.66) | 457.61 (411.19 - 506.49) | 28057.34 (24860.69 - 30824.35) | 545.08 (477.41 - 608.9) | 0.53 (0.42 - 0.64) |
| Greenland | 3.43 (2.93 - 4.03) | 82.54 (59.02 - 111.37) | 9.74 (8.32 - 11.04) | 93.85 (70.98 - 118.66) | 0.45 (0.42 - 0.47) | 21.34 (16.98 - 26.41) | 520.57 (370.73 - 711.82) | 65.13 (53.64 - 77.84) | 649.65 (488.39 - 824.62) | 0.73 (0.7 - 0.76) | 1.48 (0.91 - 1.99) | 45.57 (27.67 - 62.27) | 3.06 (2.28 - 3.92) | 39.47 (28.67 - 51.97) | -0.12 (-0.33 - 0.08) | 28.1 (18.44 - 36.92) | 747.17 (482.02 - 995.86) | 60.21 (46.3 - 73.9) | 660.31 (496.72 - 845.07) | -0.08 (-0.26 - 0.09) |
| Grenada | 5.83 (5.29 - 6.55) | 42.08 (31.57 - 54.93) | 9.61 (8.45 - 10.79) | 55.01 (42.9 - 70.24) | 0.75 (0.69 - 0.81) | 35.94 (29.73 - 43.57) | 270.59 (199.54 - 357.87) | 68.32 (57.22 - 83.38) | 380.59 (287.1 - 497.55) | 0.94 (0.83 - 1.05) | 3.39 (3.09 - 3.66) | 23.03 (20.55 - 25.22) | 5.41 (4.82 - 5.93) | 35.07 (30.66 - 38.97) | 1.37 (0.83 - 1.91) | 53.29 (48.75 - 57.57) | 380.05 (340.16 - 418.61) | 97.19 (87.07 - 106.68) | 573.65 (504.04 - 639.38) | 1.25 (0.79 - 1.72) |
| Guam | 7.11 (5.84 - 8.59) | 72.44 (48.95 - 101.01) | 22.81 (20.24 - 27.18) | 63.76 (46.49 - 87.66) | -0.38 (-0.46 - -0.3) | 52.31 (41.69 - 65.72) | 562.31 (406.12 - 760.91) | 196.38 (166.4 - 248.18) | 535.96 (411.07 - 720.28) | -0.08 (-0.21 - 0.04) | 2.19 (1.78 - 2.57) | 30.53 (23.39 - 38.35) | 4 (3.16 - 4.89) | 11.47 (8.66 - 14.77) | -2.33 (-2.75 - -1.91) | 46.43 (38.31 - 53.35) | 529.12 (418.37 - 643.79) | 96.39 (80.23 - 113.28) | 270.61 (215.71 - 333.15) | -1.62 (-1.92 - -1.31) |
| Guatemala | 215.83 (185.98 - 252.39) | 50.92 (38.78 - 66.13) | 953.02 (822.15 - 1092.81) | 56.51 (41.31 - 74.56) | 0.24 (0.16 - 0.32) | 1494.72 (1220.27 - 1870.91) | 316.29 (232.97 - 427.55) | 7477.91 (5975.99 - 9264.5) | 434.51 (317.28 - 566.78) | 0.8 (0.66 - 0.95) | 107.85 (103.22 - 111.37) | 31.54 (29.41 - 33.37) | 334.22 (290.13 - 376.92) | 21.24 (18.28 - 24.21) | -1.42 (-1.57 - -1.28) | 1898.08 (1810.98 - 1989.57) | 472.19 (437.2 - 507.95) | 5979.74 (5264.08 - 6730.62) | 359.19 (309.26 - 411.8) | -1.07 (-1.18 - -0.96) |
| Guinea | 276.57 (237.73 - 309.28) | 57.68 (44.09 - 72.48) | 502.36 (440.96 - 562.37) | 65.44 (51.39 - 82.59) | 0.39 (0.36 - 0.43) | 1544.74 (1220.29 - 1838.48) | 320.45 (229.48 - 419.62) | 2966.41 (2427.51 - 3523.7) | 385.86 (288.21 - 499.34) | 0.56 (0.49 - 0.63) | 122.49 (96.66 - 153.36) | 29.41 (22.43 - 38.17) | 231.42 (183.87 - 293.59) | 34.57 (26.3 - 44.74) | 0.74 (0.66 - 0.83) | 2104.25 (1673.41 - 2594.7) | 459.85 (357.33 - 589.22) | 3911.96 (3099.54 - 4843.61) | 538.6 (414.98 - 686.59) | 0.7 (0.62 - 0.77) |
| Guinea-Bissau | 29.89 (25.61 - 33.95) | 60.26 (45.86 - 76.9) | 52.97 (45.54 - 60.92) | 65.4 (51.45 - 83.44) | 0.27 (0.23 - 0.31) | 167.5 (129.04 - 210.6) | 333.23 (239.11 - 444.78) | 312.34 (250.21 - 383.2) | 382.62 (284.07 - 508.42) | 0.42 (0.34 - 0.5) | 14.89 (11.32 - 18.08) | 37.72 (28.5 - 47.39) | 24.3 (18.88 - 29.11) | 40.32 (30.55 - 50.5) | 0.37 (0.32 - 0.43) | 281.08 (215.9 - 341.01) | 604.3 (457.73 - 754.36) | 454.53 (358.93 - 537.65) | 628.52 (485 - 774.28) | 0.26 (0.21 - 0.31) |
| Guyana | 24.54 (21.97 - 27.8) | 47.33 (37.27 - 60.8) | 47.6 (42.73 - 52.66) | 52.75 (42.22 - 64.19) | 0.26 (0.21 - 0.31) | 151.53 (125.41 - 187.56) | 271.52 (203.79 - 364.96) | 335.93 (284.68 - 402.6) | 345.1 (272.08 - 437.65) | 0.46 (0.33 - 0.58) | 13.16 (12.08 - 14.23) | 26.65 (24.04 - 29.17) | 23.6 (19.4 - 28.44) | 29.69 (23.88 - 35.97) | 0.51 (0.26 - 0.77) | 238.11 (218.6 - 258.82) | 448.88 (404.92 - 495.55) | 438.75 (364.88 - 527.48) | 494.55 (404.08 - 596) | 0.48 (0.28 - 0.67) |
| Haiti | 201.5 (178.65 - 226.73) | 50.58 (40.3 - 62.43) | 486.71 (424.38 - 549.46) | 54.72 (42.2 - 68.92) | 0.16 (0.11 - 0.2) | 1196.05 (978.52 - 1437.43) | 260.52 (194.91 - 340.75) | 3174.83 (2629.68 - 3837.19) | 319.98 (242.06 - 415.66) | 0.46 (0.35 - 0.56) | 140.76 (96.37 - 176.78) | 40.91 (28.18 - 52.68) | 285.55 (208.81 - 375.54) | 38.2 (26.65 - 52.01) | -0.07 (-0.12 - -0.02) | 2678.54 (1840.49 - 3362.68) | 664.47 (458.28 - 854.15) | 5314.62 (3913.87 - 6925.94) | 615.19 (439.93 - 824.82) | -0.12 (-0.17 - -0.08) |
| Honduras | 175.53 (155.62 - 198.86) | 62.61 (47.75 - 80.58) | 868.76 (760.74 - 999.54) | 96.72 (72.08 - 125.52) | 1.48 (1.36 - 1.6) | 1103.95 (900.84 - 1351.67) | 371.59 (275.6 - 493.48) | 5633.64 (4608.67 - 7029.96) | 605.09 (450.9 - 820.01) | 1.47 (1.32 - 1.62) | 99.18 (85.04 - 114.89) | 38.23 (31.07 - 46.75) | 449.82 (360.2 - 545.6) | 56.47 (43.24 - 70.1) | 1.55 (1.37 - 1.73) | 1709.38 (1483.46 - 1949.67) | 606.62 (502.61 - 727.47) | 7867.49 (6487.33 - 9393.73) | 894.72 (703.82 - 1093.48) | 1.54 (1.38 - 1.7) |
| Hungary | 1419.47 (1296.4 - 1551.87) | 54.1 (43.69 - 66.84) | 2115.35 (1943.41 - 2338.4) | 58.61 (45.77 - 72.49) | 0.35 (0.29 - 0.4) | 10940.84 (9466.25 - 12526.61) | 421.5 (341.78 - 512.53) | 17384.67 (15184.82 - 20065.97) | 474.39 (383.82 - 578.76) | 0.5 (0.42 - 0.58) | 623.71 (593.68 - 648.2) | 25.46 (23.72 - 26.88) | 950.43 (845.2 - 1040.94) | 24.91 (21.85 - 27.55) | 0.16 (0.04 - 0.28) | 11060.22 (10429.95 - 11689.4) | 431.89 (398.66 - 465.5) | 16137.57 (14470.71 - 17778.47) | 431.12 (379.89 - 481.58) | 0.17 (0.07 - 0.27) |
| Iceland | 44 (38.26 - 49.1) | 86.36 (64.25 - 112.79) | 115.2 (98.6 - 133.56) | 111.18 (82.92 - 144.19) | 0.83 (0.75 - 0.91) | 364.12 (304.99 - 433.17) | 707.78 (546.5 - 899.23) | 1019.91 (828.41 - 1230.34) | 960.67 (728.98 - 1218.57) | 1 (0.9 - 1.09) | 17.64 (15.81 - 18.76) | 33.3 (29.54 - 36.06) | 41.51 (34.23 - 46.16) | 36.65 (30.24 - 41.2) | 0.87 (0.64 - 1.1) | 301.3 (273.56 - 324.71) | 578.26 (513.73 - 637.33) | 682.86 (595.19 - 761.49) | 629.71 (534.08 - 712.79) | 0.71 (0.52 - 0.9) |
| India | 32222.78 (26332.6 - 38184.48) | 54.5 (37.03 - 73.67) | 114268.55 (94830.21 - 133007.69) | 62.72 (42.51 - 84.83) | 0.56 (0.49 - 0.64) | 212908.03 (172568.78 - 259441.7) | 343.43 (253.43 - 454.57) | 896355.4 (733172.48 - 1081812.71) | 495.83 (372.99 - 641.25) | 1.38 (1.29 - 1.47) | 11158.61 (8517.37 - 14845.71) | 23.08 (17 - 30.95) | 41885.36 (34625.12 - 49311.89) | 26.83 (21.49 - 32.31) | 0.64 (0.4 - 0.87) | 223075.22 (178317.55 - 286010.53) | 397.69 (306.59 - 515.59) | 794667 (669087.6 - 917989.46) | 463.56 (380.95 - 547.49) | 0.58 (0.41 - 0.74) |
| Indonesia | 5790.52 (4775.42 - 6797.4) | 45.72 (30.37 - 62.9) | 19497.62 (16153.15 - 22699.98) | 60.34 (41.11 - 81.47) | 0.98 (0.94 - 1.01) | 40852.33 (33349.47 - 49566.09) | 318.27 (235.09 - 421.7) | 139510.91 (114366.48 - 168801.26) | 430.08 (320.91 - 562.5) | 1.02 (0.98 - 1.06) | 2410.85 (1945.52 - 2976.92) | 23.31 (18.31 - 31.04) | 7843.24 (6176.55 - 9780.49) | 32.56 (25.11 - 42.03) | 1.09 (0.98 - 1.2) | 46620.88 (38967.35 - 55534.69) | 394.53 (320.33 - 502.99) | 150053.96 (121416.13 - 182425.18) | 527.58 (421.91 - 663.6) | 0.94 (0.84 - 1.03) |
| Iran (Islamic Republic of) | 1745.63 (1422.98 - 2065.4) | 54.04 (36.55 - 73.61) | 7938.19 (6628.84 - 9281.69) | 70.19 (47.81 - 95.58) | 0.8 (0.74 - 0.86) | 12046.58 (9989.63 - 14590.62) | 371.41 (274.48 - 487.28) | 63366.67 (52639.45 - 75305.3) | 563.03 (424.51 - 725.51) | 1.25 (1.17 - 1.32) | 619.89 (362.29 - 716.04) | 25.57 (15.73 - 29.81) | 2487.37 (1345.24 - 2779.75) | 23.81 (12.7 - 27.01) | -0.01 (-0.09 - 0.07) | 12602.17 (7899.33 - 14450.29) | 428.07 (270.83 - 493.05) | 45447.95 (27598.7 - 51535.46) | 415.37 (251.85 - 476.41) | 0.05 (-0.02 - 0.12) |
| Iraq | 596.18 (523.08 - 677.51) | 50.92 (36.63 - 67.97) | 2178.62 (1833.69 - 2543.3) | 70.34 (50.6 - 93.97) | 1.31 (1.22 - 1.4) | 3899.31 (3185.9 - 4744.2) | 331.98 (243.03 - 442.69) | 15870.55 (12747.29 - 19177.71) | 521.07 (382.8 - 684.33) | 1.76 (1.64 - 1.87) | 330.92 (269.59 - 405.89) | 29.01 (22.5 - 37.15) | 870.6 (661.46 - 1053.24) | 35.01 (25.85 - 43.83) | 0.19 (-0.02 - 0.41) | 5577.2 (4662.41 - 6769.37) | 479.73 (382.18 - 602.1) | 15923.94 (12582.42 - 18700.26) | 563.93 (427.34 - 695.63) | 0.23 (0.06 - 0.4) |
| Ireland | 524.77 (464.36 - 619.04) | 70.77 (54 - 91.56) | 1368.94 (1151.5 - 1558.34) | 96.36 (70.29 - 125.29) | 1.05 (0.97 - 1.12) | 4135.51 (3579.91 - 4864.78) | 571.57 (453.46 - 727.65) | 12193.84 (9907.69 - 14042.66) | 851.24 (654.48 - 1056.43) | 1.33 (1.24 - 1.43) | 195.14 (183.52 - 202.35) | 27.89 (25.46 - 29.7) | 385.82 (330.01 - 419.54) | 26.12 (21.93 - 28.82) | 0.24 (0.1 - 0.39) | 3473.4 (3239.83 - 3699.41) | 476.45 (431.82 - 520.9) | 6926.36 (6187.41 - 7590.06) | 475.87 (408.52 - 539.69) | 0.33 (0.22 - 0.43) |
| Israel | 870.37 (759.25 - 1050.51) | 100.46 (74.58 - 132.66) | 2651.63 (2245.71 - 3167.31) | 120.25 (86.04 - 159.55) | 0.29 (0.14 - 0.44) | 7704.03 (6477.5 - 9403.18) | 913.27 (724.48 - 1169.36) | 25188.73 (20645.22 - 30583.56) | 1115.46 (853.68 - 1427.6) | 0.26 (0.05 - 0.47) | 236.61 (216.12 - 248.54) | 29.5 (26.32 - 31.68) | 633.25 (537.46 - 689.1) | 26.8 (22.55 - 29.46) | -0.18 (-0.27 - -0.08) | 4523.56 (4120.24 - 4921.84) | 535.56 (474.77 - 597.97) | 11885.17 (10510.97 - 13332.49) | 519.31 (440.97 - 595.28) | -0.12 (-0.19 - -0.04) |
| Italy | 15705.96 (13110.08 - 18318.66) | 96.41 (69.75 - 126.83) | 21248.8 (17581.92 - 24605.47) | 76.17 (51.53 - 103.43) | -1.21 (-1.45 - -0.98) | 145110.72 (121884.28 - 171978.41) | 895.22 (707.42 - 1108.41) | 179482.06 (145939 - 213716.29) | 614.02 (460.58 - 785.27) | -1.84 (-2.16 - -1.52) | 4421.38 (3977.12 - 4633.3) | 27.77 (24.78 - 29.38) | 8942.72 (7461.21 - 9729.09) | 26.75 (22.54 - 29.06) | 0.02 (-0.17 - 0.21) | 83610.07 (75794.16 - 91420.36) | 514.45 (458.03 - 571.2) | 138784.79 (120481.75 - 151941.36) | 452.53 (392.18 - 503.23) | -0.45 (-0.57 - -0.33) |
| Jamaica | 132.67 (118.56 - 144.98) | 41.71 (31.89 - 53.32) | 262.47 (240.77 - 295.96) | 48.38 (37.43 - 62.8) | 0.46 (0.4 - 0.53) | 913.51 (748.99 - 1091.68) | 292.09 (220.39 - 374.78) | 1963.23 (1677.03 - 2411.78) | 369.7 (285.82 - 486.16) | 0.67 (0.56 - 0.78) | 70.28 (65 - 73.63) | 21.66 (19.67 - 23.24) | 145.06 (116.6 - 176.05) | 26.57 (21.08 - 32.48) | 0.72 (0.48 - 0.96) | 1143.18 (1068.69 - 1205.38) | 353.97 (320.68 - 385.17) | 2303.14 (1903.88 - 2744.32) | 437.09 (353.48 - 528.78) | 0.73 (0.49 - 0.96) |
| Japan | 12298.53 (10217.3 - 14456.74) | 42.71 (28.42 - 58.82) | 28317.19 (24093.2 - 32820.69) | 38.61 (26.23 - 52.34) | 0.19 (0.05 - 0.33) | 102163.22 (84667.15 - 123573.31) | 354.32 (269.46 - 458.91) | 194212.8 (158825.68 - 233447.61) | 278.85 (210.1 - 362.35) | 0.03 (-0.23 - 0.29) | 4951.84 (4458.09 - 5182.45) | 18.48 (16.46 - 19.54) | 18480.03 (14917.28 - 20352.92) | 20.58 (17.02 - 22.52) | 0.62 (0.52 - 0.71) | 88170.48 (80822.46 - 94179.54) | 313.86 (282.14 - 341.83) | 262462.78 (221133.58 - 285965.91) | 331 (284.06 - 364.24) | 0.51 (0.4 - 0.62) |
| Jordan | 89.76 (79 - 100.73) | 58.54 (44.46 - 74.5) | 577.34 (518.43 - 644.24) | 62.14 (48.19 - 78.1) | 0.35 (0.29 - 0.41) | 538.49 (441.53 - 631.21) | 335.72 (245.29 - 433.81) | 3872.38 (3336.77 - 4443.82) | 412.89 (325.29 - 515.04) | 1.05 (0.91 - 1.19) | 41.44 (33.81 - 50.44) | 31.04 (24.71 - 38.7) | 172.9 (139.19 - 208.49) | 23.31 (18.08 - 29.01) | -1.05 (-1.19 - -0.91) | 733.5 (614.46 - 877.47) | 489.27 (396.3 - 604.75) | 3224.98 (2677.66 - 3855.37) | 374.9 (299.19 - 458.07) | -0.97 (-1.1 - -0.84) |
| Kazakhstan | 1051.61 (898.82 - 1200.66) | 54.85 (38.22 - 74.59) | 1870.43 (1656.97 - 2118.59) | 69.08 (52.59 - 88.39) | 0.91 (0.84 - 0.99) | 7873.49 (6211.24 - 9653.1) | 417.2 (303.56 - 551.4) | 13594.51 (11396.06 - 15822.42) | 516.4 (403.27 - 643.68) | 0.86 (0.71 - 1.01) | 381.43 (356.24 - 407.19) | 21.89 (20.01 - 23.55) | 674.3 (601.13 - 749.32) | 30.73 (27.22 - 34.08) | 0.9 (0.68 - 1.11) | 7087.89 (6578.85 - 7679.03) | 387.36 (349.96 - 428.54) | 12354.27 (11120.15 - 13622.91) | 508.76 (450.38 - 566.4) | 0.7 (0.53 - 0.86) |
| Kenya | 478.64 (391.1 - 566.15) | 42.4 (28.74 - 57.67) | 1466.2 (1207.86 - 1695.53) | 49.16 (34.36 - 65.8) | 0.51 (0.46 - 0.57) | 3165.8 (2572.27 - 3853.04) | 278.68 (204.17 - 367.67) | 9803.49 (7985.62 - 11792.2) | 329.69 (244.71 - 427.14) | 0.51 (0.44 - 0.57) | 183.64 (141.24 - 243.08) | 18.94 (14.2 - 26.06) | 583.96 (445.46 - 747.07) | 25.11 (18.38 - 33.5) | 1.1 (1.03 - 1.17) | 3391.29 (2718.72 - 4324.79) | 315.46 (249.06 - 413.72) | 11071.71 (8750.4 - 13736.08) | 410.41 (315.14 - 526.97) | 1.03 (0.96 - 1.11) |
| Kiribati | 3.4 (2.9 - 3.92) | 70.38 (52.62 - 90.58) | 6.69 (5.88 - 7.71) | 73.5 (56.53 - 95.24) | 0.2 (0.17 - 0.23) | 20.58 (16.3 - 25.48) | 426.13 (309.44 - 561.58) | 42.73 (35.27 - 52.48) | 464.59 (347.44 - 623.23) | 0.35 (0.28 - 0.41) | 1.26 (1.04 - 1.46) | 32.23 (24.85 - 41.03) | 2.27 (1.9 - 2.78) | 32.95 (25.43 - 42.19) | 0.04 (0.01 - 0.06) | 25.96 (22.13 - 29.56) | 568.44 (453.32 - 699.31) | 46.64 (39.32 - 56.47) | 565.26 (447.45 - 709.02) | -0.04 (-0.07 - -0.02) |
| Kuwait | 35.52 (30.72 - 41.63) | 51.56 (38.27 - 67.2) | 206.02 (169.54 - 241.83) | 60.65 (42.81 - 82.38) | 0.52 (0.45 - 0.6) | 271.41 (226.22 - 324.06) | 393.15 (306.04 - 502.85) | 1813.97 (1462.61 - 2210.07) | 526.88 (383.22 - 684.25) | 0.95 (0.86 - 1.05) | 20.8 (18.33 - 22.44) | 36.44 (31.28 - 40.15) | 68.25 (55.66 - 82.21) | 22.16 (17.66 - 26.74) | -0.74 (-1.33 - -0.14) | 375.78 (338.16 - 408.73) | 590.77 (516.77 - 652.19) | 1185.78 (1001.65 - 1385.85) | 370.35 (302.76 - 440.45) | -0.75 (-1.27 - -0.22) |
| Kyrgyzstan | 226.98 (198.95 - 255.63) | 48.45 (34.85 - 64.25) | 332.98 (289.7 - 377.14) | 48.34 (35.69 - 61.94) | 0 (-0.09 - 0.08) | 1789.46 (1470.45 - 2134.13) | 386.45 (295.04 - 501.2) | 2532.55 (2090.01 - 3036.02) | 382.01 (295.82 - 489.24) | -0.02 (-0.17 - 0.13) | 90.03 (78.61 - 105.27) | 21.02 (17.87 - 24.77) | 99.05 (85.06 - 113.45) | 17.64 (14.86 - 20.41) | -0.54 (-0.78 - -0.3) | 1613.26 (1413.39 - 1829.22) | 361.56 (308.62 - 421.47) | 1943.12 (1696.38 - 2217.96) | 317.59 (269.34 - 368.99) | -0.43 (-0.65 - -0.21) |
| Lao People's Democratic Republic | 139.06 (119.49 - 158.18) | 49.2 (37.8 - 62.51) | 353.83 (308.4 - 401.92) | 56.13 (42.5 - 71.87) | 0.44 (0.4 - 0.48) | 915.92 (738.16 - 1123.05) | 316.05 (235.48 - 412.54) | 2527.16 (2104.29 - 3112.11) | 399.89 (298.09 - 514.94) | 0.75 (0.69 - 0.82) | 64.64 (50.72 - 81.46) | 29.28 (21.76 - 38.52) | 153.82 (122.75 - 194.95) | 30.09 (23.19 - 38.59) | -0.02 (-0.07 - 0.04) | 1298.33 (1032.94 - 1624.4) | 495.01 (376.94 - 640.97) | 2869.12 (2297.81 - 3548.98) | 497.83 (389.92 - 627.7) | -0.08 (-0.13 - -0.04) |
| Latvia | 372.98 (337.91 - 411.02) | 59.95 (44.87 - 75.25) | 433.4 (392.03 - 478.56) | 59.35 (44.19 - 75.97) | -0.21 (-0.32 - -0.11) | 3220.57 (2774.77 - 3730.26) | 517.5 (416.63 - 637.04) | 3896.02 (3353.05 - 4728.78) | 515.1 (408.35 - 651.61) | -0.26 (-0.39 - -0.14) | 163.23 (148.14 - 178.2) | 26.76 (23.78 - 29.75) | 217.91 (192.1 - 240.02) | 25.63 (22.24 - 28.53) | -0.2 (-0.36 - -0.04) | 2835.85 (2566.8 - 3111.56) | 457.19 (405.5 - 512.85) | 3521.59 (3138.28 - 3871.79) | 438.27 (381.91 - 492.98) | -0.25 (-0.4 - -0.11) |
| Lebanon | 159.14 (138.16 - 181.19) | 51.77 (38.75 - 66.94) | 660.88 (575.33 - 751.06) | 62.12 (43.81 - 85.08) | 0.64 (0.6 - 0.67) | 1101.16 (899.03 - 1322.49) | 356.28 (263.7 - 460.52) | 5438.57 (4490.55 - 6588.34) | 504.73 (377.18 - 672.09) | 1.2 (1.13 - 1.26) | 82.25 (52.12 - 106.79) | 30.89 (19.27 - 41.41) | 268.92 (228.26 - 320.43) | 23.18 (18.72 - 29.04) | -0.54 (-0.72 - -0.35) | 1434.26 (952.88 - 1843.6) | 492.4 (317.78 - 650.96) | 4236.48 (3638.62 - 4946.09) | 379.42 (311.49 - 466.16) | -0.5 (-0.66 - -0.34) |
| Lesotho | 47.08 (40.22 - 54.04) | 37.85 (27.88 - 49.82) | 71.42 (61.53 - 82.33) | 49.65 (37.7 - 63.12) | 0.89 (0.84 - 0.94) | 310.47 (246.58 - 380.37) | 250.7 (180.86 - 331.21) | 457.88 (376.44 - 568.34) | 319.22 (235.44 - 419.8) | 0.65 (0.56 - 0.74) | 22.3 (17.96 - 28.12) | 20.68 (15.42 - 28.51) | 33.6 (25.22 - 42.98) | 28.9 (20.66 - 38.58) | 1.75 (1.44 - 2.06) | 399.14 (330.98 - 487.41) | 336.5 (261.34 - 447.52) | 645.46 (493.98 - 802.87) | 477.8 (349.03 - 622.5) | 1.65 (1.39 - 1.91) |
| Liberia | 90 (78.84 - 102.95) | 54.2 (41.48 - 71.04) | 145.96 (126.1 - 164.3) | 57.97 (43.89 - 74.22) | 0.19 (0.15 - 0.23) | 533.8 (430.68 - 692.46) | 321.92 (238.9 - 444.65) | 953.6 (761.94 - 1117.97) | 377.79 (278.29 - 486.24) | 0.52 (0.46 - 0.58) | 40.62 (34.8 - 48.42) | 30.18 (24.53 - 37.12) | 62.81 (45.15 - 83.94) | 29.93 (21.1 - 40.19) | 0.09 (0 - 0.17) | 717.4 (614.27 - 844.83) | 466.42 (383.98 - 565.63) | 1084.83 (797.23 - 1390.45) | 467.2 (339.11 - 617.54) | 0.1 (0.02 - 0.18) |
| Libya | 130.72 (115.23 - 147.57) | 48.12 (36.15 - 62.03) | 430.71 (378.27 - 489.82) | 61.07 (43.85 - 79.69) | 0.87 (0.81 - 0.94) | 913.03 (750.48 - 1065.54) | 335.26 (250.59 - 426.52) | 3442.06 (2846.85 - 4059.55) | 491.15 (367.84 - 621.08) | 1.38 (1.27 - 1.48) | 68.46 (49.13 - 91.63) | 27.42 (18.94 - 38.99) | 228.72 (145.99 - 379.01) | 36.48 (22.24 - 61.87) | 1.54 (1.34 - 1.75) | 1174.55 (897.78 - 1500.89) | 450.4 (327.16 - 613.02) | 4044.08 (2736.25 - 6361.88) | 605.26 (393.31 - 973.31) | 1.42 (1.26 - 1.57) |
| Lithuania | 394.57 (353.04 - 457.42) | 51.05 (36.57 - 67.97) | 592 (521.5 - 646.68) | 56.69 (41.96 - 72.46) | 0.13 (0.05 - 0.21) | 3730.06 (3112.56 - 4508.85) | 482.13 (382.86 - 628.1) | 5480.02 (4538.4 - 6374.22) | 506.29 (387.5 - 621.13) | -0.11 (-0.22 - 0) | 158.98 (146.69 - 172.11) | 20.77 (18.71 - 22.76) | 305.23 (270.3 - 338.24) | 24.72 (21.42 - 27.58) | 0.76 (0.58 - 0.94) | 2840.07 (2591.86 - 3110.87) | 368.34 (328.5 - 413.57) | 4870.2 (4343.5 - 5370.26) | 420.1 (365.95 - 473.65) | 0.49 (0.36 - 0.63) |
| Luxembourg | 78.55 (68.18 - 90.93) | 80.2 (59.79 - 103.28) | 187.71 (159.38 - 214.23) | 102.19 (72.35 - 132.81) | 0.82 (0.78 - 0.86) | 623.19 (513.12 - 764.07) | 637.49 (489.51 - 817.81) | 1664.1 (1359.48 - 1994.75) | 875.34 (654.6 - 1107.38) | 1.1 (1.04 - 1.15) | 31.12 (29.46 - 32.55) | 32.1 (29.73 - 34.15) | 64.01 (56.25 - 70.12) | 31.1 (26.93 - 34.38) | 0.42 (0.25 - 0.6) | 540.17 (503.94 - 577.94) | 547.9 (499.8 - 598.79) | 1062.03 (944.44 - 1179.18) | 543.14 (472.06 - 614.17) | 0.38 (0.25 - 0.52) |
| Madagascar | 248.18 (213.65 - 292.83) | 36.92 (27.05 - 49.42) | 515.52 (424.73 - 602.94) | 40 (28.5 - 53.21) | 0.28 (0.27 - 0.3) | 1611 (1286.86 - 2018.93) | 234.1 (166.76 - 318.45) | 3646.29 (2888.65 - 4395.15) | 278.97 (202.47 - 369.2) | 0.55 (0.53 - 0.57) | 115.42 (91.68 - 147.65) | 20.09 (15.08 - 27.44) | 183.04 (130.47 - 254.44) | 19.25 (12.85 - 28.44) | -0.12 (-0.19 - -0.05) | 2188.22 (1813.07 - 2652.91) | 337.21 (263.67 - 440.16) | 3752.04 (2799.29 - 4995.53) | 324.92 (229.32 - 455.28) | -0.11 (-0.18 - -0.04) |
| Malawi | 210.19 (179.67 - 242.33) | 42.66 (32.04 - 55.22) | 444.96 (374.93 - 510.52) | 46.04 (33.65 - 60.67) | 0.24 (0.22 - 0.27) | 1308.08 (1035.37 - 1611.33) | 260.26 (188.27 - 344.37) | 2929.12 (2378.66 - 3546.74) | 303.85 (219.17 - 400.64) | 0.45 (0.4 - 0.5) | 85.05 (64.09 - 105.6) | 21.82 (15.32 - 29.86) | 193.49 (127.54 - 267.54) | 24.96 (15.63 - 36.65) | 0.34 (0.23 - 0.45) | 1684.9 (1324.53 - 2009.16) | 364.25 (270.27 - 476.27) | 3696.7 (2579.73 - 4912.64) | 413.96 (276.04 - 580.98) | 0.29 (0.18 - 0.41) |
| Malaysia | 704.4 (620.45 - 796.5) | 53.06 (38.74 - 71.11) | 2802.75 (2393.75 - 3282.38) | 64.55 (45.89 - 88.14) | 0.72 (0.67 - 0.77) | 4980.42 (4012.84 - 5996.81) | 376.13 (276.68 - 499.29) | 21585.62 (17572.08 - 27117.31) | 502.85 (377.76 - 671.44) | 1.04 (0.99 - 1.08) | 275.46 (239.27 - 322.1) | 22.2 (17.98 - 27.3) | 1056.28 (941.53 - 1189.4) | 28.66 (23.79 - 34.07) | 0.8 (0.53 - 1.08) | 5106.27 (4536.7 - 5741.43) | 396.1 (331 - 471.21) | 19618.47 (17629.84 - 21894.78) | 484.39 (409.45 - 567.62) | 0.6 (0.41 - 0.79) |
| Maldives | 6.48 (5.63 - 7.53) | 63.18 (47.84 - 80.95) | 30.71 (26.84 - 35.19) | 69.38 (51.61 - 88.94) | 0.32 (0.3 - 0.35) | 44.01 (35.83 - 52.94) | 409.81 (302.99 - 533.89) | 228.95 (189.63 - 279.89) | 515 (392.85 - 664.98) | 0.74 (0.66 - 0.81) | 2.67 (2.13 - 3.06) | 34.42 (25.68 - 42.32) | 10 (8.05 - 11.87) | 24.06 (18.58 - 29.72) | -1.35 (-1.44 - -1.26) | 53 (43.19 - 61.26) | 562.31 (429.28 - 683.32) | 168.07 (141.67 - 194.45) | 393.41 (312.79 - 480.47) | -1.39 (-1.48 - -1.31) |
| Mali | 243.83 (211.93 - 276.07) | 48.68 (37.4 - 61.12) | 602.3 (528.01 - 689.66) | 53.2 (42.05 - 67.93) | 0.27 (0.25 - 0.3) | 1494.86 (1228.93 - 1779.29) | 295.35 (220.22 - 382.55) | 3851.12 (3228.96 - 4690.8) | 339.58 (261.22 - 438.99) | 0.43 (0.39 - 0.47) | 124.33 (104.58 - 147.23) | 33.22 (26.16 - 41.82) | 308.54 (250.33 - 368.29) | 35.07 (27.45 - 44.2) | 0.39 (0.29 - 0.5) | 2258.21 (1898.96 - 2644.76) | 508.89 (407.67 - 627.59) | 5426.11 (4438.32 - 6475.83) | 535.82 (422.88 - 662.24) | 0.36 (0.26 - 0.46) |
| Malta | 54.66 (46.49 - 63.28) | 74.9 (56.49 - 96.28) | 179.78 (160.47 - 207.34) | 96.47 (72.47 - 123.44) | 0.85 (0.79 - 0.91) | 420.19 (345.75 - 513.61) | 587.62 (447.39 - 759.59) | 1560.74 (1323.69 - 1855.71) | 824.76 (654.65 - 1043.91) | 1.18 (1.07 - 1.28) | 20.96 (19.42 - 22.08) | 30.65 (27.63 - 32.98) | 55.72 (47.19 - 61.64) | 28.24 (23.7 - 31.59) | -0.14 (-0.41 - 0.14) | 374.71 (344.36 - 402.03) | 526.4 (475.25 - 579.11) | 977.26 (863.55 - 1087.5) | 506.69 (433.13 - 575.72) | -0.02 (-0.25 - 0.21) |
| Marshall Islands | 1.6 (1.34 - 1.87) | 71.21 (52.54 - 95.64) | 3.19 (2.68 - 3.82) | 76.58 (57.35 - 100.28) | 0.32 (0.28 - 0.37) | 10.46 (8.27 - 13.11) | 474.94 (344.79 - 637.48) | 21.95 (17.88 - 26.87) | 535.71 (398.88 - 717.64) | 0.5 (0.42 - 0.58) | 0.74 (0.64 - 0.84) | 38.87 (31.31 - 47.24) | 1.13 (0.91 - 1.4) | 37.72 (29.05 - 48.38) | -0.08 (-0.14 - -0.02) | 14.95 (12.9 - 17.01) | 686.1 (560.88 - 817.1) | 24.92 (19.94 - 30.72) | 656.69 (516.4 - 827.46) | -0.1 (-0.15 - -0.04) |
| Mauritania | 83 (72.17 - 93.91) | 57.82 (44.92 - 72.72) | 191.02 (166.34 - 223.72) | 63 (47.97 - 83.11) | 0.26 (0.21 - 0.3) | 472.77 (382.48 - 573.15) | 328.74 (243.54 - 429.08) | 1214.71 (980.19 - 1518.74) | 402.7 (303.85 - 541.98) | 0.6 (0.53 - 0.67) | 35.25 (27.33 - 43.49) | 29.22 (21.74 - 37.61) | 77.54 (55.71 - 104.31) | 29.64 (21.05 - 40.47) | 0.05 (-0.1 - 0.21) | 621.36 (491.65 - 755.94) | 457.4 (348.9 - 576.84) | 1309.91 (976.43 - 1715.98) | 460.47 (336.71 - 614.26) | 0.02 (-0.13 - 0.17) |
| Mauritius | 53.68 (48.09 - 60.56) | 50.92 (38.78 - 65.8) | 182.16 (155.39 - 204.24) | 59.19 (43.93 - 75.34) | 0.35 (0.3 - 0.41) | 401.29 (337.92 - 490.14) | 379.4 (291.55 - 489.17) | 1456.09 (1191.2 - 1734.36) | 480.38 (353.62 - 599.64) | 0.57 (0.49 - 0.64) | 22.99 (21.76 - 24.05) | 26.44 (24.21 - 28.26) | 66.87 (60.71 - 70.85) | 24.69 (21.87 - 26.83) | -0.24 (-0.36 - -0.13) | 435.51 (409.95 - 459.73) | 444.36 (405.46 - 480.37) | 1233.08 (1133.86 - 1319.48) | 427.27 (379.97 - 472.13) | -0.21 (-0.31 - -0.11) |
| Mexico | 2935.13 (2441.98 - 3438.19) | 48.97 (32.52 - 67.72) | 12643.77 (10524.46 - 14717.6) | 63.7 (42.5 - 87.94) | 0.62 (0.51 - 0.74) | 21404.85 (17866.84 - 25925.8) | 339.38 (251.46 - 447.8) | 104359.82 (86885.84 - 124204.23) | 521.68 (391.93 - 674.5) | 1.01 (0.84 - 1.17) | 1596.25 (1519.37 - 1637.23) | 30.06 (28.36 - 31.09) | 4742.47 (4235.17 - 5249.11) | 25.44 (22.43 - 28.19) | -0.43 (-0.52 - -0.33) | 26269.36 (25028.44 - 27507.18) | 452.54 (424.14 - 478.93) | 84411.35 (75478.24 - 94270.82) | 434.05 (382.78 - 489.04) | -0.12 (-0.2 - -0.04) |
| Micronesia (Federated States of) | 4.86 (4.21 - 5.54) | 68.06 (50.84 - 89.15) | 6.84 (5.83 - 8.06) | 72.3 (54.24 - 94.52) | 0.32 (0.28 - 0.36) | 30.78 (25.08 - 37.12) | 434.8 (318.95 - 575.35) | 46.79 (37.66 - 56.99) | 490.31 (358.67 - 641.4) | 0.59 (0.53 - 0.65) | 2.38 (1.98 - 2.8) | 38.17 (30.25 - 47.05) | 2.49 (1.98 - 3.14) | 34.68 (26.16 - 45.18) | -0.34 (-0.38 - -0.3) | 45.73 (38.01 - 53.29) | 657.31 (528.58 - 804.06) | 51.35 (41.59 - 63.83) | 597.91 (465.35 - 764.2) | -0.32 (-0.36 - -0.27) |
| Monaco | 10.37 (8.9 - 12.3) | 76.82 (53.1 - 105.02) | 18.32 (15.22 - 22.15) | 99.98 (69.25 - 139.28) | 0.88 (0.84 - 0.92) | 87.59 (68.87 - 106.09) | 633.39 (468.51 - 836.9) | 166.62 (134 - 209.58) | 871.93 (646.28 - 1155.69) | 1.06 (1.01 - 1.11) | 4.6 (3.51 - 5.68) | 30.31 (22.53 - 38.45) | 7.7 (5.87 - 9.62) | 36.55 (26.88 - 46.75) | 0.8 (0.7 - 0.89) | 75.08 (59.64 - 91.14) | 518.14 (400.47 - 644.75) | 124.4 (100.07 - 147.61) | 623.38 (477.84 - 770.32) | 0.75 (0.67 - 0.84) |
| Mongolia | 84.32 (75.45 - 95.78) | 52.63 (38.84 - 69.63) | 150.66 (131.84 - 169.09) | 49.2 (35.88 - 64.48) | -0.12 (-0.17 - -0.07) | 589.79 (481 - 751.45) | 369.79 (279.03 - 498.33) | 1103 (900.55 - 1332.89) | 370.56 (278.82 - 486.8) | 0.16 (0.08 - 0.24) | 31.97 (27.22 - 38.4) | 22.85 (18.77 - 29.3) | 50.24 (42.36 - 59.44) | 20.9 (16.71 - 25.88) | -0.58 (-0.71 - -0.45) | 598.5 (522.58 - 696.53) | 394.77 (329.83 - 488.41) | 953.96 (807.88 - 1099.18) | 355.59 (291.9 - 430.92) | -0.57 (-0.68 - -0.46) |
| Montenegro | 69.67 (60.59 - 80.03) | 69.22 (48.97 - 93.57) | 127.85 (111.43 - 143.16) | 73.62 (53.19 - 95.97) | 0.32 (0.27 - 0.36) | 547.17 (445.48 - 646.44) | 550.75 (408.05 - 729.51) | 966 (790.96 - 1120.86) | 566.77 (428.8 - 719.42) | 0.23 (0.14 - 0.31) | 27.13 (23.11 - 32.24) | 28.38 (23.63 - 34.69) | 59.19 (50.16 - 69.81) | 39.88 (32.9 - 48.78) | 1.18 (0.77 - 1.6) | 475.52 (411.57 - 550.45) | 484.91 (406.88 - 581.4) | 986.69 (859.24 - 1150.95) | 619.03 (519.18 - 749.44) | 0.84 (0.58 - 1.11) |
| Morocco | 1035.95 (903.21 - 1191.32) | 48.64 (36.51 - 63.55) | 3611.65 (3122.44 - 4101.51) | 69.04 (51.07 - 90.37) | 1.34 (1.27 - 1.41) | 6815.21 (5474.14 - 8342.79) | 319.68 (234.68 - 425.88) | 26717.9 (21572.77 - 32183.59) | 514 (383.88 - 667.65) | 1.77 (1.68 - 1.87) | 496.86 (404.54 - 606.09) | 25.66 (20.11 - 33.15) | 1546.58 (1198.96 - 1828.28) | 33.65 (25.2 - 41.62) | 1.1 (1 - 1.2) | 8883.03 (7418.25 - 10533.94) | 428.52 (343.93 - 536.39) | 28370.59 (22362.14 - 33368.05) | 568.7 (436.24 - 696.85) | 1.13 (1.03 - 1.22) |
| Mozambique | 317.6 (271.07 - 373.23) | 42.01 (31.4 - 55.23) | 648.4 (548.51 - 760.57) | 47.16 (35.23 - 60.73) | 0.35 (0.33 - 0.37) | 1920.4 (1538.42 - 2432.61) | 247.39 (178.89 - 335.95) | 4129.24 (3268.98 - 5023.35) | 296.39 (214.04 - 392.28) | 0.47 (0.42 - 0.51) | 140.69 (108.22 - 186.96) | 22.88 (16.34 - 32.6) | 300.32 (206.4 - 426.99) | 27.69 (18.05 - 41.83) | 1.02 (0.88 - 1.15) | 2666.34 (2112.97 - 3451.54) | 373.34 (278.57 - 509.64) | 5725.01 (4161.73 - 7796.72) | 451.97 (311.65 - 648.12) | 0.97 (0.85 - 1.1) |
| Myanmar | 1398.83 (1215.59 - 1565.38) | 44.12 (33.86 - 57.06) | 3958.57 (3483.71 - 4402) | 55.04 (41.28 - 70.97) | 0.8 (0.76 - 0.83) | 9476.74 (7659.43 - 11353.22) | 292.99 (219.61 - 381.5) | 29232.54 (23989.65 - 34253.15) | 406.37 (311.39 - 521.23) | 1.12 (1.05 - 1.19) | 634.98 (528.62 - 772.22) | 24.66 (19.12 - 32.17) | 1708.3 (1383.48 - 2216.91) | 28.03 (21.59 - 37.38) | 0.28 (0.18 - 0.38) | 12481.67 (10378.06 - 14970.16) | 418.68 (329.41 - 534.06) | 31433.07 (25729 - 39309.1) | 469.72 (368.52 - 608.51) | 0.24 (0.15 - 0.34) |
| Namibia | 38.14 (31.71 - 45.26) | 46.05 (34.35 - 59.48) | 98.98 (85.31 - 113.62) | 54.26 (41.26 - 69.38) | 0.56 (0.51 - 0.61) | 245.48 (196.36 - 308.18) | 291.48 (213.38 - 386.33) | 666.72 (539.12 - 800.18) | 368.62 (275.64 - 472.24) | 0.73 (0.62 - 0.83) | 15.64 (12.83 - 19.26) | 24.88 (19.42 - 32.65) | 44.31 (35.8 - 53.64) | 29.61 (22.79 - 37.54) | 0.62 (0.47 - 0.76) | 306.96 (257.14 - 366.07) | 403.83 (320.97 - 514.3) | 813.37 (662.34 - 971.66) | 479.08 (376.46 - 596.8) | 0.57 (0.45 - 0.7) |
| Nauru | 0.45 (0.38 - 0.54) | 84.17 (63.15 - 112.95) | 0.58 (0.5 - 0.66) | 80.26 (60.27 - 103.36) | -0.08 (-0.16 - -0.01) | 2.76 (2.16 - 3.59) | 522.47 (379.43 - 733.34) | 3.94 (3.22 - 4.77) | 541.59 (402.66 - 703.11) | 0.23 (0.08 - 0.38) | 0.22 (0.16 - 0.28) | 51.57 (37.03 - 67.99) | 0.25 (0.18 - 0.33) | 42.93 (29.78 - 56.53) | -0.59 (-0.61 - -0.57) | 4.54 (3.41 - 5.81) | 884.8 (650.07 - 1153.35) | 5.18 (3.89 - 6.63) | 744.59 (537.47 - 968.13) | -0.55 (-0.58 - -0.53) |
| Nepal | 497.21 (419.64 - 587.22) | 40.79 (29.97 - 53.99) | 2043.83 (1741.47 - 2395.45) | 57.53 (42.94 - 77.2) | 1.28 (1.24 - 1.33) | 3468.07 (2749.6 - 4243.37) | 277.63 (202.04 - 374.24) | 15747.44 (12814.05 - 19346.91) | 446.25 (334.79 - 585.38) | 1.68 (1.63 - 1.73) | 227.68 (181.03 - 281.54) | 23.71 (17.7 - 31.35) | 855.46 (682.25 - 1066.95) | 29.58 (22.58 - 38.24) | 0.91 (0.68 - 1.14) | 4321.27 (3547.89 - 5257.25) | 389.64 (298.95 - 501.77) | 15689.68 (12867.78 - 19101.48) | 484.29 (379.29 - 610.85) | 0.89 (0.68 - 1.1) |
| Netherlands | 3764 (3316.99 - 4141.34) | 104.19 (80.64 - 131.19) | 6853.46 (6129.94 - 7621.23) | 105.21 (81.55 - 128.67) | -0.31 (-0.56 - -0.06) | 32769.58 (28669.19 - 36675.74) | 909.11 (747.15 - 1087.55) | 62445.82 (54784.05 - 71013.76) | 940.2 (773.82 - 1112) | -0.35 (-0.66 - -0.05) | 1184.04 (1077.01 - 1246.96) | 32.54 (28.94 - 34.91) | 2108.55 (1825.42 - 2282.8) | 30.36 (25.92 - 33.17) | 0.11 (-0.02 - 0.25) | 21233.56 (19339.94 - 22952.77) | 582.21 (517.67 - 641.42) | 36683.05 (32754 - 40102.27) | 541.51 (469.47 - 607.19) | -0.08 (-0.17 - 0.01) |
| New Zealand | 350.19 (287.26 - 414.86) | 50.32 (34.39 - 68.21) | 877.25 (736.93 - 1017.58) | 57.07 (39.16 - 76.22) | 0.47 (0.45 - 0.5) | 2307.64 (1856.35 - 2847.86) | 332.02 (244.74 - 435.32) | 5958.86 (4849.05 - 7220.78) | 385.63 (285.25 - 503.14) | 0.51 (0.47 - 0.55) | 162.22 (148.98 - 170.04) | 24.1 (21.72 - 25.83) | 452.49 (392.5 - 488.73) | 28.37 (24.28 - 31.09) | 0.67 (0.59 - 0.76) | 2746.23 (2548.41 - 2917.21) | 396.7 (359.84 - 431.5) | 6972.03 (6207.81 - 7498.16) | 444.63 (388.19 - 489.99) | 0.48 (0.42 - 0.55) |
| Nicaragua | 125.46 (110.12 - 139.02) | 57.01 (43.03 - 72.77) | 584.34 (514.47 - 652.63) | 78.82 (59.11 - 101.06) | 0.94 (0.85 - 1.03) | 887.3 (743.44 - 1033.23) | 393.9 (298.92 - 498.3) | 4413.2 (3575.67 - 5200.64) | 589.8 (438.27 - 745.54) | 1.1 (0.99 - 1.22) | 40.31 (35.51 - 47.5) | 20.21 (16.88 - 24.64) | 131.05 (108.14 - 154.78) | 18.94 (14.72 - 23.38) | 0.08 (-0.19 - 0.35) | 730.88 (654.38 - 828.76) | 343.05 (290.65 - 409.35) | 2604.63 (2196.18 - 3008.62) | 357.97 (287.98 - 433.54) | 0.3 (0.07 - 0.52) |
| Niger | 164.77 (138.38 - 189.91) | 50.16 (37.3 - 64.79) | 556.8 (474.78 - 658.17) | 53.87 (41.31 - 70.05) | 0.29 (0.26 - 0.33) | 987.79 (772.97 - 1220.97) | 298.86 (210.95 - 397.35) | 3435.4 (2766.8 - 4260.6) | 333.77 (244.84 - 445.32) | 0.48 (0.41 - 0.56) | 64.27 (49.11 - 79.77) | 25.82 (18.76 - 34.47) | 224.32 (174.95 - 286.25) | 28.64 (21.45 - 37.66) | 0.52 (0.45 - 0.59) | 1210.26 (958.49 - 1489.72) | 409.64 (308.44 - 533.23) | 4103.96 (3260.1 - 5177.39) | 445.14 (337.55 - 578.29) | 0.45 (0.39 - 0.52) |
| Nigeria | 3366.59 (2739.7 - 3964.55) | 55.03 (37.96 - 74.17) | 7918.11 (6586.03 - 9149.96) | 69.69 (49.13 - 92.79) | 0.84 (0.79 - 0.89) | 20257.68 (16514.21 - 24769.36) | 332.49 (243.07 - 437.26) | 48895.32 (40210.02 - 58806.54) | 429.43 (317.74 - 557.83) | 0.91 (0.82 - 1) | 1469.5 (1233.85 - 1710.49) | 28.81 (23.8 - 34.64) | 3202.42 (2659.28 - 3725.61) | 33.93 (27.19 - 39.87) | 0.78 (0.66 - 0.89) | 25435.07 (21646.54 - 29696.04) | 446.48 (374.4 - 530.75) | 53902.85 (44567.77 - 62499.26) | 515.93 (417.92 - 604.09) | 0.68 (0.58 - 0.77) |
| Niue | 0.28 (0.24 - 0.31) | 70.19 (51 - 92.65) | 0.26 (0.22 - 0.3) | 71.4 (52.11 - 93.86) | 0.06 (-0.01 - 0.13) | 2.06 (1.64 - 2.5) | 510.48 (374.47 - 669.3) | 2.02 (1.7 - 2.51) | 563.5 (427.46 - 734.66) | 0.41 (0.32 - 0.51) | 0.16 (0.14 - 0.18) | 36.75 (28.88 - 44.99) | 0.12 (0.1 - 0.13) | 34.12 (26.98 - 41.37) | -0.3 (-0.33 - -0.27) | 2.61 (2.25 - 2.94) | 633.4 (512.26 - 763.16) | 2.08 (1.77 - 2.36) | 588.39 (473.86 - 706.98) | -0.3 (-0.33 - -0.26) |
| North Macedonia | 209.12 (184.05 - 236.05) | 73.25 (55.07 - 96.39) | 467.12 (405.03 - 530) | 83.49 (65.03 - 105.64) | 0.46 (0.4 - 0.51) | 1440.66 (1193.47 - 1733.78) | 516.95 (392.92 - 677.75) | 3177.32 (2658.31 - 3730.18) | 582.58 (457.19 - 739.99) | 0.41 (0.31 - 0.5) | 82.37 (73.32 - 92.74) | 32.87 (27.91 - 39.44) | 166.02 (138.16 - 193.53) | 39.23 (30.96 - 47.17) | 0.41 (0.05 - 0.76) | 1467.97 (1324.56 - 1624.86) | 554.88 (474.26 - 657.23) | 2966.22 (2508.57 - 3443.52) | 609.94 (492.46 - 723.75) | 0.2 (-0.05 - 0.45) |
| Northern Mariana Islands | 1.26 (1.09 - 1.44) | 73.08 (54.75 - 95.1) | 5.38 (4.67 - 6.13) | 75.39 (58.84 - 94.65) | -0.13 (-0.24 - -0.01) | 9.11 (7.63 - 10.9) | 556.64 (426.58 - 716.64) | 40.53 (33.51 - 47.43) | 601.41 (467.59 - 741.8) | 0 (-0.16 - 0.17) | 0.41 (0.32 - 0.51) | 32.66 (24.19 - 41.95) | 1.52 (1.26 - 1.77) | 29 (22.68 - 35.97) | -0.44 (-0.57 - -0.31) | 8.76 (7.01 - 10.56) | 571.92 (440.74 - 716.44) | 32.38 (27.37 - 37.07) | 515.83 (418.53 - 624.78) | -0.36 (-0.42 - -0.3) |
| Norway | 356.08 (297.47 - 417.28) | 27.11 (18.83 - 36.33) | 1192.07 (998.06 - 1383.67) | 65.04 (43.99 - 88.44) | 3.41 (3.16 - 3.67) | 1656.34 (1342.48 - 2008.82) | 130.83 (94.99 - 175.31) | 9652.29 (7892.98 - 11594.09) | 521.88 (392.6 - 672.18) | 5.4 (4.97 - 5.84) | 323.88 (292.24 - 339.31) | 23.81 (21.44 - 25.11) | 623.58 (540.14 - 668.97) | 31.26 (27.1 - 33.59) | 1.29 (1.12 - 1.46) | 4876.42 (4501.21 - 5096.92) | 365.2 (335.31 - 384.11) | 9831.58 (8811.26 - 10609.07) | 513.33 (454.78 - 559.4) | 1.55 (1.37 - 1.72) |
| Oman | 61.55 (52.09 - 70.52) | 74.25 (54.96 - 96.15) | 246.16 (203.06 - 285.12) | 112.45 (80.98 - 148.64) | 1.44 (1.35 - 1.53) | 367.53 (282.57 - 449.29) | 438.28 (312.66 - 583) | 1617.39 (1283.38 - 1961.42) | 754.38 (538.71 - 990.47) | 1.85 (1.78 - 1.93) | 24.8 (19.04 - 31.58) | 32.52 (24.22 - 42.38) | 58.75 (48.85 - 70.11) | 34.34 (26.67 - 43.41) | 0.76 (0.42 - 1.1) | 473.08 (375.93 - 592.41) | 564.95 (432.04 - 722.64) | 1168.23 (984.38 - 1367.9) | 582.97 (465.22 - 716.98) | 0.54 (0.31 - 0.78) |
| Pakistan | 4692.01 (3865.82 - 5530.44) | 56.51 (38.5 - 76.51) | 10919.98 (9079.37 - 12707.91) | 67 (46.2 - 89.5) | 0.63 (0.6 - 0.66) | 30536.94 (24889.88 - 37212.03) | 361.76 (265.69 - 480.84) | 77359.35 (63203.34 - 93368.23) | 475.34 (354.24 - 619.6) | 0.95 (0.89 - 1.01) | 2075.5 (1695.71 - 2490.4) | 28.91 (22.55 - 36.5) | 4723.33 (3911.25 - 5733.63) | 36.21 (28.82 - 45.69) | 0.56 (0.39 - 0.73) | 36954.4 (31238.67 - 43130.4) | 468.15 (377.73 - 572.32) | 86038.95 (72723.52 - 102167.83) | 585.09 (472.48 - 721.47) | 0.56 (0.41 - 0.71) |
| Palau | 1.19 (1.01 - 1.39) | 82.91 (61.13 - 109.61) | 2.57 (2.14 - 3.2) | 83.06 (60.27 - 112.04) | -0.01 (-0.07 - 0.05) | 8.16 (6.56 - 9.77) | 583.13 (427.32 - 770.47) | 18.72 (15.38 - 24.11) | 628.03 (463.55 - 846.95) | 0.27 (0.18 - 0.35) | 0.39 (0.33 - 0.46) | 32.62 (26.21 - 39.9) | 0.67 (0.56 - 0.83) | 30.29 (23.59 - 38.03) | -0.04 (-0.14 - 0.06) | 7.84 (6.82 - 9.18) | 576.59 (471.23 - 696.94) | 14.34 (11.97 - 17.15) | 527.62 (420.56 - 648.64) | -0.13 (-0.2 - -0.05) |
| Palestine | 81.32 (68.98 - 91.77) | 64.64 (48.61 - 82.95) | 258.94 (220.6 - 295.51) | 77.91 (56.48 - 100.89) | 0.67 (0.6 - 0.74) | 500 (394.87 - 603.72) | 396.51 (283.46 - 516.83) | 1817.72 (1497.35 - 2158.5) | 555.89 (412.79 - 718.81) | 1.16 (1.05 - 1.28) | 40.11 (33.51 - 47.92) | 37.05 (29.36 - 46.04) | 76.01 (65.35 - 86.52) | 29.48 (23.9 - 35.27) | -0.79 (-1.09 - -0.49) | 664.63 (560.4 - 793.99) | 563.72 (451.88 - 694.25) | 1416.94 (1233.7 - 1586.51) | 478.66 (395.6 - 564.35) | -0.58 (-0.81 - -0.34) |
| Panama | 108.44 (96.9 - 120.22) | 46.84 (35.23 - 60.72) | 479.94 (420.95 - 552.13) | 63.77 (44.75 - 87.16) | 0.8 (0.71 - 0.89) | 871.03 (729.95 - 1006.49) | 373.26 (288.17 - 468) | 4218.58 (3494.42 - 4937.25) | 563.22 (423.38 - 726.54) | 1 (0.87 - 1.13) | 45.94 (42 - 48.56) | 20.9 (18.7 - 22.56) | 180.77 (141.14 - 214.07) | 23.75 (18.49 - 28.34) | 0.45 (0.2 - 0.69) | 808.9 (750.19 - 866.37) | 354.71 (316.75 - 389.85) | 3118.41 (2539.07 - 3661.72) | 415.92 (335.29 - 492.82) | 0.46 (0.27 - 0.64) |
| Papua New Guinea | 115.73 (96.1 - 137.97) | 52.38 (38.2 - 70.11) | 348.35 (291.48 - 401.97) | 56.4 (40.59 - 74.38) | 0.34 (0.29 - 0.4) | 806.91 (631.56 - 984.17) | 380.44 (277.26 - 504.59) | 2531.55 (2035.55 - 3022.17) | 420.84 (304.87 - 550.56) | 0.41 (0.33 - 0.5) | 48.5 (36.79 - 64.25) | 29.08 (21.16 - 40.24) | 132.21 (99.21 - 184.52) | 27.36 (19.71 - 39.33) | -0.19 (-0.24 - -0.14) | 1058.38 (803.36 - 1377.4) | 517.87 (384.65 - 692.2) | 2775.56 (2103.81 - 3743.96) | 488.11 (360.54 - 681.34) | -0.18 (-0.23 - -0.12) |
| Paraguay | 150.26 (130.29 - 175.33) | 44.16 (31.73 - 59.66) | 479.73 (410.04 - 538.3) | 52.13 (37.61 - 68.58) | 0.49 (0.44 - 0.53) | 1181 (956.44 - 1467.63) | 343.83 (258.07 - 459.37) | 4190.92 (3467.47 - 4887.74) | 453.09 (342.44 - 573.29) | 0.78 (0.71 - 0.85) | 59.47 (50.3 - 68.92) | 18.58 (15.21 - 22.68) | 228.38 (180.46 - 284.57) | 26.86 (20.12 - 34.19) | 1.52 (1.4 - 1.64) | 1042.47 (902.34 - 1192.56) | 313.43 (261.26 - 377.19) | 3941.45 (3172.01 - 4816.17) | 447.43 (347.48 - 557.65) | 1.37 (1.27 - 1.46) |
| Peru | 983.3 (835.55 - 1120.89) | 55.19 (41.3 - 72.48) | 4219.71 (3655.15 - 4886.64) | 76.88 (53.72 - 104.94) | 1.07 (1.05 - 1.09) | 8314.84 (6806.03 - 10104.66) | 452.16 (340.31 - 589.29) | 44597.01 (36972.28 - 54557.96) | 808.76 (625.07 - 1046.89) | 1.85 (1.81 - 1.89) | 516.85 (444.82 - 589.25) | 30.24 (24.93 - 36.39) | 1643.74 (1292.61 - 2059.02) | 29.91 (22.78 - 38.56) | -0.13 (-0.35 - 0.1) | 8788.94 (7714.97 - 9920.11) | 498.97 (416.91 - 590.83) | 29491.61 (24119.89 - 35777.96) | 539.63 (423.99 - 671.16) | 0.16 (-0.03 - 0.36) |
| Philippines | 1971.25 (1616.59 - 2317.44) | 50.79 (34.11 - 70.25) | 6145.06 (5087.05 - 7059.83) | 53.07 (35.8 - 72.61) | 0.1 (0.03 - 0.17) | 13961.17 (11396.09 - 17043.93) | 351.15 (259.14 - 465.44) | 46082.92 (38089.4 - 55321.39) | 398.47 (298.11 - 517.52) | 0.4 (0.3 - 0.49) | 731.16 (641.01 - 836.12) | 23.72 (19.96 - 28.29) | 2461.83 (2113.71 - 2886.81) | 25.04 (20.96 - 30.81) | 0.52 (0.43 - 0.6) | 13474.27 (11818.49 - 15353.46) | 376.17 (321.3 - 445.74) | 46505.98 (40271.4 - 52906.08) | 427.82 (364.24 - 515.24) | 0.63 (0.57 - 0.68) |
| Poland | 4775.69 (3923.29 - 5649.26) | 64.12 (43.28 - 87.85) | 9033.35 (8058.71 - 10123.25) | 69.4 (53.14 - 86.65) | 0.25 (0.23 - 0.28) | 36140.55 (29341.15 - 44354.97) | 487.57 (362.89 - 635.12) | 76237.82 (68371.82 - 85511.88) | 582.92 (493.12 - 685.74) | 0.54 (0.52 - 0.56) | 1887.08 (1775.52 - 1959.75) | 27.06 (25.14 - 28.27) | 3816.94 (3396.2 - 4129.66) | 28.48 (25.18 - 30.95) | -0.02 (-0.11 - 0.07) | 33380.58 (31504.12 - 35343.6) | 459.17 (424.97 - 495.33) | 64362.11 (58018.63 - 70436.93) | 488.24 (436.08 - 537.2) | 0.06 (-0.01 - 0.12) |
| Portugal | 1347.66 (1150.77 - 1534.71) | 54.86 (41.09 - 69.98) | 3431.54 (3002.17 - 3901.65) | 73.44 (53.43 - 94.12) | 0.96 (0.91 - 1) | 10178.39 (8374.11 - 12015.1) | 419.16 (315.82 - 524.85) | 29682.44 (23550.23 - 35265.57) | 612.28 (457.45 - 761.88) | 1.27 (1.19 - 1.36) | 521.31 (488 - 541.66) | 23.44 (21.42 - 24.9) | 1332.49 (1142.51 - 1435.78) | 24.15 (20.59 - 26.32) | 0.38 (0.22 - 0.53) | 9117.98 (8500.09 - 9725.23) | 384.05 (349.56 - 418.54) | 21390.07 (19008.85 - 23085.14) | 418.33 (364.92 - 466.46) | 0.53 (0.4 - 0.65) |
| Puerto Rico | 281.23 (245.42 - 305.75) | 45.98 (34.66 - 58.52) | 725.34 (648.08 - 796.01) | 50.82 (36.78 - 65.8) | 0.29 (0.22 - 0.36) | 2065.79 (1707.47 - 2348.61) | 334.74 (252.01 - 417.61) | 5785.73 (4733.23 - 6799.8) | 422.63 (311.97 - 531.5) | 0.71 (0.61 - 0.81) | 164 (153.96 - 171.06) | 28.17 (25.67 - 30.37) | 365.33 (299.01 - 421.82) | 23.46 (19.13 - 27.4) | -0.55 (-0.62 - -0.48) | 2660.74 (2522.25 - 2790.44) | 436.37 (397.22 - 474.73) | 5718.21 (4873.57 - 6507.87) | 395.36 (330.08 - 457.97) | -0.34 (-0.4 - -0.28) |
| Qatar | 8.44 (7.11 - 9.84) | 93.98 (68.7 - 122.94) | 98.07 (80.47 - 116.89) | 136.39 (102.07 - 178.11) | 1.33 (1.27 - 1.39) | 51.75 (41.63 - 63.14) | 557.58 (395.21 - 754.63) | 637.18 (505.93 - 776.24) | 905.89 (667.82 - 1188.16) | 1.74 (1.65 - 1.84) | 4.87 (4.18 - 5.58) | 76.3 (62.09 - 93.04) | 18.47 (14.34 - 23.27) | 38.97 (27.67 - 52.75) | -2.58 (-3.27 - -1.88) | 88.24 (76.87 - 100.77) | 1150.48 (941.96 - 1385.06) | 404.4 (323.47 - 514.39) | 666.07 (499.34 - 877.32) | -1.93 (-2.5 - -1.37) |
| Republic of Korea | 1632.29 (1397.21 - 1897.66) | 40.17 (29.44 - 52.65) | 9782.95 (8558.49 - 10750.82) | 59.6 (43.82 - 76.79) | 1.23 (1.15 - 1.3) | 11376.97 (9164.64 - 13935.16) | 273.91 (202.5 - 359.94) | 79997.7 (65776.99 - 93299.93) | 488.56 (375.83 - 615.62) | 1.78 (1.65 - 1.91) | 826.92 (717.98 - 1102.38) | 25.92 (21.37 - 35.22) | 3652.97 (2822.43 - 4314.3) | 22.98 (16.86 - 27.83) | -0.07 (-0.19 - 0.05) | 15327.3 (13586.6 - 19716.12) | 414.94 (347.62 - 552.42) | 62710.52 (50800.17 - 72713.69) | 387.74 (299.54 - 464.32) | 0.06 (-0.04 - 0.17) |
| Republic of Moldova | 416.04 (355.74 - 488.13) | 58.31 (41.77 - 78.48) | 491.69 (430.83 - 566.03) | 46.64 (32.71 - 62.22) | -0.95 (-1.02 - -0.89) | 3459.14 (2770.04 - 4261.03) | 501.41 (381.01 - 661.51) | 4488.13 (3630.24 - 5392.27) | 431.27 (330.22 - 554.44) | -0.73 (-0.82 - -0.65) | 146.67 (139.01 - 154.35) | 26.19 (24.25 - 27.95) | 169.82 (153.13 - 187.32) | 16.69 (14.78 - 18.46) | -1.81 (-2.06 - -1.55) | 2796.4 (2588.21 - 3022.13) | 444.48 (402.82 - 489.81) | 3313.34 (3007.07 - 3647.66) | 322.84 (286.44 - 362.89) | -1.34 (-1.53 - -1.15) |
| Romania | 2703.98 (2414.09 - 2978.3) | 57.81 (45.17 - 71.85) | 4359.47 (3961.39 - 4773.21) | 63.66 (48.95 - 80.85) | 0.27 (0.24 - 0.31) | 19087.34 (15817.26 - 22530.19) | 416.3 (325.91 - 517.36) | 34133.07 (28660.82 - 38739.43) | 490.35 (390.11 - 609.47) | 0.47 (0.41 - 0.53) | 1128.78 (1069.97 - 1183.32) | 27.98 (25.92 - 29.81) | 2169.3 (1968.97 - 2391.19) | 29.7 (26.23 - 33.06) | 0.17 (0.06 - 0.29) | 20305.58 (19121.6 - 21481.57) | 463.86 (427.37 - 502.23) | 35558.88 (32421.69 - 38751.27) | 499.26 (443.59 - 557.33) | 0.17 (0.08 - 0.26) |
| Russian Federation | 16696.3 (13771.39 - 19736.3) | 55.48 (37.27 - 76.12) | 24611.6 (20457.64 - 28523.2) | 57.83 (39.29 - 77.77) | 0.05 (-0.05 - 0.15) | 141747.08 (115329.85 - 173256.27) | 477.35 (360.9 - 617.68) | 208795.8 (170382.7 - 251178.88) | 495.41 (375.82 - 634.31) | 0.03 (-0.12 - 0.18) | 6050.57 (5737.84 - 6185.65) | 22.69 (21.3 - 23.34) | 10356.52 (9467.51 - 11114.42) | 25.19 (22.7 - 27.12) | 0.12 (-0.01 - 0.25) | 114913.83 (108202.92 - 122250.41) | 401.87 (372.14 - 433.07) | 180161.17 (165907.73 - 195358.06) | 434.88 (393.83 - 476.44) | 0.1 (0 - 0.2) |
| Rwanda | 156.11 (134.13 - 179.69) | 43.23 (31.61 - 57.62) | 365.18 (310.32 - 423.35) | 44.77 (32.33 - 59.47) | 0.18 (0.13 - 0.22) | 964.62 (771.15 - 1204.18) | 259.82 (187.61 - 348.73) | 2459.84 (1948.39 - 3037.01) | 300.18 (219.12 - 399.15) | 0.56 (0.5 - 0.63) | 90.09 (65.55 - 123.95) | 31.78 (21.18 - 44.73) | 177.63 (115.03 - 240.2) | 27.98 (17.48 - 39.8) | -0.92 (-1.12 - -0.71) | 1754.54 (1309.18 - 2397.79) | 520.37 (358.08 - 725.71) | 3283.75 (2268.37 - 4354.96) | 446.47 (296.48 - 616.73) | -0.99 (-1.19 - -0.79) |
| Saint Kitts and Nevis | 3.63 (3.19 - 4.12) | 54.02 (42.28 - 67.75) | 5.67 (4.98 - 6.48) | 60.8 (47.95 - 75.89) | 0.33 (0.29 - 0.36) | 20.94 (16.81 - 25.02) | 307.92 (228.25 - 403.32) | 41.42 (34.46 - 50.4) | 404.28 (304.84 - 519.22) | 0.77 (0.68 - 0.87) | 3.24 (3.07 - 3.4) | 50.73 (47.15 - 53.96) | 4.1 (3.55 - 4.53) | 53.32 (44.74 - 60.97) | 0.67 (0.45 - 0.9) | 53.36 (50.5 - 56.13) | 775.16 (719 - 830.34) | 71.51 (61.15 - 80.24) | 820.27 (685.07 - 936.92) | 0.66 (0.47 - 0.85) |
| Saint Lucia | 7.4 (6.66 - 8.37) | 54.38 (44.18 - 67.07) | 23.68 (21.18 - 26.2) | 59.83 (45.9 - 74.48) | 0.24 (0.2 - 0.29) | 46.7 (38.53 - 56.62) | 322.9 (246.83 - 417.8) | 163.31 (131.02 - 195.03) | 406.18 (298.79 - 517.73) | 0.53 (0.43 - 0.63) | 4.88 (4.64 - 5.1) | 39.89 (37.06 - 42.44) | 13.57 (11.35 - 15.66) | 35.26 (29.2 - 41.1) | -0.94 (-1.25 - -0.62) | 78.97 (75.03 - 83.05) | 586.63 (542.72 - 632) | 211.89 (179.44 - 243.33) | 540.22 (452.72 - 627.03) | -0.72 (-0.98 - -0.45) |
| Saint Vincent and the Grenadines | 5.08 (4.49 - 5.62) | 43.29 (33.09 - 55.02) | 12.21 (11.11 - 13.47) | 52.57 (42.37 - 64.81) | 0.59 (0.56 - 0.62) | 33.4 (27.36 - 40.26) | 279.32 (206.43 - 366.24) | 85.18 (70.84 - 101.93) | 355.33 (274.15 - 448.64) | 0.7 (0.63 - 0.76) | 3.08 (2.88 - 3.24) | 27.65 (25.38 - 29.77) | 6.97 (6.3 - 7.66) | 32.34 (28.83 - 35.99) | 0.62 (0.48 - 0.77) | 50.92 (47.53 - 53.96) | 431.25 (393.73 - 469.46) | 112.99 (102.33 - 124.44) | 496.33 (442.34 - 554.61) | 0.53 (0.4 - 0.66) |
| Samoa | 7.8 (6.78 - 8.89) | 63.77 (47.84 - 84.95) | 14.33 (12.37 - 16.1) | 67.01 (49.49 - 87.8) | 0.25 (0.19 - 0.3) | 55.42 (44.4 - 67.11) | 462.87 (343.96 - 612.67) | 109.73 (90.89 - 131.01) | 523.14 (392.71 - 667.98) | 0.52 (0.42 - 0.61) | 3.55 (2.91 - 4.4) | 34.43 (26.73 - 44.17) | 5.85 (4.75 - 7.55) | 31.37 (24.04 - 41.59) | -0.29 (-0.36 - -0.22) | 66.89 (55.02 - 82.43) | 585.39 (466.07 - 741.6) | 108.56 (90.19 - 137.76) | 537.66 (422.45 - 693.1) | -0.25 (-0.31 - -0.19) |
| San Marino | 4.6 (3.88 - 5.31) | 71.79 (51.22 - 95.29) | 10.76 (8.78 - 13.07) | 79.34 (54.13 - 113.46) | 0.25 (0.18 - 0.31) | 42.39 (35.07 - 49.92) | 659.29 (508.8 - 824.58) | 111.65 (88.14 - 141.8) | 754.53 (555.25 - 1015.53) | 0.31 (0.22 - 0.41) | 1.29 (1.07 - 1.47) | 19.69 (15.63 - 23.58) | 1.72 (1.17 - 2.33) | 10.13 (6.69 - 14.34) | -1.03 (-1.46 - -0.6) | 23.59 (20.39 - 26.79) | 361.14 (297.24 - 428.36) | 37.31 (28.39 - 47.3) | 246.32 (178.64 - 323.91) | -0.62 (-0.88 - -0.36) |
| Sao Tome and Principe | 5.48 (4.74 - 6.35) | 55.83 (42.08 - 74.04) | 10.34 (8.95 - 11.9) | 71.8 (55.45 - 93.14) | 0.89 (0.84 - 0.95) | 33.82 (26.78 - 41.33) | 344.72 (250.36 - 466.69) | 66.59 (53.62 - 81.31) | 461.04 (344.38 - 611.47) | 0.99 (0.89 - 1.08) | 2.5 (2.21 - 2.77) | 28.64 (23.23 - 34.47) | 4.26 (3.53 - 5.03) | 34.35 (26.64 - 42.33) | 0.9 (0.8 - 1) | 41.5 (36.49 - 46.01) | 443.78 (363.63 - 528.02) | 73.16 (62.03 - 84.32) | 541.29 (431.06 - 655.09) | 0.86 (0.78 - 0.93) |
| Saudi Arabia | 570.18 (487.72 - 644.92) | 78.49 (59.54 - 99.14) | 1878.38 (1592.23 - 2248.34) | 103.72 (75.8 - 138.05) | 0.86 (0.8 - 0.92) | 3240.57 (2536.96 - 3928.11) | 444.87 (320.23 - 580.63) | 13140.87 (10791.75 - 16666.36) | 734.05 (542.56 - 995.55) | 1.58 (1.51 - 1.66) | 326.72 (249.34 - 408.03) | 50.74 (37.77 - 66.99) | 619.95 (511.87 - 753.6) | 47.61 (37.61 - 61.07) | -0.36 (-0.44 - -0.28) | 5597.46 (4355.42 - 6919.33) | 792.91 (601.97 - 1023.9) | 12514.24 (10594.95 - 15053.04) | 770.09 (622.98 - 971.78) | -0.22 (-0.28 - -0.15) |
| Senegal | 263.51 (227.85 - 297.38) | 60.2 (46.93 - 75.98) | 744.24 (650.64 - 846.36) | 70.39 (55.43 - 88.48) | 0.47 (0.43 - 0.51) | 1496.58 (1201.4 - 1848.53) | 340.01 (252.57 - 448.22) | 4514 (3685.49 - 5484.92) | 428.59 (325.41 - 555.31) | 0.64 (0.56 - 0.71) | 107.85 (88.17 - 129.22) | 28.95 (22.68 - 36.4) | 313.66 (245.6 - 389.69) | 34.82 (26.08 - 44.77) | 0.67 (0.59 - 0.74) | 1886.04 (1562.22 - 2233.98) | 454.41 (364.26 - 561.48) | 5351.02 (4298.86 - 6484.79) | 541.03 (414.2 - 680.52) | 0.62 (0.55 - 0.7) |
| Serbia | 1101.17 (979.39 - 1241.19) | 64.14 (48.07 - 82.57) | 2192.75 (1889.97 - 2455.27) | 71.52 (53.32 - 88.66) | 0.45 (0.41 - 0.48) | 7983.15 (6689.91 - 9450.01) | 478.84 (372.09 - 608.85) | 16157.09 (12472.55 - 18741.57) | 529.85 (383.78 - 657.2) | 0.48 (0.43 - 0.53) | 529.85 (468.88 - 591.99) | 41.38 (34.65 - 49.38) | 864.73 (754.74 - 988.7) | 28.42 (23.16 - 33.71) | -1.3 (-1.44 - -1.17) | 8904.68 (7932.14 - 9831) | 636.5 (541.44 - 752.88) | 14756.78 (13006.99 - 16467.85) | 480.88 (398.88 - 566.08) | -0.92 (-1 - -0.85) |
| Seychelles | 6.62 (5.9 - 7.45) | 68.54 (52.24 - 88.27) | 14.52 (12.76 - 16.52) | 83.35 (63.13 - 108.35) | 0.63 (0.6 - 0.66) | 43.55 (36.45 - 52.76) | 450.81 (344.38 - 587.79) | 100.42 (85.04 - 120.53) | 574.31 (434.66 - 750) | 0.75 (0.67 - 0.83) | 3.9 (3.33 - 4.42) | 41.12 (33.26 - 48.73) | 5.85 (4.64 - 6.96) | 37.6 (28.08 - 47.01) | -0.06 (-0.24 - 0.12) | 65.21 (56.13 - 73) | 676.85 (559.12 - 790.84) | 102.85 (85.06 - 119.67) | 620.74 (487.25 - 757.05) | -0.1 (-0.25 - 0.06) |
| Sierra Leone | 158.79 (138.13 - 179.91) | 51.67 (39.26 - 64.94) | 290.96 (254.69 - 326.23) | 57.25 (43.83 - 74.11) | 0.32 (0.26 - 0.38) | 921.13 (727.96 - 1119.32) | 301.85 (218.59 - 394.56) | 1804.47 (1467.96 - 2189.29) | 355.96 (267.14 - 469.94) | 0.49 (0.37 - 0.61) | 71.29 (60.07 - 84.52) | 26.9 (21.31 - 33.54) | 122.97 (95.9 - 152.92) | 28.56 (21.57 - 36.73) | 0.36 (0.27 - 0.45) | 1222.41 (1032.56 - 1446.48) | 419.12 (337.33 - 516.45) | 2135.38 (1696.35 - 2642.44) | 448.3 (343.31 - 569.5) | 0.38 (0.3 - 0.46) |
| Singapore | 145.19 (130.31 - 162.71) | 45.65 (34.38 - 58.7) | 782.88 (683.92 - 897) | 55.23 (39.23 - 73.22) | 0.6 (0.51 - 0.7) | 1059.5 (903.44 - 1251.77) | 332.72 (259.63 - 419.24) | 6293.69 (5201.22 - 7597.91) | 445.62 (333.18 - 574.18) | 0.92 (0.76 - 1.09) | 62.77 (59.33 - 65.2) | 22.05 (20.17 - 23.63) | 239.32 (209.67 - 256.72) | 17.87 (15.44 - 19.56) | -0.4 (-0.57 - -0.23) | 1189.15 (1119.62 - 1253.33) | 385.25 (351.51 - 418.8) | 4361.78 (3916.74 - 4742.92) | 316.98 (276.31 - 356.9) | -0.38 (-0.54 - -0.22) |
| Slovakia | 596.35 (520.89 - 664.6) | 57.19 (41.89 - 73.94) | 1006.55 (891.6 - 1138.65) | 58.47 (43.13 - 76.91) | 0.01 (-0.03 - 0.05) | 4664.04 (3829.82 - 5606.03) | 452.7 (341.79 - 578.55) | 8131.84 (6742.83 - 9733.26) | 477.4 (370.23 - 612.13) | 0.1 (0.05 - 0.14) | 273.98 (247.36 - 307.54) | 27.63 (23.44 - 32.79) | 384.91 (331.11 - 437.02) | 23.47 (18.88 - 28.05) | -0.31 (-0.4 - -0.22) | 4826.06 (4368.75 - 5350.3) | 472.58 (406.83 - 554.66) | 6787.77 (5924.98 - 7641.86) | 403.94 (331.81 - 476.08) | -0.39 (-0.46 - -0.32) |
| Slovenia | 274.09 (246.46 - 298.57) | 64.99 (49.93 - 83.15) | 538.34 (461.08 - 592.76) | 67.44 (50.49 - 83.54) | 0.1 (0.05 - 0.15) | 2281.42 (1972.17 - 2625.56) | 541.04 (435.67 - 672.53) | 4707.5 (3836.29 - 5438.03) | 568.09 (435.97 - 689.12) | 0.12 (0.06 - 0.19) | 109.5 (102.77 - 115.4) | 25.85 (23.66 - 27.73) | 250.64 (213.25 - 279.74) | 27.27 (23.03 - 30.71) | 0.05 (-0.1 - 0.2) | 1945.47 (1812.59 - 2080.54) | 457.57 (416.25 - 500.34) | 3997.32 (3474.83 - 4456.56) | 459.95 (394.09 - 520.2) | -0.11 (-0.23 - 0.02) |
| Solomon Islands | 10.97 (9.2 - 13.03) | 64.09 (47.13 - 85.82) | 29.19 (24.65 - 33.48) | 66.44 (49.48 - 86.99) | 0.17 (0.13 - 0.21) | 70.79 (56.58 - 88.12) | 422.79 (306.35 - 575.82) | 201.84 (161.62 - 247.29) | 464.48 (343.13 - 619.01) | 0.36 (0.28 - 0.44) | 4.23 (3.14 - 5.22) | 33.56 (25.4 - 42.23) | 10.85 (8.61 - 14.24) | 30.79 (23.67 - 40.72) | -0.35 (-0.4 - -0.31) | 89.83 (65.34 - 110.47) | 583.06 (436.49 - 724.22) | 216.49 (173.27 - 272.74) | 533.72 (417.14 - 694.36) | -0.35 (-0.41 - -0.29) |
| Somalia | 99.7 (84.68 - 116.34) | 39.34 (28.65 - 52.03) | 271.61 (224.74 - 328.52) | 39.92 (29.25 - 53.08) | 0.04 (0 - 0.07) | 630.23 (500.24 - 779.1) | 238.56 (169.47 - 318.91) | 1766.76 (1373.73 - 2214.58) | 254.08 (181.85 - 345.1) | 0.13 (0.08 - 0.19) | 45.98 (33.27 - 60.04) | 22.83 (15.51 - 31.96) | 111.73 (72.37 - 154.64) | 22.63 (13.56 - 33.73) | 0.13 (0.08 - 0.18) | 918.17 (695.68 - 1190.06) | 386.21 (275.71 - 523.13) | 2370.32 (1646.84 - 3163.19) | 385.21 (249.26 - 548.76) | 0.1 (0.06 - 0.14) |
| South Africa | 1246.51 (1025.75 - 1458.32) | 42.2 (28.3 - 58.48) | 3444.9 (2851.31 - 4022.05) | 52.39 (35.89 - 70.82) | 0.72 (0.67 - 0.77) | 8647.68 (7143.86 - 10571.03) | 289.22 (212.03 - 382.96) | 24014.29 (19827.92 - 28879.85) | 365.44 (270.76 - 476.66) | 0.74 (0.66 - 0.82) | 465.6 (381.46 - 578.41) | 17.43 (13.93 - 22.24) | 1406.26 (1258.99 - 1524.59) | 24.68 (20.75 - 27.63) | 1.21 (0.87 - 1.55) | 7889.01 (6539.76 - 9632.28) | 276.64 (225.27 - 346.38) | 24476.28 (21884.48 - 26609.71) | 391.5 (333.88 - 439.44) | 1.18 (0.87 - 1.49) |
| South Sudan | 154.96 (130.29 - 180.59) | 41.75 (30.57 - 55.2) | 196.86 (162.12 - 228.22) | 42.89 (30.96 - 56.88) | 0.07 (0.05 - 0.09) | 968.79 (764.47 - 1234.56) | 260.63 (184.95 - 349.93) | 1335.38 (1010.65 - 1626.62) | 286.4 (198.65 - 378.86) | 0.3 (0.28 - 0.32) | 79.68 (63.01 - 100.14) | 25.34 (18.67 - 33.92) | 101.51 (75.64 - 136.85) | 26.77 (19.11 - 37.78) | 0.14 (0.11 - 0.18) | 1505.67 (1207.91 - 1881.6) | 422.77 (318.59 - 551.48) | 1901.47 (1450.6 - 2478.99) | 440.71 (323.88 - 604.78) | 0.07 (0.02 - 0.13) |
| Spain | 9474.17 (8721.08 - 10527.62) | 95.18 (75.9 - 116.06) | 19369.91 (16862.25 - 21613.94) | 110.23 (82.71 - 140.49) | 0.39 (0.15 - 0.63) | 79352.53 (71998.51 - 88273.95) | 811.91 (684.71 - 961.21) | 189788.1 (158109.06 - 223262.62) | 993.47 (787.98 - 1220.7) | 0.52 (0.24 - 0.8) | 2682.31 (2450.19 - 2811.52) | 28.3 (25.36 - 30.34) | 6041.56 (5114.25 - 6581.31) | 27.62 (23.32 - 30.28) | 0.24 (0.15 - 0.33) | 48857.48 (44653.49 - 52580.86) | 500.81 (445.69 - 551.55) | 103304.62 (90965.28 - 114120.65) | 519.85 (447.84 - 588.83) | 0.32 (0.2 - 0.44) |
| Sri Lanka | 785.37 (663.42 - 891.34) | 51.7 (36.58 - 67.17) | 2788.32 (2319.27 - 3246.99) | 61.6 (41.86 - 84.37) | 0.58 (0.53 - 0.63) | 5794.5 (4572.99 - 6894.39) | 381.82 (278.1 - 486.82) | 22531.39 (17630.52 - 28099.17) | 507.62 (369.73 - 675.41) | 0.97 (0.91 - 1.03) | 357.49 (319.88 - 404.37) | 28.67 (24.04 - 33.84) | 932.65 (672.19 - 1201.35) | 24.07 (16.81 - 31.97) | -0.06 (-0.26 - 0.15) | 6412.18 (5781.86 - 7159.05) | 459.39 (390.83 - 540.15) | 17349.83 (13092.92 - 21874) | 411.27 (299.65 - 532.03) | 0.03 (-0.13 - 0.19) |
| Sudan | 674.73 (580.89 - 768.55) | 50.29 (37.83 - 65.19) | 1687.01 (1429.07 - 1942.69) | 63.07 (45.68 - 83.01) | 0.8 (0.74 - 0.85) | 4270.33 (3455.3 - 5157.23) | 318.48 (234.9 - 419.74) | 12607.04 (10183.49 - 15436.55) | 478.01 (347.85 - 623.95) | 1.37 (1.27 - 1.48) | 288.79 (234.58 - 382.01) | 25.33 (19.12 - 34.87) | 579.51 (448.97 - 770.67) | 25.24 (18.52 - 34.85) | 0.03 (-0.03 - 0.09) | 5565.38 (4550.03 - 7151.11) | 431.15 (334 - 578.31) | 11152.58 (8993.92 - 14373.62) | 441.82 (339.73 - 589.15) | 0.11 (0.05 - 0.17) |
| Suriname | 14.01 (12.59 - 15.71) | 37.3 (29.02 - 47.73) | 45.84 (41.1 - 51.55) | 45.58 (35.28 - 58.17) | 0.62 (0.58 - 0.65) | 95.93 (80.21 - 115.27) | 244.26 (182.82 - 318.22) | 338.91 (271.75 - 408.97) | 329.15 (248.13 - 428.99) | 0.82 (0.75 - 0.89) | 7.92 (7.04 - 9.21) | 22.47 (18.84 - 27.85) | 21.17 (15.97 - 26.5) | 22.38 (16.29 - 28.96) | 0.33 (0.16 - 0.51) | 135.43 (122.14 - 151.01) | 370.63 (316.17 - 448.43) | 372.56 (293.85 - 452.38) | 377.64 (286.5 - 474.94) | 0.34 (0.18 - 0.51) |
| Sweden | 1400.56 (1147.13 - 1682.74) | 49.66 (32.81 - 69.06) | 3300 (2722.81 - 3879.71) | 80.6 (55.68 - 109.62) | 1.72 (1.64 - 1.79) | 11527.5 (9361.86 - 14145.34) | 405.88 (303.68 - 529.77) | 28608.89 (23153.44 - 34379.64) | 676.88 (508.78 - 874.34) | 1.74 (1.67 - 1.81) | 779.01 (707.97 - 815.31) | 25.32 (22.75 - 26.97) | 1189.96 (1017.52 - 1312.46) | 25.4 (21.5 - 28.38) | 0.06 (-0.19 - 0.31) | 12514.82 (11510.3 - 13249.27) | 416.81 (375.59 - 454.91) | 19554.78 (17282.33 - 21688.73) | 440.96 (378.6 - 502.16) | 0.22 (0.02 - 0.43) |
| Switzerland | 1429.3 (1220.39 - 1644.53) | 74.59 (54.03 - 100.01) | 3063.75 (2613.12 - 3587.83) | 93.07 (68.53 - 124.2) | 0.75 (0.67 - 0.83) | 12014.94 (9823.05 - 14233.38) | 617.52 (471.02 - 787.72) | 28738.23 (23275.34 - 34457.03) | 825.7 (636.09 - 1049.91) | 0.98 (0.87 - 1.08) | 527.91 (475.36 - 559.82) | 25.59 (22.86 - 27.55) | 963.61 (805.9 - 1051.6) | 24.81 (20.73 - 27.35) | 0.24 (0.11 - 0.37) | 9013.83 (8178.4 - 9714.45) | 449.61 (400.5 - 497.58) | 16457.55 (14230.99 - 18246.58) | 455.37 (387.52 - 520.49) | 0.31 (0.2 - 0.41) |
| Syrian Arab Republic | 371.22 (328.65 - 422.86) | 53.37 (39.93 - 69.6) | 1283.72 (1088.27 - 1512.15) | 70.49 (51.7 - 93.65) | 1.03 (0.95 - 1.11) | 2384.95 (1967.62 - 2928.13) | 337.42 (248.44 - 444.66) | 9387.19 (7726.87 - 11549.68) | 522.39 (395.01 - 695.81) | 1.62 (1.5 - 1.74) | 206.17 (171.32 - 256.42) | 33.82 (26.79 - 43.98) | 487.56 (382.42 - 605.45) | 35.6 (27.48 - 45.02) | 0.04 (-0.09 - 0.16) | 3435.47 (2869.31 - 4132.35) | 519.95 (415.54 - 659.07) | 8907.29 (7204.74 - 10975.34) | 550.71 (432.8 - 687.05) | 0.05 (-0.06 - 0.16) |
| Taiwan (Province of China) | 1195.67 (1122.3 - 1275.39) | 53.68 (45.08 - 62.55) | 8753.97 (8381.71 - 9175.89) | 117.51 (102.24 - 133.77) | 2.98 (2.59 - 3.38) | 7865.48 (7073.15 - 8670.93) | 345.58 (293.63 - 402.71) | 81713.85 (77986.17 - 85759.8) | 1098.01 (1010.94 - 1191.33) | 4.42 (3.82 - 5.03) | 573.32 (544.14 - 594.08) | 30.54 (27.85 - 32.56) | 2106.49 (1837.81 - 2291.84) | 28.61 (24.73 - 31.42) | -0.07 (-0.15 - 0.02) | 10865.35 (10320.12 - 11367.32) | 504.46 (463.17 - 541.35) | 40929.43 (36454.16 - 44942.63) | 557.41 (486.54 - 623.25) | 0.53 (0.42 - 0.64) |
| Tajikistan | 280.34 (240.42 - 321.16) | 69.13 (47.24 - 94.86) | 681.6 (595.6 - 773.04) | 99.46 (75.84 - 126.69) | 1.27 (1.18 - 1.35) | 1882.26 (1450.6 - 2322.36) | 463.88 (328.48 - 632.85) | 3969.89 (3157.32 - 4811.51) | 578 (425.53 - 764.84) | 0.7 (0.64 - 0.76) | 177.21 (120.96 - 293.59) | 46.33 (30.15 - 78.1) | 220.2 (179.05 - 267.2) | 39.05 (30.18 - 48.81) | -0.76 (-1.07 - -0.45) | 2780.04 (2046.88 - 4181.11) | 709.18 (499.11 - 1099.3) | 3941.71 (3329.64 - 4648.81) | 630.6 (503.59 - 771.02) | -0.55 (-0.84 - -0.27) |
| Thailand | 2941.47 (2588.01 - 3398.04) | 61.24 (44.93 - 81.87) | 11684.77 (10399.14 - 13092.77) | 62.78 (47.7 - 81.19) | 0.1 (0.07 - 0.13) | 19760.91 (16555.28 - 24635.65) | 405.09 (301.5 - 549.22) | 91262.74 (77242.24 - 107608.1) | 492.57 (385.43 - 622.48) | 0.68 (0.63 - 0.72) | 1385.54 (1157.39 - 1638.79) | 33.9 (26.65 - 42.33) | 5238.66 (4096.24 - 6533.88) | 29.01 (21.87 - 37.76) | -0.99 (-1.17 - -0.81) | 24360.61 (20892.56 - 28067.53) | 541.81 (437.73 - 660.07) | 88035.17 (72053.45 - 108127.44) | 483.98 (377.77 - 616.77) | -0.78 (-0.94 - -0.63) |
| Timor-Leste | 14.19 (12.24 - 16.1) | 45.66 (34.82 - 59.55) | 75.44 (64.94 - 86.69) | 57.67 (43.65 - 74.51) | 0.84 (0.8 - 0.87) | 97.2 (80.42 - 115.8) | 305.9 (227.02 - 398.63) | 528.66 (425.4 - 657.73) | 401.38 (296.45 - 526.72) | 0.97 (0.92 - 1.02) | 5.44 (4.16 - 6.8) | 22.13 (16.14 - 29.47) | 27.13 (20.91 - 34.77) | 25.17 (18.74 - 33.42) | 0.59 (0.47 - 0.71) | 104.52 (83.54 - 127.53) | 371.18 (281.38 - 477.89) | 517.01 (412.84 - 635.88) | 420.69 (321.68 - 544.08) | 0.56 (0.46 - 0.67) |
| Togo | 80.21 (69.56 - 91.8) | 52.64 (40.29 - 67.03) | 263.27 (217.16 - 310.26) | 58.49 (43.92 - 75.97) | 0.32 (0.26 - 0.38) | 480.67 (394.1 - 585.5) | 314.68 (233.21 - 412.63) | 1641.97 (1282.82 - 2056.43) | 369.77 (260.85 - 497.67) | 0.49 (0.39 - 0.59) | 31.03 (24.31 - 38.66) | 25.11 (19.01 - 33.07) | 101.66 (75.28 - 135.81) | 30.04 (21.52 - 40.98) | 0.65 (0.58 - 0.73) | 557.47 (441.26 - 683.62) | 396.25 (307.7 - 511.81) | 1879.73 (1402.71 - 2431.06) | 470.31 (342.86 - 624.27) | 0.62 (0.55 - 0.68) |
| Tokelau | 0.14 (0.12 - 0.16) | 62.29 (44.17 - 85.46) | 0.16 (0.15 - 0.19) | 63.76 (46.68 - 85.09) | 0.12 (0.06 - 0.19) | 0.99 (0.8 - 1.24) | 449.66 (327.38 - 613.27) | 1.31 (1.11 - 1.56) | 512.8 (386.96 - 656.66) | 0.52 (0.44 - 0.6) | 0.08 (0.06 - 0.1) | 38.35 (29.72 - 49.84) | 0.08 (0.06 - 0.1) | 30.71 (22.77 - 40.89) | -0.76 (-0.79 - -0.73) | 1.43 (1.18 - 1.78) | 650.89 (514.01 - 833.52) | 1.36 (1.1 - 1.72) | 529.51 (406.6 - 686.1) | -0.7 (-0.72 - -0.68) |
| Tonga | 4.42 (3.7 - 5.08) | 54.14 (38.67 - 73.61) | 7.52 (6.45 - 8.83) | 58.38 (42.7 - 76.52) | 0.31 (0.25 - 0.37) | 33.21 (26.68 - 40.12) | 418.19 (305.53 - 549.34) | 60.77 (50.38 - 73.4) | 473.95 (361.11 - 612.86) | 0.49 (0.4 - 0.58) | 1.77 (1.36 - 2.46) | 25.37 (18.79 - 36.23) | 3.25 (2.43 - 4.54) | 25.97 (18.66 - 36.81) | 0.17 (0.05 - 0.29) | 34.41 (27.41 - 46.61) | 446.18 (341.22 - 615.04) | 57.59 (44.66 - 77.93) | 452.42 (336.1 - 621.55) | 0.13 (0.02 - 0.24) |
| Trinidad and Tobago | 54.82 (48.24 - 60.63) | 42.75 (32.92 - 53.97) | 151.48 (135.83 - 173.17) | 46.46 (35.12 - 61.8) | 0.18 (0.12 - 0.24) | 373.05 (309.94 - 439.42) | 279.22 (210.02 - 358.82) | 1151.35 (934.55 - 1412.66) | 347.64 (261.8 - 461.9) | 0.58 (0.47 - 0.68) | 32.45 (30.92 - 33.68) | 28.01 (26.01 - 29.82) | 73.81 (57.81 - 89.42) | 24.23 (18.93 - 29.59) | -0.41 (-0.52 - -0.29) | 553.34 (527.24 - 580.44) | 434.86 (401.46 - 468.86) | 1287.73 (1061.38 - 1533.26) | 403.53 (323.82 - 489.57) | -0.29 (-0.41 - -0.17) |
| Tunisia | 385.01 (321.57 - 454.87) | 52.87 (38.66 - 69.24) | 1391.74 (1174.78 - 1583.19) | 66.01 (48.17 - 88.98) | 0.79 (0.75 - 0.82) | 2570.83 (2064.55 - 3188.63) | 359.4 (259.06 - 477.76) | 10884.01 (8708.21 - 12956.43) | 523.77 (386.01 - 675.69) | 1.32 (1.26 - 1.38) | 136.78 (110.56 - 177.41) | 23.31 (17.83 - 31.79) | 455.56 (332.77 - 602.29) | 24.14 (17.1 - 33.23) | 0.09 (-0.02 - 0.21) | 2578.17 (2109.65 - 3210.78) | 383.57 (299.93 - 506.02) | 8280.58 (6314.87 - 10586.58) | 412.54 (307.38 - 546.95) | 0.23 (0.15 - 0.32) |
| Turkey | 2482.26 (2165.13 - 2794.09) | 51.74 (37.29 - 68.44) | 10526.01 (8903.86 - 12320.38) | 70.36 (47.69 - 97.1) | 1.04 (0.99 - 1.1) | 16508.57 (13643.95 - 20012.65) | 337.63 (248.79 - 444.13) | 79868.59 (63408.37 - 98853.03) | 539.73 (386.14 - 717.56) | 1.63 (1.55 - 1.72) | 1610.4 (1391.22 - 1858.98) | 38.29 (30.64 - 47.6) | 4116.73 (3417.94 - 4913.54) | 30.82 (24.38 - 38.73) | -0.61 (-0.91 - -0.3) | 26054.66 (22619.23 - 29499.87) | 575.61 (470.51 - 702.67) | 69688.52 (58624.61 - 81280.98) | 491.63 (397.24 - 602.3) | -0.44 (-0.67 - -0.21) |
| Turkmenistan | 120.77 (104.26 - 137.4) | 42.23 (30.51 - 55.53) | 264.17 (234.94 - 293.92) | 44.68 (33.27 - 55.41) | 0.27 (0.21 - 0.33) | 960.34 (781.53 - 1199) | 347.39 (268.15 - 449.67) | 2025.41 (1707.49 - 2380.37) | 363.51 (280.65 - 445.86) | 0.26 (0.17 - 0.36) | 44.3 (41.64 - 46.74) | 18.7 (17.31 - 19.92) | 98.29 (78.54 - 119.31) | 20.6 (16.49 - 25.12) | -0.07 (-0.3 - 0.17) | 852.21 (788.99 - 925.43) | 329.58 (300.83 - 360.75) | 1900.61 (1560.38 - 2284.46) | 367.99 (300.01 - 443.07) | 0.08 (-0.11 - 0.27) |
| Tuvalu | 0.63 (0.54 - 0.73) | 66.72 (49.75 - 86.91) | 1.1 (0.96 - 1.24) | 69.31 (51.09 - 92.13) | 0.2 (0.16 - 0.25) | 4.16 (3.44 - 5.18) | 449.36 (338.57 - 594.5) | 7.92 (6.73 - 9.43) | 507.41 (381.1 - 661.29) | 0.52 (0.45 - 0.59) | 0.28 (0.23 - 0.32) | 38.09 (29.39 - 47.58) | 0.45 (0.37 - 0.57) | 34.01 (25.84 - 44.73) | -0.34 (-0.36 - -0.32) | 5.83 (4.89 - 6.76) | 663.28 (527.3 - 813.4) | 8.83 (7.21 - 10.89) | 586.81 (459.87 - 754.64) | -0.37 (-0.39 - -0.35) |
| Uganda | 388.28 (330.54 - 446.74) | 44.04 (32.29 - 57.49) | 932.24 (795.43 - 1053.92) | 48.78 (35.37 - 63.6) | 0.41 (0.36 - 0.45) | 2429.87 (1941.64 - 3008.46) | 273.28 (198.86 - 364.19) | 6192.16 (4918.07 - 7391.75) | 322.39 (234.67 - 421.27) | 0.62 (0.53 - 0.7) | 175.34 (117.62 - 237.67) | 23.66 (15.28 - 34.34) | 413.83 (278.81 - 537.55) | 26.03 (16.99 - 35.8) | 0.13 (0.01 - 0.25) | 3291.23 (2361.54 - 4480.94) | 392.11 (265.78 - 556.25) | 7563.28 (5422.14 - 9601.8) | 425.21 (294.32 - 566.77) | 0.07 (-0.04 - 0.19) |
| Ukraine | 7330.92 (6060.91 - 8740.65) | 59.72 (39.79 - 82.39) | 8396.96 (6993.68 - 9712.83) | 60.1 (40.43 - 82.22) | -0.07 (-0.15 - 0) | 65578.15 (53121.7 - 79770.04) | 537.21 (404.26 - 697.15) | 73191.07 (59589.66 - 87793.48) | 522.12 (393.73 - 667.86) | -0.19 (-0.29 - -0.08) | 2352.85 (2098.76 - 2587.67) | 21.11 (18.44 - 23.83) | 3147.69 (2490.68 - 3840.62) | 22.5 (17.61 - 27.72) | -0.19 (-0.37 - 0) | 46145.58 (40867.7 - 51897.55) | 389.2 (336.35 - 447.46) | 57618.5 (46867.78 - 69159.51) | 410.8 (329.37 - 501.39) | -0.12 (-0.25 - 0.01) |
| United Arab Emirates | 38.76 (33.35 - 43.59) | 95.38 (71.2 - 122.86) | 335.83 (258.34 - 419.07) | 105.73 (75.41 - 143.22) | 0.19 (0.05 - 0.33) | 219.91 (173.31 - 269.22) | 529.81 (384.87 - 698.86) | 2438.63 (1951.9 - 3109.95) | 766.27 (548.74 - 1035.19) | 1.13 (0.94 - 1.32) | 11.57 (8.02 - 15.19) | 33.15 (22.53 - 44.06) | 36.62 (29.22 - 43.87) | 30.26 (23.12 - 38.48) | 2.03 (1.26 - 2.81) | 232.96 (167.85 - 299.45) | 583.62 (413.54 - 758.55) | 1078.82 (897.67 - 1282.36) | 545.22 (429.64 - 677.7) | 1.63 (1.03 - 2.23) |
| United Kingdom | 12704.78 (10768.45 - 14759.26) | 76.8 (54.09 - 102.89) | 21568.32 (18396.11 - 24652.12) | 89.98 (64.22 - 119.29) | 0.57 (0.48 - 0.66) | 117077.58 (98375.19 - 139273.56) | 700.91 (553.42 - 878.83) | 189783.6 (160460.22 - 221545.77) | 772.74 (603.6 - 961.36) | 0.34 (0.15 - 0.54) | 5114.04 (4735.14 - 5284.28) | 29.64 (27.29 - 30.78) | 8023.9 (7020.99 - 8492.64) | 29.99 (26.32 - 31.78) | 0.62 (0.43 - 0.82) | 90698.33 (84661.46 - 96692.68) | 528.48 (483.01 - 573.69) | 133497.03 (121236.18 - 144372.56) | 524.7 (466.24 - 576) | 0.43 (0.26 - 0.6) |
| United Republic of Tanzania | 586.33 (510.29 - 666.3) | 40 (30.08 - 51.54) | 1624.87 (1485.85 - 1760.42) | 47.29 (37.58 - 58.32) | 0.5 (0.48 - 0.53) | 3633.88 (2966.21 - 4278.66) | 244.93 (184.78 - 316.72) | 10872.64 (9567.17 - 12179.93) | 315.88 (257.32 - 384.55) | 0.71 (0.67 - 0.75) | 270.88 (210.4 - 338.85) | 22.66 (16.58 - 30.48) | 695.84 (498.21 - 963.24) | 23.35 (16.08 - 33.96) | 0.01 (-0.05 - 0.06) | 5166.02 (4150.11 - 6250.53) | 371.75 (284.04 - 481.65) | 12641.26 (9588.74 - 16994.82) | 386.61 (279.88 - 535.34) | 0.03 (-0.02 - 0.08) |
| United States of America | 8.05 (7 - 9.15) | 70.33 (55.58 - 88.53) | 28.89 (25.52 - 32.1) | 83.29 (66.52 - 99.8) | 0.4 (0.34 - 0.47) | 50.01 (40.35 - 60.94) | 406.56 (298.44 - 536.94) | 180.92 (151.99 - 220.5) | 527.96 (410.52 - 667.76) | 0.63 (0.51 - 0.74) | 4.69 (3.72 - 5.84) | 46.24 (34.22 - 59.65) | 9.09 (7.36 - 11.29) | 28.32 (21.68 - 36.27) | -1.6 (-1.87 - -1.32) | 81.73 (65.5 - 101.24) | 722.08 (550.52 - 915.17) | 157.67 (128.99 - 192.75) | 465.39 (365.01 - 580.77) | -1.52 (-1.77 - -1.28) |
| United States Virgin Islands | 35022.03 (28632.18 - 41162.18) | 60.39 (40.4 - 83.24) | 83837.34 (76212.25 - 91990.87) | 79.05 (63.15 - 96.08) | 0.78 (0.71 - 0.85) | 274807.84 (222191.04 - 332095.4) | 473.49 (354.91 - 614.68) | 672137.94 (614895.69 - 732933.1) | 631.43 (547.04 - 720.22) | 0.82 (0.75 - 0.9) | 13079.79 (11508.79 - 13809.15) | 22.24 (19.51 - 23.56) | 37240.9 (31838.43 - 39924.87) | 34.16 (29.28 - 36.65) | 1.49 (1.37 - 1.62) | 224096.72 (204185.93 - 241491.71) | 382.57 (341.05 - 418.11) | 599982.09 (534343.27 - 644055.08) | 558.22 (494.31 - 603.8) | 1.26 (1.17 - 1.36) |
| Uruguay | 467.05 (423.82 - 512.64) | 66.89 (53.58 - 82.21) | 816.32 (735.91 - 914.83) | 81 (63.73 - 100.82) | 0.75 (0.68 - 0.82) | 3323.51 (2853.66 - 3730.43) | 480.44 (384.53 - 584.24) | 6751.32 (5771.81 - 8050.39) | 647.05 (515.25 - 814.78) | 1.17 (1.09 - 1.25) | 193.64 (181.62 - 202.5) | 28.49 (26.09 - 30.37) | 379.12 (338.7 - 402.7) | 33.01 (29.32 - 35.7) | 0.3 (0.2 - 0.39) | 3399.29 (3201.69 - 3580.65) | 489.79 (448.54 - 529.3) | 6070.35 (5549.58 - 6517.87) | 560.73 (502.85 - 615.95) | 0.29 (0.21 - 0.38) |
| Uzbekistan | 1066.05 (924.35 - 1211.82) | 59.88 (42.02 - 79.56) | 2649.97 (2367.73 - 2925.89) | 74.66 (59.76 - 92.37) | 0.76 (0.72 - 0.79) | 7896.3 (6274.49 - 9592.22) | 443.04 (326.34 - 582.28) | 17458.86 (14393.15 - 20429.15) | 506.31 (395.67 - 623.05) | 0.41 (0.31 - 0.5) | 262.98 (204.52 - 357.48) | 15.28 (11.77 - 20.89) | 528.14 (462.08 - 599.93) | 18.09 (15.53 - 20.6) | 0.72 (0.55 - 0.9) | 5134.98 (4137.33 - 6516.97) | 294.05 (230.35 - 382.4) | 11082.64 (9690.56 - 12528.33) | 344.64 (296.82 - 394.69) | 0.6 (0.46 - 0.73) |
| Vanuatu | 5.48 (4.67 - 6.41) | 70.16 (50.88 - 93.06) | 15.77 (13.53 - 18.26) | 69.53 (52.05 - 90.97) | 0.03 (-0.01 - 0.06) | 35.98 (29.22 - 44.43) | 471.62 (346.85 - 626.07) | 110.39 (90.54 - 134.74) | 493.22 (368.92 - 651.06) | 0.23 (0.16 - 0.3) | 2.2 (1.76 - 2.71) | 36.02 (28 - 46.04) | 5.9 (4.72 - 7.04) | 32.65 (25.02 - 40.96) | -0.37 (-0.4 - -0.34) | 46.13 (37.22 - 56.62) | 629.26 (495.06 - 791.29) | 121.8 (99.02 - 144.25) | 573.38 (451.85 - 705.28) | -0.35 (-0.38 - -0.32) |
| Venezuela (Bolivarian Republic of) | 619.7 (550.54 - 703.28) | 44.35 (33.14 - 57.4) | 2721.06 (2397.49 - 3087.19) | 56.58 (42.28 - 72.8) | 0.63 (0.54 - 0.72) | 4747.26 (3937.56 - 5620.54) | 329.68 (246.69 - 422.16) | 23055.34 (19784.5 - 26604.84) | 478.6 (373.28 - 596.05) | 0.95 (0.8 - 1.1) | 278.21 (257.47 - 291.77) | 21.25 (19.16 - 22.76) | 1100.18 (889.34 - 1349.14) | 25.15 (19.93 - 30.9) | 0.42 (0.18 - 0.65) | 4952.73 (4591.47 - 5277.78) | 358.25 (321.89 - 390.98) | 19713.59 (16338.25 - 23906.73) | 429.9 (347.47 - 526.29) | 0.4 (0.22 - 0.57) |
| Viet Nam | 3479.18 (3021.3 - 3951.88) | 55.74 (41.12 - 73.04) | 10945.25 (9554.05 - 12511.18) | 74.14 (54.36 - 96.55) | 0.97 (0.93 - 1) | 24047.66 (19347.53 - 29686.16) | 383.7 (279.2 - 506.22) | 82262.12 (69168.94 - 100259.01) | 560.06 (426.3 - 732.18) | 1.28 (1.23 - 1.34) | 1478.47 (1219.96 - 1831.78) | 26.47 (20.71 - 34.86) | 4135.22 (3446.2 - 5005.43) | 32.36 (25.19 - 41.78) | 0.74 (0.66 - 0.82) | 26441.41 (22246.29 - 32018.07) | 442.17 (354.08 - 565.92) | 74411.71 (63026.29 - 86852.11) | 542.47 (434.86 - 678.12) | 0.77 (0.7 - 0.84) |
| Yemen | 261.96 (226.37 - 302.61) | 40.99 (30.97 - 52.53) | 1027.99 (872.09 - 1187.46) | 54.27 (40.7 - 71.51) | 1.03 (0.97 - 1.1) | 1697.9 (1375.36 - 2027.13) | 264.44 (194.43 - 344.62) | 7417.36 (6091.48 - 9159.63) | 397.7 (300.4 - 524.08) | 1.46 (1.37 - 1.56) | 125.82 (94.35 - 175.01) | 24.41 (17.3 - 35.68) | 421.81 (302.42 - 588.51) | 27.32 (18.93 - 39.07) | 0.42 (0.37 - 0.47) | 2507.83 (1938.32 - 3379.38) | 415.36 (303.31 - 584.76) | 8068.93 (6082.52 - 10777.58) | 460.68 (333.19 - 634.15) | 0.38 (0.33 - 0.43) |
| Zambia | 169.72 (147.35 - 193.45) | 46.76 (35.57 - 59.25) | 416.85 (352.42 - 483.67) | 49.23 (37.11 - 63.54) | 0.19 (0.15 - 0.23) | 1028.51 (816.74 - 1246.1) | 275.74 (199.53 - 362.05) | 2743.94 (2211.48 - 3406.99) | 319.53 (231.91 - 425.17) | 0.52 (0.42 - 0.62) | 68.77 (53.76 - 86.92) | 22.49 (16.73 - 30.47) | 163.18 (122.55 - 222.62) | 23.45 (16.54 - 33.48) | -0.01 (-0.12 - 0.09) | 1312.67 (1061.73 - 1602.42) | 377.27 (291.4 - 490.63) | 3119.39 (2402.03 - 4044.32) | 392.38 (289.48 - 535.57) | -0.06 (-0.16 - 0.05) |
| Zimbabwe | 302.58 (260.41 - 348.68) | 57.68 (43.2 - 74.72) | 495.8 (420.34 - 572.83) | 61.88 (47.13 - 79.69) | 0.14 (0.11 - 0.16) | 1810.52 (1461.31 - 2228.81) | 343.46 (250.05 - 456.31) | 3029.82 (2447.56 - 3649.49) | 376.49 (274.78 - 492.89) | 0 (-0.08 - 0.08) | 124.67 (102.26 - 146.63) | 29.59 (23.24 - 36.72) | 214.1 (172.13 - 262.45) | 33.54 (25.99 - 42.77) | 0.74 (0.48 - 1) | 2289.03 (1883.59 - 2649.45) | 470.06 (377.48 - 570.55) | 4085.26 (3327.67 - 4977.71) | 542.93 (426.91 - 680.81) | 0.65 (0.42 - 0.89) |

**Abbreviations**: UI, uncertainty interval; ASR, age-standerised rate per 100,000; CI, confidence interval; DALYs, disability-adjusted life-year; EAPC, estimated annual percentage change.

**Table S9 Global burden of Alzheimer’s disease and other dementias among adults aged≥55 years from 1990 to 2021, categorized by countries**

| **Countries** | **Incidence** | | | | | **Prevalence** | | | | | **Deaths** | | | | | **DALYs** | | | | |
| --- | --- | --- | --- | --- | --- | --- | --- | --- | --- | --- | --- | --- | --- | --- | --- | --- | --- | --- | --- | --- |
|  | **No.(95% UI) in 1990** | **ASR per 100000 (95% UI) in 1990** | **No.(95% UI) in 2021** | **ASR per 100000 (95% UI) in 2021** | **EAPC**  **(95% CI)** | **No.(95% UI) in 1990** | **ASR per 100000 (95% UI) in 1990** | **No.(95% UI) in 2021** | **ASR per 100000 (95% UI) in 2021** | **EAPC**  **(95% CI)** | **No.(95% UI) in 1990** | **ASR per 100000 (95% UI) in 1990** | **No.(95% UI) in 2021** | **ASR per 100000 (95% UI) in 2021** | **EAPC**  **(95% CI)** | **No.(95% UI) in 1990** | **ASR per 100000 (95% UI) in 1990** | **No.(95% UI) in 2021** | **ASR per 100000 (95% UI) in 2021** | **EAPC**  **(95% CI)** |
| Afghanistan | 6371.87 (5475.03 - 7325.21) | 755.12 (513.13 - 1034.65) | 7666.31 (6605.7 - 8891.08) | 745.27 (505.89 - 1017.8) | -0.05 (-0.06 - -0.03) | 36806.17 (31311.53 - 42279.52) | 4468.88 (3492.02 - 5587.55) | 44239.42 (37657.05 - 51153.47) | 4379.2 (3427.55 - 5495.99) | -0.07 (-0.09 - -0.06) | 1252.16 (298.2 - 3368.81) | 208.05 (48.66 - 584.75) | 1591.07 (391.48 - 4209.36) | 195.98 (46.32 - 531.34) | -0.2 (-0.23 - -0.17) | 26690.35 (11688.07 - 61741.12) | 3518 (1442.23 - 8390.59) | 31790.17 (14116 - 69830.13) | 3361.84 (1417.66 - 7823.68) | -0.16 (-0.18 - -0.14) |
| Albania | 1725.01 (1494.29 - 1984.02) | 633.66 (429.34 - 881.44) | 4667.12 (4013.28 - 5369.39) | 636.79 (433.55 - 875.99) | 0.03 (0.02 - 0.04) | 10062.8 (8711.86 - 11524.23) | 3696.79 (2871.2 - 4661.78) | 27086.4 (23303.64 - 31383) | 3708.06 (2904.6 - 4643.82) | 0.03 (0.02 - 0.04) | 311.89 (76.76 - 839.22) | 127.39 (29.36 - 349.89) | 788.57 (187.51 - 2164.73) | 122.65 (27.46 - 347.47) | -0.11 (-0.14 - -0.09) | 6201.03 (2951.67 - 13799.19) | 2339.09 (1070.2 - 5203.78) | 15933.15 (7589 - 34488.05) | 2273.28 (1046.53 - 5085.9) | -0.08 (-0.1 - -0.06) |
| Algeria | 11373.56 (9651.78 - 13274.83) | 761.84 (519.99 - 1039.13) | 34839.59 (29974.42 - 40669.2) | 730.52 (497.06 - 1001.51) | -0.14 (-0.15 - -0.14) | 65471.97 (55259.43 - 76448.87) | 4518.3 (3542.35 - 5667.22) | 203737.43 (174694.42 - 236672.8) | 4319.51 (3380.53 - 5422.88) | -0.16 (-0.17 - -0.15) | 1481.69 (347.15 - 4120.3) | 165.17 (37.7 - 468.55) | 5543.47 (1366.2 - 14758.76) | 154.32 (35.33 - 423.68) | -0.17 (-0.2 - -0.14) | 37446.7 (17997.64 - 86032.21) | 2971.1 (1335.74 - 6872.59) | 120968.52 (57423.2 - 260284.82) | 2785.38 (1262.11 - 6228.13) | -0.2 (-0.22 - -0.18) |
| American Samoa | 14.72 (12.29 - 17.18) | 629.4 (424.83 - 874.69) | 37.81 (32.02 - 43.9) | 617.23 (417.63 - 854.32) | -0.08 (-0.09 - -0.07) | 85.66 (71.88 - 99.97) | 3665.44 (2818.87 - 4634.91) | 219.56 (183.6 - 253.91) | 3584.07 (2750.92 - 4530.74) | -0.09 (-0.1 - -0.08) | 2.38 (0.57 - 6.71) | 142.22 (32.64 - 394.47) | 6.49 (1.6 - 17.81) | 132.32 (30.22 - 365.45) | -0.17 (-0.22 - -0.12) | 54.33 (25.25 - 123.33) | 2521.46 (1093.76 - 5819.41) | 135.26 (63.35 - 295.47) | 2351.28 (1054.68 - 5265.9) | -0.19 (-0.22 - -0.15) |
| Andorra | 58.97 (50.21 - 69.06) | 696.15 (470.94 - 962.22) | 184.83 (158.68 - 212.46) | 654.77 (446.25 - 888.64) | -0.19 (-0.2 - -0.18) | 328.55 (278.01 - 380.29) | 3965.25 (3079.68 - 4985.75) | 1058.98 (897.74 - 1220.95) | 3713.15 (2880.49 - 4671.7) | -0.21 (-0.21 - -0.2) | 9.66 (2.33 - 24.57) | 150.67 (36.29 - 391.24) | 46.29 (12.02 - 121.29) | 142.03 (35.3 - 378.99) | -0.12 (-0.17 - -0.08) | 199.33 (96.42 - 411.72) | 2618.03 (1206.45 - 5578.12) | 731.12 (344.53 - 1554.55) | 2438.86 (1119.17 - 5327.75) | -0.19 (-0.22 - -0.15) |
| Angola | 2613.88 (2236.78 - 2990.78) | 722.58 (495.93 - 993.53) | 8162.62 (7029.28 - 9339.76) | 703.81 (480.01 - 966.14) | -0.08 (-0.09 - -0.07) | 15406.09 (13126.32 - 17629.12) | 4358.72 (3418.6 - 5459.76) | 48127.56 (41078.35 - 55163.16) | 4229.96 (3319.92 - 5288.79) | -0.1 (-0.1 - -0.09) | 396.53 (92.39 - 1092.54) | 166.19 (37.35 - 470.71) | 1543.28 (362.77 - 4363.84) | 196.85 (46.31 - 552.77) | 0.53 (0.48 - 0.58) | 9588.87 (4433.34 - 21536.2) | 2977.34 (1306.59 - 6915.29) | 34100.34 (14895.09 - 80332.88) | 3320.26 (1385.22 - 7909.64) | 0.34 (0.3 - 0.38) |
| Antigua and Barbuda | 55.64 (47.56 - 64.39) | 551.92 (375.87 - 762.47) | 83.23 (71.62 - 96.02) | 538.51 (365.06 - 746.78) | -0.07 (-0.08 - -0.06) | 322.48 (276.18 - 371.64) | 3198.31 (2490.42 - 4022.29) | 481.91 (410.39 - 554.08) | 3120.2 (2425.67 - 3941.52) | -0.07 (-0.08 - -0.06) | 9.82 (2.39 - 27.24) | 98.39 (22.84 - 278.28) | 12.25 (2.89 - 34.33) | 92.91 (21.52 - 265.15) | -0.07 (-0.11 - -0.02) | 188.84 (90.88 - 410.71) | 1892.36 (891.33 - 4236.7) | 267.42 (129.56 - 578.79) | 1807.25 (850.73 - 4065) | -0.09 (-0.11 - -0.06) |
| Argentina | 31490.46 (26869.42 - 36266.36) | 635.96 (435.18 - 875.32) | 60646.15 (51931.35 - 69806.57) | 605.88 (410.33 - 829.44) | -0.16 (-0.18 - -0.15) | 175336.15 (150036.06 - 200869.67) | 3565.71 (2781.65 - 4480.93) | 337722.08 (290790.33 - 388251.52) | 3384.63 (2633.26 - 4263.27) | -0.18 (-0.2 - -0.16) | 5189.87 (1285.08 - 14240.5) | 123.02 (29.28 - 339.99) | 11978.37 (3068.66 - 31466.55) | 119.07 (29.08 - 317.98) | -0.06 (-0.07 - -0.04) | 103698.64 (50604.11 - 221611.89) | 2219.98 (1028.34 - 4929.75) | 214388.03 (102025.6 - 449286.79) | 2144.77 (990.49 - 4669.21) | -0.09 (-0.1 - -0.07) |
| Armenia | 2409.14 (2065.39 - 2775.92) | 648.2 (443.8 - 886.51) | 4772.22 (4117.54 - 5494.09) | 645.61 (441.36 - 882.18) | -0.02 (-0.02 - -0.01) | 14003.54 (12048.01 - 16020.51) | 3748.21 (2927.38 - 4680.54) | 27712.96 (23756.23 - 31798.75) | 3727.12 (2916.14 - 4685.53) | -0.02 (-0.03 - -0.01) | 384.5 (95.07 - 1048.44) | 117.71 (27.45 - 327.59) | 900 (223.63 - 2379.73) | 125.79 (29.96 - 338.62) | 0.3 (0.24 - 0.36) | 7991.14 (3920.49 - 17219.65) | 2226.04 (1045.18 - 4913.39) | 17060.71 (8173.33 - 36076.55) | 2314.88 (1059.76 - 5081.47) | 0.18 (0.14 - 0.22) |
| Australia | 22609.53 (19696.92 - 25814.1) | 698.62 (491.97 - 936.05) | 50842.99 (44566.1 - 57221.02) | 581.82 (427.91 - 756.15) | -0.64 (-0.69 - -0.59) | 128416.44 (110845.1 - 146713.39) | 4021.23 (3182.78 - 4975.43) | 293946.33 (256048.53 - 331416.22) | 3366.94 (2720.89 - 4109.28) | -0.61 (-0.65 - -0.57) | 4086.43 (998.62 - 10935) | 142.65 (34.5 - 384.12) | 12818.87 (3355.4 - 32338.04) | 134.93 (33.52 - 350.36) | -0.16 (-0.18 - -0.14) | 78584.02 (38038.56 - 164698.91) | 2551.7 (1186.2 - 5570.19) | 208505.98 (96273.79 - 434230.86) | 2316.47 (1051.39 - 4975.05) | -0.32 (-0.33 - -0.31) |
| Austria | 15622.11 (13362.88 - 18380.92) | 708.31 (482.45 - 975.31) | 24830.32 (21296.6 - 28835.88) | 665.05 (450.87 - 910.33) | -0.24 (-0.27 - -0.22) | 88029.78 (74855.72 - 102059.89) | 4030.18 (3137.28 - 5082.49) | 141580.94 (120205.53 - 165214.87) | 3766.1 (2907.37 - 4756.26) | -0.26 (-0.29 - -0.23) | 2997.79 (741.84 - 8171.04) | 150.06 (35.56 - 411.81) | 6063.91 (1568.17 - 15166.75) | 144.12 (36.17 - 374.6) | -0.1 (-0.11 - -0.08) | 55065.22 (26116.78 - 119289.83) | 2615.7 (1196.95 - 5845.3) | 97320.34 (46381.3 - 201667.41) | 2483.6 (1154.48 - 5351.17) | -0.16 (-0.17 - -0.15) |
| Azerbaijan | 4385.51 (3781.03 - 5056.44) | 644.56 (438.25 - 882.68) | 7719.02 (6687.11 - 8818.63) | 624.06 (422.72 - 859.31) | -0.13 (-0.15 - -0.11) | 25385.26 (21883.89 - 29149.21) | 3720.02 (2911.29 - 4664.27) | 45063.72 (38895.26 - 52008.92) | 3597.72 (2810.46 - 4536.74) | -0.14 (-0.16 - -0.11) | 717.04 (176.43 - 2027.05) | 124.86 (29.14 - 345.97) | 1275.68 (318.83 - 3579.82) | 121 (28.32 - 343.4) | -0.04 (-0.08 - 0.01) | 14848.06 (7230.83 - 31324.3) | 2306.97 (1068.81 - 5116.33) | 26769.33 (12665.27 - 57873.09) | 2231.61 (1027.64 - 5034.04) | -0.09 (-0.11 - -0.07) |
| Bahamas | 119.59 (103.42 - 136.98) | 551.5 (374.53 - 762.76) | 305.94 (265.97 - 350.87) | 539.93 (365.89 - 745.37) | -0.07 (-0.08 - -0.07) | 689.38 (589.53 - 791) | 3191.74 (2482 - 4027.57) | 1769.95 (1517.17 - 2027.16) | 3122.11 (2427.37 - 3939.34) | -0.08 (-0.09 - -0.07) | 18.27 (4.38 - 50.45) | 95.32 (22.28 - 267.52) | 47.66 (11.36 - 131.23) | 92.81 (21.09 - 261.97) | -0.05 (-0.09 - -0.01) | 389.92 (188.44 - 847.02) | 1868 (888.42 - 4105.19) | 1004.32 (502.05 - 2145.06) | 1811.59 (856.42 - 3957.02) | -0.09 (-0.11 - -0.06) |
| Bahrain | 117.82 (99.87 - 137.01) | 759.95 (517.17 - 1038.55) | 586.38 (498.24 - 686.98) | 743.3 (504.64 - 1018.55) | -0.05 (-0.06 - -0.03) | 677.47 (574.22 - 785.13) | 4531.81 (3532.4 - 5671.1) | 3397.29 (2887.21 - 3940.48) | 4399.05 (3438.5 - 5525.82) | -0.07 (-0.09 - -0.05) | 14.84 (3.54 - 42.5) | 172.6 (40.46 - 490.33) | 69.24 (16.18 - 194.96) | 151.06 (34.51 - 429.34) | -0.48 (-0.56 - -0.41) | 386.34 (183.76 - 872.65) | 3030.42 (1348.22 - 6960.41) | 1806.42 (874.97 - 3910.42) | 2707.58 (1223.77 - 6257.04) | -0.4 (-0.44 - -0.35) |
| Bangladesh | 28451.57 (24297.82 - 32846.65) | 467.31 (318.83 - 644.71) | 86821.03 (74546.32 - 100566.74) | 449.85 (304.06 - 617.97) | -0.12 (-0.13 - -0.11) | 161807.18 (139202.9 - 185335.26) | 2639.2 (2065.12 - 3313.3) | 493238.57 (423064.4 - 569001.04) | 2538.24 (1976.69 - 3195.55) | -0.13 (-0.14 - -0.12) | 4722.84 (1113.07 - 13097.89) | 90.63 (20.63 - 258.52) | 16422.58 (3844.39 - 48817.92) | 101.64 (22.83 - 301.5) | 0.29 (0.17 - 0.41) | 99697.91 (45876.42 - 223867.09) | 1691.05 (752.61 - 3865.82) | 332669.89 (149346.22 - 782997.9) | 1802.29 (769.22 - 4325.91) | 0.16 (0.09 - 0.22) |
| Barbados | 311.51 (266.54 - 364.85) | 558.27 (378.43 - 771.07) | 481.04 (416.67 - 557.18) | 535.34 (363.51 - 737.56) | -0.14 (-0.16 - -0.13) | 1805.76 (1540.07 - 2099.09) | 3236.43 (2525.87 - 4060.68) | 2782.38 (2383.79 - 3191.34) | 3100.15 (2416.29 - 3907.56) | -0.14 (-0.16 - -0.13) | 46.44 (11.17 - 129.65) | 93.23 (21.21 - 268.26) | 81.46 (19.15 - 226.02) | 94.09 (21.19 - 261.35) | 0.08 (0 - 0.17) | 983.36 (481.55 - 2090.44) | 1849.35 (881.24 - 4102.4) | 1615.83 (778.95 - 3435.09) | 1819.87 (855.42 - 3983.97) | -0.02 (-0.07 - 0.04) |
| Belarus | 13774.77 (11809.74 - 15905.5) | 661.72 (451.58 - 913.34) | 18541.29 (15922.74 - 21326.57) | 660.09 (449.46 - 906.09) | 0 (-0.01 - 0.02) | 79991.67 (68243.83 - 92634.58) | 3844.64 (2992.55 - 4847.73) | 108054.42 (92642.51 - 124710.3) | 3832.77 (2990.98 - 4820.23) | 0 (-0.01 - 0.02) | 2324.06 (571.15 - 6475.22) | 123.9 (28.8 - 346.14) | 3406.29 (823.92 - 9360.61) | 121.21 (28.21 - 340.16) | -0.1 (-0.12 - -0.08) | 46467.86 (22408.38 - 98903.91) | 2313.98 (1082.64 - 5144.5) | 64652.28 (30691.23 - 138608.66) | 2287.86 (1066.18 - 5050.8) | -0.05 (-0.06 - -0.03) |
| Belgium | 22013.18 (19157.91 - 25255.76) | 783.54 (548.21 - 1063.34) | 33661.96 (29132.19 - 38681.06) | 695.67 (470 - 953.13) | -0.43 (-0.46 - -0.41) | 126015.88 (108721.8 - 144918.26) | 4533.32 (3592.88 - 5608.47) | 195870.5 (166719.75 - 226818.8) | 3990.59 (3087.11 - 5018.01) | -0.46 (-0.49 - -0.43) | 4411.83 (1133.9 - 11591.71) | 171.81 (42.08 - 454.59) | 8813.28 (2407.15 - 21549.25) | 156.15 (40.67 - 393.82) | -0.27 (-0.33 - -0.2) | 79728.38 (37998.31 - 169710.81) | 2960.57 (1361.08 - 6412.61) | 137877.28 (65720.95 - 283471.49) | 2666.22 (1229.34 - 5641.89) | -0.34 (-0.38 - -0.29) |
| Belize | 87.28 (75.82 - 99.97) | 572.58 (390.36 - 788.03) | 227.31 (195.62 - 259.69) | 550.35 (374.86 - 756.59) | -0.12 (-0.12 - -0.11) | 505.76 (436.12 - 578.78) | 3322.7 (2598.19 - 4168.76) | 1318.64 (1128.87 - 1500.7) | 3190.8 (2506.53 - 4002.01) | -0.12 (-0.13 - -0.11) | 15.09 (3.73 - 39.31) | 97.25 (22.92 - 263.8) | 37.96 (9.21 - 101.11) | 95.33 (22.43 - 258.44) | 0.02 (-0.05 - 0.09) | 293.38 (141.52 - 612.02) | 1916.23 (914.22 - 4128.94) | 767.19 (361.83 - 1614.47) | 1861.28 (884.99 - 4036.68) | -0.04 (-0.09 - 0) |
| Benin | 1282.29 (1107.74 - 1479.95) | 477.24 (319.48 - 660.24) | 2610.9 (2265.37 - 2999.94) | 425.74 (293.22 - 581.63) | -0.25 (-0.29 - -0.21) | 7410.61 (6319.8 - 8516.23) | 2728.18 (2119.38 - 3451.74) | 15276.76 (13348.43 - 17585.92) | 2432.05 (1922.64 - 3043.93) | -0.26 (-0.3 - -0.21) | 242.19 (56.63 - 641.59) | 105.86 (24.11 - 301.22) | 542.91 (127.26 - 1472.84) | 108.58 (24.63 - 307.21) | 0.09 (0.06 - 0.13) | 4832.04 (2209.51 - 10408.13) | 1871.51 (810.29 - 4365.07) | 10671.87 (4840.88 - 24057.87) | 1830.25 (765.43 - 4333.59) | -0.04 (-0.07 - -0.02) |
| Bermuda | 53.57 (45.47 - 61.81) | 565.46 (381.31 - 783.66) | 146.18 (126.03 - 168.51) | 556.43 (377.35 - 767.49) | -0.06 (-0.06 - -0.05) | 312.48 (266.21 - 360.24) | 3300.07 (2573.4 - 4167.64) | 852.07 (732.96 - 980.99) | 3244.42 (2533.4 - 4094.97) | -0.06 (-0.06 - -0.05) | 8.17 (1.97 - 22.79) | 100.22 (23.11 - 282.07) | 27.22 (6.81 - 69.77) | 96.87 (23.21 - 258.27) | -0.1 (-0.14 - -0.06) | 175.02 (85.33 - 372.07) | 1935.87 (912.36 - 4262.59) | 502.2 (247.71 - 1034.96) | 1873.28 (895.49 - 3950.94) | -0.12 (-0.14 - -0.09) |
| Bhutan | 116.57 (99.67 - 134.31) | 474.79 (320.55 - 659.57) | 391 (335.52 - 450.48) | 438.06 (295.9 - 605.72) | -0.25 (-0.28 - -0.23) | 675.1 (572.08 - 770.48) | 2698.78 (2096.5 - 3411.5) | 2221 (1899.62 - 2561.52) | 2477.06 (1925.77 - 3131.79) | -0.27 (-0.29 - -0.25) | 17.61 (4.05 - 47.67) | 91.33 (19.07 - 255.94) | 94.33 (22.84 - 265.85) | 115.58 (26.22 - 328.37) | 0.85 (0.79 - 0.91) | 412.56 (190.87 - 896.61) | 1727.17 (764.8 - 3876.82) | 1704.08 (740.12 - 3932.44) | 1956.53 (794.43 - 4699) | 0.47 (0.44 - 0.5) |
| Bolivia (Plurinational State of) | 1859.05 (1586.04 - 2146.34) | 469.85 (319.57 - 648.83) | 5633.47 (4820.2 - 6503.62) | 462.55 (313.11 - 640.22) | -0.01 (-0.03 - 0.01) | 10506.25 (8976.58 - 12076.47) | 2640.39 (2051.81 - 3323.62) | 31776.87 (27192.29 - 36646.09) | 2595.53 (2019.9 - 3272.92) | -0.02 (-0.04 - 0.01) | 285.88 (67.06 - 760.5) | 91.12 (20.53 - 261.46) | 924.64 (218 - 2520.44) | 92.07 (20.99 - 253.04) | 0.05 (0.02 - 0.08) | 6384.26 (3002.7 - 13795.34) | 1699.47 (759.67 - 3934.26) | 19807.39 (9204.4 - 43260.36) | 1701.18 (765.72 - 3811.33) | 0.02 (0 - 0.04) |
| Bosnia and Herzegovina | 3284.04 (2780.78 - 3818.39) | 632.3 (430.11 - 872.58) | 6927.23 (5949.29 - 8078.12) | 632.96 (430.54 - 871.25) | 0.02 (-0.02 - 0.05) | 19087.84 (16223.18 - 22054.14) | 3664.19 (2845.75 - 4622.91) | 40153.79 (34099.12 - 46488.23) | 3667.4 (2854.05 - 4596.84) | 0.02 (-0.01 - 0.05) | 501.09 (123.6 - 1346.85) | 120.05 (28.18 - 331.35) | 1198.16 (293.7 - 3124.22) | 115.27 (26.58 - 307.09) | -0.13 (-0.16 - -0.1) | 10972.32 (5292.83 - 24106.53) | 2238.99 (1039.33 - 4976.64) | 23441.49 (11341.98 - 48735.5) | 2172.79 (1030.9 - 4669.26) | -0.09 (-0.11 - -0.08) |
| Botswana | 358.14 (306.6 - 414.31) | 612.16 (415.94 - 842.03) | 963.83 (831.01 - 1114.37) | 587.78 (398.54 - 804.72) | -0.11 (-0.13 - -0.08) | 2090.83 (1769.25 - 2413.29) | 3561.7 (2785.84 - 4462.01) | 5627.05 (4798.15 - 6467.33) | 3413.56 (2658.83 - 4281.76) | -0.11 (-0.13 - -0.08) | 53.82 (12.2 - 154.54) | 135.53 (29.42 - 399.51) | 170.19 (39.98 - 462.73) | 133.03 (30.85 - 366.45) | 0 (-0.06 - 0.07) | 1303.49 (594.21 - 2996.04) | 2435.59 (1054.98 - 5807.72) | 3640.65 (1670.16 - 8227.05) | 2360.64 (1037.24 - 5422.42) | -0.06 (-0.11 - -0.01) |
| Brazil | 83793.99 (72734 - 95547.59) | 728.94 (502.6 - 989.84) | 289930.53 (254013.18 - 330362.3) | 716.79 (491.5 - 982.98) | -0.11 (-0.15 - -0.07) | 497388.78 (429247.22 - 570238.34) | 4335.42 (3410.59 - 5421.01) | 1757235.62 (1522599.05 - 2009037.73) | 4349.63 (3418.34 - 5451.67) | -0.05 (-0.1 - 0) | 15334.97 (3806.42 - 40385.08) | 165.9 (40.9 - 445.71) | 63590.81 (16725.44 - 160905.32) | 160.76 (40.95 - 417.89) | -0.07 (-0.09 - -0.06) | 322570.6 (150014.02 - 719435.82) | 2973.66 (1322.49 - 6699.8) | 1177308.12 (549332.93 - 2516146.79) | 2926.54 (1328.4 - 6413.75) | -0.06 (-0.07 - -0.05) |
| Brunei Darussalam | 71.23 (60.23 - 83.64) | 577.32 (392.33 - 801.58) | 214.92 (183.55 - 250.09) | 576.24 (391.94 - 792.25) | 0.03 (-0.01 - 0.07) | 406.68 (343.51 - 472.25) | 3325.01 (2569.14 - 4196.65) | 1231.46 (1051.24 - 1424.58) | 3313.79 (2568.94 - 4172.35) | 0.02 (-0.02 - 0.06) | 12.29 (3.01 - 34.87) | 132.22 (30.84 - 369.51) | 33.17 (7.92 - 89.57) | 137.1 (32.57 - 368.79) | 0.23 (0.14 - 0.32) | 248.7 (117.78 - 548.03) | 2243.43 (991.6 - 5178.92) | 744.36 (350.56 - 1610.42) | 2305.92 (1018.93 - 5105.55) | 0.18 (0.11 - 0.25) |
| Bulgaria | 11225.03 (9340.64 - 13399.5) | 651.68 (439.73 - 900.43) | 16840.28 (14386.23 - 19764.66) | 637.2 (432.59 - 873.18) | -0.08 (-0.11 - -0.06) | 64120.31 (53408.36 - 75138.45) | 3767.34 (2918.72 - 4751.1) | 96808.54 (82528.34 - 112490.19) | 3684.75 (2868.45 - 4631.65) | -0.08 (-0.11 - -0.06) | 1336.2 (310.65 - 3808.6) | 117.59 (26.35 - 338.24) | 2780.8 (704.6 - 7789.99) | 118.03 (27.36 - 335.16) | -0.01 (-0.03 - 0.02) | 33807.33 (16744.33 - 75464.52) | 2228.76 (1033.08 - 5033.04) | 55753.47 (26954.99 - 117936.6) | 2212.12 (1040.31 - 4919.69) | -0.04 (-0.07 - -0.02) |
| Burkina Faso | 2357.83 (2015.71 - 2724.94) | 481.02 (323.89 - 660.82) | 4914.83 (4209.98 - 5666.85) | 449.77 (301.9 - 623.49) | -0.23 (-0.24 - -0.23) | 13754.62 (11671.31 - 15827.88) | 2736.67 (2139.37 - 3435.04) | 28438.18 (24271.94 - 32689.9) | 2549.65 (1975.16 - 3220.56) | -0.25 (-0.25 - -0.24) | 454.95 (111 - 1272.51) | 130.41 (29.87 - 364.36) | 1008.62 (236.96 - 2838.42) | 119.41 (27.22 - 334.44) | -0.35 (-0.42 - -0.29) | 9791.08 (4394.7 - 22236.06) | 2173.4 (890.48 - 5143.19) | 20181.48 (8828.47 - 46556.95) | 1983.92 (818.48 - 4709.21) | -0.34 (-0.39 - -0.29) |
| Burundi | 1716.68 (1466.04 - 1988.66) | 616.97 (421.86 - 848.56) | 2903.35 (2513.31 - 3331.39) | 567.12 (384.75 - 783.02) | -0.24 (-0.26 - -0.22) | 10068.07 (8570.54 - 11617) | 3618.66 (2833.15 - 4543.42) | 17152.83 (14635.45 - 19611.26) | 3315.83 (2586.33 - 4166.32) | -0.25 (-0.27 - -0.23) | 264.56 (61.82 - 734.13) | 132.89 (29.81 - 371.64) | 555.96 (130.78 - 1564.36) | 147.59 (33.62 - 419.77) | 0.38 (0.35 - 0.41) | 6071.22 (2840.05 - 13660.42) | 2398.7 (1074.59 - 5478.16) | 11814.36 (5231.32 - 27793.52) | 2509.35 (1043.36 - 6020.04) | 0.18 (0.16 - 0.21) |
| Cabo Verde | 191.34 (163.35 - 222.78) | 464.2 (313.47 - 643.5) | 300.09 (257.31 - 341.07) | 440.53 (298.44 - 609.46) | -0.17 (-0.18 - -0.17) | 1089.73 (933.66 - 1251.79) | 2651.95 (2079 - 3328.69) | 1713.92 (1463.65 - 1949.34) | 2510.73 (1954.67 - 3166.18) | -0.18 (-0.19 - -0.17) | 37.84 (9.19 - 99.27) | 97.31 (22.27 - 269.12) | 72.41 (17.87 - 191.23) | 103.28 (23.77 - 282.79) | 0.15 (0.11 - 0.19) | 707.49 (331.87 - 1529.96) | 1759.64 (789.16 - 3962.74) | 1238.71 (547.53 - 2788.98) | 1790.88 (783.98 - 4087.57) | 0.02 (0 - 0.05) |
| Cambodia | 3259.58 (2764.16 - 3766.88) | 653.59 (443.37 - 902.24) | 9350.62 (8041.16 - 10819.66) | 631.69 (427.75 - 872.43) | -0.16 (-0.18 - -0.14) | 19571.85 (16579.59 - 22442.26) | 3903.38 (3041.56 - 4905.61) | 56031.92 (47609.76 - 64619.38) | 3762.02 (2921.36 - 4734.94) | -0.17 (-0.19 - -0.15) | 457.44 (108.46 - 1299.89) | 124.5 (28.24 - 350.91) | 1670.82 (392 - 4837.26) | 151.53 (34.64 - 428.25) | 0.74 (0.7 - 0.79) | 11190.29 (5369.82 - 23817.19) | 2389 (1085.13 - 5397.12) | 37055.68 (17069.94 - 87209.51) | 2686.57 (1157.68 - 6285.79) | 0.44 (0.41 - 0.46) |
| Cameroon | 2231.7 (1906.57 - 2575.56) | 449.44 (305.86 - 621.34) | 5752.73 (4924.83 - 6622.95) | 425.33 (286.39 - 588.33) | -0.18 (-0.18 - -0.17) | 12907.23 (10970.61 - 14779.85) | 2538.13 (1976.33 - 3195.46) | 33308.59 (28337.85 - 38295.82) | 2399.08 (1859.56 - 3031.71) | -0.18 (-0.19 - -0.17) | 460.08 (112.07 - 1244.14) | 120.47 (27.5 - 336.99) | 1159.41 (268.62 - 3351.35) | 113.34 (25.01 - 336.1) | -0.24 (-0.3 - -0.17) | 9391.81 (4123.55 - 20919.71) | 2007.7 (825.41 - 4717.58) | 23907.08 (10730.63 - 56464.44) | 1887.94 (775.83 - 4669.47) | -0.23 (-0.28 - -0.18) |
| Canada | 46584.21 (41466.8 - 52011.75) | 842.64 (614.34 - 1094.24) | 102049.58 (90591.74 - 113861.02) | 741.06 (538.16 - 967.24) | -0.56 (-0.62 - -0.5) | 275752.81 (241749.26 - 310254.41) | 5035.46 (4114.11 - 6023.07) | 614963.95 (539354.83 - 690918.67) | 4478.65 (3643.58 - 5369.27) | -0.56 (-0.65 - -0.47) | 6654.73 (1695.48 - 17491.71) | 129.74 (31.73 - 348.85) | 18607.24 (4946.06 - 47117.41) | 124.55 (31.24 - 324.68) | -0.16 (-0.18 - -0.13) | 139542.33 (71764.37 - 279437.61) | 2605.4 (1322.62 - 5364.85) | 340830.56 (169894.89 - 676512.65) | 2407.33 (1197.73 - 4921.37) | -0.35 (-0.4 - -0.31) |
| Central African Republic | 776.62 (659.68 - 893.53) | 754.11 (518.25 - 1030.47) | 1425.06 (1207.75 - 1627.69) | 743.87 (508.75 - 1008.94) | -0.09 (-0.11 - -0.08) | 4571.92 (3886.19 - 5239.13) | 4589.48 (3597.17 - 5714.45) | 8403.94 (7132 - 9642.89) | 4567.16 (3610.88 - 5674.98) | -0.1 (-0.13 - -0.07) | 124.63 (29.73 - 334.04) | 193.66 (44.8 - 520) | 218.13 (48.83 - 593.39) | 188.49 (41.48 - 512) | -0.08 (-0.13 - -0.04) | 3013.39 (1365.31 - 6677.05) | 3353.7 (1435.44 - 7627.4) | 5401.97 (2390.38 - 11898.87) | 3304.03 (1385.06 - 7491.26) | -0.08 (-0.11 - -0.05) |
| Chad | 1748.75 (1502.92 - 2027.75) | 474.09 (321.49 - 658.3) | 2730.75 (2321.8 - 3138.83) | 430.37 (289.4 - 596.48) | -0.32 (-0.33 - -0.32) | 10089.21 (8593.81 - 11563.97) | 2694.7 (2094.05 - 3391.88) | 15848.32 (13409.59 - 18144.81) | 2434.9 (1892.86 - 3065.35) | -0.34 (-0.34 - -0.33) | 297.01 (70.18 - 820.34) | 101.24 (22.03 - 286.35) | 510.74 (114.67 - 1423.4) | 104.51 (23 - 293.71) | 0.14 (0.12 - 0.16) | 6308.22 (2947.54 - 13685.94) | 1808.41 (793.58 - 4197.17) | 10854.97 (4862.3 - 25130.77) | 1802.31 (752.2 - 4236.11) | 0.01 (0 - 0.02) |
| Chile | 9064.33 (7881.37 - 10412.1) | 621.46 (432.16 - 839.91) | 27468.44 (23764.17 - 31582.88) | 612.08 (417.17 - 837.53) | -0.02 (-0.04 - 0.01) | 50626.21 (44131 - 58117.61) | 3481.8 (2753.52 - 4329.21) | 154238.82 (132421.2 - 176770.08) | 3432.65 (2681.16 - 4309.7) | -0.01 (-0.04 - 0.02) | 1434.86 (345.56 - 3901.8) | 114.84 (27.53 - 313.36) | 5178.16 (1317.37 - 13354.18) | 114.37 (28.03 - 302.42) | -0.02 (-0.04 - 0) | 29458.73 (14333.65 - 62829.64) | 2123.64 (990.68 - 4705.76) | 94525.88 (45775.59 - 196969.83) | 2099.84 (984.77 - 4500.8) | -0.04 (-0.05 - -0.03) |
| China | 649232.02 (553982.17 - 754733.92) | 683.82 (463.64 - 937.09) | 2785360.25 (2388028.57 - 3206095.04) | 861.46 (591.65 - 1178.24) | 0.42 (0.34 - 0.5) | 3784990.03 (3237847.89 - 4367691.96) | 4013.32 (3121.15 - 5066.39) | 16417647.94 (13893114.62 - 19080449.26) | 5180.9 (4033.4 - 6513.45) | 0.45 (0.36 - 0.54) | 118612.58 (28106.81 - 318891.59) | 184.96 (43.38 - 502.99) | 489054.9 (124164.04 - 1319945.92) | 181.67 (44.29 - 492.83) | -0.19 (-0.23 - -0.15) | 2611521.97 (1183441.05 - 5891609.86) | 3106.33 (1327.65 - 7095.06) | 9860689.08 (4826146.29 - 21802628.43) | 3272.96 (1505.09 - 7181.81) | -0.02 (-0.06 - 0.02) |
| Colombia | 15072.81 (13035.93 - 17249.04) | 645.11 (438.86 - 888.07) | 61148.25 (53253.88 - 69589.5) | 635.77 (435.82 - 874.8) | -0.04 (-0.06 - -0.02) | 86709.09 (74818.67 - 98801.3) | 3709.54 (2907.18 - 4632.75) | 351755.34 (303024.64 - 400320.04) | 3658.34 (2863.45 - 4603.13) | -0.04 (-0.06 - -0.01) | 2210.9 (550.92 - 6034.05) | 105.84 (25.28 - 294.03) | 10990.57 (2799.54 - 27164.54) | 105.11 (25.68 - 278.48) | -0.04 (-0.05 - -0.02) | 47471.24 (23402.7 - 101738.01) | 2082.45 (1001.83 - 4554.35) | 205361.14 (100631.25 - 415980.77) | 2080.54 (1015.45 - 4402.4) | -0.01 (-0.02 - 0) |
| Comoros | 125.45 (106.65 - 144.48) | 594.75 (403.66 - 818.91) | 349.59 (301.24 - 401.76) | 569.88 (385.8 - 780.23) | -0.12 (-0.13 - -0.11) | 741.34 (629.38 - 855.23) | 3495.24 (2733.5 - 4388.86) | 2049.87 (1747.73 - 2348.38) | 3322.52 (2599.58 - 4150.54) | -0.15 (-0.16 - -0.14) | 21.55 (5.11 - 57.02) | 147.33 (33.79 - 393.87) | 81.98 (20.14 - 225.02) | 165.92 (38.9 - 458.08) | 0.42 (0.39 - 0.45) | 486.19 (225.7 - 1055.38) | 2537.61 (1099.12 - 5744.07) | 1580.58 (687.32 - 3687.09) | 2747.67 (1122.89 - 6576.9) | 0.29 (0.27 - 0.31) |
| Congo | 787.53 (672.26 - 907.73) | 708.72 (484.69 - 971.47) | 1838.18 (1646.63 - 2024.86) | 684.14 (506.06 - 882.28) | -0.09 (-0.11 - -0.07) | 4597.5 (3884.78 - 5298.02) | 4256.47 (3320.81 - 5335.72) | 10749.91 (9658.14 - 11829.19) | 4038.55 (3375.66 - 4788.97) | -0.14 (-0.16 - -0.13) | 133.01 (32.08 - 351.12) | 205.73 (47.94 - 558.43) | 372.18 (88.79 - 997.55) | 203.23 (46.61 - 545.79) | -0.03 (-0.06 - 0) | 3159.07 (1406.45 - 7013.7) | 3408.45 (1417.04 - 7904.66) | 7903.94 (3482.25 - 17666.73) | 3328.05 (1356.98 - 7683.15) | -0.06 (-0.08 - -0.04) |
| Cook Islands | 9.97 (8.41 - 11.68) | 630.68 (426.06 - 875.31) | 26.93 (22.71 - 31.21) | 619.17 (417.58 - 855.93) | -0.07 (-0.08 - -0.06) | 58.13 (48.98 - 67.8) | 3685.48 (2828.79 - 4646.2) | 156.8 (132.09 - 181.55) | 3611.64 (2786.56 - 4561.47) | -0.08 (-0.1 - -0.07) | 1.83 (0.44 - 5.15) | 148.56 (34.65 - 415.87) | 5.11 (1.31 - 13.59) | 128.97 (30.45 - 348.78) | -0.42 (-0.45 - -0.38) | 38.01 (17.46 - 86.45) | 2588.05 (1122.56 - 5975.55) | 96.94 (45.4 - 209.1) | 2304.05 (1043.38 - 5085.33) | -0.34 (-0.37 - -0.32) |
| Costa Rica | 1719.28 (1483.85 - 1973.65) | 643.76 (435.04 - 883.55) | 5897.53 (5127.69 - 6742.49) | 626.98 (422.95 - 866.9) | -0.08 (-0.09 - -0.06) | 9891.54 (8493.15 - 11364.53) | 3704.4 (2898.49 - 4647.6) | 33986.74 (29563.44 - 38847.18) | 3612.94 (2815.9 - 4553.58) | -0.07 (-0.09 - -0.05) | 276.21 (68.84 - 751.02) | 108.56 (25.77 - 297.96) | 1074.21 (276.97 - 2742.46) | 106.2 (26 - 280.18) | -0.06 (-0.08 - -0.04) | 5574.85 (2732.19 - 11757.13) | 2115.25 (1005.47 - 4558.63) | 19951.8 (9752.63 - 40553.16) | 2069 (997.94 - 4356.42) | -0.07 (-0.08 - -0.05) |
| Côte d'Ivoire | 5799.14 (4987.65 - 6730.71) | 660 (447.3 - 907.36) | 11029.6 (9446.51 - 12738.85) | 637.73 (432.37 - 879.92) | -0.09 (-0.1 - -0.07) | 33885.32 (28646.17 - 39253.41) | 3842.23 (2995.52 - 4848.18) | 64002.59 (54510.81 - 74211.69) | 3703.23 (2869.89 - 4677.33) | -0.09 (-0.11 - -0.08) | 917.92 (223.52 - 2581.08) | 122.98 (28.41 - 346.38) | 2045.32 (491.77 - 5468.24) | 119.82 (27.86 - 330.7) | -0.09 (-0.12 - -0.07) | 19393.17 (9409.5 - 42769.66) | 2304.8 (1071.99 - 5125.57) | 38077.12 (18145.76 - 81872.8) | 2221.97 (1032.19 - 4898.15) | -0.11 (-0.13 - -0.09) |
| Croatia | 8636.59 (7424.33 - 9937.41) | 522.13 (360.47 - 713.15) | 18609.74 (16219.98 - 21159.95) | 518.05 (359.04 - 703.1) | -0.2 (-0.25 - -0.16) | 49337.5 (42399.07 - 56672.96) | 2977.32 (2355.24 - 3708.76) | 107870.41 (93399.34 - 122652.89) | 3009.35 (2364.93 - 3743.45) | -0.19 (-0.24 - -0.13) | 1316.77 (318.53 - 3698.44) | 93 (21.69 - 261.5) | 3480.99 (856.48 - 9572.7) | 90.66 (21.05 - 257.88) | -0.11 (-0.13 - -0.1) | 27882.72 (13826.15 - 60342.21) | 1773.81 (832.58 - 3981.14) | 64215.67 (30794.3 - 138109.92) | 1754.96 (829.78 - 3898.53) | -0.14 (-0.16 - -0.11) |
| Cuba | 799.97 (670.56 - 952.47) | 698.96 (474.42 - 960.6) | 2395.37 (2041.16 - 2813.43) | 672.18 (450.76 - 926.25) | -0.09 (-0.11 - -0.07) | 4340.72 (3595.04 - 5146.46) | 3971.26 (3075.51 - 5002.55) | 13386.72 (11287.91 - 15587.63) | 3806.87 (2948.52 - 4801.97) | -0.09 (-0.11 - -0.07) | 122.25 (29.03 - 345.23) | 170.6 (38.53 - 484.54) | 438.52 (108.55 - 1211.78) | 152.25 (35.52 - 424.82) | -0.37 (-0.44 - -0.29) | 2736.26 (1275.85 - 6405.55) | 2882.5 (1236.72 - 6835.11) | 8361.74 (3961.98 - 18096.51) | 2573.63 (1144.1 - 5823.94) | -0.37 (-0.41 - -0.32) |
| Cyprus | 14507.86 (12345.8 - 17004.78) | 633.62 (430.25 - 875.14) | 25607.43 (22090.3 - 29552.22) | 629.77 (426.24 - 865.61) | -0.05 (-0.07 - -0.04) | 83874.76 (71235.85 - 97245.65) | 3682.43 (2865.39 - 4636.46) | 147496.82 (126373.09 - 169557.11) | 3651.05 (2844.38 - 4594.1) | -0.06 (-0.07 - -0.05) | 2378.31 (569.75 - 6683.39) | 121.13 (28.36 - 341.24) | 4886.33 (1206.56 - 12845.87) | 119.98 (28.86 - 326.17) | 0.02 (0 - 0.04) | 48435.67 (23528.36 - 108142.58) | 2234.77 (1029.53 - 5055.27) | 89763.99 (43405.18 - 186561.33) | 2221.65 (1032.33 - 4815.96) | -0.01 (-0.02 - 0.01) |
| Czechia | 1637.81 (1396.01 - 1896.31) | 448.66 (302.06 - 620.84) | 4937.48 (4193.02 - 5700.21) | 432.09 (289.71 - 597.97) | -0.13 (-0.15 - -0.11) | 9602.16 (8160.3 - 10998.76) | 2542.57 (1976.71 - 3215.19) | 28686.62 (24350.39 - 32844.7) | 2441.55 (1890.81 - 3086.31) | -0.13 (-0.15 - -0.11) | 287.67 (70.27 - 772.01) | 114.27 (26.43 - 313.94) | 971.33 (217.21 - 2713.37) | 112.47 (24.76 - 322.51) | -0.04 (-0.08 - -0.01) | 6378.41 (2849.28 - 14170.47) | 1900.69 (796.73 - 4445.55) | 20089.01 (8969.17 - 46291.26) | 1876.14 (780.14 - 4500.52) | -0.04 (-0.06 - -0.01) |
| Democratic People's Republic of Korea | 11399.69 (9745.11 - 13257.73) | 630.47 (425.68 - 868.69) | 29765.02 (25211.58 - 34951.4) | 616.99 (417.36 - 851.38) | -0.09 (-0.11 - -0.06) | 65272.2 (55107.77 - 75333.47) | 3628.77 (2790.71 - 4576.61) | 169741.86 (143956.33 - 196504.46) | 3554.54 (2759.31 - 4479.77) | -0.08 (-0.11 - -0.06) | 1947.06 (448.45 - 5387.69) | 137.32 (29.73 - 386.06) | 5785.23 (1333.91 - 16301.85) | 142.56 (32.13 - 395.26) | 0.21 (0.17 - 0.26) | 41494.33 (19436.31 - 93086.89) | 2456.45 (1082.71 - 5594.6) | 113751.47 (52328.23 - 252889.38) | 2504.71 (1090.8 - 5711.02) | 0.12 (0.09 - 0.15) |
| Democratic Republic of the Congo | 10967.42 (9364.72 - 12729.55) | 715.7 (491.6 - 983.53) | 26848.46 (23273.62 - 30644.36) | 720.35 (492.97 - 982.99) | 0.03 (-0.01 - 0.06) | 64111.9 (54675.66 - 73821.39) | 4274.06 (3353.48 - 5353.7) | 158820.04 (137370.88 - 181239.16) | 4331.11 (3411.87 - 5406.11) | 0.06 (0.02 - 0.1) | 1678 (400.74 - 4593.49) | 177.57 (40.43 - 497.09) | 5599.2 (1335.67 - 15273.8) | 208.87 (48.04 - 571.53) | 0.62 (0.55 - 0.69) | 41363.72 (18246.33 - 91792.01) | 3088.84 (1307.18 - 7212.6) | 117701.65 (50680.57 - 276166.41) | 3494.96 (1435.84 - 8199.47) | 0.46 (0.42 - 0.51) |
| Denmark | 9068.46 (7909.5 - 10413.84) | 587.09 (411.99 - 790.54) | 11222.04 (9695.28 - 12915.65) | 479.58 (329.11 - 655.34) | -0.74 (-0.78 - -0.7) | 51690.53 (44792.67 - 59397.66) | 3366.55 (2672.42 - 4179.71) | 63232.47 (54648.5 - 72245.88) | 2712.14 (2123.22 - 3387.58) | -0.79 (-0.84 - -0.75) | 2043.39 (514.55 - 5556.22) | 136.89 (32.59 - 378.4) | 3505.15 (902.55 - 8806.69) | 142.26 (34.85 - 372.88) | 0.1 (0.01 - 0.19) | 35484.15 (16344.31 - 77244.66) | 2345.22 (1037.89 - 5356.05) | 53489.21 (23192.58 - 116348.15) | 2245.65 (945.32 - 5083.41) | -0.16 (-0.22 - -0.1) |
| Djibouti | 73.58 (62.52 - 84.68) | 619.35 (422.17 - 847.65) | 348.07 (296.09 - 401) | 587.14 (401.19 - 808.56) | -0.13 (-0.15 - -0.11) | 436.79 (370.11 - 499.01) | 3649.31 (2857.79 - 4561.22) | 2067.1 (1760.8 - 2366.17) | 3443.69 (2696.46 - 4334.91) | -0.15 (-0.16 - -0.13) | 12.19 (2.83 - 33.05) | 154.03 (35.04 - 435) | 66.73 (15.3 - 182.29) | 169.46 (38.03 - 463.76) | 0.3 (0.27 - 0.34) | 284.83 (130.24 - 643.66) | 2657.58 (1128.89 - 6282.02) | 1488.28 (659.33 - 3394.94) | 2801.14 (1152.55 - 6501.55) | 0.18 (0.15 - 0.2) |
| Dominica | 56.1 (48.28 - 64.73) | 564.13 (383.39 - 778.77) | 70.45 (60.88 - 80.69) | 547.32 (368.72 - 752.68) | -0.09 (-0.1 - -0.08) | 322.5 (276.41 - 372.31) | 3257.42 (2547.16 - 4105.49) | 406.95 (348.67 - 465.19) | 3160.05 (2457.61 - 3975.24) | -0.09 (-0.1 - -0.08) | 8.43 (2.05 - 23.32) | 98.11 (22.51 - 283.02) | 11.35 (2.77 - 30.25) | 96.93 (22.67 - 275.01) | -0.01 (-0.04 - 0.02) | 180.22 (87.41 - 391.98) | 1910.61 (894.48 - 4242.6) | 233.68 (110.59 - 483.42) | 1861.51 (876.12 - 4127.56) | -0.06 (-0.08 - -0.05) |
| Dominican Republic | 2802.43 (2400.6 - 3238.94) | 561.73 (386.59 - 765.83) | 9036.64 (7849.24 - 10273.94) | 565.25 (391.67 - 767.99) | -0.11 (-0.15 - -0.08) | 16190.09 (13999.15 - 18495.61) | 3239.78 (2551.46 - 4042.7) | 52487.46 (45343.01 - 60032.46) | 3282.86 (2580.24 - 4083.35) | -0.11 (-0.15 - -0.07) | 407.16 (96.49 - 1133.99) | 100.72 (22.87 - 280.33) | 1517.65 (355.32 - 4002.99) | 96.49 (21.97 - 265.07) | -0.03 (-0.08 - 0.02) | 9057.71 (4482.18 - 19090.13) | 1926.88 (898.99 - 4215.68) | 29990.51 (14444.75 - 60888.91) | 1883.79 (893.64 - 4047.47) | -0.07 (-0.1 - -0.04) |
| Ecuador | 3524.51 (3010.01 - 4084.64) | 468.52 (318.42 - 647.29) | 11846.32 (10073.59 - 13754.26) | 457.3 (307.91 - 633.65) | -0.03 (-0.05 - -0.02) | 20051.81 (17320.51 - 23050.75) | 2653.09 (2068.44 - 3352.67) | 67118.68 (57049.13 - 77028.79) | 2587.7 (2004.81 - 3266.2) | -0.04 (-0.06 - -0.02) | 565.5 (132.67 - 1543.55) | 85.49 (19.59 - 242.13) | 1938.4 (440.12 - 5394.34) | 82.1 (17.9 - 231.96) | -0.11 (-0.13 - -0.09) | 11785.75 (5653.79 - 25731.94) | 1623.26 (746.84 - 3671.31) | 39409.11 (18946.61 - 84650.64) | 1565.85 (720.43 - 3475.78) | -0.09 (-0.11 - -0.08) |
| Egypt | 20749.53 (18147.06 - 23457.7) | 744.47 (522.56 - 1006.61) | 46780.07 (40459.2 - 53391.04) | 720.9 (496.67 - 975.34) | -0.04 (-0.06 - -0.01) | 118090.56 (102415.03 - 132648.92) | 4335.97 (3469.77 - 5335.56) | 265068.38 (227133.21 - 300259.18) | 4179.96 (3287.6 - 5197.44) | -0.06 (-0.08 - -0.03) | 3037.09 (719.11 - 8537.36) | 159.64 (36.29 - 455) | 6417.76 (1533.93 - 17472.1) | 143.66 (33.31 - 401.36) | -0.38 (-0.4 - -0.35) | 70975.99 (33892.52 - 162416.1) | 2861.29 (1274.93 - 6645.92) | 153359.99 (73217.97 - 341460.43) | 2629.45 (1196.02 - 5915.17) | -0.29 (-0.31 - -0.27) |
| El Salvador | 2912.87 (2524.98 - 3315.26) | 629.54 (427.27 - 871.9) | 7336.76 (6414.74 - 8388.85) | 636.54 (430.13 - 877.54) | 0.07 (0.05 - 0.08) | 16713.63 (14449.5 - 19033.54) | 3612.05 (2819.97 - 4530.67) | 42110.67 (36610.26 - 48306.05) | 3654.1 (2854.08 - 4596.48) | 0.07 (0.05 - 0.08) | 516.94 (131.94 - 1347.23) | 109.98 (26.21 - 301.8) | 1402.15 (354.34 - 3491.96) | 107.61 (25.94 - 280.03) | -0.07 (-0.09 - -0.04) | 9890.59 (4847.53 - 21150.07) | 2122.45 (1008.32 - 4634.05) | 25098.73 (12298.74 - 51323.71) | 2098.62 (1006.01 - 4407.04) | -0.03 (-0.05 - -0.02) |
| Equatorial Guinea | 147.86 (126.68 - 170.1) | 734.27 (502.62 - 1002.26) | 386.69 (333.15 - 444.73) | 705.02 (481.01 - 964.47) | -0.16 (-0.17 - -0.15) | 868.59 (736.87 - 996.72) | 4420.59 (3470.77 - 5526.03) | 2277.7 (1939.74 - 2620.54) | 4218.45 (3307.29 - 5273.49) | -0.18 (-0.19 - -0.17) | 23.53 (5.49 - 63.47) | 174.35 (39.11 - 496.18) | 78.57 (17.99 - 210.55) | 194.78 (43.76 - 537.04) | 0.4 (0.31 - 0.49) | 552.56 (254.42 - 1255.28) | 3084.11 (1323.69 - 7226.07) | 1616.93 (704.75 - 3679.9) | 3271.91 (1355.11 - 7644.79) | 0.2 (0.15 - 0.26) |
| Eritrea | 520.53 (440.85 - 602.22) | 626.6 (424.42 - 861.36) | 1516.93 (1293.71 - 1752.57) | 589.18 (398.78 - 815.62) | -0.19 (-0.19 - -0.18) | 3078.69 (2602 - 3545.76) | 3680.53 (2874.94 - 4617.01) | 8930.26 (7531.6 - 10264.79) | 3443.75 (2682.58 - 4319.22) | -0.2 (-0.21 - -0.19) | 71.23 (16.31 - 205.44) | 141.36 (32.11 - 402.29) | 297.21 (68.31 - 819.28) | 177.02 (39.62 - 491.86) | 0.73 (0.65 - 0.81) | 1902.25 (878.38 - 4225.38) | 2539.12 (1113.6 - 5882.29) | 6622.81 (2824.87 - 15402.42) | 2904.87 (1167.94 - 6894.21) | 0.44 (0.39 - 0.5) |
| Estonia | 2180.75 (1864.54 - 2533.59) | 646.67 (439.05 - 893.68) | 3584.93 (3113.33 - 4131.22) | 639.32 (433.43 - 877.84) | -0.01 (-0.06 - 0.04) | 12657.23 (10720.98 - 14628.95) | 3767.68 (2936.09 - 4732.84) | 20848.9 (17821.66 - 24004.11) | 3716.28 (2891.1 - 4661.3) | -0.02 (-0.07 - 0.03) | 355.53 (85.21 - 1004.3) | 122.34 (28.13 - 347.15) | 783.36 (194.04 - 2061.23) | 129.5 (30.85 - 347.49) | 0.19 (0.17 - 0.2) | 7287.33 (3565.02 - 15812.93) | 2282.51 (1063.41 - 5139.04) | 13472.65 (6469.96 - 28456.77) | 2343.98 (1076.02 - 5117.88) | 0.1 (0.07 - 0.14) |
| Eswatini | 183.36 (156.67 - 211.17) | 594.56 (401.49 - 819.84) | 323.39 (274.16 - 375.96) | 571.71 (387.06 - 790.03) | -0.13 (-0.14 - -0.12) | 1067.51 (913.11 - 1221.53) | 3453.63 (2686.55 - 4336.87) | 1883.55 (1584.33 - 2173.52) | 3309.51 (2584.53 - 4168.28) | -0.15 (-0.16 - -0.13) | 31.74 (7.62 - 85) | 137.55 (30.78 - 392.6) | 53.4 (12.14 - 152.94) | 137.62 (30.64 - 381.05) | 0.18 (0.12 - 0.23) | 698.91 (317.33 - 1543.92) | 2436.8 (1050.38 - 5654.07) | 1272.14 (571.64 - 2905.01) | 2442.88 (1032.83 - 5638.59) | 0.1 (0.07 - 0.12) |
| Ethiopia | 11509.15 (9836.23 - 13297.33) | 623.17 (424.36 - 856.09) | 30925.74 (26776.01 - 35426.45) | 577.7 (393.35 - 792.98) | -0.21 (-0.25 - -0.18) | 68116.02 (57940.34 - 79186.37) | 3626.02 (2827.79 - 4560.92) | 179526.66 (154666.58 - 205972.6) | 3324.81 (2593.13 - 4210.23) | -0.25 (-0.29 - -0.22) | 1862.69 (431.17 - 5162.95) | 158.18 (36.63 - 444.3) | 7338.45 (1802.83 - 19472.04) | 168.9 (40.5 - 449.82) | 0.23 (0.15 - 0.31) | 46389.49 (20568.22 - 108298.57) | 2740.08 (1154.13 - 6495.36) | 141198.38 (60923.8 - 322614.79) | 2799.17 (1155.34 - 6519.47) | 0.08 (0.01 - 0.15) |
| Fiji | 227.44 (190.68 - 266.95) | 637.96 (432.81 - 885.02) | 536.61 (452.34 - 625.9) | 626.45 (422.79 - 866.88) | -0.08 (-0.09 - -0.07) | 1321.31 (1107.48 - 1543.84) | 3725.88 (2874 - 4718.72) | 3115.48 (2638.15 - 3616.13) | 3652.21 (2828.58 - 4611.51) | -0.09 (-0.1 - -0.07) | 35.04 (8.21 - 99.32) | 138.7 (31.56 - 398.57) | 75.64 (17.91 - 206.05) | 132.74 (30.34 - 367.07) | -0.21 (-0.25 - -0.18) | 803.52 (372.21 - 1823.35) | 2466.27 (1080.17 - 5717.96) | 1784.87 (847.5 - 3860.65) | 2350.51 (1060.52 - 5219.72) | -0.2 (-0.22 - -0.18) |
| Finland | 8778.59 (7577.46 - 10040.92) | 693.78 (478.77 - 939.02) | 16434.86 (13992.33 - 19098.57) | 620.39 (420.91 - 856.11) | -0.4 (-0.41 - -0.39) | 49468.2 (42327.52 - 56315.29) | 3970.52 (3098.3 - 4937.18) | 94301.31 (79142.64 - 108888.63) | 3546.68 (2740.95 - 4495.06) | -0.4 (-0.41 - -0.39) | 1895.51 (488.19 - 5029.97) | 165.87 (40.45 - 440.55) | 4584.68 (1229.89 - 11231.62) | 154.18 (39.08 - 393.05) | -0.18 (-0.22 - -0.14) | 33452.25 (15512.35 - 73797.06) | 2778.33 (1237.95 - 6172.46) | 70870.39 (32748.64 - 148451.88) | 2555.39 (1139.02 - 5495.53) | -0.25 (-0.27 - -0.22) |
| France | 90035.75 (80369.75 - 99725.13) | 568.41 (435.65 - 715.61) | 160511.54 (140285.31 - 181357.54) | 535.03 (391 - 697.24) | -0.23 (-0.26 - -0.19) | 502031.7 (442790.23 - 558793.18) | 3178.95 (2640.31 - 3766.86) | 911446.25 (793550.4 - 1033067.86) | 3015.61 (2434.79 - 3669.2) | -0.2 (-0.23 - -0.16) | 20454.75 (5108.14 - 54342.17) | 135.48 (32.59 - 365.75) | 46513.25 (12115.86 - 116191.61) | 127.92 (32.16 - 328.69) | -0.18 (-0.2 - -0.16) | 348761.54 (160410.84 - 761599.71) | 2260.57 (1011.64 - 5062.48) | 694675.71 (316504.57 - 1473596.29) | 2137.67 (966.3 - 4602.57) | -0.18 (-0.2 - -0.17) |
| Gabon | 538.26 (460.63 - 618.29) | 713.29 (487.73 - 973.63) | 834.28 (719.3 - 957.6) | 696.87 (476.38 - 951.49) | -0.06 (-0.07 - -0.04) | 3192.63 (2728.33 - 3668.43) | 4316 (3371.18 - 5384.26) | 4959.45 (4241.42 - 5698.36) | 4204.57 (3289.36 - 5264.57) | -0.05 (-0.06 - -0.04) | 119.09 (29.14 - 314.66) | 208.31 (49.98 - 540.52) | 185.63 (44.66 - 504.44) | 208.91 (48.87 - 565.48) | 0 (0 - 0.01) | 2356.3 (1047.16 - 5126.84) | 3451.22 (1442.49 - 7776.32) | 3701.31 (1649.44 - 8576.7) | 3424.47 (1428.45 - 7906.68) | -0.02 (-0.04 - -0.01) |
| Gambia | 179.21 (153.46 - 206.22) | 475.59 (321.82 - 656.98) | 522.78 (446.58 - 608.08) | 439.41 (294.77 - 609.64) | -0.26 (-0.27 - -0.25) | 1042.18 (885.61 - 1197.42) | 2699.74 (2103.4 - 3382.15) | 3007.08 (2557.75 - 3464.15) | 2487.91 (1929.37 - 3138.07) | -0.28 (-0.29 - -0.27) | 31.36 (7.43 - 84.34) | 116.38 (25.8 - 327.47) | 113.46 (26.25 - 313.98) | 118.64 (26.76 - 342.68) | 0 (-0.05 - 0.04) | 687.51 (315.85 - 1528.45) | 1981.09 (847.74 - 4591.32) | 2224.33 (964.16 - 5219.13) | 1979.15 (791.58 - 4836.97) | -0.07 (-0.12 - -0.02) |
| Georgia | 6108.44 (5241.55 - 7019.33) | 644.75 (437.2 - 887.95) | 7371.94 (6392.39 - 8397.98) | 646.21 (444.13 - 885.79) | 0.01 (0 - 0.02) | 35286.91 (30309.48 - 40440.91) | 3726.47 (2911.74 - 4668.35) | 42691.21 (36958.62 - 48662.11) | 3728.95 (2917.47 - 4670.6) | 0.01 (0 - 0.03) | 1006.61 (246.02 - 2797.01) | 121.32 (28.3 - 339.03) | 1563.69 (413.71 - 4003.35) | 124.34 (30.25 - 335.7) | 0.09 (0 - 0.17) | 20668.3 (9903.56 - 44611.81) | 2275.66 (1067.22 - 5069.84) | 27408.73 (13282.73 - 56897.34) | 2316.39 (1074.39 - 5109.54) | 0.06 (0.02 - 0.11) |
| Germany | 195174.73 (173231.49 - 217528.09) | 831.23 (613.98 - 1077.54) | 335019.57 (294656.7 - 377462.54) | 814.9 (586.69 - 1076.26) | -0.1 (-0.13 - -0.06) | 1068859.35 (939121.22 - 1214510.08) | 4660.44 (3816.45 - 5655.8) | 1971203.22 (1701906.37 - 2247030.56) | 4730.91 (3781.7 - 5782.38) | 0.06 (0.03 - 0.09) | 37336.33 (9390.89 - 94734.56) | 174.02 (44.06 - 452.48) | 78481.37 (21390.04 - 186561.33) | 172.57 (45.26 - 432.28) | -0.07 (-0.12 - -0.02) | 666248.22 (323238.85 - 1402598.48) | 2979.7 (1418.05 - 6339.73) | 1280635.8 (620083.31 - 2579457.89) | 2968.29 (1417.23 - 6101.06) | -0.05 (-0.08 - -0.01) |
| Ghana | 2867.6 (2445.55 - 3312.87) | 433.83 (291.88 - 602.33) | 7743.88 (6617.74 - 8918.22) | 423.56 (284.52 - 585.41) | -0.06 (-0.07 - -0.05) | 16655.93 (14181.16 - 19201.7) | 2448.72 (1896.51 - 3103.04) | 44875.53 (38175.55 - 51615.88) | 2390.77 (1854.91 - 3010.65) | -0.06 (-0.07 - -0.05) | 478.28 (112.72 - 1367.42) | 98.58 (22.25 - 279.99) | 1572.14 (359.42 - 4388.8) | 114.12 (25.67 - 332.05) | 0.5 (0.45 - 0.55) | 10679.78 (4880.35 - 25059.48) | 1724.5 (748.03 - 4014.23) | 32516.24 (14335.3 - 77583.87) | 1901.24 (775.91 - 4637.02) | 0.34 (0.29 - 0.38) |
| Greece | 18317.85 (15587.6 - 21419.08) | 710.99 (481.92 - 973.97) | 36538.26 (31278.05 - 42348.04) | 682.97 (466.85 - 939.21) | -0.11 (-0.13 - -0.08) | 103021.34 (87411.72 - 119665.68) | 4048.7 (3136.68 - 5094.5) | 209689.24 (178575.86 - 243790.9) | 3866.11 (3004.17 - 4853.19) | -0.13 (-0.15 - -0.1) | 3203.89 (792.3 - 8834.7) | 146.52 (35 - 404.63) | 8914.48 (2262.06 - 22782.64) | 142.89 (35 - 377.6) | -0.09 (-0.11 - -0.08) | 62127.12 (29750.32 - 134078.85) | 2579.24 (1184.51 - 5779.02) | 142639.77 (68139.96 - 303978.55) | 2504.41 (1157.98 - 5409.47) | -0.1 (-0.1 - -0.09) |
| Greenland | 26.44 (22.28 - 30.97) | 779.6 (529.41 - 1075.13) | 64.98 (55.6 - 75.25) | 765.61 (522.37 - 1055.07) | -0.07 (-0.1 - -0.05) | 153.64 (129.7 - 179.25) | 4594.5 (3542.06 - 5790.26) | 379.02 (321.72 - 437.54) | 4487.83 (3482.01 - 5643.21) | -0.09 (-0.12 - -0.06) | 3.54 (0.83 - 9.75) | 155.96 (35.46 - 442.24) | 9.36 (2.36 - 24.67) | 151.33 (36.01 - 397.47) | -0.06 (-0.09 - -0.03) | 83.96 (41.08 - 181.73) | 2816.19 (1301.87 - 6369.65) | 209.64 (103.91 - 429.01) | 2730.05 (1284.07 - 5840.14) | -0.08 (-0.1 - -0.05) |
| Grenada | 81.18 (70.26 - 94.09) | 564.3 (382.47 - 777.27) | 86.2 (74.47 - 99.2) | 555.97 (377.49 - 768.67) | -0.02 (-0.05 - 0.01) | 470.82 (401.34 - 541.2) | 3265.55 (2550.88 - 4097.22) | 495.57 (424.66 - 570.8) | 3221.05 (2509.21 - 4055.68) | -0.02 (-0.05 - 0.01) | 15.92 (3.79 - 42.53) | 104.25 (24.15 - 287.2) | 12.42 (2.9 - 34.85) | 98.61 (22.56 - 279.47) | 0.17 (0.07 - 0.26) | 286.6 (138.01 - 618.53) | 1957.23 (912.64 - 4320.69) | 275.22 (134.07 - 602.66) | 1894.23 (887.02 - 4196.51) | 0.09 (0.04 - 0.14) |
| Guam | 48.44 (40.37 - 56.95) | 619.76 (418.2 - 861.52) | 232.73 (200.87 - 265.58) | 616.32 (417.83 - 851.24) | -0.02 (-0.03 - -0.02) | 283.87 (239.05 - 331.8) | 3616.09 (2779.55 - 4579.19) | 1370.18 (1179.87 - 1575.68) | 3602.9 (2791.31 - 4549.34) | -0.02 (-0.03 - -0.01) | 7.22 (1.71 - 19.86) | 136.23 (31.85 - 378.98) | 47.62 (13.92 - 106.45) | 107.7 (29.18 - 260.07) | -0.7 (-0.77 - -0.64) | 174.56 (81.46 - 388.99) | 2444.95 (1085.29 - 5525.74) | 843.6 (426.3 - 1611.78) | 2131.73 (1047.32 - 4302.66) | -0.43 (-0.47 - -0.39) |
| Guatemala | 2536.64 (2178.08 - 2911.79) | 644.89 (437.09 - 886.82) | 10346.07 (8929.81 - 11799.85) | 638.13 (434.93 - 876.27) | -0.02 (-0.03 - 0) | 14564.42 (12516.19 - 16636.98) | 3704.91 (2889.1 - 4667.34) | 59407.47 (51068.57 - 68317.97) | 3664.63 (2869.9 - 4579.1) | -0.02 (-0.03 - 0) | 320.26 (74.16 - 898.94) | 111.31 (25 - 318.11) | 1521.24 (373.08 - 4089.15) | 107.65 (24.82 - 298.03) | -0.13 (-0.16 - -0.11) | 7805.13 (3846.46 - 17158.6) | 2140.71 (994.06 - 4847.74) | 32503.88 (15802.13 - 66897.68) | 2088.89 (990.88 - 4518.03) | -0.09 (-0.1 - -0.07) |
| Guinea | 2029.63 (1744.83 - 2343.2) | 468.75 (314.15 - 645.98) | 3093.41 (2668.66 - 3547.55) | 441.65 (300.03 - 609.22) | -0.2 (-0.21 - -0.18) | 11711.13 (10088.37 - 13390.86) | 2657.02 (2079.32 - 3341.35) | 17789.94 (15262.75 - 20426.66) | 2496.69 (1943.39 - 3141.38) | -0.2 (-0.22 - -0.18) | 357.55 (88.3 - 992.42) | 100.32 (22.28 - 282.82) | 644.79 (145.99 - 1777.95) | 108.9 (23.66 - 306.83) | 0.31 (0.28 - 0.35) | 7484.88 (3349.61 - 16190.03) | 1798.01 (777.01 - 4132.6) | 12548.64 (5689.06 - 28961.2) | 1860.61 (783.4 - 4367.17) | 0.12 (0.09 - 0.15) |
| Guinea-Bissau | 177.94 (150.05 - 206.4) | 446.16 (298.79 - 617.24) | 288.74 (245.42 - 334.32) | 434.88 (293.15 - 602.11) | -0.06 (-0.07 - -0.05) | 1040.78 (882.26 - 1204.1) | 2529.47 (1960.18 - 3186.65) | 1690.72 (1426.79 - 1939.3) | 2456.98 (1908.08 - 3116.36) | -0.08 (-0.09 - -0.07) | 30.55 (7.37 - 84.41) | 111.23 (24.79 - 316.67) | 53.07 (12 - 146.99) | 116.17 (25.44 - 341.57) | 0.17 (0.13 - 0.2) | 708.78 (315.69 - 1607.1) | 1907.32 (795.86 - 4527.13) | 1216.51 (526.02 - 2873.04) | 1961.1 (799.45 - 4807.03) | 0.1 (0.08 - 0.12) |
| Guyana | 269.82 (231.54 - 311.98) | 550.53 (374.32 - 758.26) | 449.81 (389.66 - 520.26) | 545.42 (370.56 - 749.75) | -0.04 (-0.05 - -0.03) | 1555.66 (1329.65 - 1790.83) | 3175.43 (2478.46 - 3984.49) | 2589.69 (2204.54 - 2962.9) | 3141.69 (2448.13 - 3943.25) | -0.05 (-0.06 - -0.04) | 36.67 (8.81 - 101.9) | 89.77 (20.71 - 254.34) | 62.91 (14.85 - 173.11) | 91.57 (20.38 - 257.77) | 0.01 (-0.1 - 0.12) | 822.82 (403.16 - 1739.25) | 1761.95 (837.61 - 3857.92) | 1408.99 (697.06 - 3046.35) | 1782.56 (838.87 - 3935.27) | 0 (-0.08 - 0.08) |
| Haiti | 2038.33 (1742.52 - 2335.39) | 580.24 (393.57 - 799) | 4252.73 (3653.55 - 4935.66) | 537.15 (365.36 - 741.8) | -0.27 (-0.28 - -0.25) | 11703.26 (9924.9 - 13403.91) | 3358.42 (2622.15 - 4215.16) | 24481.3 (20932.52 - 28144.51) | 3099.96 (2434.31 - 3896.95) | -0.28 (-0.3 - -0.27) | 252.2 (59.95 - 702.09) | 100.83 (22.91 - 291.48) | 579.66 (127.89 - 1671.65) | 97.05 (20.48 - 283.25) | -0.1 (-0.11 - -0.09) | 6432.38 (3141.52 - 14397.52) | 1974.55 (918.88 - 4455.4) | 13891.68 (6691.5 - 31599.26) | 1866.73 (852.24 - 4250.56) | -0.17 (-0.18 - -0.16) |
| Honduras | 1723.44 (1492.83 - 1988.85) | 652.48 (443.38 - 898.19) | 5300.45 (4541.48 - 6129.68) | 641.86 (436.48 - 881.72) | -0.06 (-0.06 - -0.05) | 9877.07 (8483.01 - 11290.01) | 3745.25 (2921.84 - 4680.86) | 30259.63 (25759.15 - 34930.76) | 3675.08 (2872.59 - 4602.23) | -0.07 (-0.07 - -0.06) | 260.06 (62.6 - 695.9) | 119.77 (27.3 - 334.16) | 901.02 (213.11 - 2514.72) | 135.21 (31.22 - 382.44) | 0.36 (0.27 - 0.45) | 5796.01 (2753.55 - 12494.66) | 2298.1 (1064.81 - 5102.78) | 19432.55 (8983.57 - 43856.7) | 2481.04 (1089.55 - 5689.5) | 0.24 (0.19 - 0.29) |
| Hungary | 15114.31 (12981.11 - 17719.03) | 637.89 (434.3 - 880.61) | 23665.51 (20373.04 - 27126.22) | 631.48 (430.72 - 869.81) | -0.02 (-0.05 - 0.02) | 86908.35 (73906.79 - 100292.3) | 3696.25 (2866.12 - 4646.8) | 136488.39 (116814.81 - 156859.77) | 3652.3 (2844.61 - 4600.79) | -0.02 (-0.06 - 0.02) | 2351.27 (566.28 - 6617.8) | 122.13 (28.13 - 342.43) | 4551.06 (1109.28 - 12082.25) | 118.39 (27.95 - 323.12) | -0.06 (-0.07 - -0.05) | 49329.98 (23924.91 - 109675.29) | 2240.41 (1025.55 - 5052.94) | 83255.08 (40051.61 - 172040) | 2211.53 (1032.99 - 4796.71) | -0.01 (-0.02 - 0.01) |
| Iceland | 406.86 (352.55 - 459.45) | 774.33 (553.96 - 1023.6) | 783.56 (674.86 - 880.57) | 701.79 (507.66 - 925.41) | -0.36 (-0.38 - -0.35) | 2307 (1972.4 - 2623.24) | 4384.96 (3476.05 - 5376.87) | 4455.07 (3800.52 - 5029.69) | 3937.75 (3121.43 - 4819.97) | -0.4 (-0.42 - -0.38) | 78.66 (19.64 - 203.6) | 145.54 (35.52 - 386.45) | 182.94 (50.05 - 450.73) | 143.37 (36.29 - 362.48) | 0 (-0.05 - 0.05) | 1404.3 (688.77 - 2929.95) | 2648.79 (1268.36 - 5666.47) | 2958.67 (1439.8 - 6026.27) | 2506.66 (1188.92 - 5214.13) | -0.16 (-0.19 - -0.13) |
| India | 219412.39 (188496.92 - 253909.75) | 449.37 (304.79 - 618.93) | 698848.94 (598423.17 - 807081.45) | 445.64 (300.93 - 615.47) | -0.16 (-0.21 - -0.11) | 1250232.8 (1070556.92 - 1433554.81) | 2508.37 (1957.4 - 3173.47) | 3938968.82 (3357942.36 - 4543345.7) | 2482.36 (1928.3 - 3151.81) | -0.17 (-0.22 - -0.12) | 29871.61 (6762.55 - 87864.42) | 78.55 (17.18 - 231.61) | 132098.67 (31151.45 - 363865.7) | 99.96 (23.25 - 277.07) | 0.89 (0.83 - 0.95) | 724442.59 (335000.12 - 1719635.71) | 1516.2 (686.46 - 3551.03) | 2698981.73 (1188454.97 - 6066883.22) | 1771.16 (759.19 - 4141.08) | 0.52 (0.49 - 0.56) |
| Indonesia | 70829.32 (60880.91 - 81458.95) | 651.33 (443.43 - 896.82) | 170381.09 (145861.58 - 197494.88) | 636.37 (431.27 - 878.83) | -0.07 (-0.09 - -0.05) | 425410.66 (365473.53 - 492396.85) | 3860.96 (2995.87 - 4889.76) | 1023891.13 (878270.57 - 1187362.85) | 3766.7 (2910.54 - 4786.14) | -0.08 (-0.1 - -0.05) | 8839.91 (2070.87 - 24663.38) | 103.21 (22.31 - 294.38) | 25906.72 (6220.64 - 72322.1) | 135.23 (31.18 - 389.19) | 0.78 (0.68 - 0.87) | 220881.43 (109569.14 - 469254.93) | 2107.98 (998.67 - 4689.36) | 603591.17 (284384.38 - 1329386.62) | 2443.17 (1086.55 - 5689.39) | 0.42 (0.35 - 0.49) |
| Iran (Islamic Republic of) | 21534.68 (18386.05 - 24772.47) | 780.88 (535.44 - 1066.36) | 82339.18 (71591.06 - 94056.65) | 758.4 (518.88 - 1036.72) | -0.08 (-0.09 - -0.07) | 125724.36 (108075.93 - 144261.82) | 4607.06 (3604.8 - 5796.43) | 481638.52 (415935.12 - 551717.98) | 4439.23 (3462.57 - 5591.15) | -0.11 (-0.12 - -0.11) | 3379.94 (825.45 - 8874.78) | 160.9 (38.61 - 438.79) | 14841.36 (3696.45 - 39380.58) | 148.38 (35.69 - 398.65) | -0.29 (-0.32 - -0.27) | 75903.1 (35917.56 - 167135.93) | 2944.68 (1345.82 - 6587.83) | 292167.17 (139202.68 - 624335.75) | 2752.78 (1268.98 - 6021.1) | -0.25 (-0.26 - -0.23) |
| Iraq | 8930.21 (7723.44 - 10195) | 769.91 (527.65 - 1046.56) | 20338.67 (17577.18 - 23454.69) | 737.62 (503.16 - 1010.44) | -0.17 (-0.19 - -0.16) | 53051.96 (45129.65 - 60772.82) | 4581.01 (3569.27 - 5731.54) | 118334.69 (100644.13 - 135954.08) | 4366.34 (3404.51 - 5464.77) | -0.19 (-0.21 - -0.18) | 1764.32 (433.4 - 4572.63) | 154.12 (37.3 - 417.39) | 3277.94 (819.43 - 8717.58) | 151.9 (35.5 - 417.03) | -0.16 (-0.19 - -0.12) | 33002.36 (15536.07 - 70938.91) | 2854.29 (1313.25 - 6209.9) | 69979.91 (33179.7 - 150803.38) | 2753.75 (1246.86 - 6133.06) | -0.2 (-0.23 - -0.17) |
| Ireland | 4870.06 (4148.69 - 5675.57) | 701.94 (475.7 - 959.45) | 9357.5 (8001.59 - 10843.19) | 642.4 (435.3 - 886.78) | -0.3 (-0.32 - -0.28) | 27105.39 (22929.24 - 31446) | 3984.68 (3082.96 - 5008.79) | 52773.35 (44641.2 - 61000.22) | 3625.84 (2798.33 - 4560.66) | -0.32 (-0.34 - -0.29) | 855.95 (208.44 - 2335.58) | 149.16 (35.34 - 413.32) | 2111.39 (549.64 - 5283.15) | 142.09 (35.69 - 362.73) | -0.09 (-0.12 - -0.07) | 16537.44 (7963.15 - 36091.39) | 2590.97 (1178.74 - 5837.49) | 35649.16 (17135.36 - 73737.11) | 2434.26 (1128.08 - 5194.26) | -0.17 (-0.19 - -0.15) |
| Israel | 5583.14 (4713.44 - 6534.21) | 688.08 (464.46 - 945.67) | 15043.65 (12912.35 - 17361.1) | 647.53 (440.12 - 887.3) | -0.2 (-0.21 - -0.2) | 30999.84 (26225.57 - 36171.61) | 3897.67 (3021.27 - 4929.72) | 85252.5 (72785.96 - 98939.13) | 3662.91 (2837.97 - 4627.26) | -0.21 (-0.22 - -0.2) | 943.96 (235.46 - 2596.72) | 144.04 (34.41 - 396.54) | 3603.46 (948.94 - 8818.38) | 143.25 (36.11 - 366.42) | -0.02 (-0.05 - 0.02) | 18727.79 (9028.66 - 41355.88) | 2523.12 (1148.86 - 5664.46) | 58884.82 (28304.43 - 123745.1) | 2457.16 (1122.8 - 5239.61) | -0.09 (-0.12 - -0.07) |
| Italy | 103175.4 (86684.22 - 119818.37) | 658.97 (458.9 - 889.9) | 244842.32 (210765.34 - 281356.99) | 771.16 (521.21 - 1058.6) | 0.3 (0.09 - 0.5) | 616578.96 (515890.29 - 719871.08) | 3957.07 (3088.57 - 4913.35) | 1407328.98 (1192138.43 - 1627998.04) | 4361.16 (3381.11 - 5519.76) | 0.14 (-0.01 - 0.29) | 25530.99 (6485.65 - 67491.26) | 185.79 (46.01 - 490.53) | 64673.34 (17871.31 - 156498.69) | 175.47 (45.99 - 440.97) | -0.09 (-0.13 - -0.05) | 445130.83 (202607.68 - 968864.03) | 3008.48 (1312.24 - 6744.98) | 1001075.62 (475669.53 - 2034611.87) | 2957.36 (1373.82 - 6225.28) | -0.03 (-0.06 - 0) |
| Jamaica | 2007.8 (1740.31 - 2308.87) | 626.57 (422.03 - 863.68) | 3351.2 (2904.79 - 3821.4) | 593.11 (401.53 - 815.74) | -0.2 (-0.21 - -0.18) | 11769.32 (10046.77 - 13452.43) | 3681.28 (2874.4 - 4632.88) | 19701.47 (16933.43 - 22379.76) | 3487.45 (2714.17 - 4371.33) | -0.2 (-0.21 - -0.18) | 304.48 (73.6 - 835.9) | 96.69 (22.73 - 266.27) | 645.01 (164.79 - 1681.22) | 94.35 (22.37 - 259.17) | -0.09 (-0.12 - -0.05) | 6263.6 (3123.62 - 13045.37) | 1975.37 (970.3 - 4198.63) | 11491.07 (5656.9 - 23933.61) | 1904.43 (938.06 - 4054.76) | -0.14 (-0.17 - -0.11) |
| Japan | 175892.95 (151585.75 - 203132.2) | 655.61 (450.36 - 893.43) | 567743.19 (496001.86 - 652813.41) | 665.55 (455.09 - 910.32) | 0.22 (0.16 - 0.28) | 984451.56 (847373.12 - 1129741.17) | 3706.66 (2897.65 - 4672.12) | 3326449.52 (2850932.81 - 3829623.71) | 3857.34 (3022.35 - 4861.2) | 0.32 (0.26 - 0.38) | 36810.06 (9390.16 - 95931.37) | 160.2 (40.11 - 420.35) | 172633.28 (50366.53 - 398305.42) | 155.2 (42.1 - 377.19) | -0.07 (-0.1 - -0.04) | 675006.32 (314406.54 - 1469704.77) | 2676.9 (1192.98 - 5893.64) | 2524923.21 (1201119.4 - 5015009.85) | 2652.07 (1243.99 - 5450.22) | 0.04 (0.01 - 0.06) |
| Jordan | 1109.71 (956.15 - 1268.65) | 758.35 (517.66 - 1043.25) | 6607.26 (5712.89 - 7594.35) | 765.09 (525.52 - 1045.29) | 0.07 (0.03 - 0.11) | 6506.34 (5595.75 - 7513.55) | 4505.96 (3513.4 - 5661.08) | 38367.12 (32985.55 - 43930.11) | 4519.31 (3546.72 - 5656.87) | 0.05 (0.01 - 0.09) | 183.13 (47 - 485.94) | 154.66 (37.52 - 419.15) | 903.87 (217.39 - 2459.46) | 137.75 (32.7 - 384.48) | -0.43 (-0.46 - -0.4) | 3888.84 (1884.25 - 8289.61) | 2835.8 (1312.9 - 6220.57) | 20731.81 (10327.54 - 44623.42) | 2618 (1240.52 - 5770.01) | -0.29 (-0.32 - -0.26) |
| Kazakhstan | 11217.83 (9742.6 - 13028.53) | 645.15 (440.51 - 887.24) | 14482.65 (12495.65 - 16665.91) | 629.47 (427.95 - 861.25) | -0.08 (-0.11 - -0.06) | 64658.55 (55617.82 - 74279.55) | 3722.19 (2901.56 - 4683.35) | 83639.7 (71683.95 - 96244.39) | 3627.59 (2826.86 - 4543.54) | -0.08 (-0.11 - -0.06) | 1874.31 (460.17 - 5052.19) | 122.51 (28.86 - 340.17) | 2118.84 (510.22 - 6122.36) | 114.52 (26.03 - 329.68) | -0.33 (-0.39 - -0.27) | 37920.57 (18341.98 - 82836.04) | 2271.26 (1054.27 - 5044.1) | 46923.69 (23159.99 - 103265.06) | 2156.14 (993.49 - 4859.19) | -0.25 (-0.3 - -0.21) |
| Kenya | 6007.35 (5173.22 - 6880.7) | 601.98 (410.6 - 823.41) | 14783.7 (12728.33 - 16972.07) | 592.31 (403.4 - 811.68) | -0.06 (-0.08 - -0.03) | 34994.21 (30034.94 - 40276.64) | 3483.15 (2717.33 - 4395.9) | 86918.17 (74663.35 - 99491.29) | 3440.76 (2688.41 - 4339.96) | -0.04 (-0.07 - -0.01) | 1095.54 (253.86 - 3103.04) | 140.72 (32.27 - 387.31) | 3112.43 (735.93 - 8026.98) | 169.16 (38.68 - 435.74) | 0.67 (0.65 - 0.7) | 22980.4 (10493.52 - 51992.16) | 2461.52 (1065.27 - 5590.36) | 64097.92 (27625.02 - 142671.72) | 2792.11 (1137.02 - 6282.29) | 0.46 (0.44 - 0.48) |
| Kiribati | 25.74 (21.89 - 29.88) | 688.44 (466.4 - 952.23) | 49.25 (42.06 - 57.04) | 691.93 (470.52 - 951.42) | 0.01 (-0.03 - 0.04) | 149 (126.87 - 172.41) | 4014.71 (3120 - 5064.88) | 285.52 (242.41 - 329.59) | 4029.23 (3139.71 - 5054.05) | 0 (-0.03 - 0.04) | 3.74 (0.86 - 10.29) | 139.85 (30.02 - 397.49) | 7.77 (1.77 - 21.24) | 161.32 (35.7 - 450.54) | 0.46 (0.42 - 0.5) | 89.68 (42.36 - 204.88) | 2582.95 (1149.62 - 5941.59) | 180.75 (82.95 - 398.78) | 2814.94 (1230.83 - 6441.85) | 0.28 (0.24 - 0.31) |
| Kuwait | 482.1 (418.48 - 548.02) | 784.06 (535.2 - 1071.67) | 2469.06 (2158.26 - 2800.54) | 747.5 (513.55 - 1022.92) | -0.15 (-0.17 - -0.14) | 2866.92 (2474.33 - 3259.66) | 4715.12 (3696.59 - 5867.7) | 14670.71 (12708.97 - 16704.39) | 4430.63 (3474.03 - 5560.7) | -0.2 (-0.22 - -0.18) | 83.16 (21.04 - 210.9) | 159.06 (39.73 - 418.67) | 468.69 (122.98 - 1206.61) | 143.64 (35.81 - 381.53) | -0.36 (-0.47 - -0.24) | 1716.64 (836.89 - 3646.39) | 2936.43 (1378.55 - 6346.05) | 8826.26 (4292.07 - 18195.61) | 2665.62 (1263 - 5672.1) | -0.33 (-0.4 - -0.26) |
| Kyrgyzstan | 2772.32 (2398.98 - 3183.02) | 640.5 (437.97 - 876.46) | 3837.09 (3311.95 - 4373.41) | 645.35 (441.4 - 880.02) | 0.05 (0.04 - 0.06) | 16019.35 (13764.49 - 18293.53) | 3695.33 (2885.13 - 4629.38) | 22312.73 (19196.1 - 25449.32) | 3718.98 (2909.12 - 4624) | 0.05 (0.03 - 0.06) | 471.14 (115.08 - 1270.89) | 125.16 (29.03 - 344.94) | 626.74 (155.85 - 1622.75) | 118.35 (28.2 - 322.6) | -0.22 (-0.24 - -0.19) | 9514.46 (4649.62 - 20255.75) | 2301.15 (1062.7 - 5091.96) | 13138.78 (6258.78 - 27090.7) | 2245.82 (1055.68 - 4893.35) | -0.09 (-0.1 - -0.07) |
| Lao People's Democratic Republic | 1427.5 (1211.49 - 1659.88) | 643.81 (436.56 - 886.55) | 3416.55 (2947.98 - 3938.15) | 629.54 (427.35 - 864.89) | -0.06 (-0.08 - -0.05) | 8594.67 (7245.4 - 9960.16) | 3854.89 (2997.99 - 4843.04) | 20453.11 (17629.08 - 23505.97) | 3746.59 (2919.91 - 4694.38) | -0.08 (-0.09 - -0.06) | 170.88 (39.02 - 492.21) | 115.27 (25.24 - 331.41) | 540.43 (126.5 - 1507.69) | 127.11 (28.73 - 370.16) | 0.36 (0.32 - 0.4) | 4563.5 (2219.06 - 10082.29) | 2253.98 (1053.63 - 5051.51) | 12052.62 (5703.66 - 26918.14) | 2360.62 (1064.2 - 5439.25) | 0.19 (0.16 - 0.21) |
| Latvia | 3899.52 (3338.18 - 4541.17) | 650.29 (441.77 - 891.19) | 5351.89 (4619.34 - 6152.93) | 652.05 (446.46 - 896.4) | 0.04 (0.02 - 0.06) | 22612.2 (19187.16 - 26269.35) | 3779.88 (2934.03 - 4747.69) | 31055.81 (26521.61 - 35960.86) | 3786.02 (2963.65 - 4759.02) | 0.04 (0.02 - 0.06) | 656.46 (155.07 - 1854.51) | 122.76 (28.28 - 345.89) | 1051.07 (257.16 - 2837.19) | 122.53 (28.83 - 338.53) | 0.02 (-0.01 - 0.05) | 13124.22 (6288.39 - 28248.07) | 2283.25 (1060.13 - 5103.34) | 18896.68 (9081.77 - 39862.24) | 2279.58 (1065.19 - 5009.54) | 0.02 (-0.01 - 0.05) |
| Lebanon | 2264.52 (1967.34 - 2604.72) | 794.69 (541.68 - 1089.29) | 8860.68 (7666.33 - 10135.57) | 799.06 (547.55 - 1088.28) | 0 (-0.01 - 0.01) | 13374.47 (11485.79 - 15319.06) | 4749.8 (3713.58 - 5936.41) | 53015.58 (45149.41 - 61164.17) | 4758.96 (3741.55 - 5939.08) | -0.02 (-0.04 - -0.01) | 343.52 (84.56 - 940.37) | 145.62 (33.93 - 404.12) | 1651.1 (421.43 - 4313.79) | 140.49 (34 - 381.26) | -0.05 (-0.17 - 0.07) | 7388.94 (3592.42 - 15344.05) | 2756.13 (1308.74 - 6045.01) | 30296.25 (14982.79 - 62725.97) | 2678.08 (1292.63 - 5719.41) | -0.07 (-0.14 - 0.01) |
| Lesotho | 691.49 (591.99 - 792.27) | 625.66 (422.93 - 861.35) | 731.31 (617.88 - 849.34) | 617.25 (420.93 - 850.78) | -0.02 (-0.03 - -0.01) | 4018.99 (3439.65 - 4602.82) | 3641.52 (2837.92 - 4571.53) | 4254.42 (3628.99 - 4852.55) | 3580.07 (2800.73 - 4501.94) | -0.03 (-0.04 - -0.01) | 117.98 (27.63 - 322.57) | 130.46 (28.52 - 358.95) | 117.96 (28.81 - 330.87) | 138.02 (31.57 - 393.64) | 0.4 (0.28 - 0.51) | 2520.66 (1178.9 - 5280.54) | 2402.56 (1066.15 - 5303.26) | 2762.44 (1251.53 - 6415.85) | 2501.48 (1080.72 - 5891.8) | 0.25 (0.18 - 0.33) |
| Liberia | 616.83 (524.35 - 714.47) | 438.11 (294.35 - 608.23) | 966.14 (823.42 - 1111.7) | 423.29 (285.86 - 582.18) | -0.11 (-0.12 - -0.1) | 3578.9 (3040.64 - 4133.99) | 2479.25 (1926.18 - 3123.29) | 5576.48 (4761.36 - 6377.93) | 2388.6 (1868.47 - 3001.08) | -0.12 (-0.13 - -0.11) | 108.94 (27.12 - 307.37) | 104.65 (23.54 - 303.05) | 195.04 (44.32 - 552.21) | 105.17 (22.51 - 303.91) | 0.07 (0.03 - 0.1) | 2339.98 (1054.23 - 5564.88) | 1787.79 (759.39 - 4330.8) | 3878.45 (1757.35 - 9159.56) | 1781.03 (744.21 - 4319.63) | 0.03 (0 - 0.06) |
| Libya | 2027.73 (1763.21 - 2308.06) | 779.66 (539.15 - 1067.58) | 4837.81 (4204.77 - 5534.99) | 742.45 (505.03 - 1016.2) | -0.16 (-0.18 - -0.14) | 11997.26 (10278.78 - 13607.92) | 4627.76 (3637.28 - 5777.43) | 28376.13 (24519.86 - 32297.83) | 4382.37 (3423.84 - 5475.31) | -0.18 (-0.2 - -0.16) | 463.14 (120.86 - 1150.63) | 181.53 (44.17 - 468.62) | 980.67 (234.95 - 2633.77) | 165.27 (38.63 - 451.28) | -0.21 (-0.26 - -0.16) | 8313.98 (3842.66 - 17201.28) | 3206.36 (1442.32 - 6923.75) | 18469.15 (8828.46 - 41382.85) | 2923.65 (1313.47 - 6500.08) | -0.26 (-0.29 - -0.23) |
| Lithuania | 4889.8 (4181.66 - 5711.79) | 641.31 (437.68 - 881.85) | 7600.04 (6533.94 - 8825.28) | 639.02 (433.08 - 877.69) | 0.02 (-0.01 - 0.05) | 28443.22 (24231.03 - 32982.11) | 3728.87 (2916.19 - 4680.33) | 44226.83 (37972.03 - 51074.04) | 3715.42 (2899.04 - 4658.9) | 0.02 (-0.01 - 0.05) | 856.43 (206.91 - 2392.62) | 122.19 (28.5 - 348.13) | 1543.08 (382.62 - 4188.01) | 123.87 (29.17 - 344.93) | 0.05 (0.03 - 0.06) | 16832.74 (7982.77 - 36088.26) | 2275.7 (1049.6 - 5076.55) | 27476.49 (13169.21 - 59050.52) | 2286.93 (1055.59 - 5040.92) | 0.02 (-0.01 - 0.04) |
| Luxembourg | 543.89 (459.94 - 640.56) | 577.57 (391.68 - 796.45) | 1006.7 (851.86 - 1161.77) | 499.52 (343.65 - 684.35) | -0.52 (-0.58 - -0.45) | 2967.93 (2496.17 - 3467.53) | 3170.16 (2440.05 - 4004.99) | 5477.07 (4632.61 - 6384.5) | 2719.08 (2118.8 - 3461.5) | -0.54 (-0.62 - -0.47) | 83.86 (19.87 - 228.77) | 103.79 (23.71 - 292.52) | 232.11 (60.13 - 583.31) | 104.48 (25.32 - 275.34) | 0.06 (0.02 - 0.11) | 1680.55 (824.2 - 3596.43) | 1895.88 (888.94 - 4186.73) | 3749.33 (1736.38 - 7908.91) | 1794.37 (823.42 - 3906.35) | -0.17 (-0.2 - -0.14) |
| Madagascar | 3490.39 (2983.32 - 4000.77) | 594.17 (407.39 - 814.65) | 5891.61 (4973.76 - 6790.99) | 563.42 (383.75 - 776.7) | -0.16 (-0.17 - -0.14) | 20503.09 (17510.93 - 23403.05) | 3467.14 (2721.34 - 4360) | 34817.35 (29434.33 - 40091.83) | 3283.6 (2561.71 - 4133.69) | -0.16 (-0.17 - -0.15) | 504.09 (115.29 - 1398.48) | 110.89 (25.21 - 318.34) | 842.11 (198.87 - 2289.17) | 116.27 (26.29 - 324.76) | 0.16 (0.14 - 0.18) | 11568.7 (5609.57 - 25572.46) | 2091.87 (961.99 - 4737.65) | 20405.5 (9716.55 - 44936.76) | 2121.92 (960.03 - 4809.42) | 0.06 (0.05 - 0.08) |
| Malawi | 2443.96 (2085.78 - 2815.34) | 595.34 (405.4 - 820.25) | 4768.74 (4078.29 - 5497.89) | 591.78 (401.44 - 813.74) | 0 (-0.02 - 0.01) | 14363.94 (12229.58 - 16462.83) | 3472.92 (2706.65 - 4360.59) | 28038.09 (23818.66 - 32021.34) | 3459.13 (2706.22 - 4334.58) | 0 (-0.01 - 0.02) | 399.72 (97.38 - 1139.04) | 139.98 (32.04 - 394.1) | 946.86 (220.34 - 2574.08) | 159.49 (36.24 - 447.68) | 0.49 (0.45 - 0.53) | 9185.43 (4281.15 - 21013.48) | 2454.29 (1068.58 - 5721.4) | 19963.52 (8934.95 - 45091.34) | 2696.1 (1136.04 - 6338.13) | 0.34 (0.31 - 0.37) |
| Malaysia | 8341.94 (7244.44 - 9508.77) | 652.86 (440.36 - 900.59) | 24270.49 (20776.77 - 27941.3) | 625.44 (419.3 - 867.66) | -0.12 (-0.14 - -0.1) | 50803.19 (43980.35 - 57912.19) | 3971.62 (3096.97 - 4987.94) | 146354.75 (124072.18 - 168005.48) | 3750.85 (2903.95 - 4725.73) | -0.13 (-0.16 - -0.11) | 1709.4 (451.32 - 4453.04) | 139.13 (34.16 - 372.75) | 4679.42 (1144.55 - 12823.63) | 143.61 (34.18 - 396.89) | 0.02 (-0.06 - 0.1) | 32738.89 (15586.4 - 70701.15) | 2580.03 (1177.94 - 5736.36) | 95956.79 (43659.72 - 212285.97) | 2582.38 (1125.14 - 5939.85) | -0.05 (-0.09 - 0) |
| Maldives | 55.03 (45.91 - 64.06) | 616.22 (416.12 - 848.46) | 278.44 (238.37 - 320.52) | 636.84 (433.15 - 878.82) | 0.1 (0.07 - 0.13) | 335.37 (284.78 - 388.94) | 3688 (2875.28 - 4646.6) | 1669.75 (1425.34 - 1919.59) | 3808.24 (2973.56 - 4778.21) | 0.1 (0.07 - 0.14) | 6.41 (1.5 - 17.81) | 104.29 (23.28 - 299) | 46.71 (11.47 - 126.17) | 115.04 (27.63 - 319.89) | 0.3 (0.21 - 0.39) | 177.06 (86.62 - 394.26) | 2093.41 (984.68 - 4725.15) | 958.48 (462.76 - 2009.5) | 2227.07 (1045.27 - 4875.9) | 0.19 (0.14 - 0.23) |
| Mali | 1925.45 (1634.75 - 2233.06) | 461.75 (310.64 - 635.13) | 4239.9 (3629.36 - 4869.15) | 442.43 (300.21 - 609.74) | -0.13 (-0.14 - -0.12) | 11292.41 (9595.06 - 12979.38) | 2624.74 (2042.13 - 3298.98) | 24687.65 (21284.78 - 28326.11) | 2501.05 (1949.92 - 3133.61) | -0.15 (-0.16 - -0.14) | 378.35 (90.22 - 1017.04) | 129.73 (29.34 - 362.71) | 889.5 (211.36 - 2512.07) | 128.66 (29.23 - 361.84) | 0.04 (0 - 0.08) | 8164.66 (3538.46 - 18447.94) | 2128.36 (858.22 - 5041.78) | 18690.46 (7961.09 - 44395.44) | 2109.09 (835.2 - 5104.27) | 0.01 (-0.02 - 0.04) |
| Malta | 474.19 (407.63 - 552.2) | 704.57 (480.93 - 967.64) | 1277.42 (1096.93 - 1475.47) | 653.85 (443.27 - 897.3) | -0.22 (-0.25 - -0.2) | 2653.72 (2257.66 - 3089.36) | 4006.96 (3104.37 - 5041.26) | 7214.19 (6165.14 - 8370.07) | 3700.16 (2866.94 - 4668.64) | -0.24 (-0.27 - -0.21) | 82.72 (20.43 - 225.2) | 145.68 (34.89 - 396.56) | 290.87 (75.84 - 712.44) | 141.55 (36.24 - 360.05) | -0.17 (-0.2 - -0.13) | 1619.94 (769.24 - 3469.14) | 2576.4 (1182.4 - 5723.94) | 4874.32 (2385.67 - 10143.55) | 2460.7 (1137.28 - 5228.71) | -0.19 (-0.21 - -0.17) |
| Marshall Islands | 10.74 (9.01 - 12.58) | 608.42 (411.49 - 842.13) | 18.08 (14.92 - 21.4) | 587.81 (396.37 - 814.38) | -0.11 (-0.12 - -0.11) | 61.96 (51.92 - 73.16) | 3535.26 (2700.97 - 4484.37) | 105.3 (87.65 - 123.72) | 3398.14 (2618.87 - 4312.09) | -0.13 (-0.14 - -0.12) | 1.82 (0.42 - 5.28) | 152.6 (34.57 - 433.18) | 2.69 (0.62 - 8) | 142.29 (31.97 - 406.43) | -0.27 (-0.28 - -0.25) | 41.23 (18.9 - 95.79) | 2613.8 (1112.48 - 6138.48) | 67.18 (30.56 - 161.25) | 2441.89 (1034.91 - 5769.74) | -0.24 (-0.26 - -0.23) |
| Mauritania | 583.93 (491.86 - 679.48) | 471.89 (319.98 - 652.32) | 1214.34 (1041.18 - 1395.76) | 436.96 (294.05 - 605.77) | -0.25 (-0.26 - -0.24) | 3371.28 (2858.63 - 3899.11) | 2669.76 (2076.82 - 3373.81) | 6971.95 (5978.52 - 7995.83) | 2464.71 (1920.36 - 3106.29) | -0.26 (-0.27 - -0.25) | 104.58 (25.06 - 297.23) | 110.73 (25.06 - 311.3) | 263.01 (59.71 - 772.57) | 112.31 (24.27 - 329.51) | -0.02 (-0.05 - 0) | 2232.58 (1013.85 - 5085.01) | 1921.95 (823.83 - 4453.66) | 5104.17 (2276.29 - 12438.1) | 1906.99 (799.66 - 4642.83) | -0.08 (-0.1 - -0.05) |
| Mauritius | 570.35 (489.88 - 660.93) | 632.5 (427.27 - 874.07) | 1792.44 (1547.46 - 2050.21) | 629.1 (423.72 - 867.74) | 0.01 (-0.02 - 0.04) | 3437.52 (2912.06 - 3988.06) | 3789.5 (2940.67 - 4795.79) | 10750.64 (9217.97 - 12238.24) | 3753.41 (2922.75 - 4728.28) | 0 (-0.02 - 0.03) | 90.91 (22.02 - 248.48) | 129.04 (30.42 - 357.61) | 321.29 (79.1 - 870.1) | 119.52 (28.66 - 327.66) | -0.31 (-0.4 - -0.22) | 2009.55 (961.37 - 4477.66) | 2377.75 (1088.14 - 5330.43) | 6380.09 (3063.88 - 13681.65) | 2260.46 (1052.76 - 5019.43) | -0.18 (-0.25 - -0.11) |
| Mexico | 33851.67 (29060.02 - 39291.78) | 600.74 (408.6 - 825.97) | 104119.93 (89562.12 - 119425.63) | 549.37 (372.9 - 760.93) | -0.19 (-0.23 - -0.15) | 189763.11 (162857.79 - 220065.08) | 3342.03 (2601.29 - 4238.93) | 579284.4 (498023.09 - 669311.18) | 3042.79 (2362.79 - 3856.57) | -0.2 (-0.24 - -0.16) | 4161.17 (997.25 - 11813.46) | 88.85 (20.35 - 253.83) | 15431.96 (3627.42 - 43521.59) | 87.11 (20.39 - 249.07) | -0.07 (-0.09 - -0.06) | 96964.92 (48060.72 - 209701.37) | 1789.64 (859.22 - 3943.17) | 321208.72 (157326.48 - 693858.49) | 1716.4 (814.98 - 3798.48) | -0.11 (-0.12 - -0.1) |
| Micronesia (Federated States of) | 40.42 (34.1 - 47.39) | 679.53 (461.97 - 939.49) | 51.34 (43.29 - 59.23) | 684.72 (464.61 - 943.04) | 0.06 (0.05 - 0.07) | 234.06 (198.36 - 272.53) | 3966.42 (3068.91 - 4998.24) | 300.07 (253.85 - 345.97) | 3988.39 (3092.34 - 5021.25) | 0.05 (0.04 - 0.06) | 6.96 (1.68 - 20.16) | 156.12 (35.61 - 445.33) | 8.56 (1.97 - 24.33) | 154.48 (35.14 - 439.8) | -0.03 (-0.07 - 0.01) | 151.9 (69.43 - 362.03) | 2763.28 (1207.47 - 6484.89) | 190.74 (87.98 - 438.94) | 2720.69 (1197.51 - 6348.16) | -0.04 (-0.07 - 0) |
| Monaco | 102.8 (87.62 - 120.2) | 705.87 (479.38 - 975.27) | 133.8 (114.28 - 155.04) | 651.23 (440.3 - 899.34) | -0.28 (-0.28 - -0.27) | 582.4 (492.27 - 677.57) | 4005.88 (3103.73 - 5062.36) | 758.9 (647.17 - 883.03) | 3677.83 (2825.22 - 4650.33) | -0.3 (-0.3 - -0.29) | 22.3 (5.21 - 61.11) | 152.24 (35.4 - 419.93) | 37.99 (9.57 - 88.32) | 160.59 (39.71 - 401.04) | 0.21 (0.17 - 0.24) | 384.12 (181.51 - 845.35) | 2644.18 (1213.43 - 5852.67) | 574.3 (268.68 - 1165.76) | 2630.63 (1196.75 - 5602.57) | 0 (-0.02 - 0.01) |
| Mongolia | 895.66 (767.5 - 1036.38) | 649.13 (444.36 - 888.05) | 1651.01 (1434.17 - 1886.13) | 657.2 (448.63 - 899.2) | 0.05 (0.04 - 0.06) | 5133.3 (4418.2 - 5905.4) | 3743.52 (2935.03 - 4691.93) | 9541.22 (8239.83 - 10883.11) | 3780.45 (2955.66 - 4731.43) | 0.05 (0.04 - 0.06) | 145.29 (36.91 - 392.27) | 133.54 (31.32 - 368.79) | 244.9 (61.31 - 677.98) | 127.66 (30.08 - 353.64) | -0.25 (-0.29 - -0.2) | 3060.22 (1449.39 - 6396.94) | 2400.99 (1091.6 - 5351.11) | 5449.72 (2636.67 - 11974.73) | 2338.31 (1087.79 - 5198.33) | -0.16 (-0.18 - -0.13) |
| Montenegro | 622.1 (539.98 - 718.8) | 649.48 (442.26 - 897.29) | 968.94 (826.2 - 1127.38) | 632.38 (429.96 - 869.48) | -0.13 (-0.17 - -0.08) | 3602.2 (3108.53 - 4152.32) | 3761 (2933.49 - 4735.21) | 5588.22 (4744.34 - 6434.24) | 3658.37 (2846.5 - 4587.82) | -0.13 (-0.18 - -0.09) | 107.42 (25.88 - 291.33) | 121.27 (28.15 - 332.48) | 150.85 (36.64 - 407.68) | 120.58 (27.77 - 334.76) | 0 (-0.07 - 0.06) | 2126.79 (1012.31 - 4580.18) | 2276.67 (1061.71 - 5024.07) | 3201.99 (1528.48 - 6879.24) | 2228.03 (1024.67 - 4956.14) | -0.08 (-0.11 - -0.05) |
| Morocco | 15355.3 (13305.37 - 17595.88) | 778.28 (535.61 - 1065.47) | 34518.14 (29747.76 - 39514.4) | 726.93 (497.12 - 997.75) | -0.23 (-0.24 - -0.22) | 90024.14 (77565.2 - 102578.95) | 4622.45 (3620.94 - 5780.75) | 201920.35 (172997.2 - 230444.91) | 4298.91 (3347.71 - 5378.61) | -0.24 (-0.25 - -0.23) | 2601.24 (637.48 - 6975.52) | 155.98 (36.77 - 429.22) | 6042.8 (1447.7 - 16332.24) | 153.97 (35.75 - 427.12) | 0.01 (-0.02 - 0.05) | 54030.43 (26297.17 - 117956.71) | 2897.52 (1345.27 - 6419.97) | 124502.25 (58055.77 - 274747.38) | 2789.23 (1246.78 - 6275.82) | -0.09 (-0.11 - -0.07) |
| Mozambique | 3926.99 (3324.91 - 4527.09) | 608.66 (414.34 - 836.81) | 6726.63 (5804.38 - 7757.86) | 589.78 (403.49 - 803.77) | -0.08 (-0.1 - -0.06) | 23131 (19605.4 - 26434.34) | 3571.23 (2786.85 - 4478.31) | 39682.4 (33813.2 - 45433.49) | 3459.53 (2702.51 - 4332.62) | -0.08 (-0.1 - -0.06) | 711.41 (176.4 - 1965.95) | 158.29 (37.31 - 439.37) | 1419.11 (340.65 - 3959.9) | 175.37 (39.71 - 503.82) | 0.49 (0.44 - 0.54) | 15352.73 (7023.82 - 34618.12) | 2657.62 (1130.51 - 6157) | 29323.34 (12704.66 - 69094.31) | 2853.29 (1158.05 - 6947.89) | 0.35 (0.31 - 0.38) |
| Myanmar | 18187.65 (15621.81 - 21017.71) | 688.81 (469.19 - 944.83) | 40746.4 (35184.79 - 46755.48) | 636.65 (429.05 - 876.65) | -0.27 (-0.29 - -0.26) | 109333.56 (93546.31 - 125405.71) | 4120.19 (3235.29 - 5175.53) | 243890.28 (208667.86 - 279581.13) | 3790.01 (2959.6 - 4768.43) | -0.28 (-0.3 - -0.27) | 2156.11 (504.18 - 5858.17) | 112.16 (25.23 - 323.32) | 6793.14 (1552.14 - 19449.61) | 126.57 (28.7 - 364.51) | 0.36 (0.32 - 0.41) | 55818.49 (27901.12 - 120815.15) | 2260.55 (1072.41 - 4968.98) | 144748.69 (67194.27 - 321965.9) | 2360.57 (1058.68 - 5401.23) | 0.12 (0.09 - 0.14) |
| Namibia | 417.42 (356.39 - 479.11) | 606.43 (413.22 - 833.79) | 924 (792.46 - 1061.91) | 585.08 (398 - 807.58) | -0.11 (-0.12 - -0.1) | 2437.06 (2053.81 - 2789.79) | 3524.28 (2742.35 - 4410.57) | 5378.66 (4640.88 - 6139.16) | 3391.52 (2644.24 - 4259.19) | -0.12 (-0.13 - -0.11) | 62.27 (14.7 - 177.26) | 127.74 (29.34 - 356.17) | 173.24 (39.41 - 505.31) | 142.88 (31.95 - 413.22) | 0.39 (0.36 - 0.43) | 1498.26 (693.16 - 3318.43) | 2337.83 (1043.28 - 5339.07) | 3723.45 (1686.18 - 8969.5) | 2512.87 (1080.03 - 5980.21) | 0.24 (0.22 - 0.26) |
| Nauru | 2.59 (2.13 - 3.06) | 626.94 (422.47 - 866.25) | 3.74 (3.17 - 4.36) | 645.8 (439.33 - 893.12) | 0.07 (0.02 - 0.11) | 15 (12.45 - 17.61) | 3621.16 (2788.94 - 4574.49) | 21.57 (18.11 - 25.13) | 3739.5 (2892.03 - 4736.74) | 0.08 (0.03 - 0.12) | 0.39 (0.09 - 1.1) | 148.47 (33.82 - 430.29) | 0.59 (0.13 - 1.66) | 148.3 (33.04 - 430.9) | 0.04 (0.01 - 0.06) | 9.68 (4.41 - 22.35) | 2593.39 (1108.62 - 6199.12) | 13.66 (6.27 - 31.23) | 2602.66 (1121.63 - 6103.7) | 0.03 (0.01 - 0.05) |
| Nepal | 5197.22 (4455.5 - 5947.59) | 517.12 (352.23 - 712.18) | 13906.39 (11944.1 - 16048.84) | 460.18 (311.74 - 635.26) | -0.46 (-0.49 - -0.43) | 29840.81 (25712.42 - 34009.69) | 2924.38 (2283.16 - 3655.77) | 79222.61 (67814.67 - 91141.66) | 2595.97 (2017.35 - 3264.66) | -0.47 (-0.5 - -0.44) | 648.54 (151.28 - 1832.98) | 82.29 (18.1 - 237.44) | 2472.62 (565.88 - 6912.48) | 101.96 (22.73 - 292.49) | 0.77 (0.7 - 0.84) | 15779.2 (7661.9 - 33193.97) | 1629 (766.53 - 3642.66) | 51622 (23571.73 - 116396.46) | 1803.26 (772.27 - 4202.09) | 0.34 (0.3 - 0.39) |
| Netherlands | 26284.27 (22900.97 - 29030.1) | 731.11 (534.99 - 944.15) | 47435.77 (41275.96 - 53814.51) | 698.64 (493.01 - 931.62) | -0.13 (-0.16 - -0.11) | 145461.89 (127036.65 - 162128.92) | 4092.21 (3331.54 - 4883.05) | 274552.43 (237829.53 - 311817.72) | 4036.81 (3202.49 - 4960.56) | 0.02 (-0.02 - 0.05) | 5668.71 (1446.76 - 14403.22) | 167.59 (42.03 - 436.34) | 11634.49 (3133.12 - 29250.65) | 164.83 (41.89 - 423.65) | -0.03 (-0.05 - -0.01) | 97898.18 (46495.37 - 207038.75) | 2811.37 (1284.39 - 6063.22) | 191067.71 (89318.45 - 395202.69) | 2773.21 (1258.85 - 5953.4) | -0.03 (-0.03 - -0.02) |
| New Zealand | 4771.9 (4116.24 - 5518.47) | 724.55 (490.15 - 998.92) | 10785.71 (9325.91 - 12424.86) | 684.99 (461.38 - 940.13) | -0.22 (-0.25 - -0.18) | 27055.46 (23044.25 - 31284.39) | 4147.25 (3215.01 - 5226.48) | 61334.79 (52368.49 - 70852.05) | 3903.28 (3015.61 - 4937.14) | -0.24 (-0.29 - -0.19) | 899.77 (226.7 - 2398) | 153.98 (37.58 - 411.2) | 2399.32 (640.19 - 6154.66) | 146.37 (36.98 - 382.65) | -0.18 (-0.23 - -0.14) | 16987.42 (8096.75 - 36420.86) | 2704.29 (1232.43 - 5943.29) | 40882.86 (19423.42 - 84557.01) | 2563.4 (1176.46 - 5529.07) | -0.21 (-0.25 - -0.16) |
| Nicaragua | 1351.55 (1181.49 - 1542.99) | 655.06 (446.82 - 899.1) | 4602.2 (3983.63 - 5301.02) | 650.31 (441.54 - 891.74) | 0 (-0.01 - 0.01) | 7800.06 (6679.92 - 8867.37) | 3783.26 (2957.37 - 4738.27) | 26586.61 (23095.23 - 30520.58) | 3752.11 (2936.04 - 4695.08) | 0 (-0.01 - 0.01) | 201.76 (50.9 - 520.51) | 107.65 (25.91 - 290.84) | 661.83 (163.51 - 1756.87) | 103.45 (24.93 - 277.01) | -0.08 (-0.12 - -0.05) | 4279.07 (2146.39 - 8924.08) | 2119.67 (1026.21 - 4501.63) | 14262.82 (7266.67 - 29341.84) | 2069.33 (1003.39 - 4317.99) | -0.05 (-0.07 - -0.02) |
| Niger | 1268.59 (1087.65 - 1466.45) | 469.41 (319.66 - 650.2) | 3871.96 (3332.53 - 4466.67) | 441.16 (295.64 - 612.05) | -0.2 (-0.21 - -0.2) | 7462.64 (6344.9 - 8535.71) | 2680.59 (2086.89 - 3383.52) | 22647.2 (19291.35 - 25972.12) | 2499.66 (1946.19 - 3146.67) | -0.23 (-0.24 - -0.22) | 209.56 (52.43 - 552.65) | 111.16 (24.94 - 311.53) | 714.95 (164.64 - 1948.03) | 113.82 (25.42 - 324.32) | 0.18 (0.12 - 0.25) | 4815.88 (2181.77 - 10511.46) | 1919.49 (814.53 - 4482.51) | 15597 (7066.87 - 35565.81) | 1919.85 (795.23 - 4570.99) | 0.07 (0.03 - 0.11) |
| Nigeria | 22669.56 (19470.97 - 26050.72) | 423.89 (285.23 - 585.48) | 39973.99 (34340.77 - 45974.24) | 385.3 (259.23 - 532.93) | -0.33 (-0.37 - -0.28) | 129917.32 (111128.91 - 148948.6) | 2363.02 (1833.25 - 3005.25) | 228682.97 (196987.78 - 263673.52) | 2129.93 (1646.74 - 2710.56) | -0.36 (-0.4 - -0.32) | 4279.58 (1060.9 - 11950.98) | 102.26 (23.79 - 289.91) | 9422.3 (2279.45 - 25687.18) | 111.93 (25.56 - 317.24) | 0.43 (0.35 - 0.5) | 86410.25 (38945.96 - 193089.27) | 1719.18 (730.48 - 4045.94) | 176748.53 (75730.95 - 412481.67) | 1791.36 (730.26 - 4355.34) | 0.22 (0.15 - 0.28) |
| Niue | 2.64 (2.25 - 3.1) | 623.81 (420.73 - 864.86) | 2.07 (1.75 - 2.41) | 606.89 (406.02 - 842.54) | -0.11 (-0.12 - -0.1) | 15.34 (12.92 - 17.89) | 3636.05 (2804.62 - 4610.39) | 12.05 (10.09 - 13.94) | 3526.68 (2701.93 - 4477.31) | -0.13 (-0.14 - -0.11) | 0.7 (0.17 - 1.95) | 163.53 (37.46 - 458.4) | 0.45 (0.11 - 1.2) | 146.97 (34.97 - 400.92) | -0.37 (-0.39 - -0.35) | 11.7 (5.2 - 26.67) | 2778.69 (1168.87 - 6501.99) | 8.32 (3.77 - 18.37) | 2519.91 (1099.1 - 5802.1) | -0.33 (-0.35 - -0.32) |
| North Macedonia | 1654.6 (1433.6 - 1887.34) | 633.26 (429.72 - 875.16) | 2887.15 (2450.16 - 3391.43) | 628.96 (427.37 - 863.04) | -0.01 (-0.02 - 0) | 9596.86 (8181.19 - 10981.42) | 3667.89 (2842.17 - 4633.37) | 16575.91 (13912.75 - 19325.16) | 3640.91 (2820.77 - 4601.22) | -0.01 (-0.02 - 0.01) | 275.98 (68.98 - 758.15) | 113.1 (26.49 - 310.83) | 371.29 (86.68 - 1036.59) | 115.87 (26.44 - 329.54) | 0.02 (-0.03 - 0.06) | 5549.22 (2706.34 - 11968.95) | 2152.6 (1015.68 - 4733.65) | 8944.24 (4337.17 - 19129.93) | 2169.51 (1004.87 - 4889.12) | 0 (-0.03 - 0.02) |
| Northern Mariana Islands | 8.24 (6.83 - 9.71) | 619.65 (421.88 - 859.79) | 34.5 (28.71 - 40.16) | 610.3 (408.89 - 842.81) | -0.04 (-0.05 - -0.03) | 48.1 (39.76 - 56.71) | 3609.31 (2761.42 - 4571.66) | 202.04 (169.45 - 234.15) | 3552.55 (2719.31 - 4509.57) | -0.05 (-0.06 - -0.03) | 1.21 (0.29 - 3.47) | 137.41 (31.07 - 385.13) | 5.54 (1.32 - 14.98) | 134.35 (31.77 - 372.54) | -0.05 (-0.11 - 0.01) | 29.32 (13.67 - 65.33) | 2442.05 (1080.09 - 5557.51) | 125.58 (59.7 - 282.24) | 2385.18 (1064.18 - 5439.91) | -0.05 (-0.09 - -0.01) |
| Norway | 10450.31 (9083.63 - 11999.77) | 782.12 (538.08 - 1062.54) | 12869.48 (11097.97 - 14842.76) | 652.83 (441.63 - 900.55) | -0.65 (-0.7 - -0.61) | 58664.21 (50450 - 67430.57) | 4424.29 (3470.82 - 5549.69) | 72425.78 (62194.98 - 83782.12) | 3665.48 (2826.27 - 4648.52) | -0.69 (-0.73 - -0.64) | 2075.16 (524.66 - 5457.73) | 156.54 (38.13 - 419.42) | 3236.36 (859.31 - 8119.4) | 146.67 (36.47 - 381.68) | -0.21 (-0.26 - -0.16) | 36771.74 (17550.55 - 78508.22) | 2781.77 (1295.76 - 6059.45) | 51266.56 (23728.64 - 107926.32) | 2492.02 (1134.58 - 5426.43) | -0.38 (-0.41 - -0.35) |
| Oman | 577.81 (497.48 - 663.46) | 756.39 (517.26 - 1040.77) | 1405.42 (1201.37 - 1630.64) | 707.23 (480.43 - 973.6) | -0.2 (-0.24 - -0.15) | 3389.34 (2893.54 - 3932.21) | 4525.64 (3534.41 - 5678.06) | 8139.23 (6902.48 - 9432.76) | 4182.42 (3226.83 - 5270.11) | -0.24 (-0.29 - -0.19) | 101.16 (24.13 - 273.62) | 166.63 (39.4 - 446.44) | 222.54 (56.24 - 636.61) | 153.96 (38.32 - 426.21) | -0.24 (-0.37 - -0.11) | 2113.47 (1012.53 - 4543.2) | 2985.96 (1348.26 - 6606.47) | 4925.72 (2371.14 - 11314.03) | 2749.4 (1258.87 - 6293.47) | -0.26 (-0.36 - -0.16) |
| Pakistan | 34561.82 (29714.45 - 39922.2) | 476.9 (322.21 - 660.12) | 61259.23 (52577.42 - 70311.72) | 444.76 (299.53 - 611.75) | -0.25 (-0.26 - -0.24) | 193243.4 (165089.39 - 223218.82) | 2645.48 (2060.99 - 3358) | 345824.8 (294155.75 - 399087.72) | 2466.19 (1914.52 - 3133.41) | -0.25 (-0.27 - -0.24) | 5968.72 (1448.19 - 16956.83) | 100.6 (21.92 - 290.64) | 12786.08 (3019.7 - 35544.01) | 115.35 (26.03 - 319.91) | 0.36 (0.3 - 0.42) | 124788.95 (56123.81 - 282275.72) | 1801.86 (779.74 - 4203.88) | 254122.57 (107455.36 - 591105.67) | 1936.7 (787.85 - 4555.51) | 0.17 (0.13 - 0.21) |
| Palau | 7.11 (5.9 - 8.4) | 605.72 (405.73 - 848.36) | 14.49 (12.02 - 16.99) | 587.54 (392.97 - 816.87) | -0.09 (-0.1 - -0.08) | 41.17 (34.12 - 48.22) | 3522.42 (2689.75 - 4470.49) | 84.43 (69.82 - 98.47) | 3396.04 (2589.8 - 4303.53) | -0.11 (-0.12 - -0.1) | 1.22 (0.29 - 3.47) | 145.52 (32.69 - 411.98) | 2.17 (0.5 - 6.16) | 127.75 (28.53 - 371.78) | -0.39 (-0.44 - -0.34) | 26.94 (12.36 - 61.8) | 2527.51 (1076.5 - 5883.92) | 50.88 (23.82 - 114.8) | 2259.73 (981.05 - 5356.45) | -0.33 (-0.37 - -0.3) |
| Palestine | 908.95 (787.3 - 1040.43) | 776.01 (532.3 - 1062.36) | 2206.54 (1907.07 - 2531.21) | 748.23 (512.91 - 1025.34) | -0.13 (-0.16 - -0.1) | 5330.8 (4557.26 - 6089.32) | 4615.64 (3609.27 - 5773.66) | 12889.43 (11090.8 - 14831.06) | 4445.08 (3486.46 - 5564.56) | -0.14 (-0.16 - -0.11) | 151.18 (36 - 420.76) | 157.27 (35.54 - 453.35) | 325.78 (81.07 - 922.75) | 145.51 (33.61 - 410.42) | -0.25 (-0.32 - -0.18) | 3143.97 (1486.81 - 7043.92) | 2878.11 (1313.91 - 6603.81) | 7218.34 (3520.67 - 15802.57) | 2687.98 (1248.87 - 6066.09) | -0.23 (-0.29 - -0.17) |
| Panama | 1391.66 (1208.55 - 1595.78) | 624.6 (425.41 - 859.49) | 4731.93 (4114.78 - 5415) | 615.43 (416.08 - 851.49) | -0.05 (-0.06 - -0.05) | 8003.83 (6893.11 - 9121.48) | 3598.72 (2797.26 - 4519.33) | 27270.94 (23296.49 - 31216.47) | 3550.55 (2772.12 - 4461.2) | -0.05 (-0.05 - -0.04) | 218.59 (54.22 - 585.45) | 103.18 (24.86 - 282.43) | 854.72 (211.58 - 2157.86) | 103.61 (25.39 - 269.29) | 0.04 (0.01 - 0.07) | 4458.19 (2210.95 - 9554.19) | 2028.74 (971.66 - 4347.6) | 15875.85 (7788.86 - 31843.14) | 2021.95 (967.9 - 4203.85) | 0 (-0.02 - 0.02) |
| Papua New Guinea | 1095.16 (922.24 - 1278.69) | 679.57 (462.25 - 935.56) | 3093.13 (2622.49 - 3578.34) | 638.91 (432.54 - 878.54) | -0.25 (-0.28 - -0.22) | 6400.14 (5420.61 - 7439.91) | 3972.64 (3096.77 - 4998.92) | 18037.75 (15285.32 - 20810.93) | 3719.13 (2897.72 - 4672.65) | -0.27 (-0.3 - -0.24) | 132.29 (30.7 - 376.33) | 125.08 (27.59 - 354.81) | 427.98 (99.37 - 1227.06) | 120.98 (27.14 - 356.94) | -0.15 (-0.17 - -0.13) | 3602.22 (1727.48 - 7782.41) | 2396.79 (1094.83 - 5395.46) | 10393.06 (4936.88 - 23301.95) | 2278.54 (1021.35 - 5289.84) | -0.21 (-0.24 - -0.19) |
| Paraguay | 2289.51 (1974.26 - 2610.5) | 696.39 (471.35 - 957.91) | 5789.76 (5050.02 - 6624.75) | 653.47 (443.79 - 897.42) | -0.23 (-0.24 - -0.22) | 13714.86 (11830.26 - 15693.47) | 4178.84 (3274.75 - 5248.02) | 34822.96 (29943.69 - 39565.1) | 3929.45 (3087.22 - 4918.64) | -0.23 (-0.24 - -0.22) | 458.23 (111.47 - 1182.07) | 146.85 (35.27 - 395.96) | 1322.37 (333.29 - 3358.22) | 150.93 (36.23 - 394.5) | 0.14 (0.1 - 0.17) | 8807.35 (4174.65 - 18649.1) | 2717.25 (1240.93 - 5944.42) | 24006.57 (11007.47 - 50574.78) | 2706.33 (1204.5 - 5914.01) | 0 (-0.02 - 0.01) |
| Peru | 7731.21 (6686.76 - 8827.19) | 444.04 (298.8 - 611.21) | 24477.66 (21035.44 - 28033.74) | 440.4 (300.36 - 605.05) | -0.09 (-0.11 - -0.06) | 43893.5 (37896.84 - 50155.74) | 2506.27 (1968.48 - 3142.09) | 137764.01 (118739.41 - 158957.17) | 2477.54 (1934.21 - 3102.77) | -0.09 (-0.12 - -0.07) | 1427.75 (343.92 - 3895.81) | 84.82 (19.51 - 239.96) | 4762.03 (1216.73 - 12584.35) | 81.77 (19.09 - 219.5) | -0.17 (-0.21 - -0.13) | 27588.59 (12788.87 - 60705.5) | 1588.81 (719.85 - 3587.73) | 87661.37 (42097.6 - 187611.33) | 1550.9 (717 - 3360.14) | -0.14 (-0.17 - -0.11) |
| Philippines | 23280.54 (19972.87 - 26990.18) | 669.01 (456.38 - 918.02) | 65982.14 (57345.25 - 75801.92) | 640.33 (435.44 - 879.77) | -0.18 (-0.2 - -0.16) | 138134.38 (118260.24 - 160002.63) | 3939.53 (3077.12 - 4965.92) | 392596.96 (338787.95 - 452741.37) | 3774.4 (2940.37 - 4769.87) | -0.17 (-0.2 - -0.15) | 2926.29 (710.59 - 8248.64) | 117.84 (26.9 - 338.06) | 10936.17 (2622.8 - 27501.11) | 126.49 (29.61 - 348.17) | 0.29 (0.26 - 0.32) | 72824.76 (36021.53 - 158345.2) | 2278.51 (1063.98 - 5143.36) | 235978.54 (110793.01 - 492029.72) | 2375.2 (1079.47 - 5262.16) | 0.15 (0.14 - 0.17) |
| Poland | 46795.14 (40114.7 - 54486.52) | 683.06 (463.69 - 936.95) | 85985.65 (74200.64 - 98708.59) | 646.47 (437.33 - 886.62) | -0.18 (-0.2 - -0.17) | 266250.03 (227069.73 - 309037.17) | 3904.45 (3025.03 - 4934.1) | 490468.54 (421195.36 - 564576.27) | 3683.44 (2850.94 - 4664.09) | -0.19 (-0.2 - -0.18) | 7367.36 (1806.5 - 20694.58) | 126.48 (29.8 - 352.9) | 17121.55 (4346.29 - 44529.46) | 124.15 (30 - 337.24) | -0.05 (-0.07 - -0.03) | 153277.34 (73627.11 - 338734.94) | 2357.53 (1094.82 - 5278.29) | 308882.26 (147218.79 - 658842.78) | 2289.8 (1060.25 - 5056.85) | -0.09 (-0.1 - -0.08) |
| Portugal | 15333.05 (13164.69 - 17972.95) | 678.97 (458.74 - 939.25) | 35089.79 (30142.41 - 40828.61) | 669.54 (454.37 - 918.11) | -0.02 (-0.04 - 0) | 85458.04 (72564.46 - 99101.27) | 3866.59 (2995.62 - 4884.86) | 200316.38 (168823.95 - 233166.28) | 3792.84 (2949.63 - 4773.55) | -0.04 (-0.06 - -0.02) | 2706.31 (650.83 - 7656.7) | 151.25 (35.28 - 419.67) | 8544.71 (2248.96 - 21365.18) | 146.19 (36.43 - 380.5) | -0.09 (-0.12 - -0.07) | 53002.65 (25166.45 - 118398.83) | 2589.32 (1155.44 - 5839.06) | 137959.87 (65557.37 - 288656.12) | 2522.7 (1159.11 - 5415.17) | -0.07 (-0.09 - -0.04) |
| Puerto Rico | 3361.35 (2863.19 - 3892.2) | 565.76 (384.97 - 778.52) | 8604.57 (7509.21 - 9825.44) | 554.14 (376.26 - 765.14) | -0.08 (-0.09 - -0.07) | 19571.3 (16682.21 - 22492.07) | 3296.14 (2583.47 - 4123.31) | 49757.37 (42724.34 - 57221.36) | 3223.97 (2519.19 - 4072.14) | -0.08 (-0.09 - -0.07) | 507.81 (122.07 - 1447.56) | 99.39 (23.07 - 281.53) | 1749.12 (440.43 - 4362.6) | 93.98 (22.84 - 250.67) | -0.18 (-0.2 - -0.16) | 10906.23 (5326.43 - 23684.63) | 1930.58 (903.79 - 4268.63) | 29993.51 (14726.18 - 61703.49) | 1846.03 (886.91 - 3879.48) | -0.16 (-0.17 - -0.14) |
| Qatar | 58.65 (49.82 - 67.49) | 736.31 (504.41 - 1005.58) | 471.29 (399.48 - 545.26) | 720.16 (487.41 - 983.53) | -0.01 (-0.05 - 0.03) | 341.72 (292.2 - 393.12) | 4373.63 (3423.9 - 5488.28) | 2733.45 (2324.63 - 3140.95) | 4256.33 (3298.42 - 5356.96) | -0.02 (-0.06 - 0.03) | 9.18 (2.17 - 25.32) | 176.76 (40.69 - 496.04) | 61 (14.85 - 170.22) | 156.51 (36.75 - 439.58) | -0.41 (-0.48 - -0.34) | 209.09 (98.06 - 466.81) | 3021.36 (1325.58 - 6968.34) | 1510.68 (722.36 - 3306.53) | 2726.85 (1214.41 - 6211.68) | -0.31 (-0.36 - -0.26) |
| Republic of Korea | 24463.41 (21043.04 - 27924.87) | 725.27 (510.33 - 971.67) | 113295.64 (98647.85 - 128792.76) | 707.49 (494.15 - 955.09) | 0.01 (-0.04 - 0.07) | 140195.88 (120671.28 - 158277.26) | 4307.52 (3417.85 - 5269.3) | 677527.26 (588999.55 - 769255.83) | 4242.29 (3373.46 - 5217.28) | 0.01 (-0.05 - 0.06) | 4879.8 (1216.86 - 12788.9) | 210.46 (51.53 - 564.02) | 25773.58 (6890.59 - 62462.41) | 171.09 (44.4 - 420.02) | -0.53 (-0.6 - -0.46) | 98129.63 (44875.79 - 216907.67) | 3384.73 (1444.6 - 7682.82) | 454889.65 (214068.26 - 917812.48) | 2911.27 (1332.63 - 6033.94) | -0.36 (-0.41 - -0.31) |
| Republic of Moldova | 3656.15 (3112.48 - 4238.48) | 627.23 (426.85 - 865.05) | 6583.04 (5674.94 - 7482) | 634.76 (432.58 - 866.17) | 0.03 (0.01 - 0.05) | 21074.34 (18014.53 - 24198.75) | 3631.03 (2834.96 - 4570.58) | 38138.63 (32459.28 - 43640.85) | 3668.87 (2861.54 - 4598.89) | 0.03 (0.01 - 0.05) | 535.22 (128.12 - 1493.37) | 122.33 (28.39 - 346.93) | 1192.95 (296.65 - 3177.64) | 112 (26.57 - 301.8) | -0.29 (-0.32 - -0.26) | 11998.93 (5730.21 - 26195.76) | 2251.89 (1027.95 - 5091.15) | 22724.72 (11251.72 - 48658.24) | 2161.35 (1028.54 - 4650.69) | -0.14 (-0.17 - -0.11) |
| Romania | 25222.54 (21470.79 - 29524.08) | 631.9 (428.94 - 869.97) | 44776.45 (38283.11 - 51956.5) | 630.46 (430.15 - 866.75) | -0.04 (-0.05 - -0.02) | 145006.01 (123071.07 - 168109.74) | 3655.17 (2846.47 - 4613.11) | 258794.74 (220488.54 - 298814.83) | 3646.73 (2838.14 - 4590.05) | -0.04 (-0.05 - -0.02) | 3562.02 (855.34 - 10214.95) | 118.82 (27.42 - 340.26) | 8508.83 (2052.03 - 22981.02) | 118.13 (27.49 - 330.61) | -0.03 (-0.04 - -0.02) | 81036.49 (39498.28 - 181039.16) | 2222.49 (1034.19 - 5018.66) | 158153.44 (76732.79 - 335679.82) | 2221.77 (1033.83 - 4909.41) | -0.02 (-0.03 - -0.01) |
| Russian Federation | 174746.95 (150520.71 - 202881.01) | 662.87 (451.19 - 909.07) | 270748.24 (234046.97 - 311674.49) | 655.92 (447.31 - 898.91) | -0.08 (-0.13 - -0.03) | 1002507.49 (858845.14 - 1161488.38) | 3828.01 (2976.77 - 4833.09) | 1564471.03 (1345566.62 - 1804113.51) | 3777.25 (2948.26 - 4765.2) | -0.09 (-0.14 - -0.03) | 26729.61 (6558.75 - 75293.08) | 127.36 (29.59 - 357.76) | 50769.6 (12543.54 - 141466.56) | 124.02 (29.34 - 340.99) | -0.09 (-0.11 - -0.08) | 575680.09 (278525.57 - 1269366.68) | 2357 (1094.63 - 5301.52) | 958024.27 (452118.71 - 2065617.13) | 2308.31 (1076.63 - 5160.68) | -0.08 (-0.1 - -0.06) |
| Rwanda | 1762.72 (1499.45 - 2032.3) | 605.44 (412.78 - 834.14) | 4189.93 (3625.1 - 4794.24) | 602.26 (411.27 - 824.64) | 0.04 (0.02 - 0.06) | 10372.2 (8838.14 - 11906.47) | 3545.93 (2776.71 - 4450.31) | 24658.66 (21098.58 - 28060.09) | 3521.12 (2758.13 - 4402.65) | 0.04 (0.02 - 0.06) | 287.38 (70.1 - 803.35) | 147.32 (34.03 - 420.21) | 879.77 (215.51 - 2371.3) | 173.21 (40.68 - 485.32) | 0.56 (0.54 - 0.59) | 6683.67 (3137.08 - 14989.21) | 2551.15 (1104.46 - 6004.42) | 18188.16 (7998.72 - 41571.86) | 2865.74 (1174.01 - 6883.02) | 0.41 (0.39 - 0.43) |
| Saint Kitts and Nevis | 33.95 (28.66 - 40.22) | 534.32 (363.07 - 743.01) | 45.16 (38.76 - 52.02) | 529.04 (358.64 - 728.65) | -0.04 (-0.05 - -0.03) | 194.44 (163.27 - 226.22) | 3090.97 (2398.61 - 3895.76) | 261.58 (222.66 - 300.01) | 3054.2 (2375.13 - 3847.98) | -0.05 (-0.06 - -0.04) | 4.56 (1.07 - 13.29) | 95.1 (21.31 - 278.66) | 6.55 (1.57 - 18.1) | 91.37 (20.89 - 257.28) | 0.04 (-0.03 - 0.1) | 104.88 (52.35 - 232.65) | 1822.29 (849.92 - 4112.45) | 148.57 (71.4 - 316.27) | 1783.59 (840.23 - 3947.07) | 0.02 (-0.02 - 0.06) |
| Saint Lucia | 74.13 (62.9 - 86.74) | 560.04 (379.04 - 775.6) | 212.38 (181.79 - 244.77) | 543.76 (370.67 - 752.07) | -0.12 (-0.15 - -0.1) | 428.27 (363.96 - 496.09) | 3253.78 (2525.78 - 4089.22) | 1234.26 (1052.46 - 1417.54) | 3155.46 (2456.85 - 3973.68) | -0.13 (-0.15 - -0.1) | 10.21 (2.42 - 29.22) | 97.93 (22.01 - 283.13) | 36.02 (8.59 - 99.4) | 96.56 (22.43 - 268.96) | -0.14 (-0.18 - -0.11) | 231.04 (113.06 - 509.77) | 1886.01 (876.76 - 4227.78) | 710.69 (341.21 - 1506.14) | 1845.14 (869.89 - 4059.26) | -0.14 (-0.16 - -0.11) |
| Saint Vincent and the Grenadines | 64.67 (54.94 - 74.88) | 575.87 (391.14 - 789.55) | 122.02 (105.61 - 140.44) | 547.01 (370.91 - 755.95) | -0.17 (-0.17 - -0.16) | 373.25 (317.99 - 430.27) | 3339.71 (2606.28 - 4188.66) | 708.61 (607.02 - 814.7) | 3169.31 (2471.96 - 3978.93) | -0.17 (-0.17 - -0.16) | 9.58 (2.33 - 26.47) | 104.76 (23.85 - 296.26) | 18.1 (4.35 - 50.27) | 93.33 (21.02 - 263.77) | -0.23 (-0.31 - -0.14) | 208.6 (101.58 - 443.23) | 1993.2 (924.47 - 4456.85) | 389.57 (190.99 - 837.34) | 1817.22 (864.35 - 4008.97) | -0.22 (-0.27 - -0.17) |
| Samoa | 65.64 (55.92 - 76.39) | 630.92 (425.4 - 873.28) | 116.41 (99.43 - 135.09) | 613.12 (408.74 - 847.39) | -0.11 (-0.12 - -0.1) | 383.66 (323.67 - 443.49) | 3685.81 (2847.81 - 4651.65) | 676.37 (570.89 - 781.17) | 3563.22 (2742.56 - 4511.71) | -0.13 (-0.14 - -0.12) | 13.84 (3.34 - 37.53) | 163.33 (37.21 - 447.85) | 24.84 (6.06 - 69.66) | 155.96 (36.22 - 440.67) | -0.15 (-0.21 - -0.09) | 274.83 (123.64 - 616.91) | 2783.37 (1191.97 - 6457.27) | 478.5 (212.9 - 1099.93) | 2653.75 (1120.91 - 6221.94) | -0.15 (-0.19 - -0.12) |
| San Marino | 46.13 (40.15 - 52.9) | 711.2 (486.77 - 973.38) | 104.14 (89.97 - 120.28) | 635.49 (428.68 - 876.52) | -0.37 (-0.4 - -0.35) | 261.74 (224.92 - 301.96) | 4054.35 (3169.42 - 5070.72) | 595.59 (509.31 - 688.35) | 3585.5 (2775.03 - 4523.51) | -0.41 (-0.43 - -0.38) | 9 (2.32 - 23.14) | 139.52 (34.05 - 367.31) | 26.79 (7.11 - 63.92) | 126.75 (32.66 - 319.28) | -0.2 (-0.27 - -0.13) | 160.28 (78.58 - 329.11) | 2487.29 (1165.72 - 5303.57) | 407.38 (197.91 - 825.84) | 2240.81 (1063.07 - 4616.75) | -0.28 (-0.32 - -0.24) |
| Sao Tome and Principe | 39.86 (34.04 - 45.85) | 443.33 (298.63 - 615.95) | 54.39 (46.43 - 63.29) | 413.93 (276.58 - 573.28) | -0.21 (-0.23 - -0.19) | 229.44 (197.31 - 262.53) | 2521.86 (1959.37 - 3174.7) | 313.39 (264.49 - 360.8) | 2339.45 (1812.88 - 2967.4) | -0.24 (-0.26 - -0.21) | 8.1 (1.89 - 22.86) | 104.89 (23.73 - 296.82) | 11.26 (2.79 - 30.51) | 104.09 (24.15 - 295.21) | -0.01 (-0.07 - 0.05) | 156.32 (70.21 - 358.6) | 1803.71 (781.19 - 4243.83) | 222.88 (98.99 - 507.41) | 1770.14 (746.66 - 4182.45) | -0.05 (-0.09 - -0.01) |
| Saudi Arabia | 4936.38 (4264.39 - 5679.74) | 724.19 (493.02 - 1002.57) | 10909.07 (9270.8 - 12654.97) | 684.78 (463.98 - 944.97) | -0.19 (-0.2 - -0.17) | 28827.86 (24518.48 - 33235.79) | 4294.22 (3335.56 - 5410.21) | 62969.13 (53489.74 - 72255.41) | 4029.86 (3125.32 - 5108.08) | -0.2 (-0.22 - -0.19) | 925.32 (221.24 - 2442.16) | 167.92 (39.04 - 462.67) | 1657.93 (395.51 - 4618.78) | 152.34 (35.64 - 427.63) | -0.36 (-0.39 - -0.33) | 18447.03 (8643.13 - 39277.38) | 2918.58 (1284.93 - 6594.66) | 37710.65 (17267.17 - 81835.46) | 2657.99 (1167.36 - 6102.58) | -0.33 (-0.35 - -0.31) |
| Senegal | 1800.78 (1538.79 - 2066.59) | 463.49 (314.9 - 639.72) | 4140.36 (3567.94 - 4754.85) | 431.77 (289.21 - 600.87) | -0.26 (-0.27 - -0.25) | 10433.49 (8899.73 - 11975.42) | 2631.69 (2057.48 - 3310.81) | 23899.62 (20390.51 - 27363.52) | 2445.36 (1900.28 - 3089.57) | -0.27 (-0.28 - -0.25) | 338.48 (83.67 - 944.96) | 111.36 (24.86 - 314.21) | 962.47 (216.15 - 2680.94) | 122.79 (26.64 - 351.58) | 0.3 (0.27 - 0.32) | 7034.73 (3162.05 - 15909.3) | 1915.78 (805.1 - 4534.14) | 18498.7 (7953.2 - 43235.26) | 2025.65 (817.56 - 4931.15) | 0.16 (0.14 - 0.19) |
| Serbia | 9092.13 (7698.01 - 10541.99) | 629.51 (428.54 - 869.54) | 19009.06 (16358.57 - 22000.58) | 632.09 (428.83 - 870.16) | 0 (-0.02 - 0.01) | 53083.87 (44910.69 - 61132.39) | 3642.06 (2826.84 - 4588.91) | 109348.62 (93116.62 - 125882.93) | 3652.72 (2829.47 - 4606.31) | -0.01 (-0.03 - 0) | 1415.52 (330.21 - 4060.99) | 119.65 (26.98 - 346.34) | 3466.17 (853.14 - 9293.07) | 117.29 (27.01 - 330.48) | -0.16 (-0.19 - -0.12) | 30842.07 (14719.95 - 67519.24) | 2224.45 (1022.06 - 5085.58) | 65545.67 (31318.08 - 140769.12) | 2198.59 (1019.05 - 4872.77) | -0.1 (-0.13 - -0.07) |
| Seychelles | 60.09 (51.82 - 69.07) | 636.87 (429.67 - 883.18) | 100.6 (86.1 - 116.34) | 614.88 (414.39 - 851.06) | -0.14 (-0.14 - -0.13) | 360.01 (306.8 - 414.86) | 3815.15 (2953.24 - 4796.37) | 603.95 (513.56 - 699.22) | 3675 (2841.74 - 4637.8) | -0.14 (-0.15 - -0.13) | 11.78 (2.92 - 32.83) | 129.59 (30.16 - 362.67) | 17.81 (4.26 - 48.57) | 122.24 (27.97 - 338.74) | -0.15 (-0.18 - -0.12) | 225.7 (105.45 - 495.73) | 2419.28 (1109.16 - 5428.81) | 359.82 (170.25 - 775.29) | 2264.8 (1024.05 - 5005.84) | -0.2 (-0.22 - -0.18) |
| Sierra Leone | 1270.21 (1078.37 - 1458.8) | 472.89 (319.22 - 652.05) | 2002.65 (1713.53 - 2319.72) | 446.55 (301.92 - 618.03) | -0.18 (-0.2 - -0.17) | 7293.68 (6212.85 - 8396.19) | 2676.82 (2089.91 - 3368.11) | 11527.11 (9843.88 - 13205.23) | 2521.32 (1968.5 - 3188.92) | -0.19 (-0.2 - -0.18) | 212.38 (49.35 - 590.6) | 99.82 (21.98 - 289.33) | 356.56 (84.38 - 1001.74) | 100.59 (22.77 - 292.33) | 0.02 (0 - 0.05) | 4461.69 (2036.62 - 10028.85) | 1766.67 (779.41 - 4129.92) | 7432.01 (3364.7 - 17300.95) | 1756.79 (752.91 - 4187.58) | -0.01 (-0.02 - 0.01) |
| Singapore | 1461.58 (1260.19 - 1647.12) | 519.94 (377.52 - 675.46) | 7466.7 (6705.24 - 8253.22) | 540.6 (398.95 - 696.88) | 0.16 (0.14 - 0.19) | 8301.99 (7092.63 - 9458.92) | 2954.81 (2363.27 - 3589.13) | 42444.47 (37729.67 - 46812.31) | 3066.96 (2507.94 - 3682.01) | 0.12 (0.08 - 0.16) | 249.74 (62.01 - 659.53) | 110.73 (26.54 - 298.49) | 1453.92 (389.76 - 3558.09) | 105.48 (27.03 - 269.51) | -0.07 (-0.11 - -0.04) | 5066.68 (2444.56 - 11046.84) | 1942.88 (887.85 - 4339.03) | 26381.62 (12900.36 - 53963.64) | 1904.92 (914.82 - 3974.87) | 0 (-0.02 - 0.02) |
| Slovakia | 6134.55 (5259.29 - 7115.98) | 638.36 (433.36 - 880.93) | 10299.71 (8942.74 - 11835.41) | 627.78 (428.6 - 862.13) | -0.05 (-0.07 - -0.04) | 35437.52 (30072.92 - 40593.05) | 3700.91 (2879.02 - 4668.7) | 59475.05 (50926.13 - 69032.49) | 3633.16 (2836.29 - 4572.64) | -0.06 (-0.07 - -0.05) | 1032.98 (248.16 - 2897.13) | 123.15 (28.62 - 342.02) | 1922.57 (485.02 - 5050.22) | 121.78 (28.83 - 329.27) | 0.02 (-0.02 - 0.05) | 20922.59 (10091.76 - 45466.14) | 2277.61 (1050.33 - 5101.08) | 36392.3 (17482.87 - 76261.4) | 2244.59 (1035.77 - 4893.56) | -0.02 (-0.04 - 0) |
| Slovenia | 2559.34 (2192.6 - 2963.19) | 623.41 (420.98 - 862.74) | 5596.77 (4852.39 - 6435.88) | 625.43 (425.92 - 860.31) | 0.05 (0.02 - 0.08) | 14874.2 (12659.29 - 17199.53) | 3628.59 (2806.94 - 4570.8) | 32575.04 (27797.87 - 37712.35) | 3635.89 (2830.44 - 4565.21) | 0.05 (0.02 - 0.08) | 445.8 (109.89 - 1212.55) | 118.79 (27.82 - 325.78) | 1218.17 (306.9 - 3071.28) | 122.62 (29.95 - 326.81) | 0.12 (0.05 - 0.19) | 8819.97 (4137.86 - 19455.74) | 2217.74 (1026.75 - 4927.83) | 20770.37 (10044.32 - 42581.29) | 2240.99 (1047.13 - 4785.26) | 0.06 (0 - 0.11) |
| Solomon Islands | 80.8 (67.58 - 94.65) | 642.9 (432.74 - 887.64) | 229.52 (194.84 - 267.34) | 644.31 (434.86 - 888.85) | 0.05 (0.03 - 0.07) | 471.33 (397.01 - 549.15) | 3748.2 (2912.28 - 4735.4) | 1328.42 (1121.43 - 1540.8) | 3737.88 (2896.66 - 4715.92) | 0.04 (0.01 - 0.06) | 11.39 (2.58 - 33.92) | 138.06 (30.48 - 407.44) | 36.28 (8.22 - 102.68) | 139 (31.16 - 400.68) | 0.02 (-0.01 - 0.04) | 288.47 (134.53 - 677.57) | 2502.72 (1096.23 - 5923.46) | 824.22 (381.77 - 1877.69) | 2499.97 (1092.72 - 5853.83) | 0.01 (-0.03 - 0.05) |
| Somalia | 1267.84 (1093.47 - 1452.13) | 609.42 (414.53 - 836.87) | 3105.24 (2595.54 - 3604.87) | 603.62 (411.9 - 828.74) | -0.01 (-0.02 - -0.01) | 7458 (6358.37 - 8471.93) | 3563.48 (2800.88 - 4463.75) | 18337.59 (15515.81 - 21104.23) | 3528.66 (2768.66 - 4424.85) | -0.01 (-0.02 - 0) | 196.48 (45.07 - 540.1) | 133.89 (29.85 - 382.57) | 477.4 (109.37 - 1358.59) | 144.85 (32.48 - 423.89) | 0.44 (0.38 - 0.51) | 4623.08 (2148.48 - 10109.98) | 2414.68 (1049.71 - 5644.81) | 12203.2 (5440.43 - 27661.79) | 2586.06 (1107.46 - 6170.68) | 0.34 (0.29 - 0.39) |
| South Africa | 18159.57 (15591.52 - 20736.14) | 644.89 (440.45 - 884.52) | 36897.72 (31807.67 - 42343.2) | 607.62 (413.66 - 836.56) | -0.16 (-0.18 - -0.15) | 104053.48 (89492.61 - 119618.54) | 3680.82 (2864.31 - 4654.31) | 211927.67 (181581.56 - 243821.29) | 3464.77 (2695.88 - 4386.3) | -0.17 (-0.18 - -0.15) | 3097.81 (754.58 - 8403.58) | 124.65 (28.6 - 344.53) | 6613.16 (1603.86 - 18543.19) | 132.99 (31.18 - 371.64) | 0.18 (0.09 - 0.26) | 62781.61 (29834.35 - 136074.12) | 2300.31 (1058.13 - 5148.85) | 135783.66 (62561.04 - 306535.03) | 2357.71 (1042.29 - 5431.99) | 0.07 (0.01 - 0.13) |
| South Sudan | 1830.97 (1564.52 - 2115.13) | 595.75 (407.01 - 821.34) | 2246.99 (1927.28 - 2576.92) | 558.47 (380.21 - 767.52) | -0.19 (-0.22 - -0.16) | 10855.02 (9279 - 12517.49) | 3518.2 (2751.63 - 4419.34) | 13270.07 (11314.15 - 15239.24) | 3262.53 (2542.62 - 4102.61) | -0.23 (-0.26 - -0.2) | 310.99 (72.16 - 830.44) | 136.12 (29.87 - 381.98) | 434.39 (101.44 - 1173.02) | 138.78 (30.05 - 386.38) | 0.05 (0 - 0.09) | 6807.64 (3092.29 - 15000.1) | 2390.7 (1018.6 - 5564.96) | 9071.21 (3983.17 - 20625.05) | 2398.2 (996.91 - 5571.93) | 0 (-0.04 - 0.05) |
| Spain | 63664.8 (55522.59 - 71161.01) | 670.33 (506.01 - 850.39) | 121203.8 (104789.06 - 139260.08) | 584.76 (404.39 - 793.71) | -0.38 (-0.45 - -0.3) | 366152.77 (317527.97 - 406017.88) | 3893.85 (3224.33 - 4610.91) | 701126.5 (605303.25 - 799270.88) | 3338.63 (2637.61 - 4149.49) | -0.38 (-0.45 - -0.31) | 13299.32 (3279.24 - 34758.92) | 156.6 (39.04 - 418.86) | 36099.6 (9659.12 - 87847.45) | 146.6 (38.06 - 370.76) | -0.17 (-0.19 - -0.14) | 243234.24 (112636.97 - 511544.87) | 2684.85 (1202.84 - 5912.63) | 543185.81 (248820.3 - 1116719.17) | 2436.68 (1100.09 - 5184.36) | -0.25 (-0.28 - -0.22) |
| Sri Lanka | 8325.17 (7138.19 - 9618.12) | 624.18 (424.39 - 862.6) | 24346.88 (20886.04 - 28310.78) | 603.26 (408.73 - 836.25) | -0.13 (-0.14 - -0.11) | 50477.23 (43058.15 - 58087.29) | 3753.74 (2938.98 - 4712.44) | 146754.38 (124019.05 - 169947.39) | 3613.97 (2805.17 - 4555.35) | -0.13 (-0.15 - -0.12) | 1129.72 (267.49 - 3144.67) | 111.02 (25.62 - 312.05) | 3803.16 (865.96 - 10126.81) | 111.35 (25.13 - 302.89) | 0.03 (-0.01 - 0.07) | 27049.57 (13433.67 - 59126.07) | 2165.29 (1013.56 - 4787.66) | 82743.65 (39511.36 - 169521.72) | 2134.72 (982.12 - 4641.42) | -0.04 (-0.06 - -0.02) |
| Sudan | 8885.91 (7657.36 - 10263.46) | 766.58 (522.19 - 1047.06) | 17064.38 (14746.67 - 19521.75) | 715.14 (487.35 - 980.2) | -0.23 (-0.24 - -0.22) | 51412.15 (44066.18 - 59316.85) | 4545.58 (3561.76 - 5662.75) | 99164.88 (85118.21 - 113701.73) | 4204.82 (3301.28 - 5286.5) | -0.27 (-0.27 - -0.26) | 1250.97 (301.51 - 3480.59) | 148.64 (34.19 - 423.97) | 2740.04 (658 - 7407.66) | 137.6 (31.13 - 376.65) | -0.27 (-0.29 - -0.24) | 29054.92 (13893.55 - 68051.29) | 2782.36 (1285.5 - 6475.62) | 57939.63 (27742.17 - 123008.65) | 2570.08 (1193.82 - 5658.23) | -0.28 (-0.3 - -0.26) |
| Suriname | 219.26 (190.1 - 249.78) | 595.79 (405.25 - 818.48) | 550.54 (477.42 - 630.36) | 568.19 (388.69 - 776.94) | -0.17 (-0.18 - -0.15) | 1271.69 (1092.1 - 1453.22) | 3454.06 (2699.19 - 4318.14) | 3184.64 (2730.11 - 3635.9) | 3287.32 (2571.56 - 4130.01) | -0.17 (-0.19 - -0.16) | 35.96 (8.89 - 96.94) | 97.62 (22.81 - 272.01) | 89.21 (22.5 - 235.11) | 95.98 (21.74 - 260.69) | -0.04 (-0.08 - 0) | 717.64 (354.7 - 1534.78) | 1935.44 (924.59 - 4209.02) | 1800.6 (898.56 - 3826.25) | 1874.96 (901.14 - 4005.04) | -0.1 (-0.13 - -0.08) |
| Sweden | 23791.1 (20874.75 - 26913.87) | 789.95 (564.96 - 1054.32) | 33010.6 (28581.63 - 37861.14) | 723.54 (495.69 - 987.3) | -0.24 (-0.28 - -0.21) | 128445.41 (110731.41 - 147329.07) | 4346.6 (3451.09 - 5356.75) | 182779.07 (157263.12 - 209711.88) | 4006.42 (3103.57 - 5054.38) | -0.18 (-0.23 - -0.13) | 4190.89 (1064.92 - 10987.85) | 145.37 (35.36 - 387.3) | 6915.03 (1824.08 - 17482.12) | 136.83 (34.46 - 348.95) | -0.12 (-0.18 - -0.06) | 75982.98 (36806.63 - 157010.03) | 2605.03 (1248.25 - 5576.72) | 114512.46 (55143.86 - 235816.38) | 2428.88 (1162.03 - 5023.61) | -0.15 (-0.18 - -0.13) |
| Switzerland | 14202.97 (12237.36 - 16373.56) | 713.21 (487.11 - 974.11) | 24339.82 (21047.34 - 27985.13) | 648.7 (440.45 - 888.81) | -0.34 (-0.36 - -0.31) | 81880.58 (69829.93 - 94683.65) | 4117.98 (3209.42 - 5156.09) | 139426.57 (119157.72 - 161996.14) | 3682.04 (2850.47 - 4636.36) | -0.4 (-0.43 - -0.37) | 2877.02 (731.83 - 7527.97) | 146.7 (35.6 - 394.82) | 6350.97 (1737.37 - 15322.84) | 145.26 (37.77 - 362.43) | -0.04 (-0.14 - 0.06) | 51583.66 (24586.76 - 110056.53) | 2619.49 (1206.11 - 5716.54) | 99017.11 (46862.78 - 201954.23) | 2481.61 (1146.9 - 5198.01) | -0.19 (-0.26 - -0.12) |
| Syrian Arab Republic | 5279.46 (4614.89 - 6012.07) | 793.47 (543.42 - 1087.45) | 12145.69 (10424.51 - 14010.45) | 737.98 (502.94 - 1009.03) | -0.24 (-0.25 - -0.23) | 31079.66 (26768.16 - 35421.63) | 4714.12 (3697.38 - 5915.06) | 70165.33 (59968.2 - 80761.07) | 4328.9 (3380.15 - 5446) | -0.28 (-0.29 - -0.27) | 909.74 (218.5 - 2460.08) | 149.24 (35.04 - 422.78) | 1678.84 (388.43 - 4696.63) | 140.44 (31.71 - 404.78) | -0.21 (-0.24 - -0.18) | 18220.11 (8804.79 - 39288.42) | 2807.1 (1312.06 - 6394.25) | 39205.33 (19125.14 - 84600.35) | 2627.91 (1204.52 - 6011.55) | -0.23 (-0.25 - -0.21) |
| Taiwan (Province of China) | 9693.98 (8244.5 - 11185.04) | 517.37 (356.08 - 696.18) | 41858.04 (35624.51 - 47307.57) | 557.44 (395.01 - 737.48) | 0.39 (0.29 - 0.48) | 54481.9 (46801.14 - 63164.46) | 2905.37 (2273.06 - 3607.94) | 240328.37 (202932.41 - 269954.54) | 3185.2 (2513.11 - 3892.56) | 0.47 (0.35 - 0.58) | 1619.14 (388.25 - 4400.08) | 118.97 (28.32 - 325.19) | 10020.71 (2631.84 - 25090.39) | 124.5 (31.05 - 322.9) | -0.24 (-0.4 - -0.08) | 34695.57 (16134.15 - 77823.64) | 2036.21 (899.2 - 4621.05) | 167347.02 (77876.79 - 356661.02) | 2163.46 (990.38 - 4643.15) | 0 (-0.11 - 0.1) |
| Tajikistan | 2475.35 (2158.38 - 2833.45) | 633.78 (431.76 - 867.45) | 3767.75 (3264.9 - 4321.27) | 593.19 (403.26 - 815.52) | -0.23 (-0.24 - -0.23) | 14314.33 (12348.49 - 16360.99) | 3648.22 (2850.7 - 4574.7) | 21985.23 (18805.11 - 25241.63) | 3409.07 (2657.96 - 4302.82) | -0.24 (-0.25 - -0.23) | 439.53 (105.67 - 1209.57) | 123.19 (28.4 - 335.08) | 664.83 (162.22 - 1772.73) | 127.16 (29.94 - 352.23) | 0.08 (0.04 - 0.12) | 8679.57 (4087.87 - 18827.24) | 2280.04 (1044.02 - 5009.31) | 13906.91 (6408.17 - 30235.72) | 2272.54 (1015.03 - 5170.99) | -0.03 (-0.05 - 0) |
| Thailand | 25144.1 (21562.8 - 28656.79) | 574.08 (394.81 - 787.32) | 107287.86 (93197.96 - 122723.01) | 581.15 (394.02 - 804.3) | 0.12 (0.07 - 0.18) | 152213.51 (131090.43 - 172704.21) | 3423.44 (2707.7 - 4249.32) | 636452.54 (547365.7 - 728131.41) | 3442.75 (2690.06 - 4323.74) | 0.15 (0.08 - 0.23) | 4819.87 (1180.98 - 12721.36) | 130.45 (30.45 - 360) | 23369.49 (6083.77 - 60450.05) | 124.21 (29.96 - 321.24) | -0.34 (-0.41 - -0.28) | 99257.07 (45970.37 - 213383.59) | 2344.58 (1034.05 - 5277.81) | 427148.7 (198917.64 - 904551.87) | 2290.68 (1047.41 - 4961.51) | -0.15 (-0.19 - -0.12) |
| Timor-Leste | 184.98 (159.31 - 211.5) | 677.65 (461.74 - 931.24) | 714.9 (615.51 - 827.36) | 637.2 (434.77 - 878.71) | -0.18 (-0.21 - -0.16) | 1118.44 (965.34 - 1283.68) | 4066 (3197.33 - 5104.29) | 4286.83 (3630.49 - 4948.2) | 3794.97 (2967.48 - 4764.75) | -0.21 (-0.23 - -0.18) | 24.73 (5.9 - 70.21) | 110.36 (24.39 - 317.8) | 104.56 (25.16 - 304.36) | 123.2 (28.06 - 356.37) | 0.42 (0.38 - 0.46) | 585.3 (288.28 - 1263.2) | 2213.38 (1054.58 - 4903.2) | 2420.32 (1148.56 - 5380.92) | 2315.28 (1046.3 - 5344.91) | 0.2 (0.17 - 0.24) |
| Togo | 605.3 (518.7 - 701.88) | 457.44 (308.15 - 631.7) | 1677.4 (1430.82 - 1938.7) | 441.71 (298.31 - 610.73) | -0.09 (-0.1 - -0.08) | 3505.63 (3007.32 - 4018.22) | 2588.88 (2022.13 - 3260.62) | 9783.11 (8294.38 - 11271.68) | 2497.19 (1935.19 - 3152.55) | -0.1 (-0.11 - -0.09) | 109.33 (25.97 - 305.12) | 109.24 (24.14 - 311.96) | 319.43 (72.94 - 904.85) | 118.32 (25.94 - 338.41) | 0.26 (0.22 - 0.3) | 2340.16 (1055.27 - 5094.35) | 1882.78 (799.45 - 4414.17) | 6962.39 (3061.31 - 16670.83) | 1977.21 (818.28 - 4755.81) | 0.17 (0.14 - 0.2) |
| Tokelau | 1.26 (1.06 - 1.47) | 629.69 (424.5 - 873.28) | 1.56 (1.33 - 1.82) | 618.57 (417.58 - 860.29) | -0.06 (-0.07 - -0.05) | 7.31 (6.14 - 8.5) | 3662.32 (2815.04 - 4630.28) | 9.04 (7.61 - 10.52) | 3585.11 (2768.46 - 4543.84) | -0.07 (-0.08 - -0.06) | 0.27 (0.07 - 0.76) | 166.67 (38.61 - 476.89) | 0.36 (0.09 - 0.95) | 149.46 (34.77 - 409.58) | -0.38 (-0.39 - -0.37) | 5.4 (2.39 - 12.11) | 2849.65 (1183.84 - 6871.8) | 6.41 (2.9 - 14.29) | 2589.52 (1124.39 - 5957.28) | -0.32 (-0.33 - -0.32) |
| Tonga | 45.69 (38.99 - 52.87) | 651.29 (442.45 - 901.24) | 79.31 (68.01 - 91.86) | 632.57 (427.93 - 872.86) | -0.11 (-0.12 - -0.09) | 266.87 (227.9 - 308.86) | 3810.23 (2958.22 - 4819.16) | 461.91 (393.72 - 533.54) | 3686.87 (2862.19 - 4636.46) | -0.12 (-0.13 - -0.1) | 8.83 (2.24 - 23.87) | 155.59 (36.59 - 429.42) | 18.14 (4.45 - 47.63) | 152.46 (35.89 - 410.34) | -0.01 (-0.04 - 0.01) | 179.2 (82.92 - 402.46) | 2709.06 (1197.3 - 6234.21) | 322.72 (147.16 - 709.55) | 2622.26 (1154.35 - 5835.05) | -0.07 (-0.08 - -0.06) |
| Trinidad and Tobago | 696.7 (595.8 - 811.56) | 572.08 (391.85 - 791.05) | 1753.43 (1520.9 - 2004.7) | 559.23 (378.38 - 769.22) | -0.06 (-0.07 - -0.05) | 4025.21 (3435.38 - 4662.19) | 3311.31 (2589.2 - 4167.32) | 10096.77 (8650.83 - 11507.64) | 3232.8 (2519.71 - 4061.79) | -0.07 (-0.07 - -0.06) | 90.71 (21.75 - 258.53) | 98.11 (22.21 - 283.18) | 277 (68.15 - 736.35) | 92.66 (21.17 - 257.21) | -0.11 (-0.14 - -0.07) | 2116.19 (1062.36 - 4705.58) | 1896.65 (897.31 - 4245.08) | 5653.24 (2748.51 - 12082.64) | 1824.59 (873.94 - 3973.89) | -0.07 (-0.1 - -0.05) |
| Tunisia | 5167.27 (4440.85 - 5932.82) | 812.58 (554.15 - 1105.57) | 15098.08 (13183.81 - 17262.56) | 763.62 (523.9 - 1037.53) | -0.19 (-0.2 - -0.18) | 30106.24 (25750.26 - 34586.18) | 4864.68 (3816.13 - 6070.84) | 89161.54 (77091.67 - 101690.74) | 4543.11 (3562.16 - 5670.92) | -0.21 (-0.22 - -0.2) | 735.18 (176.42 - 2022.41) | 166.51 (39.12 - 453.51) | 2689.57 (655.01 - 7029.45) | 153.37 (35.78 - 418.59) | -0.28 (-0.3 - -0.26) | 17301.36 (8241.05 - 38096.33) | 3068.86 (1405.86 - 6808.78) | 53736.93 (26010.61 - 113254.77) | 2827.34 (1309.72 - 6206.61) | -0.27 (-0.28 - -0.26) |
| Turkey | 38044.24 (33073.64 - 43320.36) | 838.03 (577.3 - 1136.89) | 112752.31 (97572.15 - 128528.58) | 791.47 (539.56 - 1079.55) | -0.22 (-0.24 - -0.21) | 225384.43 (192933.36 - 256711.23) | 5022.11 (3946.71 - 6271.55) | 665586.45 (576412.22 - 760072.27) | 4708.69 (3677.87 - 5891.13) | -0.25 (-0.28 - -0.23) | 6821.72 (1699.59 - 18021.93) | 177.35 (42.16 - 481.89) | 19915.63 (5030.52 - 52387.58) | 154.24 (36.86 - 426.62) | -0.45 (-0.59 - -0.32) | 137569 (66773.08 - 292036.15) | 3211.66 (1465.5 - 7066.13) | 394366.23 (192372.34 - 827157.73) | 2864.64 (1337.61 - 6354.68) | -0.4 (-0.47 - -0.32) |
| Turkmenistan | 1541.58 (1339.86 - 1764.38) | 637.88 (434.48 - 873.83) | 3063.45 (2651.38 - 3495.71) | 606.33 (411.32 - 836.39) | -0.18 (-0.21 - -0.16) | 8876.99 (7589.44 - 10118.79) | 3670.94 (2858.51 - 4602.2) | 17793.23 (15171.82 - 20260.03) | 3487.36 (2728.27 - 4370.02) | -0.18 (-0.21 - -0.15) | 250.55 (61.27 - 675.81) | 123.73 (29.17 - 343.94) | 563.51 (136.46 - 1570) | 116.11 (26.95 - 323.08) | -0.32 (-0.37 - -0.27) | 5260.51 (2535.58 - 11403.3) | 2291.25 (1055.39 - 5131.56) | 11002.22 (5307.84 - 24480.93) | 2166.25 (1011.17 - 4805.84) | -0.26 (-0.29 - -0.23) |
| Tuvalu | 4.62 (3.87 - 5.43) | 657.59 (446.11 - 909.43) | 8.61 (7.4 - 10.04) | 642.23 (433.26 - 886.26) | -0.07 (-0.08 - -0.06) | 26.83 (22.53 - 31.49) | 3829.63 (2974.54 - 4843.21) | 49.82 (42.43 - 58.16) | 3726.24 (2878.11 - 4695.91) | -0.09 (-0.1 - -0.07) | 0.74 (0.18 - 2.17) | 158.82 (36.62 - 465.94) | 1.56 (0.36 - 4.49) | 152.13 (34.55 - 437.91) | -0.18 (-0.19 - -0.16) | 17.74 (8.01 - 42.23) | 2780.4 (1211.41 - 6702.28) | 32.94 (15.3 - 77.32) | 2650.23 (1156.3 - 6254.74) | -0.17 (-0.18 - -0.17) |
| Uganda | 4407.89 (3766.24 - 5077.39) | 587.74 (399.77 - 809.54) | 9690.74 (8354.28 - 11186.98) | 579.84 (393.89 - 798.97) | -0.05 (-0.07 - -0.02) | 25885.29 (22078.07 - 29745.1) | 3438.09 (2687.37 - 4306.61) | 56981.2 (48686.2 - 65234.7) | 3392.87 (2649.68 - 4254.78) | -0.05 (-0.07 - -0.02) | 700.6 (176.96 - 1940.45) | 129.85 (30 - 354.61) | 2041.34 (496.3 - 5594.34) | 158.37 (37.06 - 438.11) | 0.7 (0.65 - 0.74) | 15590.06 (7344.14 - 33548.41) | 2292.77 (1006.99 - 5126.02) | 40592.56 (18282.51 - 93719.3) | 2638.65 (1102.22 - 6242.24) | 0.49 (0.45 - 0.52) |
| Ukraine | 74640.9 (64019.1 - 86823.82) | 674.16 (456.24 - 929.33) | 90600.67 (77616.44 - 104827.32) | 654.4 (443.89 - 899.79) | -0.11 (-0.16 - -0.07) | 422524.99 (357961.37 - 494958.86) | 3839.08 (2964.21 - 4888.27) | 513894.98 (441453.33 - 597002.74) | 3717.54 (2873.37 - 4710.81) | -0.12 (-0.17 - -0.08) | 11463.89 (2765.23 - 33165.74) | 126.73 (29.22 - 359.87) | 16326.67 (3922.42 - 44642.32) | 123.78 (28.38 - 346.57) | -0.1 (-0.11 - -0.08) | 241294.31 (116131.68 - 530050.38) | 2337.67 (1081.35 - 5264.16) | 313360.09 (142240.61 - 688500.16) | 2299.23 (1045.62 - 5124.06) | -0.07 (-0.09 - -0.05) |
| United Arab Emirates | 250.17 (210.88 - 292.81) | 702.5 (475.97 - 968.29) | 1472.53 (1195.17 - 1786.68) | 644.47 (434.11 - 895.09) | -0.26 (-0.3 - -0.23) | 1447.82 (1210.61 - 1686.69) | 4157.47 (3206.55 - 5242.71) | 8467.79 (7046 - 9955.82) | 3740.38 (2877.39 - 4719.04) | -0.33 (-0.38 - -0.29) | 43.46 (10.64 - 116.64) | 169.99 (40.29 - 460.7) | 144.9 (33.88 - 396.02) | 142.48 (33.25 - 391.9) | -0.37 (-0.51 - -0.24) | 945.55 (437.35 - 2112.79) | 2952.9 (1298.23 - 6686.85) | 4702.81 (2254.27 - 10615.47) | 2486.63 (1101.31 - 5585.47) | -0.42 (-0.52 - -0.33) |
| United Kingdom | 110560.45 (94697.48 - 128085.93) | 656.16 (453.51 - 891.61) | 162210.49 (140307.69 - 187154.63) | 619.67 (425.5 - 841.57) | -0.16 (-0.19 - -0.12) | 596443.07 (510177.32 - 686761.59) | 3593.19 (2801.18 - 4512.64) | 892122.41 (762803.47 - 1022977.42) | 3401.71 (2633.86 - 4280.4) | -0.12 (-0.17 - -0.08) | 21640.36 (5395.01 - 58106.05) | 139.69 (33.52 - 379.43) | 37497.13 (9830.57 - 94799.73) | 133.36 (33.26 - 349.62) | -0.09 (-0.13 - -0.05) | 384841.74 (181093.13 - 827171.14) | 2384.87 (1088.22 - 5302.51) | 610383.99 (285737.88 - 1277571.91) | 2276.66 (1041.96 - 4961.21) | -0.1 (-0.13 - -0.07) |
| United Republic of Tanzania | 7435.81 (6400.84 - 8597.28) | 604.55 (411.41 - 827.7) | 17113.31 (14986.64 - 19259.2) | 555.07 (389.54 - 743.84) | -0.25 (-0.27 - -0.23) | 43777.08 (37483.13 - 50366.39) | 3543.19 (2773.45 - 4428.22) | 100920.08 (88375.41 - 112817.78) | 3250.2 (2612.7 - 3994.71) | -0.26 (-0.27 - -0.24) | 1301.21 (309.74 - 3609.54) | 151.53 (35.6 - 417.19) | 3807.62 (930.74 - 10242.13) | 152.21 (36.32 - 421.84) | 0 (-0.03 - 0.02) | 29107.43 (13365.8 - 65454.36) | 2608.79 (1112.02 - 6024.88) | 74352.53 (32693.48 - 170278.95) | 2560.83 (1068.6 - 6050.74) | -0.06 (-0.08 - -0.05) |
| United States of America | 58.49 (49.97 - 68.29) | 552.44 (374.86 - 764.6) | 176.77 (150.78 - 206.72) | 535.51 (360.79 - 744.58) | -0.11 (-0.12 - -0.11) | 339.42 (287.27 - 393.16) | 3207.6 (2508.38 - 4037.22) | 1021.76 (872.62 - 1199.78) | 3109.62 (2406.33 - 3933.13) | -0.11 (-0.12 - -0.1) | 8.16 (2.02 - 22.35) | 102.65 (23.99 - 295.58) | 25.17 (6.02 - 67) | 89.53 (20.42 - 245.54) | -0.4 (-0.42 - -0.38) | 191.66 (92.87 - 406.58) | 1958.57 (905.01 - 4404.32) | 545.62 (273.57 - 1130.78) | 1753.96 (836.39 - 3767.29) | -0.34 (-0.35 - -0.33) |
| United States Virgin Islands | 454768.05 (394262.87 - 522763.61) | 780.22 (537.76 - 1062.84) | 797565.45 (691509.86 - 909939.56) | 738.41 (506.13 - 1006.58) | -0.19 (-0.2 - -0.18) | 2658192.84 (2285436.82 - 3052804.3) | 4597.26 (3601.18 - 5780.95) | 4752151.97 (4108235.99 - 5457675.13) | 4390.64 (3429.7 - 5509.06) | -0.17 (-0.19 - -0.16) | 99689.03 (25848.43 - 257709.99) | 174.69 (43.41 - 459.84) | 197596.87 (53196.07 - 493203.29) | 171.94 (43.55 - 442.4) | -0.08 (-0.09 - -0.06) | 1764287.3 (828633.6 - 3780953.29) | 3067.64 (1405.98 - 6716.7) | 3278708.72 (1524747.3 - 6891760.75) | 2957.48 (1343.8 - 6417.71) | -0.15 (-0.16 - -0.13) |
| Uruguay | 4275.04 (3687.06 - 4915.65) | 639.02 (436.45 - 871.65) | 6844.87 (5957.21 - 7866.64) | 607.98 (414.01 - 834.4) | -0.21 (-0.23 - -0.19) | 23845.75 (20352.47 - 27419.85) | 3590.86 (2802.34 - 4502.55) | 38327.57 (33131.65 - 44115.47) | 3400.94 (2659.53 - 4289.83) | -0.23 (-0.25 - -0.2) | 740.51 (180.96 - 2052.98) | 122.01 (28.72 - 337.05) | 1569.41 (407.72 - 4041.1) | 124.74 (30.45 - 332.28) | 0.08 (0.06 - 0.09) | 14231.12 (6813.52 - 30652.84) | 2212.44 (1029.16 - 4933.98) | 25843.27 (12252.93 - 55378.71) | 2205.65 (1009.39 - 4817.96) | -0.02 (-0.03 - -0.02) |
| Uzbekistan | 10556.07 (9052.93 - 12252.84) | 604.15 (409.01 - 829.39) | 18504.55 (15920.52 - 21210.06) | 600.92 (404.94 - 825.07) | -0.03 (-0.04 - -0.01) | 61043.86 (51929.12 - 70111.34) | 3478.89 (2712.42 - 4377.67) | 107574.24 (91989.55 - 123043.78) | 3460.64 (2686.78 - 4355.68) | -0.02 (-0.04 - 0) | 1919.79 (479.02 - 5251.05) | 119.48 (28.11 - 328.91) | 2974.06 (728.02 - 8479.61) | 113.76 (26.69 - 318.44) | -0.15 (-0.16 - -0.13) | 37343.95 (17693.08 - 80048.76) | 2197.01 (1007.4 - 4898.1) | 63517.62 (30593.57 - 138333.8) | 2128.62 (981.79 - 4776.47) | -0.1 (-0.11 - -0.09) |
| Vanuatu | 36.29 (30.13 - 42.64) | 607.21 (408.39 - 843.32) | 107.4 (90.52 - 125.54) | 595.63 (400.74 - 824.62) | -0.08 (-0.11 - -0.06) | 211.25 (176.46 - 246.74) | 3532.74 (2700.46 - 4481.22) | 623.08 (526.7 - 728.4) | 3457.29 (2659.26 - 4367.05) | -0.09 (-0.12 - -0.07) | 5.35 (1.19 - 15.89) | 134.61 (29.31 - 388.64) | 17.17 (4.14 - 49.25) | 135.87 (30.58 - 395.46) | -0.01 (-0.03 - 0.01) | 131.07 (59.25 - 303.97) | 2406.9 (1048.14 - 5597.13) | 397.19 (183.5 - 920.62) | 2405.1 (1047.1 - 5739.17) | -0.04 (-0.06 - -0.02) |
| Venezuela (Bolivarian Republic of) | 9560.67 (8259.91 - 10915.32) | 720.11 (494.02 - 982.17) | 32624.99 (28640.86 - 36988.5) | 704.99 (483.56 - 965.64) | -0.08 (-0.12 - -0.04) | 55623.09 (47956.4 - 63628.98) | 4197.31 (3304.12 - 5224.56) | 189949.09 (164242.49 - 215699.94) | 4111.91 (3239.92 - 5131.71) | -0.08 (-0.13 - -0.04) | 1459.64 (361.73 - 3921.32) | 124.84 (29.9 - 342.37) | 5669.36 (1419.19 - 14359.74) | 122.03 (28.99 - 325.41) | -0.06 (-0.09 - -0.03) | 30930.84 (15229.82 - 65150.59) | 2413.06 (1151.41 - 5224.89) | 110617.71 (54790.52 - 230149.14) | 2373.49 (1132 - 5021.11) | -0.06 (-0.08 - -0.05) |
| Viet Nam | 37698.67 (32532.27 - 43236.04) | 656.23 (443.6 - 906.53) | 83780.78 (72555.37 - 95988.98) | 619.7 (421.87 - 856.26) | -0.22 (-0.23 - -0.21) | 226932.58 (193855.92 - 259517.84) | 3938.21 (3083.54 - 4958.36) | 504132 (429558.62 - 576475.39) | 3708.72 (2889.37 - 4678.54) | -0.23 (-0.24 - -0.22) | 7335.8 (1752.62 - 19515.58) | 144.94 (33.03 - 396.82) | 19125.9 (4633.56 - 53502.78) | 160.72 (37.98 - 446.02) | 0.31 (0.27 - 0.36) | 146695.58 (68678.45 - 318200.78) | 2635.3 (1185.41 - 5880.36) | 361830.71 (161802.93 - 820135.72) | 2764.14 (1193.35 - 6412.21) | 0.13 (0.1 - 0.17) |
| Yemen | 4360.83 (3771.23 - 5025.37) | 821.45 (564.33 - 1119.56) | 12347.73 (10629.68 - 14163.38) | 757.38 (517.19 - 1033.97) | -0.27 (-0.3 - -0.24) | 25345.81 (21619.61 - 29191.47) | 4930.14 (3869.2 - 6124.53) | 71522.3 (61567.62 - 81960.16) | 4470.87 (3493.47 - 5593.69) | -0.33 (-0.36 - -0.3) | 601.52 (140.21 - 1660.32) | 161.56 (37.38 - 454.43) | 1913.02 (439.81 - 5469.37) | 153.76 (33.76 - 452.21) | -0.17 (-0.19 - -0.15) | 14438.43 (6953.34 - 31214.11) | 3035.72 (1394.94 - 6941.25) | 42302.4 (20416.78 - 96751.7) | 2829.35 (1290.62 - 6550.83) | -0.25 (-0.27 - -0.22) |
| Zambia | 1795.58 (1541.67 - 2067.82) | 577.48 (391.03 - 796.36) | 4182.97 (3565.48 - 4805.75) | 579.6 (398.42 - 796.2) | 0.06 (0.04 - 0.09) | 10531.25 (8963.61 - 12095.01) | 3363.69 (2635.93 - 4231.22) | 24567.59 (20987.27 - 28085.17) | 3378.25 (2655.4 - 4215.89) | 0.06 (0.04 - 0.09) | 291.17 (70.2 - 802) | 129.38 (29.28 - 365.72) | 760.36 (177.38 - 2183.39) | 144.75 (32.6 - 401.89) | 0.46 (0.4 - 0.52) | 6573.47 (3035.6 - 14597.67) | 2301.08 (1012.07 - 5317.11) | 16586.39 (7624.57 - 38067.09) | 2504.81 (1072.32 - 5808.08) | 0.35 (0.3 - 0.39) |
| Zimbabwe | 2749.37 (2342.31 - 3165.64) | 601.44 (408.74 - 833.35) | 4032.39 (3479.55 - 4654.75) | 578.97 (392.03 - 798.66) | -0.11 (-0.12 - -0.1) | 16027.4 (13535.74 - 18427.82) | 3494.45 (2731.45 - 4408.8) | 23466.35 (20038.6 - 26843.93) | 3337.13 (2617.48 - 4193.15) | -0.14 (-0.15 - -0.13) | 488.53 (123.46 - 1342.57) | 146.19 (34.35 - 398.87) | 659.53 (153.72 - 1904.19) | 136.05 (30.57 - 384.53) | -0.16 (-0.26 - -0.06) | 10617.77 (4883.51 - 22553.76) | 2527.14 (1091.68 - 5729.58) | 15564.22 (7060.31 - 36511.77) | 2419.09 (1040.68 - 5666.99) | -0.12 (-0.18 - -0.06) |

**Abbreviations**: UI, uncertainty interval; ASR, age-standerised rate per 100,000; CI, confidence interval; DALYs, disability-adjusted life-year; EAPC, estimated annual percentage change.

**Table S10 Decomposition of change in incidence globally and by SDI quintile, 1990 to 2021**

| **Cause** | **Location** | **Overall difference** | **Aging** | **Population**  **growth** | **Epidemiological**  **changes** |
| --- | --- | --- | --- | --- | --- |
| LOMS | Global | 2410.17 | -6.50  (-0.27%) | 2971.268  (123.28%) | -554.604  (-23.01%) |
|  | High SDI | 412.08 | -17.42  (-4.23%) | 629.027  (152.65) | -199.531  (-48.42%) |
|  | High-middle SDI | 361.76 | -0.18  (-0.05%) | 617.201  (170.61%) | -255.268  (-70.56%) |
|  | Middle SDI | 1015.27 | 6.3  (00.62%) | 1074.911  (105.87%) | -65.939  (-6.49%) |
|  | Low-middle SDI | 488.52 | -1.11  (-0.23%) | 483.356  (98.94%) | 6.275  (1.28%) |
|  | Low SDI | 131.86 | -0.002  (0%) | 137.241  (104.08%) | -5.383  (-4.08%) |
| PD | Global | 812389.2 | 39890.66  (4.91%) | 548898.6  (67.57%) | 223600  (27.52%) |
|  | High SDI | 190470.9 | 11387.8  (5.98%) | 122774.7  (64.46%) | 56308.42  (29.56%) |
|  | High-middle SDI | 215725.7 | 12482.37  (5.79%) | 136890.2  (63.46%) | 66353.2  (30.76%) |
|  | Middle SDI | 290676.5 | 17753.71  (6.11%) | 187188.1  (64.40%) | 85734.7  (29.49%) |
|  | Low-middle SDI | 91924.32 | 5783.591  (6.29%) | 69864.52  (76%) | 16276.21  (17.71%) |
|  | Low SDI | 23158.33 | 1128.032  (4.87%) | 18813.71  (81.24%) | 3216.595  (13.89%) |
| AD and other dementias | Global | 5784198 | 812143.4  (14.04%) | 4776735  (82.58%) | 195319.5  (3.38%) |
|  | High SDI | 1494354 | 327571.9  (21.92%) | 1254338.9  (83.94%) | -87556.5  (-5.86%) |
|  | High-middle SDI | 1544977 | 249594  (16.16%) | 1096074  (70.94%) | 199308.7  (12.9%) |
|  | Middle SDI | 1966453 | 268435.9  (13.65%) | 1527160  (77.66%) | 170857.7  (8.69%) |
|  | Low-middle SDI | 607645.3 | 75120.9  (12.36%) | 554413.4  (91.24%) | -21889  (-3.6%) |
|  | Low SDI | 166818.1 | 17643.63  (10.58%) | 160148.421  (96%) | -10974  (-6.58%) |

**Abbreviations**: SDI, sociodemographic index; LOMS, late-onset multiple sclerosis.

**Table S11 Decomposition of change in DALYs globally and by SDI quintile, 1990 to 2021**

| **Cause** | **Location** | **Overall difference** | **Aging** | **Population**  **growth** | **Epidemiological**  **changes** |
| --- | --- | --- | --- | --- | --- |
| LOMS | Global | 242674.06 | -2188.464  (-0.9%) | 249169.9  (102.68%) | -4307.36  (-1.77%) |
| High SDI | 161044.8 | -4974.27  (-3.09%) | 128733.5  (79.94%) | 37285.57  (23.15%) |
| High-middle SDI | 30607.88 | -893.163  (-2.92%) | 44242.52  (144.55%) | -12741.5  (-41.63%) |
| Middle SDI | 33298.51 | -204.902  (-0.62%) | 23965.68  (71.97%) | 9537.738  (28.64%) |
| Low-middle SDI | 14563.45 | -117.936  (-0.81%) | 9666.027  (66.37%) | 5015.357  (34.44%) |
| Low SDI | 3007.53 | -24.718  (-0.82%) | 2288.004  (76.08%) | 744.241  (24.75%) |
| PD | Global | 4419168 | 422116.9  (9.55%) | 3560910  (80.58%) | 436141.6  (9.87%) |
| High SDI | 1139259 | 143553.3  (12.6%) | 800406.3  (70.26%) | 195299.9  (17.14%) |
[truncated: 59,135 more chars]
